# Development and validation of prediction models for neurocognitive disorders in adult patients admitted to the ICU with sleep disturbance

**DOI:** 10.1111/cns.13772

**Published:** 2021-12-23

**Authors:** Yun Li, Lina Zhao, Ye Wang, Xizhe Zhang, Jiannan Song, Qi Zhou, Yi Sun, Chenyi Yang, Haiyun Wang

**Affiliations:** ^1^ The Third Central Clinical College of Tianjin Medical University Tianjin China; ^2^ Tianjin Key Laboratory of Extracorporeal Life Support for Critical Diseases Artificial Cell Engineering Technology Research Center Tianjin Institute of Hepatobiliary Disease Tianjin China; ^3^ Department of Anesthesiology Chifeng Municipal Hospital Chifeng Clinical Medical College of Inner Mongolia Medical University Chifeng China; ^4^ Emergency Department Peking Union Medical College Hospital Peking Union Medical College Chinese Academy of Medical Sciences Beijing China; ^5^ Department of Anesthesiology The Third Central Hospital of Tianjin The Third Central Clinical College of Tianjin Medical University Nankai University Affinity The Third Central Hospital Tianjin Key Laboratory of Extracorporeal Life Support for Critical Diseases Artificial Cell Engineering Technology Research Center Tianjin Institute of Hepatobiliary Disease Tianjin China

**Keywords:** ICU, LASSO, logistic regression, neurocognitive disorders, nomograms, sleep disturbance

## Abstract

**Background:**

Neurocognitive disorders (NCDs) and sleep disturbance are highly prevalent in the perioperative period and intensive care unit (ICU). There has been a lack of individualized evaluation tools designed for the high‐risk NCDs in critically ill patients with sleep disturbance.

**Objectives:**

The aim of this study was to develop and validate prediction models for NCDs among adult patients with sleep disturbance.

**Methods:**

The R software was used to analyze the dataset of adult patients admitted to the ICU with sleep disturbance, who were diagnosed following the codes of the International Classification of Diseases, 9th Revision (ICD‐9) and 10th Revision (ICD‐10) using the MIMIC‐IV database. We used logistic regression and LASSO analyses to identify important risk factors associated with NCDs and develop nomograms for NCDs predictions. We measured the performances of the nomograms using the bootstrap resampling procedure, sensitivity, specificity of the receiver operating characteristic (ROC), area under the ROC curves (AUC), and decision curve analysis (DCA).

**Results:**

The prediction models shared the 10 risk factors (age, gender, midazolam, morphine, glucose, diabetes diseases, potassium, international normalized ratio, partial thromboplastin time, and respiratory rate). Cardiovascular diseases were included in the logistic regression, the sensitivity was 74.1%, and specificity was 64.6%. When platelet and Glasgow Coma Score (GCS) were included and cardiovascular diseases were removed in the LASSO prediction model, the sensitivity was 86.1% and specificity was 82.8%. Discriminative abilities of the logistic prediction and LASSO prediction models for NCDs in the validation set were evaluated as the AUC scores, which were 0.730 (95% CI 0.716–0.743) and 0.920 (95% CI 0.912–0.927). Net benefits of the prediction models were observed at threshold probabilities of 0.567 and 0.914.

**Conclusions:**

The LASSO prediction model showed better performance than the logistic prediction model and should be preferred for nomogram‐assisted decisions on clinical risk management of NCDs among adult patients with sleep disturbance in the ICU.

## Graphical abstract

Development and validation of prediction models for neurocognitive disorders in adult patients admitted to the ICU with sleep disturbance. They are logistic prediction models and LASSO prediction models. The LASSO prediction model showed better performance than the logistic prediction model and should be preferred for nomogram‐assisted decisions on clinical risk management of NCDs among adult patients with sleep disturbance in the ICU.

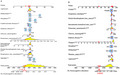

## 1 INTRODUCTION

Neurocognitive disorders (NCDs), including delirium, mild cognitive impairment (MCI), and dementia, pose a grave public health challenge.^1^, ^2^ NCDs are commonly prevalent among perioperative patients with sleep disturbance,^3^ especially those admitted to the intensive care unit (ICU).^4^, ^5^ Coupled with the impact of sleep disturbance,^6^, ^7^ NCDs in the ICU are associated with increased mortality and even continue to deteriorate cognition and sleep after ICU discharge.^8^ In the ICU, sleep disturbance is widespread in adult patients, with some studies reporting a prevalence of sleep disturbance in the range of more than 50%.^4^, ^9^, ^10^ Notably, NCDs are closely associated with sleep disturbance and are highly prevalent among perioperative patients in the ICUs.^11^, ^12^, ^13^ Adult ICU patients with sleep disturbance have been suffered from serious yet still largely unresolved NCDs.^4^, ^14^ Risk assessment tools for ICU delirium are available,^15^, ^16^ not specifically for ICU patients with sleep disturbance. Research on MCI and dementia in the ICU, especially their risk assessment, is rarely available, not to mention that in ICU patients with sleep disturbance. There is currently a lack of an overall risk assessment system of NCDs specifically for ICU patients with sleep disturbance, including those perioperatively admitted to the ICU.

Accumulating evidence indicates potential bidirectional regulatory connections between sleep disturbance and cognitive impairment.^5^, ^14^, ^17^, ^18^, ^19^, ^20^ Impaired amyloid‐β clearance, increased tau levels, aggravation of inflammation, impaired synaptic plasticity, and changes in neurotransmitters, may have important roles underlying the potential association between sleep disturbance and NCDs.^18^, ^21^ Additionally, evidence from epidemiological and clinical studies reveals that delirium, MCI, and dementia have a close correlation with sleep disturbance. Besides, sleep disturbance is one of the diagnostic criteria for delirium.^22^ Although mechanistic studies have identified novel biomarkers with high sensitivity and specificity, they lack popularity in clinical practice. Most importantly, no potential clinical biomarkers associated with NCDs have been identified in patients with sleep disturbance. Sleep disturbance is likely to be a precursory and concomitant symptom of NCDs and is likely to share clinical markers with NCDs. A study on the prediction for postoperative sleep disturbance indicated that gender, midazolam, and sufentanil were important clinical markers independently associated with postoperative sleep disturbance.^23^ Our previous study on predicting sleep disturbance in ICU patients suggested that some biomarkers from routine laboratory tests also were independent predictors.^24^ In the absence of NCD‐related prediction markers available in patients with sleep disturbance, based on clinical data including the previously established prediction models for sleep disturbance, developing the overall evaluation system of NCDs risk has important clinical value for ICU patients with sleep disturbance. At present, there are no prediction models for NCDs in ICU patients with sleep disturbance, though there have been many kinds of research on neurocognitive consequences of sleep disturbance. This situation may increase the risk of NCDs in ICU patients with sleep disturbance.

As recommended,^12^ early diagnostic prediction models can effectively assist healthcare providers in estimating the risk of a specific disease or present condition. This provides theoretical support for the early prediction of NCDs in patients with sleep disturbance. Based on the transparent reporting of a multivariable prediction model for individual prognosis or diagnosis,^25^ our study design specifically focused on the prediction of NCDs in adult patients with sleep disturbances, who were admitted to the ICU. We aimed to develop and internally validate novel models for the prediction of NCDs in adult ICU patients with sleep disturbance using the MIMIC‐IV database.

## 2 MATERIALS AND METHODS

### 2.1 Data source

This retrospective cohort‐based study was conducted using the MIMIC‐IV database (version 1.0), the most recent update after MIMIC‐III.^26^ It is a longitudinal, large, single‐center database that incorporates contemporary critical care data for over 60,000 patients admitted to ICUs at the Beth Israel Deaconess Medical Center between 2008 and 2019. Patient identifiers in MIMIC‐IV were removed to maximize patient privacy protection. MIMIC‐IV is a publicly available clinical database that allows for data sharing only after passing the Collaborative Institutional Training Initiative examination. One author who had access to MIMIC‐IV from our study group specialized in data extraction from this database. We constructed a clinical dataset of patient hospitalization information, including their demographics, laboratory measurements, medications, and other health‐related information, based on the structured query language for data extraction.

### 2.2 Participants

We included adult patients (age ≥18 years) with sleep disturbance admitted to the ICUs and extracted their data from the MIMIC‐IV. According to the third edition of the International Classification of Sleep Disorders, sleep disturbance was divided into seven subtypes including insomnia, sleep‐related breathing disorders, central disorders of hypersomnolence, circadian rhythm sleep–wake disorder, parasomnias, sleep‐related movement disorders, and other sleep disorders (Appendix S1). Furthermore, sleep disturbance was determined following diagnostic codes of the International Classification of Diseases, 9th revised (ICD‐9) and 10th revised (ICD‐10) editions. Only patients who met the criteria of the ICU duration of stay greater than 24 h were included.

### 2.3 Outcomes

The primary outcomes included the major NCDs during ICUs stay. Based on the recommendations in the 5th Edition of the Diagnostic and Statistical Manual of Mental Disorders,^22^ we included delirium, MCI, and dementia as the major NCDs. The information on included NCDs was extracted according to the ICD‐9 code (Appendix S2). Given that neuropsychiatric diseases may also accompany cognitive impairment, we excluded specific neuropsychiatric diseases including craniocerebral diseases, meningitis, encephalitic diseases, epilepsy, cerebrovascular diseases, encephalopathy, mental and neurological disorders, alcoholism or drug abuse, and other common neuropsychiatric disorders (Appendix S3).

### 2.4 Predictors of NCDs

For the prediction of NCDs, clinical and biological variables were extracted from the MIMIC‐IV. For patients who had been admitted to ICUs multiple times, we only used the information at their first ICU admission. Baseline data, vital signs, and system scores only within the first 24 h of ICU admission were included in the analysis. Additionally, only the first recorded data of laboratory measurements were analyzed. The variables included were as follows: (1) patient demographics, including age, gender, admission type, marital status, and ethnicity; (2) vital signs, including heart rate, blood pressure, respiratory rate, temperature, pulse oxygen saturation (SpO_2_), and partial pressure of carbon dioxide (pCO_2_); (3) laboratory parameters, including creatinine level, blood urea nitrogen, hemoglobin, platelet count, partial thromboplastin time, international normalized ratio, prothrombin time, white blood cell count, lymphocyte, neutrophils, sodium, potassium, pH, and glucose; (4) comorbidities were identified according to the ICD‐9 and ICD‐10 codes, including Charlson Comorbidity Index (CCI), hypertension, diabetes, cardiovascular diseases, chronic pulmonary diseases, liver diseases, kidney diseases, and immunodepression; (5) medications, including analgesics and sedative drugs (morphine, midazolam, propofol, etomidate, dexmedetomidine, and haloperidol) and cardiovascular drugs (norepinephrine, epinephrine, dobutamine, and dopamine); (6) system scores, including Simplified Acute Physiology Score II (SAPS II), Sequential Organ Failure Assessment (SOFA) score, and Glasgow Coma Score (GCS); (7) treatment strategies, including mechanical ventilation and renal replacement therapy, along with the length of ICU stay.

### 2.5 Sample size

After using the inclusion and exclusion criteria, a total of 4,895 eligible patients from MIMIC‐IV were enrolled in our cohort. This cohort was randomly divided in a ratio of 7:3 into two groups, namely the primary and validation cohorts. Based on the set standard of 10 events per candidate predictor parameters in the machine learning algorithm,^25^ the 60 predictors included in this study required at least 600 individuals with their respective events. Also, considering other sample size requirements for developing a clinical prediction model, 3,916 patients in the primary cohort met the standard optimal sample size for further statistical analyses.

### 2.6 Data cleaning and missing management

Firstly, we searched the “*icu*stays” table in the “*icu*” module of the MIMIC‐IV database. The “*icu*” module contains data sourced from the clinical information system of MetaVision. The MetaVision table is denormalized to create a star schema, where the “*icu*stays” and “id‐items” tables are linked to a set of data tables, all of which are suffixed with an "event". The data recorded in the “*icu*” module included venous and fluid inputs (input events), patient outputs (output events), procedures (program events), information recorded as dates or times (date time events), and other patient chart information (chart events). All event tables had a “stay_id” column to identify related ICU patients from “*icu*stays,” and an “item‐id” column for identification of concepts recorded in “id‐items”.^26^ Subsequently, information for 69,619 patients who were admitted to the ICU was retrieved. Further, we only included the “stay_id” at the first hospitalization and excluded the patients who were repeatedly admitted to the ICU. Finally, a total of 50,048 patients were enrolled in the first ICU admission. Subsequently, we merged and processed other forms according to the patient's “subject_id” number. Throughout the process, we removed patients whose vital signs and laboratory parameter information were missing value more than 10% and lacked the ICD diagnostic codes. Although missing data is frequent in data extraction, MIMIC‐IV version 1.0 has addressed this issue by updating patient data and improving the separate and combined uses of module datasets. Finally, for quality control of missing data, a data profiling report (Data S1) was used to analyze all predictor parameters. The percentage of missing values of calcium (2.43%), bun (1.86%), pCO_2_ (1.65%), and the other variables were <2%. Additionally, we processed the variables with missing values through multiple imputations and filled in the missing data using their predictive values.

### 2.7 Statistical analyses

Data normality was verified by the Shapiro–Wilk test. Continuous variables were presented as the mean ±standard deviation (for normally distributed data) or median [interquartile range, IQR] (for non‐normally distributed data) and as the frequency [percentage] (for categorical variables). All continuous variables in the dataset showed skewed distribution. Baseline characteristics between NCDs and non‐NCDs groups in the primary and validation cohorts, respectively, were compared using the Mann–Whitney *U* test or the Kruskal–Wallis test for continuous variables with non‐normal distributions or heterogeneity, and the Pearson Chi‐squared test for categorical variables.

According to significant differences (*p* < 0.05) in baseline characteristics in the primary cohort, potential variables were used in the further multivariate logistic regression and LASSO regression. Odds ratio (OR) and 95% confidence interval (CI) in the logistic regression and coefficient in the LASSO regression were calculated to identify significantly associated independent risk predictors for NCDs. Based on the results of the LASSO regression and multivariate logistic analyses, two nomograms were constructed. The final prediction models were presented as nomograms, which were the main process of developing prediction models for individual NCD diagnoses in this study. Moreover, the calibration, discrimination, and clinical utility of each nomogram were evaluated. The bootstrap resampling procedure with 1,000 repetitions was used to internally calibrate the nomogram in the validation cohort, and a calibration curve was plotted to analyze the accordance between the predicted probability using the nomogram and actual occurrence. The discriminative ability of the nomogram was analyzed using the receiver operating characteristic (ROC) curves and the area under the ROC curves (AUC), which were used along with the calibration curve to evaluate the predictive ability of the prediction models. Decision curve analysis (DCA) was used to assess the clinical utility of the prediction models for decision‐making, and we plotted corresponding net benefits for a range of risk thresholds.

Statistical analyses were performed using the R software (version 3.4.3), and statistical significance was defined as a two‐tailed *p*‐value <0.05.

## 3 RESULTS

### 3.1 Participants

We analyzed clinical data of ICU patients obtained from the MIMIC‐IV database, a total of 69,619 individuals between 2008 and 2019. Of the 50,048 patients admitted to the ICU for the first time, 5,582 were diagnosed with sleep disturbances. All patients having craniocerebral diseases (*n* = 345), mental illness (*n* = 235), alcoholism or drug abuse (*n* = 40), age <18 years old (*n* = 32), and ICU stay <24 h (*n* = 35) were excluded. Finally, a total of 4,895 patients with sleep disturbance were divided into the primary cohort (3,916 individuals) and the validation cohort (979 individuals) in this study; among them, 1,391 developed NCDs during ICU stay (Appendix S4).

A total of 1,110 patients in the primary cohort and 281 in the validation cohort exhibited NCDs. In the primary cohort, patients in NCDs group were younger than those in non‐NCDs group (61.3 (48.5–73.7) vs. 63.7 (53.4–79.0); *p* = 0.001). Baseline demographics, including gender (female), admission type (emergency), marital status (married), and ethnicity (white) also showed significant differences between the two groups. There were significant differences in all vital signs (all *p* < 0.001). Notably, patients in NCDs group had lower creatinine levels (1.4 (0.9–1.7) vs. 1.6 (1.0–1.6), *p* < 0.001), potassium levels (4.1 (3.9–4.1) vs. 4.3 (4.0–4.6), *p* < 0.001), and glucose levels (123 (102–127) vs. 125 (111–130), *p* < 0.001); shorter partial thromboplastin time (34.7 (27.6–36.9) vs. 36.9 (28.3–36.9), *p* = 0.006) and international normalized ratio (1.6 (1.2–1.8) vs. 1.6 (1.2–2.0), *p* < 0.001); higher blood urea nitrogen levels (26 (17–29) vs. 24 (19–29), *p* = 0.002), hemoglobin levels (11.7 (10.4–13.4) vs. 11.6 (10.5–12.3), *p* = 0.001), platelet counts (226 (197–243) vs. 223 (195–232), *p* = 0.008), and higher pH values (7.41 (7.38–7.44) vs. 7.38 (7.35–7.42), *p* < 0.001), than those in non‐NCDs group. The two groups significantly differed in terms of diabetes and cardiovascular disease incidences, but not for CCI. Compared with the non‐NCDs group, patients having greater use of morphine (678 (61.1%) vs. 970 (34.6%), *p* < 0.001), midazolam (601 (54.1%) vs. 716 (25.5%), *p* < 0.001), and propofol (256 (23.1%) vs. 561 (20.0%), *p* = 0.036) were more likely to suffer from NCDs. Moreover, the NCDs group had higher SAPSII scores (36 (31–45) vs. 35 (32–39), *p* < 0.001) and SOFA scores (9 (3–10) vs. 6 (3–9), *p* < 0.001), while lower GCS (14 (12–15) vs. 15 (15–15), *p* < 0.001) values as compared to the non‐NCDs group. Baseline characteristics and details at first admission to ICU for all participants are shown in Table 1.

**TABLE 1:** Characteristics of patients in the primary and validation cohorts

	Primary cohort			Validation cohort		
	NCDs group, *n* = 1,110	Non‐NCDs group, *n* = 2,806	*p*	NCDs group, *n* = 281	Non‐NCDs group, *n* = 698	*p*
Age	61.3 (48.5–73.7)	63.7 (53.4–79.0)	0.001	61.6 (49.6–76.1)	63.5 (53.8–72.8)	0.359
Gender, *n* (%)						
Female	563 (50.7)	1,152 (41.1)	<0.001	152 (54.1)	293 (42)	0.001
Male	547 (49.3)	1,654 (58.9)		129 (45.9)	405 (58)	
Admission_type (%)						
Emergency	601 (54.1)	1,390 (49.5)	<0.001	151 (53.7)	332 (47.6)	<0.001
Observation	296 (26.7)	581 (20.7)		64 (22.8)	159 (22.8)	
Elective	39 (3.5)	96 (3.4)		9 (3.2)	26 (3.7)	
Urgent	70 (6.3)	221 (7.9)		32 (11.4)	56 (8.0)	
Others	104 (9.4)	518 (18.5)		25 (8.9)	125 (17.9)	
Marital_status (%)						
Married	455 (41.0)	1,512 (53.9)	<0.001	109 (38.8)	377 (54.0)	<0.001
Single	452 (40.7)	792 (28.2)		108 (38.4)	203 (29.1)	
Divorced	101 (9.1)	191 (6.8)		26 (9.3)	55 (7.9)	
Ethnicity (%)						
White	857 (77.2)	2,030 (72.3)	0.001	221 (78.6)	512 (73.4)	0.185
Black	122 (11)	434 (15.5)		30 (10.7)	102 (14.6)	
Others	131 (11.8)	342 (12.2)		30 (10.7)	84 (12.0)	
Vital signs, [IQR]						
Heart rate (bpm)	110 (101–123)	104 (100–108)	<0.001	98 (95–102)	98 (95–102)	0.434
Diastolic blood pressure (mmHg)	47 (44–50)	45 (42–48)	<0.001	44 (41–47)	45 (41–47)	0.725
Systolic blood pressure (mmHg)	94 (81–102)	93 (88–97)	<0.001	94 (92–98)	94 (92–97)	0.725
Respiratory rate (bpm)	25 (22–29)	27 (23–30)	<0.001	25 (23–28)	26 (23–28)	0.564
Temperature (℃)	37.3 (37.1–37.6)	37.2 (36.9–37.6)	<0.001	37.1 (36.9–37.4)	37.2 (36.9–37.4)	0.237
SpO_2_ (mmHg)	129 (88–133)	123 (99–127)	<0.001	122 (88.5–127)	123 (91.8–128)	0.546
pCO_2_ (mmHg)	46 (40–49)	41 (37–44)	<0.001	41 (38–44)	42 (39–44)	0.225
Laboratory parameters, [IQR]						
Creatinine (mg/dL)	1.4 (0.9–1.7)	1.6 (1.0–1.6)	<0.001	1.6 (0.9–1.8)	1.6 (1.0–1.8)	0.674
Blood urea nitrogen (mg/dL)	26 (17–29)	24 (19–29)	0.002	27 (17–30)	28 (19–29.3)	0.399
Hemoglobin (g/dL)	11.7 (10.4–13.4)	11.6 (10.5–12.3)	0.001	11.1 (10.8–12.1)	11.1 (10.9–11.6)	0.417
Platelet (×10^9^/L)	226 (197–243)	223 (195–232)	0.008	228 (218–247)	227 (190–230)	0.001
Partial thromboplastin time (s)	34.7 (27.6–36.9)	36.9 (28.3–36.9)	0.006	32.2 (27.4–36.8)	36.6 (28.4–36.9)	0.002
International normalized ratio	1.6 (1.2–1.8)	1.6 (1.2–2.0)	<0.001	1.4 (1.2–1.6)	1.4 (1.2–1.7)	0.090
Prothrombin time (s)	14.5 (12.3–15.4)	14.7 (12.5–15.4)	0.872	14.3 (12.2–15.4)	14.7 (12.5–15.5)	0.059
White blood cell count (×10^9^/L)	11.1 (8.2–13.5)	11.5 (8.8–12.2)	0.678	11.0 (8.6–11.2)	11.0 (8.8–11.2)	0.606
Lymphocyte (%)	21.6 (15.3–26.7)	21.6 (15.0–26.0)	0.710	21.6 (15.8–26.7)	21.6 (14.0–25.1)	
Neutrophils (%)	69 (62.5–75.4)	69 (63.7–75.5)	0.468	69 (62.5–75.0)	69 (63.7–77.5)	
Sodium (mmol/L)	138 (137–139)	138 (138–138)	0.206	136 (134–139)	137 (134–139)	0.550
Potassium (mmol/L)	4.1 (3.9–4.1)	4.3 (4.0–4.6)	<0.001	4.2 (3.9–4.4)	4.2 (3.8–4.3)	0.240
pH	7.41 (7.38–7.44)	7.38 (7.35–7.42)	<0.001	7.39 (7.36–7.41)	7.38 (7.36–7.40)	0.645
Glucose (mg/dL)	123 (102–127)	125 (111–130)	<0.001	123 (107–127)	123 (108–127)	0.992
Comorbidity, *n* (%)						
CCI	4 (2–6)	4 (3–6)	<0.001	4 (3–7)	4 (3–7)	0.768
Hypertension	608 (54.8)	1,595 (56.8)	0.253	146 (52.0)	388 (55.6)	0.321
Diabetes	268 (24.1)	924 (32.9)	<0.001	66 (23.5)	241 (30.7)	0.001
Cardiovascular diseases	225 (20.3)	661 (23.6)	0.028	55 (19.6)	181 (25.9)	<0.001
Chronic pulmonary diseases	310 (27.9)	718 (25.6)	0.136	81 (28.8)	180 (25.8)	0.119
Liver. diseases	81 (7.3)	213 (7.6)	0.788	14 (5.0)	54 (7.7)	0.781
Kidney. diseases	148 (13.3)	439 (15.6)	0.074	38 (13.5)	129 (18.5)	0.074
Immunosuppressive	15 (10.4)	315 (11.2)	0.461	23 (8.2)	80 (11.5)	0.136
Medications, *n* (%)						
Analgesic and sedative drugs (%)						
Morphine	678 (61.1)	970 (34.6)	<0.001	132 (47)	274 (39.3)	0.031
Midazolam	601 (54.1)	716 (25.5)	<0.001	135 (48)	273 (39.1)	0.012
Propofol	256 (23.1)	561 (20.0)	0.036	111 (39.5)	298 (42.7)	0.390
Etomidate	215 (19.4)	563 (20.1)	0.657	96 (34.2)	217 (31.1)	0.364
Dexmedetomidine	189 (17.0)	516 (18.4)	0.333	116 (41.3)	298 (42.7)	0.721
Haloperidol	129 (11.6)	365 (13.0)	0.262	48 (17.1)	106 (15.2)	0.497
Cardiovascular system drugs (%)						
Norepinephrine	74 (6.7)	173 (6.2)	0.560	15 (5.3)	45 (6.4)	0.559
Epinephrine	13 (1.2)	49 (1.7)	0.255	1 (0.4)	9 (1.3)	0.297
Dobutamine	7 (0.6)	20 (0.7)	1.000	0 (0)	3 (0.4)	0.562
Dopamine	17 (1.5)	34 (1.2)	0.436	4 (1.4)	6 (0.9)	0.485
Score system, [IQR]						
SAPSII	36 (31–45)	35 (32–39)	<0.001	34 (31.5–37)	34 (32–38)	0.695
SOFA	9 (3–10)	6 (3–9)	<0.001	6 (3.5–9)	6 (4–9)	0.764
GCS	14 (12–15)	15 (15–15)	<0.001	13 (11–14)	13 (11–15)	0.612
Treatment measures						
Mechanical ventilation, *n* (%)	51 (4.6)	136 (4.8)	0.803	13 (4.6)	29 (4.2)	0.729
Renal replacement therapy, *n* (%)	12 (1.1)	44 (1.6)	0.297	11 (3.9)	43 (6.2)	0.215
Length of stay in ICU, days	1.0 (1.0–3.0)	1.0 (1.0–3.0)	0.007	1.0 (1.0–3.0)	1.0 (1.0–3.0)	0.494
Hospital mortality, *n* (%)	20 (1.8)	42 (1.5)	0.480	3 (1.1)	9 (1.3)	1.000

Note

*p* < 0.05 means significant different.

Abbreviations: CCI, Charlson Comorbidity Index; GCS, Glasgow Coma Scale; ICU, intensive care unit; NCDs, neurocognitive disorders; pCO_2_, partial pressure of carbon dioxide; SAPSII, simplified acute physiology score; SOFA, sequential organ failure assessment; SpO_2_, pulse oxygen saturation.

### 3.2 Model construction

The prediction models for NCDs were developed using 1,110 major NCD events in the primary cohort. Clinical and biological variables with *p* < 0.05 (Table 1) were assessed for the development of the new NCDs risk prediction models. The variables were further examined using multivariate logistic regression of NCDs risk. The strongest predictor was midazolam used with an OR of 2.820 (95% CI 2.411–3.229, *p* < 0.001). Predictors including gender (OR 1.271, 95% CI 1.109–1.1458, *p* = 0.001), respiratory rate (OR 1.041, 95% CI 1.027–1.055, *p* < 0.001), partial thromboplastin time (OR 1.004, 95% CI 1.001–1.008, *p* = 0.020), international normalized ratio (OR 1.104, 95% CI 1.015–1.201, *p* = 0.021), potassium (OR 2.191, 95% CI 1.910–2.514, *p* < 0.001), glucose (OR 1.002, 95% CI 1.000–1.003, *p* = 0.034), diabetes diseases (OR 1.430, 95% CI 1.223–1.671, *p* < 0.001), cardiovascular diseases (OR 1.303, 95% CI 1.104–1.602, *p* = 0.002), and morphine use (OR 2.144, 95% CI 1.815–2.532, *p* < 0.001) were also significant independent risk factors of NCDs. Age (OR 1.003, 95% CI 0.997–1.008, *p* = 0.314) was also considered as an important variable (Table 2). A total of 50 potential risk variables were further included in the LASSO analysis (Appendix S5). A total of 12 independently associated risk variables including gender, platelet count, glucose, potassium, international normalized ratio, partial thromboplastin rate, respiratory rate, age, diabetes disease, GCS, morphine use, and midazolam use were selected through the LASSO analysis (Appendix S6). Subsequently, 11 risk predictors from the logistic regression and 12 variables from the LASSO analyses were included in the final prediction models.

**TABLE 2:** Multivariate logistic analysis of risk factors to NCDs

	Multivariate analysis			
	OR	95.0% CI		*p*‐values
		Lower	Upper	
Age	1.003	0.997	1.008	0.314
Gender, *n* (%)	1.271	1.109	1.458	0.001
Female				
Male				
Admission_type, *n* (%)				
Emergency				0
Observation	1.380	0.948	2.009	0.094
Elective	1.608	1.210	2.138	0.001
Urgent	0.985	0.833	1.164	0.857
Others	2.087	1.657	2.628	<0.001
Ethnicity, *n* (%)				
White				0
Black	1.737	1.426	2.117	<0.001
Others	1.096	0.884	1.360	0.403
Heart rate (bpm)	0.977	0.973	0.982	<0.001
Diastolic blood pressure (mmHg)	0.988	0.979	0.997	0.008
Systolic blood pressure (mmHg)	0.141	0.990	1.001	0.132
Respiratory rate (bpm)	1.041	1.027	1.055	<0.001
Temperature (℃)	0.981	0.875	1.100	0.745
SpO_2_ (mmHg)	0.985	0.972	0.999	0.032
pCO_2_ (mmHg)	0.949	0.943	0.956	<0.001
Creatinine (mg/dL)	0.918	0.875	0.963	<0.001
Blood urea nitrogen (mg/dL)	0.999	0.995	1.004	0.767
Hemoglobin(g/dL)	1.026	0.990	1.062	0.156
Platelet (×10^9^/L)	0.998	0.997	0.999	<0.001
Partial thromboplastin time (s)	1.004	1.001	1.008	0.020
International normalized ratio	1.104	1.015	1.201	0.021
Potassium (mmol/L)	2.191	1.910	2.514	<0.001
pH	0.004	0.001	0.010	0.003
Glucose (mg/dL)	1.002	1.000	1.003	0.034
Diabetes diseases, *n* (%)	1.430	1.223	1.671	<0.001
Cardiovascular diseases, *n* (%)	1.303	1.104	1.602	0.002
Morphine, *n* (%)	2.144	1.815	2.532	<0.001
Midazolam, *n* (%)	2.820	2.411	3.229	<0.001
Propofol, *n* (%)	0.661	0.558	0.782	<0.001
SAPS II	0.981	0.973	0.989	<0.001
SOFA	0.967	0.946	0.988	0.002

### 3.3 Model specifications

**FIGURE 1:**
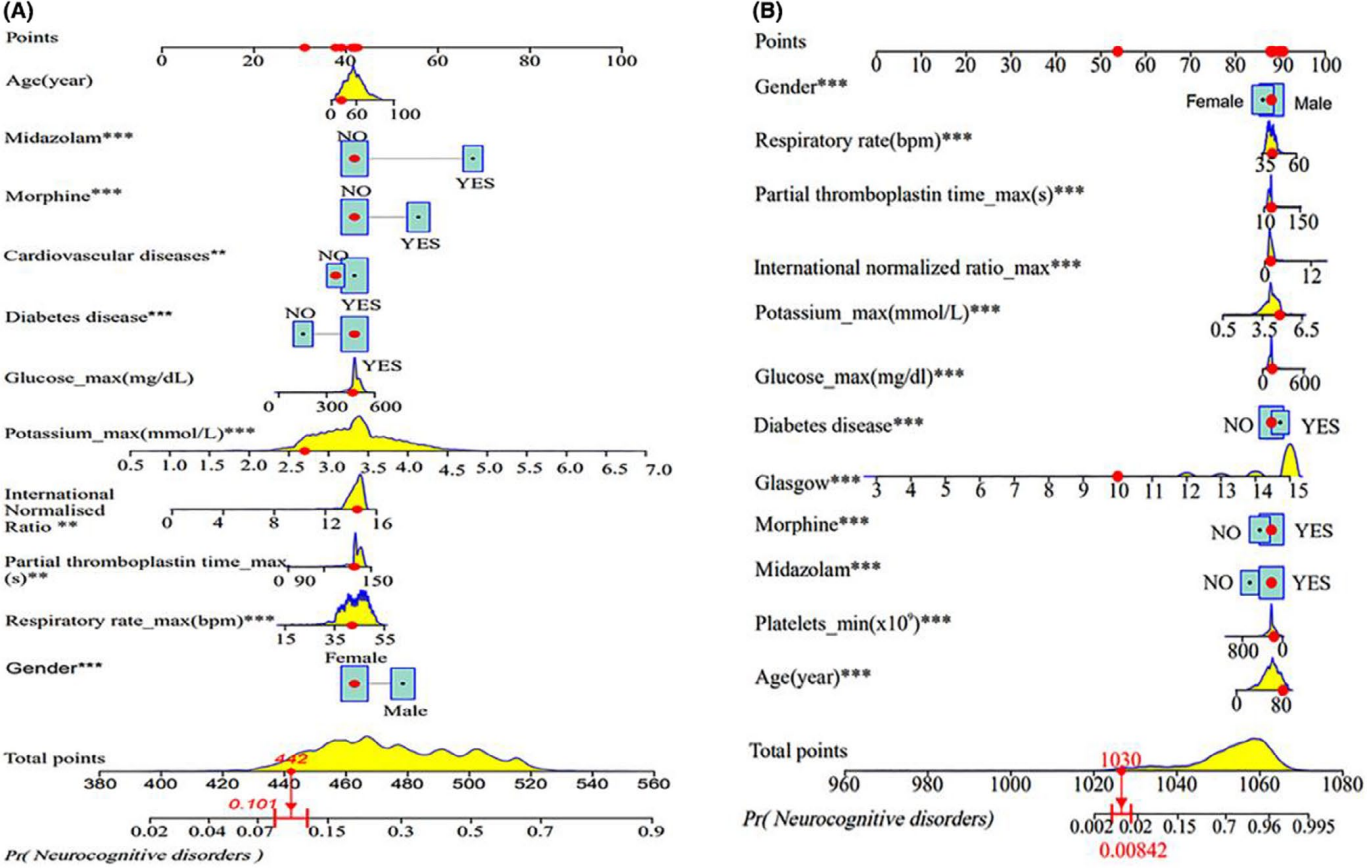
Nomograms for the prediction models. (A) Nomograms for the logistic prediction model. On a baseline line of 0–100 points, score lines were assigned to age, midazolam, opioids, cardiovascular diseases, diabetes diseases, glucose, potassium, international normalized ratio, partial thromboplastin time, respiratory rate, and gender, all of which had their own score ranges. (B) Nomograms for the LASSO prediction model score lines were assigned to gender, platelet, glucose, potassium, international normalized ratio, partial thromboplastin time, respiratory rate, age, diabetes, GCS, morphine, and midazolam; adding an example for Figure A and Figure B (red dot), the red dots on each indicator in the figure, it is the data value of each indicator of the patient; draw a vertical line up to the point line at the position of the red dot of each indicator; the score of the vertical line position is the score of the red dot of each indicator. Total score of all indicator scores and find the position of the total score on the total points line. Draw a vertical line to the Pr (neurocognitive disorders) line according to the total score position, which is the incidence of neurocognitive disorders in the patient. The total point of the nomogram was 442, with the corresponding probability of 10.1% for NCDs in (A). The total scores of the nomogram were 1,030 corresponding to the probability of 0.842% in (B)

**FIGURE 2:**
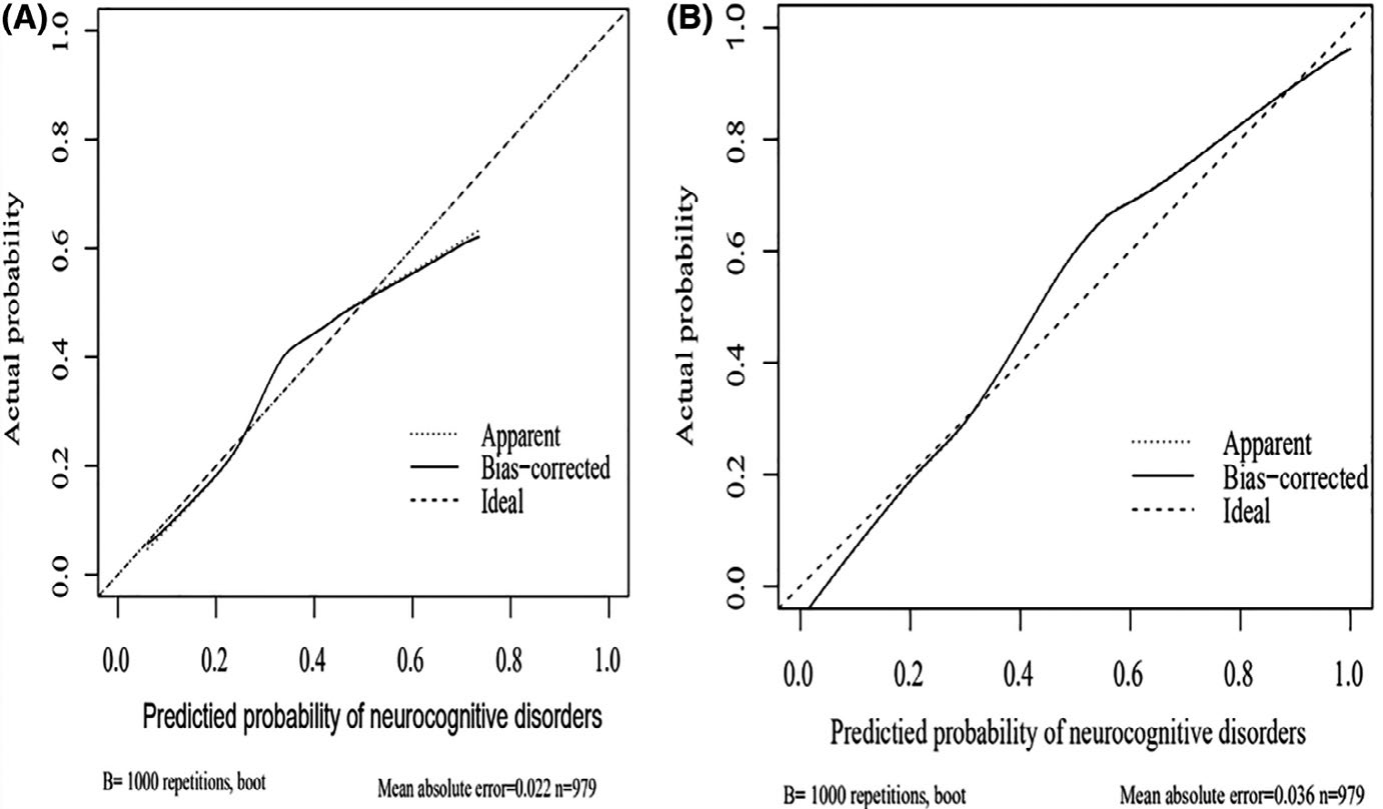
Calibration plots of the prediction models in validation cohort. In different prediction probability sections, the apparent and bias‐corrected curves showed good agreement and had a certain deviation from the ideal line. (A) Calibration plot of the logistic prediction model. (B) Calibration plot of the LASSO prediction model

The prediction models are shown in the form of nomograms in Figure 2. In the model based on the 11 risk predictors from the multimodal logistic analyses, the nomogram standard scoring line of 0–100 points was the baseline which gave a corresponding score range for each predictor, where age, glucose, potassium, international normalized ratio, partial thromboplastin rate, and respiratory rate ranged from 0 to 100, 0 to 600, 0.5 to 7, 0 to 16, 10 to 150, and 15 to 55, respectively. By calculating and summing up the weighted scores for each predictor included in the model, a total score of 380–560 was obtained, which corresponded to an approximately 2.0%–90% overall probability of NCDs (Figure 1A). In the other model based on the combination of the selected non‐zero variables from the LASSO analysis, the total score was 960–1,080, which corresponded to an overall probability of 0.2%–99.5% (Figure 1B).

### 3.4 Model performance

The performances of the prediction models were internally assessed using the validation cohort. First, the calibration plots of the prediction models showed that the apparent and bias‐corrected curves showed slight deviations from the ideal line (Figure 2), which indicated high consistency between the observations and predictions for the predictive value of the nomograms for NCDs. LASSO regression analysis prediction model calibration curve (Figure 2B) is better than logistic regression analysis prediction model (Figure 2A).

**FIGURE 3:**
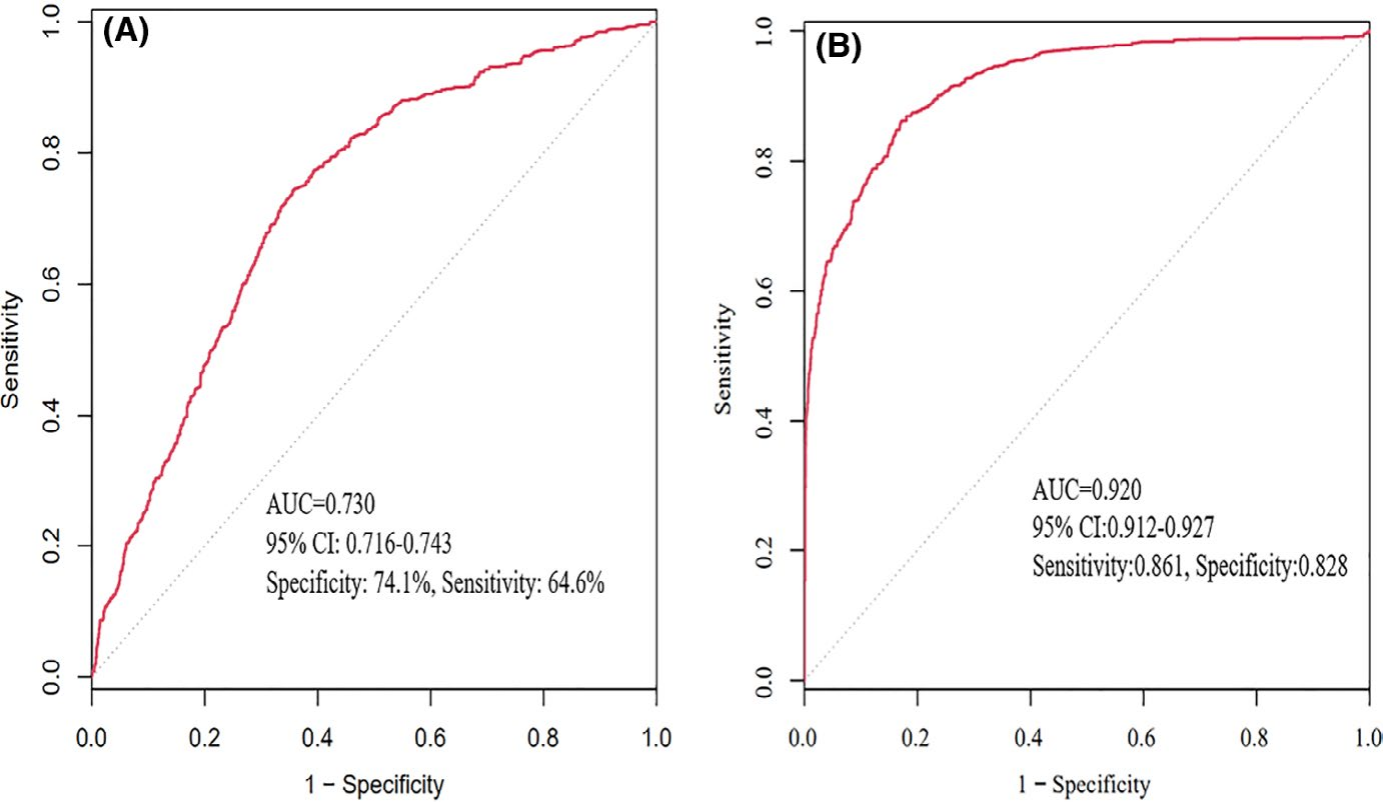
The ROC curve for the prediction models. (A) ROC curve for the logistic prediction model. Sensitivity and specificity were 74.1% and 64.6%, respectively, and AUC score was 0.730 (95% CI 0.716–0.743) in the ROC. (B) ROC curve for the LASSO prediction model. Sensitivity and specificity were 86.1% and 82.8%, respectively, and AUC score was 0.920 (95% CI 0.912–0.927) in the ROC

Second, the AUC for the logistic prediction model was 0.730 (95% CI 0.716–0.743); the sensitivity and specificity based on the ROC were 74.1% and 64.6%, respectively (Figure 3A). The AUC of the LASSO prediction model was 0.920 (95% CI 0.912–0.927); it exhibited high sensitivity and specificity at 86.1% and 82.8%, respectively (Figure 3B), which indicated that it had a better discriminative ability for the prediction of NCDs.

**FIGURE 4:**
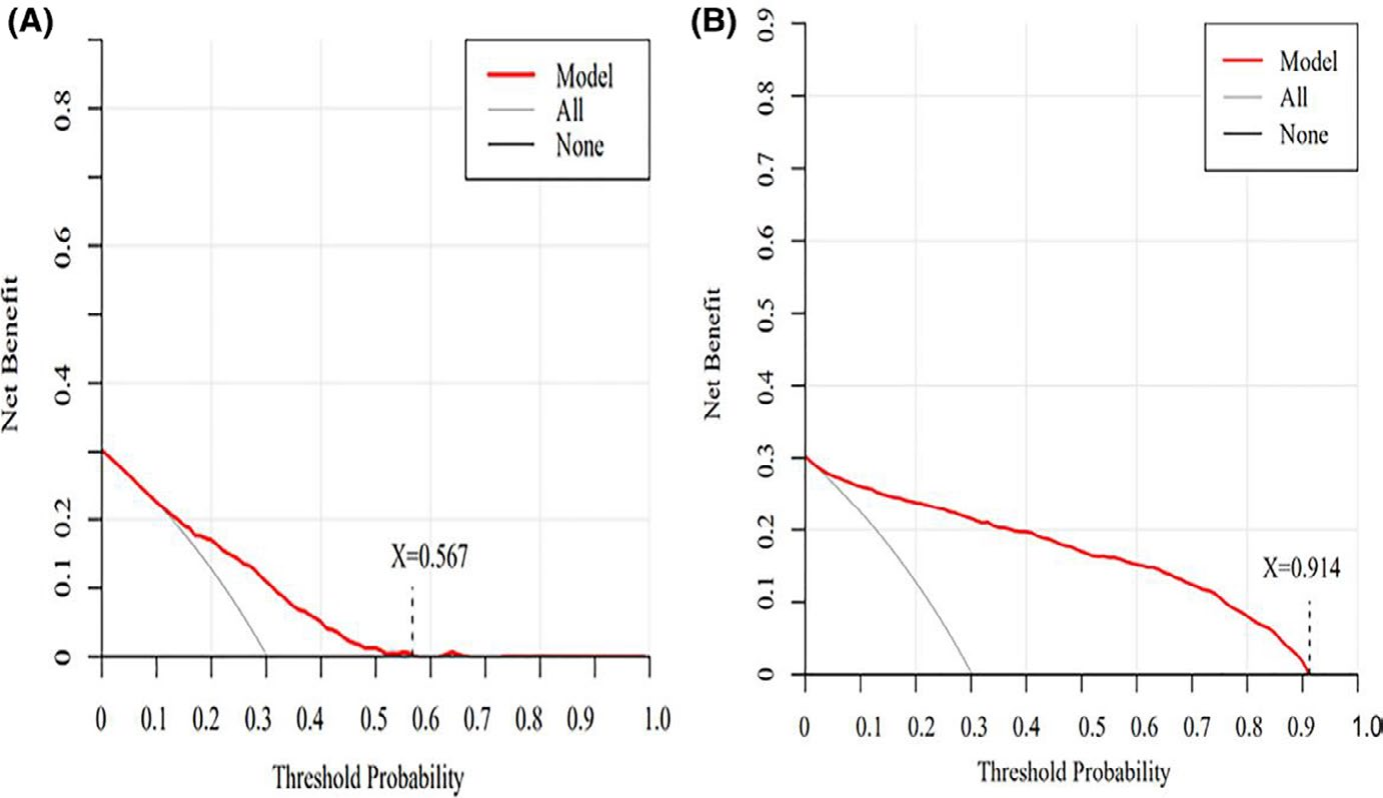
The DCA curve for the prediction models. (A) DCA curve for the logistic prediction model. A threshold probability value was 0.567. (B) DCA curve for the LASSO prediction model. A threshold probability value was 0.914

Finally, DCA was used to evaluate the clinical utility of the prediction models. The DCA curve represented a continuum of threshold probability (x‐axis) for major NCDs risk and net benefit (y‐axis) of model applicability for potential patients at risk relative to the assumption that no patients would have NCDs. The DCA showed that the threshold probability of net benefit was 0.567 for the logistic prediction model (Figure 4A) and 0.914 for the LASSO prediction model (Figure 4B). For the range of NCD risk, the net benefit of the LASSO prediction model was larger than that of the logistic prediction model. In addition, the DCA of midazolam use in predicting NCDs risk showed better performance at a threshold probability of 0.475 (Appendix S7).

## 4 DISCUSSION

Based on specifically collected clinical data from MIMIC‐IV version 1.0, we developed and validated the prediction models for NCDs in adult patients with sleep disturbances during critical care. The logistic prediction model was based on the following 11 predictors: age, midazolam use, gender, respiratory rate, partial thromboplastin time, international normalized ratio, potassium, glucose, diabetes, cardiovascular diseases, and morphine use. It comprised of the most potentially valuable predictors of the diagnostic information, including clinical and biological variables. The LASSO prediction model consisted of 12 independent risk variables, among which platelet counts and GCS were included, unlike in the logistic prediction model; the remaining 10 variables were the same as that in the logistic prediction model. Accordingly, two nomograms were drawn to predict the risk of NCDs in the primary cohort. Furthermore, we internally validated the performances of the two nomograms for risk prediction of NCDs in the validation cohort. Although the prediction models showed similar trends for calibration, the LASSO prediction model had the better discriminative ability as compared to the logistic prediction model, which suggested that it could predict the risk of NCDs with higher accuracy. Importantly, the prediction models also had great clinical utility for the prediction of NCDs risk in ICU patients with sleep disturbance as shown by the findings of the DCA. The LASSO prediction model had better performance as compared to the logistic prediction model in the net benefit of nomogram‐assisted decision‐making at the set threshold probability range of 0.567–0.914.

Nomograms are commonly used risk assessment tools for the prognosis of cancers and cerebrovascular diseases.^27^, ^28^ Interestingly, with the ability to predict the individual probability of a clinical event by integrating diverse potential variables, nomograms have now been extended to clinically and biologically integrated diagnostic models, which are an important component of modern medical decision‐making.^25^ In this study, we used clinical and biological variables to construct two visualization nomograms for the prediction of NCDs risk in a patient admitted to the ICU having sleep disturbance. Although the two nomograms followed the standard processes of proofreading, recognition, and clinical application, along with good prediction performances, the prospective effects of nomogram‐assisted decisions for patient satisfaction and outcomes remain unclear. Given that, currently, there is a paucity in nomogram availability for ICU clinicians, this study provided two alternative assessment tools for high‐risk NCDs in ICU patients suffering from a high magnitude of sleep disturbance. Notably, despite the internal validation which showed good discrimination and calibration of the nomograms, they were not assessed externally and prospectively; thus, we need to evaluate whether their use could improve patient outcomes over clinician judgment in future by conducting a prospective assessment. Software development according to the established perfect model, combining medical treatment and engineering for clinical transformation, fill in the gaps in study.

The strongest predictors of major NCDs in this cohort of ICU patients with sleep disturbance were medications, in particular, the use of midazolam and morphine. Critically ill patients, especially those with sleep disturbance, often need sedatives and analgesics, which are inseparable from the common benzodiazepines and morphine.^12^ In the population, benzodiazepines, including midazolam, have been identified as being independently associated with the development of delirium and dementia.^29^, ^30^ However, the impact of midazolam, the most powerful predictor, is changeable owing to the use of low‐risk sedatives, such as dexmedetomidine and propofol as substitutes.^12^, ^31^, ^32^ Dexmedetomidine was not significantly associated with NCDs in this cohort and even appeared to provide potential protection against delirium among adults on mechanical ventilators.^12^, ^33^ Besides, the targeted sedation level of dexmedetomidine was not different from that of midazolam in ICU patients; dexmedetomidine‐treated patients experienced less delirium.^32^ Interestingly, in previous findings on sleep disturbance, dexmedetomidine is known to stimulate natural sleep and improve sleep quality,^34^ which are potentially advantageous for preventing NCDs in ICU patients with sleep disturbance, owing to the two‐way relationship between sleep disturbance and major NCDs.^4^, ^14^, ^18^ Notably, propofol, another commonly used alternative sedative, showed protective characteristics and reduced the risk of NCDs in this study. The results supported the superiority of propofol against dexmedetomidine for the prevention of NCDs. However, this may only be suitable for adult patients with sleep disturbance in the ICU. Based on current clinical evidence,^35^ in older ICU patients at risk of delirium, compared with propofol, dexmedetomidine is associated with a lower incidence of delirium. However, our findings showed that age had no significant association with the risk of NCDs, which was not consistent with the PRE‐DELIRIC model.^16^ Although age has important associations with the development and progression of major NCDs,^16^, ^36^, ^37^ it is irreversible and lacks targeted preventive measures. However, alternative sedatives are prescribed according to age to counteract age‐related neurocognitive declines. Additionally, in this study, the use of morphine showed a significant association with major NCDs, and the association shows similar trends as morphine and delirium in the PRE‐DELIRIC model.^16^ However, the causal association between morphine and major NCDs, including delirium remains controversial,^38^ and even, the 2018 Pain, Agitation/sedation, Delirium, Immobility, and Sleep disruption (PADIS) guidelines do not propose any high‐quality recommendations in this regard for the ICU patients.^12^ Fortunately, morphine use is intervenable and replaceable by multimodal analgesics, indicating that even though the use of morphine is a powerful risk predictor for major NCDs,^39^ non‐morphine analgesics, including nonsteroidal anti‐inflammatory drugs, may be conducive for the prevention of NCDs causing sleep disturbance in the ICU patients. Nevertheless, evidence suggests that nonsteroidal anti‐inflammatory drugs are also associated with increased risk of delirium in the elderly,^40^, ^41^ but not in adults. Therefore, when using the prediction model to identify morphine use, age also should be fully considered during the selection of low‐risk alternatives. Notably, in cases of severe pain, opioid therapy can be taken, as one should not ignore that pain itself has a prominent association with delirium.^38^

The prediction models incorporated the laboratory parameters as potential predictive biomarkers for the processes underlying NCDs in sleep disturbance among ICU patients. Despite the lack of specific neurological biomarkers, increased concentrations of potassium showed a significant independent association with major NCDs in this study. Increased serum potassium is bidirectionally correlated with reduced pH value and metabolic acidosis,^42^ both of which are independently associated with delirium in ICU patients.^16^ Additionally, biomarkers reflecting coagulation dysfunction, such as increased partial thromboplastin rate and international normalized ratio, could directly reflecting the decrease in coagulation functions and were independent predictors of major NCDs events. A previous study has evaluated the association between coagulation and major NCDs in the ICU,^43^, ^44^ but the predictive value of biomarkers reflecting different coagulation stages remains inconsistent.^45^ However, this correlation was largely dependent on the initiation of inflammation, underlying mechanisms, including inflammation, damaged vascular endothelium, abnormal activation of coagulation pathways, inhibition of anticoagulant and fibrinolysis functions, and micro‐thrombosis finally leading to cognitive decline, even dementia.^43^, ^44^ Instead, our findings showed that inhibited coagulation independently increased the risk of NCDs without any significant inflammatory differences. However, biomarkers in these studies were limited to specific cytokines of the anticoagulant and fibrinolytic systems, such as protein C and plasminogen activating inhibitor‐1, which represented increased coagulation in delirium and dementia.^43^, ^45^ We did not test these specific cytokines, but these were commonly incorporated clinical coagulation parameters, which may be conducive as universally and easily available predictive biomarkers. Similarly, increased blood glucose manifested as diabetes was an important predictor of the risk of NCDs^46^; it has previously been considered a risk factor for delirium.^47^ As shown by the results, diabetes was also an independent risk factor. Therefore, for ICU patients with sleep disturbance who are at risk of NCDs, comorbid diabetes, in particular, lowering blood glucose within a safe range could reduce the probability of NCDs.

Gender, respiratory rate, and other vital signs, in several PRE‐DELIRIC models,^16^, ^48^ do not contribute to the risk of delirium and are not commonly used. As an important system score for evaluating the status of critically ill patients, APACHE II, shows an important association with ICU delirium,^16^ however, this was inconsistent with our findings. Nonetheless, the APACHE II score may have potential value for predicting delirium in ICU patients with sleep disturbance, as in our study, patients with NCDs had higher APACHE II scores than those without NCDs in the primary cohort; in addition to delirium, MCI and dementia were added to the primary outcomes, which could have reduced the overall specificity and sensitivity of this score. Most importantly, based on the integration of several clinical variables, the model could identify modifiable risks to facilitate the availability of targeted prevention of NCDs in high‐risk patients.

However, this study has some limitations. First, although MIMIC‐IV (version 1.0) included comprehensive clinical data from critically ill patients with sleep disturbance and is recently updated, this study had inherent limitations of a retrospective cohort, including selection bias, low levels of evidence, and data loss. Second, the clinical and biological variables were measured at one point on the first day of admission to ICU; however, in the development process of sleep disturbance to the NCDs, these variables are dynamic and change over time; thus, we may have missed the diagnostic importance of unrecognized variables. Third, due to a lack of prospective validation, the external applicability of the model may be limited. It needs to be resolved through a prospective external queue in future by us. Finally, the single‐outcome events were not independently analyzed, which could have reduced the specificity of the model for single NCD events. Despite the above limitations, early prediction of the NCDs may provide response time for clinicians for the implementation of preventive measures.

## 5 CONCLUSION

In conclusion, the models could predict NCDs for adult ICU patients with sleep disturbance. Most importantly, the LASSO prediction model had prominent advantages over the logistic prediction model in terms of their predictive performances and, therefore, could provide clinical decision support for neurocognitive protection in ICU patients with sleep disturbance.

## CONFLICTS OF INTEREST

The authors declare no competing interests.

## AUTHOR CONTRIBUTIONS

Yun Li and Lina Zhao analyzed the data and wrote the manuscript. Ye Wang extracted the data from MIMIC‐IV. Xizhe Zhang, Qi Zhou, and Jiannan Song performed the literature search and interpreted the results. Chenyi Yang revised the manuscript. Yun Li and Haiyun Wang conceived the study design. All authors have approved the final manuscript.

## Supporting information

App S1

App S2

App S3

App S4

App S5

App S6

App S7

Data S1

## Data Availability

The data set used in this study is in "Physionet" (https://doi.org/10.13026/s6n6‐xd98).
