# Supplementary material for: Development and validation of prediction models for neurocognitive disorders in adult patients admitted to the ICU with sleep disturbance
Source: CNS Neurosci Ther. 2021 Dec 23;28(4):554–65. doi: 10.1111/cns.13772 (PMC8928914; doi:10.1111/cns.13772)
Supplement: Supplementary file 1 — App S1 [file CNS-28-554-s006.docx]

Appendix S1

|  | | |  |  |  |  |  |  |  |  |  |
| --- | --- | --- | --- | --- | --- | --- | --- | --- | --- | --- | --- |
| Disease | ICD9-Code and ICD10-Code | Description |  |  |  |  |  |  |  |  |  |
| Sleep disorders |  |  |  |  |  |  |  |  |  |  |  |
|  | 4672 | Fatal familial insomnia |  |  |  |  |  |  |  |  |  |
|  | 32700 | Organic insomnia, unspecified |  |  |  |  |  |  |  |  |  |
|  | 32701 | Insomnia due to medical condition classified elsewhere |  |  |  |  |  |  |  |  |  |
|  | 32702 | Insomnia due to mental disorder |  |  |  |  |  |  |  |  |  |
|  | 32709 | Other organic insomnia |  |  |  |  |  |  |  |  |  |
|  | 78051 | Insomnia with sleep apnea, unspecified |  |  |  |  |  |  |  |  |  |
|  | 78052 | Insomnia, unspecified |  |  |  |  |  |  |  |  |  |
|  | A8183 | Fatal familial insomnia |  |  |  |  |  |  |  |  |  |
|  | F5101 | Primary insomnia |  |  |  |  |  |  |  |  |  |
|  | F5102 | Adjustment insomnia |  |  |  |  |  |  |  |  |  |
|  | F5103 | Paradoxical insomnia |  |  |  |  |  |  |  |  |  |
|  | F5104 | Psychophysiologic insomnia |  |  |  |  |  |  |  |  |  |
|  | F5105 | Insomnia due to other mental disorder |  |  |  |  |  |  |  |  |  |
|  | F5109 | Other insomnia not due to a substance or known physiological condition |  |  |  |  |  |  |  |  |  |
|  | G4700 | Insomnia, unspecified |  |  |  |  |  |  |  |  |  |
|  | G4701 | Insomnia due to medical condition |  |  |  |  |  |  |  |  |  |
|  | G4709 | Other insomnia |  |  |  |  |  |  |  |  |  |
|  | V695 | Behavioral insomnia of childhood |  |  |  |  |  |  |  |  |  |
|  | Z73810 | Behavioral insomnia of childhood, sleep-onset association type |  |  |  |  |  |  |  |  |  |
|  | Z73811 | Behavioral insomnia of childhood, limit setting type |  |  |  |  |  |  |  |  |  |
|  | Z73812 | Behavioral insomnia of childhood, combined type |  |  |  |  |  |  |  |  |  |
|  | Z73819 | Behavioral insomnia of childhood, unspecified type |  |  |  |  |  |  |  |  |  |
|  | 29182 | Alcohol induced sleep disorders |  |  |  |  |  |  |  |  |  |
|  | 29285 | Drug induced sleep disorders |  |  |  |  |  |  |  |  |  |
|  | 30740 | Nonorganic sleep disorder, unspecified |  |  |  |  |  |  |  |  |  |
|  | 30741 | Transient disorder of initiating or maintaining sleep |  |  |  |  |  |  |  |  |  |
|  | 30742 | Persistent disorder of initiating or maintaining sleep |  |  |  |  |  |  |  |  |  |
|  | 30743 | Transient disorder of initiating or maintaining wakefulness |  |  |  |  |  |  |  |  |  |
|  | 30744 | Persistent disorder of initiating or maintaining wakefulness |  |  |  |  |  |  |  |  |  |
|  | 30745 | Circadian rhythm sleep disorder of nonorganic origin |  |  |  |  |  |  |  |  |  |
|  | 30746 | Sleep arousal disorder |  |  |  |  |  |  |  |  |  |
|  | 30747 | Other dysfunctions of sleep stages or arousal from sleep |  |  |  |  |  |  |  |  |  |
|  | 30748 | Repetitive intrusions of sleep |  |  |  |  |  |  |  |  |  |
|  | 30749 | Other specific disorders of sleep of nonorganic origin |  |  |  |  |  |  |  |  |  |
|  | 32710 | Organic hypersomnia, unspecified |  |  |  |  |  |  |  |  |  |
|  | 32711 | Idiopathic hypersomnia with long sleep time |  |  |  |  |  |  |  |  |  |
|  | 32712 | Idiopathic hypersomnia without long sleep time |  |  |  |  |  |  |  |  |  |
|  | 32713 | Recurrent hypersomnia |  |  |  |  |  |  |  |  |  |
|  | 32714 | Hypersomnia due to medical condition classified elsewhere |  |  |  |  |  |  |  |  |  |
|  | 32715 | Hypersomnia due to mental disorder |  |  |  |  |  |  |  |  |  |
|  | 32719 | Other organic hypersomnia |  |  |  |  |  |  |  |  |  |
|  | 32720 | Organic sleep apnea, unspecified |  |  |  |  |  |  |  |  |  |
|  | 32721 | Primary central sleep apnea |  |  |  |  |  |  |  |  |  |
|  | 32722 | High altitude periodic breathing |  |  |  |  |  |  |  |  |  |
|  | 32723 | Obstructive sleep apnea (adult)(pediatric) |  |  |  |  |  |  |  |  |  |
|  | 32724 | Idiopathic sleep related non-obstructive alveolar hypoventilation |  |  |  |  |  |  |  |  |  |
|  | 32725 | Congenital central alveolar hypoventilation syndrome |  |  |  |  |  |  |  |  |  |
|  | 32726 | Sleep related hypoventilation/hypoxemia in conditions classifiable elsewhere |  |  |  |  |  |  |  |  |  |
|  | 32727 | Central sleep apnea in conditions classified elsewhere |  |  |  |  |  |  |  |  |  |
|  | 32729 | Other organic sleep apnea |  |  |  |  |  |  |  |  |  |
|  | 32730 | Circadian rhythm sleep disorder, unspecified |  |  |  |  |  |  |  |  |  |
|  | 32731 | Circadian rhythm sleep disorder, delayed sleep phase type |  |  |  |  |  |  |  |  |  |
|  | 32732 | Circadian rhythm sleep disorder, advanced sleep phase type |  |  |  |  |  |  |  |  |  |
|  | 32733 | Circadian rhythm sleep disorder, irregular sleep-wake type |  |  |  |  |  |  |  |  |  |
|  | 32734 | Circadian rhythm sleep disorder, free-running type |  |  |  |  |  |  |  |  |  |
|  | 32735 | Circadian rhythm sleep disorder, jet lag type |  |  |  |  |  |  |  |  |  |
|  | 32736 | Circadian rhythm sleep disorder, shift work type |  |  |  |  |  |  |  |  |  |
|  | 32737 | Circadian rhythm sleep disorder in conditions classified elsewhere |  |  |  |  |  |  |  |  |  |
|  | 32739 | Other circadian rhythm sleep disorder |  |  |  |  |  |  |  |  |  |
|  | 32740 | Organic parasomnia, unspecified |  |  |  |  |  |  |  |  |  |
|  | 32741 | Confusional arousals |  |  |  |  |  |  |  |  |  |
|  | 32742 | REM sleep behavior disorder |  |  |  |  |  |  |  |  |  |
|  | 32743 | Recurrent isolated sleep paralysis |  |  |  |  |  |  |  |  |  |
|  | 32744 | Parasomnia in conditions classified elsewhere |  |  |  |  |  |  |  |  |  |
|  | 32749 | Other organic parasomnia |  |  |  |  |  |  |  |  |  |
|  | 32751 | Periodic limb movement disorder |  |  |  |  |  |  |  |  |  |
|  | 32752 | Sleep related leg cramps |  |  |  |  |  |  |  |  |  |
|  | 32753 | Sleep related bruxism |  |  |  |  |  |  |  |  |  |
|  | 32759 | Other organic sleep related movement disorders |  |  |  |  |  |  |  |  |  |
|  | 3278 | Other organic sleep disorders |  |  |  |  |  |  |  |  |  |
|  | 34700 | Narcolepsy, without cataplexy |  |  |  |  |  |  |  |  |  |
|  | 34701 | Narcolepsy, with cataplexy |  |  |  |  |  |  |  |  |  |
|  | 34710 | Narcolepsy in conditions classified elsewhere, without cataplexy |  |  |  |  |  |  |  |  |  |
|  | 34711 | Narcolepsy in conditions classified elsewhere, with cataplexy |  |  |  |  |  |  |  |  |  |
|  | 78050 | Sleep disturbance, unspecified |  |  |  |  |  |  |  |  |  |
|  | 78053 | Hypersomnia with sleep apnea, unspecified |  |  |  |  |  |  |  |  |  |
|  | 78054 | Hypersomnia, unspecified |  |  |  |  |  |  |  |  |  |
|  | 78055 | Disruption of 24 hour sleep wake cycle, unspecified |  |  |  |  |  |  |  |  |  |
|  | 78056 | Dysfunctions associated with sleep stages or arousal from sleep |  |  |  |  |  |  |  |  |  |
|  | 78057 | Unspecified sleep apnea |  |  |  |  |  |  |  |  |  |
|  | 78058 | Sleep related movement disorder, unspecified |  |  |  |  |  |  |  |  |  |
|  | 78059 | Other sleep disturbances |  |  |  |  |  |  |  |  |  |
|  | F10182 | Alcohol abuse with alcohol-induced sleep disorder |  |  |  |  |  |  |  |  |  |
|  | F10282 | Alcohol dependence with alcohol-induced sleep disorder |  |  |  |  |  |  |  |  |  |
|  | F10982 | Alcohol use, unspecified with alcohol-induced sleep disorder |  |  |  |  |  |  |  |  |  |
|  | F11182 | Opioid abuse with opioid-induced sleep disorder |  |  |  |  |  |  |  |  |  |
|  | F11282 | Opioid dependence with opioid-induced sleep disorder |  |  |  |  |  |  |  |  |  |
|  | F11982 | Opioid use, unspecified with opioid-induced sleep disorder |  |  |  |  |  |  |  |  |  |
|  | F13182 | Sedative, hypnotic or anxiolytic abuse with sedative, hypnotic or anxiolytic-induced sleep disorder |  |  |  |  |  |  |  |  |  |
|  | F13282 | Sedative, hypnotic or anxiolytic dependence with sedative, hypnotic or anxiolytic-induced sleep disorder |  |  |  |  |  |  |  |  |  |
|  | F13982 | Sedative, hypnotic or anxiolytic use, unspecified with sedative, hypnotic or anxiolytic-induced sleep disorder |  |  |  |  |  |  |  |  |  |
|  | F14182 | Cocaine abuse with cocaine-induced sleep disorder |  |  |  |  |  |  |  |  |  |
|  | F14282 | Cocaine dependence with cocaine-induced sleep disorder |  |  |  |  |  |  |  |  |  |
|  | F14982 | Cocaine use, unspecified with cocaine-induced sleep disorder |  |  |  |  |  |  |  |  |  |
|  | F15182 | Other stimulant abuse with stimulant-induced sleep disorder |  |  |  |  |  |  |  |  |  |
|  | F15282 | Other stimulant dependence with stimulant-induced sleep disorder |  |  |  |  |  |  |  |  |  |
|  | F15982 | Other stimulant use, unspecified with stimulant-induced sleep disorder |  |  |  |  |  |  |  |  |  |
|  | F19182 | Other psychoactive substance abuse with psychoactive substance-induced sleep disorder |  |  |  |  |  |  |  |  |  |
|  | F19282 | Other psychoactive substance dependence with psychoactive substance-induced sleep disorder |  |  |  |  |  |  |  |  |  |
|  | F19982 | Other psychoactive substance use, unspecified with psychoactive substance-induced sleep disorder |  |  |  |  |  |  |  |  |  |
|  | F5111 | Primary hypersomnia |  |  |  |  |  |  |  |  |  |
|  | F5112 | Insufficient sleep syndrome |  |  |  |  |  |  |  |  |  |
|  | F5113 | Hypersomnia due to other mental disorder |  |  |  |  |  |  |  |  |  |
|  | F5119 | Other hypersomnia not due to a substance or known physiological condition |  |  |  |  |  |  |  |  |  |
|  | F513 | Sleepwalking [somnambulism] |  |  |  |  |  |  |  |  |  |
|  | F514 | Sleep terrors [night terrors] |  |  |  |  |  |  |  |  |  |
|  | F515 | Nightmare disorder |  |  |  |  |  |  |  |  |  |
|  | F518 | Other sleep disorders not due to a substance or known physiological condition |  |  |  |  |  |  |  |  |  |
|  | F519 | Sleep disorder not due to a substance or known physiological condition, unspecified |  |  |  |  |  |  |  |  |  |
|  | F5222 | Female sexual arousal disorder |  |  |  |  |  |  |  |  |  |
|  | G4710 | Hypersomnia, unspecified |  |  |  |  |  |  |  |  |  |
|  | G4711 | Idiopathic hypersomnia with long sleep time |  |  |  |  |  |  |  |  |  |
|  | G4712 | Idiopathic hypersomnia without long sleep time |  |  |  |  |  |  |  |  |  |
|  | G4713 | Recurrent hypersomnia |  |  |  |  |  |  |  |  |  |
|  | G4714 | Hypersomnia due to medical condition |  |  |  |  |  |  |  |  |  |
|  | G4719 | Other hypersomnia |  |  |  |  |  |  |  |  |  |
|  | G4720 | Circadian rhythm sleep disorder, unspecified type |  |  |  |  |  |  |  |  |  |
|  | G4721 | Circadian rhythm sleep disorder, delayed sleep phase type |  |  |  |  |  |  |  |  |  |
|  | G4722 | Circadian rhythm sleep disorder, advanced sleep phase type |  |  |  |  |  |  |  |  |  |
|  | G4723 | Circadian rhythm sleep disorder, irregular sleep wake type |  |  |  |  |  |  |  |  |  |
|  | G4724 | Circadian rhythm sleep disorder, free running type |  |  |  |  |  |  |  |  |  |
|  | G4725 | Circadian rhythm sleep disorder, jet lag type |  |  |  |  |  |  |  |  |  |
|  | G4726 | Circadian rhythm sleep disorder, shift work type |  |  |  |  |  |  |  |  |  |
|  | G4727 | Circadian rhythm sleep disorder in conditions classified elsewhere |  |  |  |  |  |  |  |  |  |
|  | G4729 | Other circadian rhythm sleep disorder |  |  |  |  |  |  |  |  |  |
|  | G4730 | Sleep apnea, unspecified |  |  |  |  |  |  |  |  |  |
|  | G4731 | Primary central sleep apnea |  |  |  |  |  |  |  |  |  |
|  | G4732 | High altitude periodic breathing |  |  |  |  |  |  |  |  |  |
|  | G4733 | Obstructive sleep apnea (adult) (pediatric) |  |  |  |  |  |  |  |  |  |
|  | G4734 | Idiopathic sleep related nonobstructive alveolar hypoventilation |  |  |  |  |  |  |  |  |  |
|  | G4735 | Congenital central alveolar hypoventilation syndrome |  |  |  |  |  |  |  |  |  |
|  | G4736 | Sleep related hypoventilation in conditions classified elsewhere |  |  |  |  |  |  |  |  |  |
|  | G4737 | Central sleep apnea in conditions classified elsewhere |  |  |  |  |  |  |  |  |  |
|  | G4739 | Other sleep apnea |  |  |  |  |  |  |  |  |  |
|  | G47411 | Narcolepsy with cataplexy |  |  |  |  |  |  |  |  |  |
|  | G47419 | Narcolepsy without cataplexy |  |  |  |  |  |  |  |  |  |
|  | G47421 | Narcolepsy in conditions classified elsewhere with cataplexy |  |  |  |  |  |  |  |  |  |
|  | G47429 | Narcolepsy in conditions classified elsewhere without cataplexy |  |  |  |  |  |  |  |  |  |
|  | G4750 | Parasomnia, unspecified |  |  |  |  |  |  |  |  |  |
|  | G4751 | Confusional arousals |  |  |  |  |  |  |  |  |  |
|  | G4752 | REM sleep behavior disorder |  |  |  |  |  |  |  |  |  |
|  | G4753 | Recurrent isolated sleep paralysis |  |  |  |  |  |  |  |  |  |
|  | G4754 | Parasomnia in conditions classified elsewhere |  |  |  |  |  |  |  |  |  |
|  | G4759 | Other parasomnia |  |  |  |  |  |  |  |  |  |
|  | 33394 | Restless legs syndrome (RLS) |  |  |  |  |  |  |  |  |  |
|  | G4761 | Periodic limb movement disorder |  |  |  |  |  |  |  |  |  |
|  | G4762 | Sleep related leg cramps |  |  |  |  |  |  |  |  |  |
|  | G4763 | Sleep related bruxism |  |  |  |  |  |  |  |  |  |
|  | G4769 | Other sleep related movement disorders |  |  |  |  |  |  |  |  |  |
|  | G478 | Other sleep disorders |  |  |  |  |  |  |  |  |  |
|  | G479 | Sleep disorder, unspecified |  |  |  |  |  |  |  |  |  |
|  | P283 | Primary sleep apnea of newborn |  |  |  |  |  |  |  |  |  |
|  | V694 | Lack of adequate sleep |  |  |  |  |  |  |  |  |  |
|  | Y9384 | Activity, sleeping |  |  |  |  |  |  |  |  |  |
|  | Z72820 | Sleep deprivation |  |  |  |  |  |  |  |  |  |
|  | Z72821 | Inadequate sleep hygiene |  |  |  |  |  |  |  |  |  |
|  |  |  |  |  |  |  |  |  |  |  |  |
|  |  |  |  |  |  |  |  |  |  |  |  |
|  |  |  |  |  |  |  |  |  |  |  |  |
