# Supplementary material for: Development and validation of prediction models for neurocognitive disorders in adult patients admitted to the ICU with sleep disturbance
Source: CNS Neurosci Ther. 2021 Dec 23;28(4):554–65. doi: 10.1111/cns.13772 (PMC8928914; doi:10.1111/cns.13772)
Supplement: Supplementary file 2 — App S2 [file CNS-28-554-s002.docx]

Appendix S2

| Disease | ICD-Code | Description |
| --- | --- | --- |
| Neurocognitive disorders |  | LONG_TITLE |
|  | 29011 | Presenile dementia with delirium |
|  | 2903 | Senile dementia with delirium |
|  | 29041 | Vascular dementia, with delirium |
|  | 2910 | Alcohol withdrawal delirium |
|  | 29281 | Drug-induced delirium |
|  | 2930 | Delirium due to conditions classified elsewhere |
|  | 2931 | Subacute delirium |
|  | 30012 | Dissociative amnesia |
|  | 4377 | Transient global amnesia |
|  | 29283 | Drug-induced persisting amnestic disorder |
|  | 2911 | Alcohol-induced persisting amnestic disorder |
|  | 2940 | Amnestic disorder in conditions classified elsewhere |
|  | 2900 | Senile dementia, uncomplicated |
|  | 2900 | Senile dementia, uncomplicated |
|  | 29010 | Presenile dementia, uncomplicated |
|  | 29012 | Presenile dementia with delusional features |
|  | 29013 | Presenile dementia with depressive features |
|  | 29020 | Senile dementia with delusional features |
|  | 29021 | Senile dementia with depressive features |
|  | 29040 | Vascular dementia, uncomplicated |
|  | 29042 | Vascular dementia, with delusions |
|  | 29043 | Vascular dementia, with depressed mood |
|  | 2912 | Alcohol-induced persisting dementia |
|  | 29282 | Drug-induced persisting dementia |
|  | 29410 | Dementia in conditions classified elsewhere without behavioral disturbance |
|  | 29411 | Dementia in conditions classified elsewhere with behavioral disturbance |
|  | 29420 | Dementia, unspecified, without behavioral disturbance |
|  | 29421 | Dementia, unspecified, with behavioral disturbance |
|  | 33119 | Other frontotemporal dementia |
|  | 33182 | Dementia with lewy bodies |
|  | 3310 | Alzheimer's disease |
|  | 33183 | Mild cognitive impairment, so stated |
|  | 4380 | Late effects of cerebrovascular disease, cognitive deficits |
|  | 31400 | Attention deficit disorder without mention of hyperactivity |
|  | 31401 | Attention deficit disorder with hyperactivity |
|  | 79951 | Attention or concentration deficit |
|  | 79952 | Cognitive communication deficit |
|  | 79953 | Visuospatial deficit |
|  | 79959 | Other signs and symptoms involving cognition |
|  | 78093 | Memory loss |
