# Supplementary material for: Development and validation of prediction models for neurocognitive disorders in adult patients admitted to the ICU with sleep disturbance
Source: CNS Neurosci Ther. 2021 Dec 23;28(4):554–65. doi: 10.1111/cns.13772 (PMC8928914; doi:10.1111/cns.13772)
Supplement: Supplementary file 3 — App S3 [file CNS-28-554-s004.docx]

Appendix S3

|  | |  |  |  |  |  |  |  |  |  |  |  |  |  |  |  |  |  |  |  |  |  |  |  |  |  |  |  |  |
| --- | --- | --- | --- | --- | --- | --- | --- | --- | --- | --- | --- | --- | --- | --- | --- | --- | --- | --- | --- | --- | --- | --- | --- | --- | --- | --- | --- | --- | --- |
| **Supplementary table 3: specific neuropsychiatric diseases** | | | | | | | | | | | | | | | | | | | | | | | | | | | | | |
| ICD9-code and ICD10-code | | | | | Description | | | | | | | | | | | | | | | | | | | | | | | | |
| 80016 | | | | | Closed fracture of vault of skull with cerebral laceration and contusion, with loss of consciousness of unspecified duration | | | | | | | | | | | | | | | | | | | | | | | | |
| 80019 | | | | | Closed fracture of vault of skull with cerebral laceration and contusion, with concussion, unspecified | | | | | | | | | | | | | | | | | | | | | | | | |
| 80020 | | | | | Closed fracture of vault of skull with subarachnoid, subdural, and extradural hemorrhage, unspecified state of consciousness | | | | | | | | | | | | | | | | | | | | | | | | |
| 80021 | | | | | Closed fracture of vault of skull with subarachnoid, subdural, and extradural hemorrhage, with no loss of consciousness | | | | | | | | | | | | | | | | | | | | | | | | |
| 80022 | | | | | Closed fracture of vault of skull with subarachnoid, subdural, and extradural hemorrhage, with brief [less than one hour] loss of consciousness | | | | | | | | | | | | | | | | | | | | | | | | |
| 80023 | | | | | Closed fracture of vault of skull with subarachnoid, subdural, and extradural hemorrhage, with moderate [1-24 hours] loss of consciousness | | | | | | | | | | | | | | | | | | | | | | | | |
| 80024 | | | | | Closed fracture of vault of skull with subarachnoid, subdural, and extradural hemorrhage, with prolonged [more than 24 hours] loss of consciousness and return to pre-existing conscioucs level | | | | | | | | | | | | | | | | | | | | | | | | |
| 80025 | | | | | Closed fracture of vault of skull with subarachnoid, subdural, and extradural hemorrhage, with prolonged [more than 24 hours] loss of consciousness, without return to pre-existing conscious level | | | | | | | | | | | | | | | | | | | | | | | | |
| 80026 | | | | | Closed fracture of vault of skull with subarachnoid, subdural, and extradural hemorrhage, with loss of consciousness of unspecified duration | | | | | | | | | | | | | | | | | | | | | | | | |
| 80029 | | | | | Closed fracture of vault of skull with subarachnoid, subdural, and extradural hemorrhage, with concussion, unspecified | | | | | | | | | | | | | | | | | | | | | | | | |
| 80030 | | | | | Closed fracture of vault of skull with other and unspecified intracranial hemorrhage, unspecified state of consciousness | | | | | | | | | | | | | | | | | | | | | | | | |
| 80031 | | | | | Closed fracture of vault of skull with other and unspecified intracranial hemorrhage, with no loss of consciousness | | | | | | | | | | | | | | | | | | | | | | | | |
| 80032 | | | | | Closed fracture of vault of skull with other and unspecified intracranial hemorrhage, with brief [less than one hour] loss of consciousness | | | | | | | | | | | | | | | | | | | | | | | | |
| 80033 | | | | | Closed fracture of vault of skull with other and unspecified intracranial hemorrhage, with moderate [1-24 hours] loss of consciousness | | | | | | | | | | | | | | | | | | | | | | | | |
| 80034 | | | | | Closed fracture of vault of skull with other and unspecified intracranial hemorrhage, with prolonged [more than 24 hours] loss of consciousness and return to pre-existing conscious level | | | | | | | | | | | | | | | | | | | | | | | | |
| 80035 | | | | | Closed fracture of vault of skull with other and unspecified intracranial hemorrhage, with prolonged [more than 24 hours] loss of consciousness, without return to pre-existing conscious level | | | | | | | | | | | | | | | | | | | | | | | | |
| 80036 | | | | | Closed fracture of vault of skull with other and unspecified intracranial hemorrhage, with loss of consciousness of unspecified duration | | | | | | | | | | | | | | | | | | | | | | | | |
| 80129 | | | | | Closed fracture of base of skull with subarachnoid, subdural, and extradural hemorrhage, with concussion, unspecified | | | | | | | | | | | | | | | | | | | | | | | | |
| 80130 | | | | | Closed fracture of base of skull with other and unspecified intracranial hemorrhage, unspecified state of consciousness | | | | | | | | | | | | | | | | | | | | | | | | |
| 80131 | | | | | Closed fracture of base of skull with other and unspecified intracranial hemorrhage, with no loss of consciousness | | | | | | | | | | | | | | | | | | | | | | | | |
| 80132 | | | | | Closed fracture of base of skull with other and unspecified intracranial hemorrhage, with brief [less than one hour] loss of consciousness | | | | | | | | | | | | | | | | | | | | | | | | |
| 80133 | | | | | Closed fracture of base of skull with other and unspecified intracranial hemorrhage, with moderate [1-24 hours] loss of consciousness | | | | | | | | | | | | | | | | | | | | | | | | |
| 80134 | | | | | Closed fracture of base of skull with other and unspecified intracranial hemorrhage, with prolonged [more than 24 hours] loss of consciousness and return to pre-existing conscious level | | | | | | | | | | | | | | | | | | | | | | | | |
| 80135 | | | | | Closed fracture of base of skull with other and unspecified intracranial hemorrhage, with prolonged [more than 24 hours] loss of consciousness, without return to pre-existing conscious level | | | | | | | | | | | | | | | | | | | | | | | | |
| 80136 | | | | | Closed fracture of base of skull with other and unspecified intracranial hemorrhage, with loss of consciousness of unspecified duration | | | | | | | | | | | | | | | | | | | | | | | | |
| 80139 | | | | | Closed fracture of base of skull with other and unspecified intracranial hemorrhage, with concussion, unspecified | | | | | | | | | | | | | | | | | | | | | | | | |
| 80140 | | | | | Closed fracture of base of skull with intracranial injury of other and unspecified nature, unspecified state of consciousness | | | | | | | | | | | | | | | | | | | | | | | | |
| 80141 | | | | | Closed fracture of base of skull with intracranial injury of other and unspecified nature, with no loss of consciousness | | | | | | | | | | | | | | | | | | | | | | | | |
| 80142 | | | | | Closed fracture of base of skull with intracranial injury of other and unspecified nature, with brief [less than one hour] loss of consciousness | | | | | | | | | | | | | | | | | | | | | | | | |
| 80143 | | | | | Closed fracture of base of skull with intracranial injury of other and unspecified nature, with moderate [1-24 hours] loss of consciousness | | | | | | | | | | | | | | | | | | | | | | | | |
| 80144 | | | | | Closed fracture of base of skull with intracranial injury of other and unspecified nature, with prolonged [more than 24 hours) loss of consciousness and return to pre-existing conscious level | | | | | | | | | | | | | | | | | | | | | | | | |
| 80145 | | | | | Closed fracture of base of skull with intracranial injury of other and unspecified nature, with prolonged [more than 24 hours] loss of consciousness, without return to pre-existing conscious level | | | | | | | | | | | | | | | | | | | | | | | | |
| 80146 | | | | | Closed fracture of base of skull with intracranial injury of other and unspecified nature, with loss of consciousness of unspecified duration | | | | | | | | | | | | | | | | | | | | | | | | |
| 80149 | | | | | Closed fracture of base of skull with intracranial injury of other and unspecified nature, with concussion, unspecified | | | | | | | | | | | | | | | | | | | | | | | | |
| 80150 | | | | | Open fracture of base of skull without mention of intracranial injury, unspecified state of consciousness | | | | | | | | | | | | | | | | | | | | | | | | |
| 80151 | | | | | Open fracture of base of skull without mention of intracranial injury, with no loss of consciousness | | | | | | | | | | | | | | | | | | | | | | | | |
| 80152 | | | | | Open fracture of base of skull without mention of intracranial injury, with brief [less than one hour] loss of consciousness | | | | | | | | | | | | | | | | | | | | | | | | |
| 80153 | | | | | Open fracture of base of skull without mention of intracranial injury, with moderate [1-24 hours] loss of consciousness | | | | | | | | | | | | | | | | | | | | | | | | |
| 80154 | | | | | Open fracture of base of skull without mention of intracranial injury, with prolonged [more than 24 hours] loss of consciousness and return to pre-existing conscious level | | | | | | | | | | | | | | | | | | | | | | | | |
| 80155 | | | | | Open fracture of base of skull without mention of intracranial injury, with prolonged [more than 24 hours] loss of consciousness, without return to pre-existing conscious level | | | | | | | | | | | | | | | | | | | | | | | | |
| 80156 | | | | | Open fracture of base of skull without mention of intracranial injury, with loss of consciousness of unspecified duration | | | | | | | | | | | | | | | | | | | | | | | | |
| 80159 | | | | | Open fracture of base of skull without mention of intracranial injury, with concussion, unspecified | | | | | | | | | | | | | | | | | | | | | | | | |
| 80160 | | | | | Open fracture of base of skull with cerebral laceration and contusion, unspecified state of consciousness | | | | | | | | | | | | | | | | | | | | | | | | |
| 80161 | | | | | Open fracture of base of skull with cerebral laceration and contusion, with no loss of consciousness | | | | | | | | | | | | | | | | | | | | | | | | |
| 80162 | | | | | Open fracture of base of skull with cerebral laceration and contusion, with brief [less than one hour] loss of consciousness | | | | | | | | | | | | | | | | | | | | | | | | |
| 80163 | | | | | Open fracture of base of skull with cerebral laceration and contusion, with moderate [1-24 hours] loss of consciousness | | | | | | | | | | | | | | | | | | | | | | | | |
| 80164 | | | | | Open fracture of base of skull with cerebral laceration and contusion, with prolonged [more than 24 hours] loss of consciousness and return to pre-existing conscious level | | | | | | | | | | | | | | | | | | | | | | | | |
| 80165 | | | | | Open fracture of base of skull with cerebral laceration and contusion, with prolonged [more than 24 hours] loss of consciousness, without return to pre-existing conscious level | | | | | | | | | | | | | | | | | | | | | | | | |
| 80166 | | | | | Open fracture of base of skull with cerebral laceration and contusion, with loss of consciousness of unspecified duration | | | | | | | | | | | | | | | | | | | | | | | | |
| 80169 | | | | | Open fracture of base of skull with cerebral laceration and contusion, with concussion, unspecified | | | | | | | | | | | | | | | | | | | | | | | | |
| 80170 | | | | | Open fracture of base of skull with subarachnoid, subdural, and extradural hemorrhage, unspecified state of consciousness | | | | | | | | | | | | | | | | | | | | | | | | |
| 80171 | | | | | Open fracture of base of skull with subarachnoid, subdural, and extradural hemorrhage, with no loss of consciousness | | | | | | | | | | | | | | | | | | | | | | | | |
| 80172 | | | | | Open fracture of base of skull with subarachnoid, subdural, and extradural hemorrhage, with brief [less than one hour] loss of consciousness | | | | | | | | | | | | | | | | | | | | | | | | |
| 80173 | | | | | Open fracture of base of skull with subarachnoid, subdural, and extradural hemorrhage, with moderate [1-24 hours] loss of consciousness | | | | | | | | | | | | | | | | | | | | | | | | |
| 85154 | | | | | Cerebellar or brain stem contusion with open intracranial wound, with prolonged [more than 24 hours] loss of consciousness and return | | | | | | | | | | | | | | | | | | | | | | | | |
| 85155 | | | | | Cerebellar or brain stem contusion with open intracranial wound, with prolonged [more than 24 hours] loss of consciousness without return to pre-existing conscious level | | | | | | | | | | | | | | | | | | | | | | | | |
| 85156 | | | | | Cerebellar or brain stem contusion with open intracranial wound, with loss of consciousness of unspecified duration | | | | | | | | | | | | | | | | | | | | | | | | |
| 85159 | | | | | Cerebellar or brain stem contusion with open intracranial wound, with concussion, unspecified | | | | | | | | | | | | | | | | | | | | | | | | |
| 85160 | | | | | Cerebellar or brain stem laceration without mention of open intracranial wound, unspecified state of consciousness | | | | | | | | | | | | | | | | | | | | | | | | |
| 85161 | | | | | Cerebellar or brain stem laceration without mention of open intracranial wound, with no loss of consciousness | | | | | | | | | | | | | | | | | | | | | | | | |
| 85162 | | | | | Cerebellar or brain stem laceration without mention of open intracranial wound, with brief [less than 1 hour] loss of consciousness | | | | | | | | | | | | | | | | | | | | | | | | |
| 85163 | | | | | Cerebellar or brain stem laceration without mention of open intracranial wound, with moderate [1-24 hours] loss of consciousness | | | | | | | | | | | | | | | | | | | | | | | | |
| 85164 | | | | | Cerebellar or brain stem laceration without mention of open intracranial wound, with prolonged [more than 24 hours] loss of consciousness and return to pre-existing conscious level | | | | | | | | | | | | | | | | | | | | | | | | |
| 85165 | | | | | Cerebellar or brain stem laceration without mention of open intracranial wound, with prolonged [more than 24 hours] loss of consciousness without return to pre-existing conscious level | | | | | | | | | | | | | | | | | | | | | | | | |
| 85166 | | | | | Cerebellar or brain stem laceration without mention of open intracranial wound, with loss of consciousness of unspecified duration | | | | | | | | | | | | | | | | | | | | | | | | |
| 85169 | | | | | Cerebellar or brain stem laceration without mention of open intracranial wound, with concussion, unspecified | | | | | | | | | | | | | | | | | | | | | | | | |
| 85170 | | | | | Cerebellar or brain stem laceration with open intracranial wound, unspecified state of consciousness | | | | | | | | | | | | | | | | | | | | | | | | |
| 85171 | | | | | Cerebellar or brain stem laceration with open intracranial wound, with no loss of consciousness | | | | | | | | | | | | | | | | | | | | | | | | |
| 85172 | | | | | Cerebellar or brain stem laceration with open intracranial wound, with brief [less than one hour] loss of consciousness | | | | | | | | | | | | | | | | | | | | | | | | |
| 85173 | | | | | Cerebellar or brain stem laceration with open intracranial wound, with moderate [1-24 hours] loss of consciousness | | | | | | | | | | | | | | | | | | | | | | | | |
| 85174 | | | | | Cerebellar or brain stem laceration with open intracranial wound, with prolonged [more than 24 hours] loss of consciousness and return to pre-existing conscious level | | | | | | | | | | | | | | | | | | | | | | | | |
| 85175 | | | | | Cerebellar or brain stem laceration with open intracranial wound, with prolonged [more than 24 hours] loss of consciousness without return to pre-existing conscious level | | | | | | | | | | | | | | | | | | | | | | | | |
| 85176 | | | | | Cerebellar or brain stem laceration with open intracranial wound, with loss of consciousness of unspecified duration | | | | | | | | | | | | | | | | | | | | | | | | |
| 85179 | | | | | Cerebellar or brain stem laceration with open intracranial wound, with concussion, unspecified | | | | | | | | | | | | | | | | | | | | | | | | |
| 85180 | | | | | Other and unspecified cerebral laceration and contusion, without mention of open intracranial wound, unspecified state of consciousness | | | | | | | | | | | | | | | | | | | | | | | | |
| 85181 | | | | | Other and unspecified cerebral laceration and contusion, without mention of open intracranial wound, with no loss of consciousness | | | | | | | | | | | | | | | | | | | | | | | | |
| 85182 | | | | | Other and unspecified cerebral laceration and contusion, without mention of open intracranial wound, with brief [less than one hour] loss of consciousness | | | | | | | | | | | | | | | | | | | | | | | | |
| 85183 | | | | | Other and unspecified cere85174bral laceration and contusion, without mention of open intracranial wound, with moderate [1-24 hours] loss of consciousness | | | | | | | | | | | | | | | | | | | | | | | | |
| 85184 | | | | | Other and unspecified cerebral laceration and contusion, without mention of open intracranial wound, with prolonged [more than 24 hours] loss of consciousness and return to pre- existing conscious level | | | | | | | | | | | | | | | | | | | | | | | | |
| 85185 | | | | | Other and unspecified cerebral laceration and contusion, without mention of open intracranial wound, with prolonged [more than 24 hours] loss of consciousness without return to pre-existing conscious level | | | | | | | | | | | | | | | | | | | | | | | | |
| 85186 | | | | | Other and unspecified cerebral laceration and contusion, without mention of open intracranial wound, with loss of consciousness of unspecified duration | | | | | | | | | | | | | | | | | | | | | | | | |
| 85189 | | | | | Other and unspecified cerebral laceration and contusion, without mention of open intracranial wound, with concussion, unspecified | | | | | | | | | | | | | | | | | | | | | | | | |
| 85190 | | | | | Other and unspecified cerebral laceration and contusion, with open intracranial wound, unspecified state of consciousness | | | | | | | | | | | | | | | | | | | | | | | | |
| 85191 | | | | | Other and unspecified cerebral laceration and contusion, with open intracranial wound, with no loss of consciousness | | | | | | | | | | | | | | | | | | | | | | | | |
| 85192 | | | | | Other and unspecified cerebral laceration and contusion, with open intracranial wound, with brief [less than one hour] loss of consciousness | | | | | | | | | | | | | | | | | | | | | | | | |
| 80039 | | | | | Closed fracture of vault of skull with other and unspecified intracranial hemorrhage, with concussion, unspecified | | | | | | | | | | | | | | | | | | | | | | | | |
| 80040 | | | | | Closed fracture of vault of skull with intracranial injury of other and unspecified nature, unspecified state of consciousness | | | | | | | | | | | | | | | | | | | | | | | | |
| 80041 | | | | | Closed fracture of vault of skull with intracranial injury of other and unspecified nature, with no loss of consciousness | | | | | | | | | | | | | | | | | | | | | | | | |
| 80042 | | | | | Closed fracture of vault of skull with intracranial injury of other and unspecified nature, with brief [less than one hour] loss of consciousness | | | | | | | | | | | | | | | | | | | | | | | | |
| 80043 | | | | | Closed fracture of vault of skull with intracranial injury of other and unspecified nature, with moderate [1-24 hours] loss of consciousness | | | | | | | | | | | | | | | | | | | | | | | | |
| 80044 | | | | | Closed fracture of vault of skull with intracranial injury of other and unspecified nature, with prolonged [more than 24 hours] loss of consciousness and return to pre-existing conscious level | | | | | | | | | | | | | | | | | | | | | | | | |
| 80045 | | | | | Closed fracture of vault of skull with intracranial injury of other and unspecified nature, with prolonged [more than 24 hours] loss of consciousness, without return to pre-existing conscious level | | | | | | | | | | | | | | | | | | | | | | | | |
| 80046 | | | | | Closed fracture of vault of skull with intracranial injury of other and unspecified nature, with loss of consciousness of unspecified duration | | | | | | | | | | | | | | | | | | | | | | | | |
| 80049 | | | | | Closed fracture of vault of skull with intracranial injury of other and unspecified nature, with concussion, unspecified | | | | | | | | | | | | | | | | | | | | | | | | |
| 80050 | | | | | Open fracture of vault of skull without mention of intracranial injury, unspecified state of consciousness | | | | | | | | | | | | | | | | | | | | | | | | |
| 80051 | | | | | Open fracture of vault of skull without mention of intracranial injury, with no loss of consciousness | | | | | | | | | | | | | | | | | | | | | | | | |
| 80052 | | | | | Open fracture of vault of skull without mention of intracranial injury, with brief [less than one hour] loss of consciousness | | | | | | | | | | | | | | | | | | | | | | | | |
| 80053 | | | | | Open fracture of vault of skull without mention of intracranial injury, with moderate [1-24 hours] loss of consciousness | | | | | | | | | | | | | | | | | | | | | | | | |
| 80054 | | | | | Open fracture of vault of skull without mention of intracranial injury, with prolonged [more than 24 hours] loss of consciousness and return to pre-existing conscious level | | | | | | | | | | | | | | | | | | | | | | | | |
| 80055 | | | | | Open fracture of vault of skull without mention of intracranial injury, with prolonged [more than 24 hours] loss of consciousness, without return to pre-existing conscious level | | | | | | | | | | | | | | | | | | | | | | | | |
| 80056 | | | | | Open fracture of vault of skull without mention of intracranial injury, with loss of consciousness of unspecified duration | | | | | | | | | | | | | | | | | | | | | | | | |
| 80059 | | | | | Open fracture of vault of skull without mention of intracranial injury, with concussion, unspecified | | | | | | | | | | | | | | | | | | | | | | | | |
| 80060 | | | | | Open fracture of vault of skull with cerebral laceration and contusion, unspecified state of consciousness | | | | | | | | | | | | | | | | | | | | | | | | |
| 80061 | | | | | Open fracture of vault of skull with cerebral laceration and contusion, with no loss of consciousness | | | | | | | | | | | | | | | | | | | | | | | | |
| 80062 | | | | | Open fracture of vault of skull with cerebral laceration and contusion, with brief [less than one hour] loss of consciousness | | | | | | | | | | | | | | | | | | | | | | | | |
| 80063 | | | | | Open fracture of vault of skull with cerebral laceration and contusion, with moderate [1-24 hours] loss of consciousness | | | | | | | | | | | | | | | | | | | | | | | | |
| 80064 | | | | | Open fracture of vault of skull with cerebral laceration and contusion, with prolonged [more than 24 hours] loss of consciousness and return to pre-existing conscious level | | | | | | | | | | | | | | | | | | | | | | | | |
| 80065 | | | | | Open fracture of vault of skull with cerebral laceration and contusion, with prolonged [more than 24 hours] loss of consciousness, without return to pre-existing conscious level | | | | | | | | | | | | | | | | | | | | | | | | |
| 80066 | | | | | Open fracture of vault of skull with cerebral laceration and contusion, with loss of consciousness of unspecified duration | | | | | | | | | | | | | | | | | | | | | | | | |
| 80069 | | | | | Open fracture of vault of skull with cerebral laceration and contusion, with concussion, unspecified | | | | | | | | | | | | | | | | | | | | | | | | |
| 80070 | | | | | Open fracture of vault of skull with subarachnoid, subdural, and extradural hemorrhage, unspecified state of consciousness | | | | | | | | | | | | | | | | | | | | | | | | |
| 80071 | | | | | Open fracture of vault of skull with subarachnoid, subdural, and extradural hemorrhage, with no loss of consciousness | | | | | | | | | | | | | | | | | | | | | | | | |
| 80072 | | | | | Open fracture of vault of skull with subarachnoid, subdural, and extradural hemorrhage, with brief [less than one hour] loss of consciousness | | | | | | | | | | | | | | | | | | | | | | | | |
| 80073 | | | | | Open fracture of vault of skull with subarachnoid, subdural, and extradural hemorrhage, with moderate [1-24 hours] loss of consciousness | | | | | | | | | | | | | | | | | | | | | | | | |
| 80074 | | | | | Open fracture of vault of skull with subarachnoid, subdural, and extradural hemorrhage, with moderate [1-24 hours] loss of consciousness | | | | | | | | | | | | | | | | | | | | | | | | |
| 80075 | | | | | Open fracture of vault of skull with subarachnoid, subdural, and extradural hemorrhage, with prolonged [more than 24 hours] loss of consciousness, without return to pre-existing conscious level | | | | | | | | | | | | | | | | | | | | | | | | |
| 80076 | | | | | Open fracture of vault of skull with subarachnoid, subdural, and extradural hemorrhage, with loss of consciousness of unspecified duration | | | | | | | | | | | | | | | | | | | | | | | | |
| 80079 | | | | | Open fracture of vault of skull with subarachnoid, subdural, and extradural hemorrhage, with concussion, unspecified | | | | | | | | | | | | | | | | | | | | | | | | |
| 80080 | | | | | Open fracture of vault of skull with other and unspecified intracranial hemorrhage, unspecified state of consciousness | | | | | | | | | | | | | | | | | | | | | | | | |
| 80081 | | | | | Open fracture of vault of skull with other and unspecified intracranial hemorrhage, with no loss of consciousness | | | | | | | | | | | | | | | | | | | | | | | | |
| 80082 | | | | | Open fracture of vault of skull with other and unspecified intracranial hemorrhage, with brief [less than one hour] loss of | | | | | | | | | | | | | | | | | | | | | | | | |
| 80083 | | | | | Open fracture of vault of skull with other and unspecified intracranial hemorrhage, with moderate [1-24 hours] loss of consciousness | | | | | | | | | | | | | | | | | | | | | | | | |
| 80084 | | | | | Open fracture of vault of skull with other and unspecified intracranial hemorrhage, with prolonged [more than 24 hours] loss of consciousness and return to pre-existing conscious level | | | | | | | | | | | | | | | | | | | | | | | | |
| 80085 | | | | | Open fracture of vault of skull with other and unspecified intracranial hemorrhage, with prolonged [more than 24 hours] loss of consciousness, without return to pre-existing conscious level | | | | | | | | | | | | | | | | | | | | | | | | |
| 80086 | | | | | Open fracture of vault of skull with other and unspecified intracranial hemorrhage, with loss of consciousness of unspecified duration | | | | | | | | | | | | | | | | | | | | | | | | |
| 80089 | | | | | Open fracture of vault of skull with other and unspecified intracranial hemorrhage, with concussion, unspecified | | | | | | | | | | | | | | | | | | | | | | | | |
| 80090 | | | | | Open fracture of vault of skull with intracranial injury of other and unspecified nature, unspecified state of consciousness | | | | | | | | | | | | | | | | | | | | | | | | |
| 80091 | | | | | Open fracture of vault of skull with intracranial injury of other and unspecified nature, with no loss of consciousness | | | | | | | | | | | | | | | | | | | | | | | | |
| 80092 | | | | | Open fracture of vault of skull with intracranial injury of other and unspecified nature, with brief [less than one hour] loss of consciousness | | | | | | | | | | | | | | | | | | | | | | | | |
| 80093 | | | | | Open fracture of vault of skull with intracranial injury of other and unspecified nature, with moderate [1-24 hours] loss of consciousness | | | | | | | | | | | | | | | | | | | | | | | | |
| 80094 | | | | | Open fracture of vault of skull with intracranial injury of other and unspecified nature, with prolonged [more than 24 hours] loss of consciousness and return to pre-existing conscious level | | | | | | | | | | | | | | | | | | | | | | | | |
| 80095 | | | | | Open fracture of vault of skull with intracranial injury of other and unspecified nature, with prolonged [more than 24 hours] loss of consciousness, without return to pre-existing conscious level | | | | | | | | | | | | | | | | | | | | | | | | |
| 80096 | | | | | Open fracture of vault of skull with intracranial injury of other and unspecified nature, with loss of consciousness of unspecified duration | | | | | | | | | | | | | | | | | | | | | | | | |
| 80099 | | | | | Open fracture of vault of skull with intracranial injury of other and unspecified nature, with concussion, unspecified | | | | | | | | | | | | | | | | | | | | | | | | |
| 80100 | | | | | Closed fracture of base of skull without mention of intra cranial injury, unspecified state of consciousness | | | | | | | | | | | | | | | | | | | | | | | | |
| 80101 | | | | | Closed fracture of base of skull without mention of intra cranial injury, with no loss of consciousness | | | | | | | | | | | | | | | | | | | | | | | | |
| 80102 | | | | | Closed fracture of base of skull without mention of intra cranial injury, with brief [less than one hour] loss of consciousness | | | | | | | | | | | | | | | | | | | | | | | | |
| 80103 | | | | | Closed fracture of base of skull without mention of intra cranial injury, with moderate [1-24 hours] loss of consciousness | | | | | | | | | | | | | | | | | | | | | | | | |
| 80104 | | | | | Closed fracture of base of skull without mention of intra cranial injury, with prolonged [more than 24 hours] loss of consciousness and return to pre-existing conscious level | | | | | | | | | | | | | | | | | | | | | | | | |
| 80105 | | | | | Closed fracture of base of skull without mention of intra cranial injury, with prolonged [more than 24 hours] loss of consciousness, without return to pre-existing conscious level | | | | | | | | | | | | | | | | | | | | | | | | |
| 80106 | | | | | Closed fracture of base of skull without mention of intra cranial injury, with loss of consciousness of unspecified duration | | | | | | | | | | | | | | | | | | | | | | | | |
| 80109 | | | | | Closed fracture of base of skull without mention of intra cranial injury, with concussion, unspecified | | | | | | | | | | | | | | | | | | | | | | | | |
| 80110 | | | | | Closed fracture of base of skull with cerebral laceration and contusion, unspecified state of consciousness | | | | | | | | | | | | | | | | | | | | | | | | |
| 80111 | | | | | Closed fracture of base of skull with cerebral laceration and contusion, with no loss of consciousness | | | | | | | | | | | | | | | | | | | | | | | | |
| 80112 | | | | | Closed fracture of base of skull with cerebral laceration and contusion, with brief [less than one hour] loss of consciousness | | | | | | | | | | | | | | | | | | | | | | | | |
| 80113 | | | | | Closed fracture of base of skull with cerebral laceration and contusion, with moderate [1-24 hours] loss of consciousness | | | | | | | | | | | | | | | | | | | | | | | | |
| 80114 | | | | | Closed fracture of base of skull with cerebral laceration and contusion, with prolonged [more than 24 hours] loss of consciousness and return to pre-existing conscious level | | | | | | | | | | | | | | | | | | | | | | | | |
| 80115 | | | | | Closed fracture of base of skull with cerebral laceration and contusion, with prolonged [more than 24 hours] loss of consciousness, without return to pre-existing conscious level | | | | | | | | | | | | | | | | | | | | | | | | |
| 80116 | | | | | Closed fracture of base of skull with cerebral laceration and contusion, with loss of consciousness of unspecified duration | | | | | | | | | | | | | | | | | | | | | | | | |
| 80119 | | | | | Closed fracture of base of skull with cerebral laceration and contusion, with concussion, unspecified | | | | | | | | | | | | | | | | | | | | | | | | |
| 80120 | | | | | Closed fracture of base of skull with subarachnoid, subdural, and extradural hemorrhage, unspecified state of consciousness | | | | | | | | | | | | | | | | | | | | | | | | |
| 80121 | | | | | Closed fracture of base of skull with subarachnoid, subdural, and extradural hemorrhage, with no loss of consciousness | | | | | | | | | | | | | | | | | | | | | | | | |
| 80122 | | | | | Closed fracture of base of skull with subarachnoid, subdural, and extradural hemorrhage, with brief [less than one hour] loss of consciousness | | | | | | | | | | | | | | | | | | | | | | | | |
| 80123 | | | | | Closed fracture of base of skull with subarachnoid, subdural, and extradural hemorrhage, with moderate [1-24 hours] loss of consciousness | | | | | | | | | | | | | | | | | | | | | | | | |
| 80124 | | | | | Closed fracture of base of skull with subarachnoid, subdural, and extradural hemorrhage, with prolonged [more than 24 hours] loss of consciousness and return to pre-existing conscious level | | | | | | | | | | | | | | | | | | | | | | | | |
| 80300 | | | | | Other closed skull fracture without mention of intracranial injury, unspecified state of consciousness | | | | | | | | | | | | | | | | | | | | | | | | |
| 80301 | | | | | Other closed skull fracture without mention of intracranial injury, with no loss of consciousness | | | | | | | | | | | | | | | | | | | | | | | | |
| 80302 | | | | | Other closed skull fracture without mention of intracranial injury, with brief [less than one hour] loss of consciousness | | | | | | | | | | | | | | | | | | | | | | | | |
| 80303 | | | | | Other closed skull fracture without mention of intracranial injury, with moderate [1-24 hours] loss of consciousness | | | | | | | | | | | | | | | | | | | | | | | | |
| 80304 | | | | | Other closed skull fracture without mention of intracranial injury, with prolonged [more than 24 hours] loss of consciousness and return to pre-existing conscious level | | | | | | | | | | | | | | | | | | | | | | | | |
| 80305 | | | | | Other closed skull fracture without mention of intracranial injury, with prolonged [more than 24 hours] loss of consciousness, without return to pre-existing conscious level | | | | | | | | | | | | | | | | | | | | | | | | |
| 80306 | | | | | Other closed skull fracture without mention of intracranial injury, with loss of consciousness of unspecified duration | | | | | | | | | | | | | | | | | | | | | | | | |
| 80309 | | | | | Other closed skull fracture without mention of intracranial injury, with concussion, unspecified | | | | | | | | | | | | | | | | | | | | | | | | |
| 80310 | | | | | Other closed skull fracture with cerebral laceration and contusion, unspecified state of consciousness | | | | | | | | | | | | | | | | | | | | | | | | |
| 80311 | | | | | Other closed skull fracture with cerebral laceration and contusion, with no loss of consciousness | | | | | | | | | | | | | | | | | | | | | | | | |
| 80312 | | | | | Other closed skull fracture with cerebral laceration and contusion, with brief [less than one hour] loss of consciousness | | | | | | | | | | | | | | | | | | | | | | | | |
| 80313 | | | | | Other closed skull fracture with cerebral laceration and contusion, with moderate [1-24 hours] loss of consciousness | | | | | | | | | | | | | | | | | | | | | | | | |
| 80314 | | | | | Other closed skull fracture with cerebral laceration and contusion, with prolonged [more than 24 hours] loss of consciousness and return to pre-existing conscious level | | | | | | | | | | | | | | | | | | | | | | | | |
| 80315 | | | | | Other closed skull fracture with cerebral laceration and contusion, with prolonged [more than 24 hours] loss of consciousness, without return to pre-existing conscious level | | | | | | | | | | | | | | | | | | | | | | | | |
| 80316 | | | | | Other closed skull fracture with cerebral laceration and contusion, with loss of consciousness of unspecified duration | | | | | | | | | | | | | | | | | | | | | | | | |
| 80319 | | | | | Other closed skull fracture with cerebral laceration and contusion, with concussion, unspecified | | | | | | | | | | | | | | | | | | | | | | | | |
| 80320 | | | | | Other closed skull fracture with subarachnoid, subdural, and extradural hemorrhage, unspecified state of consciousness | | | | | | | | | | | | | | | | | | | | | | | | |
| 80321 | | | | | Other closed skull fracture with subarachnoid, subdural, and extradural hemorrhage, with no loss of consciousness | | | | | | | | | | | | | | | | | | | | | | | | |
| 80322 | | | | | Other closed skull fracture with subarachnoid, subdural, and extradural hemorrhage, with brief [less than one hour] loss of consciousness | | | | | | | | | | | | | | | | | | | | | | | | |
| 80323 | | | | | Other closed skull fracture with subarachnoid, subdural, and extradural hemorrhage, with moderate [1-24 hours] loss of consciousness | | | | | | | | | | | | | | | | | | | | | | | | |
| 80324 | | | | | Other closed skull fracture with subarachnoid, subdural, and extradural hemorrhage, with prolonged [more than 24 hours] loss of consciousness and return to pre-existing conscious level | | | | | | | | | | | | | | | | | | | | | | | | |
| 80325 | | | | | Other closed skull fracture with subarachnoid, subdural, and extradural hemorrhage, with prolonged [more than 24 hours] loss of consciousness, without return to pre-existing conscious level | | | | | | | | | | | | | | | | | | | | | | | | |
| 80326 | | | | | Other closed skull fracture with subarachnoid, subdural, and extradural hemorrhage, with loss of consciousness of unspecified duration | | | | | | | | | | | | | | | | | | | | | | | | |
| 80329 | | | | | Other closed skull fracture with subarachnoid, subdural, and extradural hemorrhage, with concussion, unspecified | | | | | | | | | | | | | | | | | | | | | | | | |
| 80330 | | | | | Other closed skull fracture with other and unspecified intracranial hemorrhage, unspecified state of unconsciousness | | | | | | | | | | | | | | | | | | | | | | | | |
| 80331 | | | | | Other closed skull fracture with other and unspecified intracranial hemorrhage, with no loss of consciousness | | | | | | | | | | | | | | | | | | | | | | | | |
| 80332 | | | | | Other closed skull fracture with other and unspecified intracranial hemorrhage, with brief [less than one hour] loss of consciousness | | | | | | | | | | | | | | | | | | | | | | | | |
| 80333 | | | | | Other closed skull fracture with other and unspecified intracranial hemorrhage, with moderate [1-24 hours] loss of consciousness | | | | | | | | | | | | | | | | | | | | | | | | |
| 80334 | | | | | Other closed skull fracture with other and unspecified intracranial hemorrhage, with prolonged [more than 24 hours] loss of consciousness and return to pre-existing conscious level | | | | | | | | | | | | | | | | | | | | | | | | |
| 80335 | | | | | Other closed skull fracture with other and unspecified intracranial hemorrhage, with prolonged [more than 24 hours] loss of consciousness, without return to pre-existing conscious level | | | | | | | | | | | | | | | | | | | | | | | | |
| 80336 | | | | | Other closed skull fracture with other and unspecified intracranial hemorrhage, with loss of consciousness of unspecified duration | | | | | | | | | | | | | | | | | | | | | | | | |
| 80339 | | | | | Other closed skull fracture with other and unspecified intracranial hemorrhage, with concussion, unspecified | | | | | | | | | | | | | | | | | | | | | | | | |
| 80340 | | | | | Other closed skull fracture with intracranial injury of other and unspecified nature, unspecified state of consciousness | | | | | | | | | | | | | | | | | | | | | | | | |
| 80341 | | | | | Other closed skull fracture with intracranial injury of other and unspecified nature, with no loss of consciousness | | | | | | | | | | | | | | | | | | | | | | | | |
| 80342 | | | | | Other closed skull fracture with intracranial injury of other and unspecified nature, with brief [less than one hour] loss of consciousness | | | | | | | | | | | | | | | | | | | | | | | | |
| 80343 | | | | | Other closed skull fracture with intracranial injury of other and unspecified nature, with moderate [1-24 hours] loss of consciousness | | | | | | | | | | | | | | | | | | | | | | | | |
| 80344 | | | | | Other closed skull fracture with intracranial injury of other and unspecified nature, with prolonged [more than 24 hours] loss of consciousness and return to pre-existing conscious level | | | | | | | | | | | | | | | | | | | | | | | | |
| 80345 | | | | | Other closed skull fracture with intracranial injury of other and unspecified nature, with prolonged [more than 24 hours] loss of consciousness, without return to pre-existing conscious level | | | | | | | | | | | | | | | | | | | | | | | | |
| 8503 | | | | | Concussion with prolonged loss of consciousness and return to pre-existing conscious level | | | | | | | | | | | | | | | | | | | | | | | | |
| 85193 | | | | | Other and unspecified cerebral laceration and contusion, with open intracranial wound, with moderate [1-24 hours] loss of consciousness | | | | | | | | | | | | | | | | | | | | | | | | |
| 85194 | | | | | Other and unspecified cerebral laceration and contusion, with open intracranial wound, with prolonged [more than 24 hours] loss of consciousness and return to pre-existing conscious level | | | | | | | | | | | | | | | | | | | | | | | | |
| 85195 | | | | | Other and unspecified cerebral laceration and contusion, with open intracranial wound, with prolonged [more than 24 hours] loss of consciousness without return to pre-existing conscious level | | | | | | | | | | | | | | | | | | | | | | | | |
| 85196 | | | | | Other and unspecified cerebral laceration and contusion, with open intracranial wound, with loss of consciousness of unspecified duration | | | | | | | | | | | | | | | | | | | | | | | | |
| 85199 | | | | | Other and unspecified cerebral laceration and contusion, with open intracranial wound, with concussion, unspecified | | | | | | | | | | | | | | | | | | | | | | | | |
| 85200 | | | | | Subarachnoid hemorrhage following injury without mention of open intracranial wound, unspecified state of consciousness | | | | | | | | | | | | | | | | | | | | | | | | |
| 85201 | | | | | Subarachnoid hemorrhage following injury without mention of open intracranial wound, with no loss of consciousness | | | | | | | | | | | | | | | | | | | | | | | | |
| 85412 | | | | | Intracranial injury of other and unspecified nature with open intracranial wound, with brief [less than one hour] loss of consciousness | | | | | | | | | | | | | | | | | | | | | | | | |
| 85413 | | | | | Intracranial injury of other and unspecified nature with open intracranial wound, with moderate [1-24 hours] loss of consciousness | | | | | | | | | | | | | | | | | | | | | | | | |
| 85414 | | | | | Intracranial injury of other and unspecified nature with open intracranial wound, with prolonged [more than 24 hours] loss of consciousness and return to pre-existing conscious level | | | | | | | | | | | | | | | | | | | | | | | | |
| 85415 | | | | | Intracranial injury of other and unspecified nature with open intracranial wound, with prolonged [more than 24 hours] loss of consciousness without return to pre-existing conscious level | | | | | | | | | | | | | | | | | | | | | | | | |
| 85416 | | | | | Intracranial injury of other and unspecified nature with open intracranial wound, with loss of consciousness of unspecified duration | | | | | | | | | | | | | | | | | | | | | | | | |
| 85419 | | | | | Intracranial injury of other and unspecified nature with open intracranial wound, with concussion, unspecified | | | | | | | | | | | | | | | | | | | | | | | | |
| 80411 | | | | | Closed fractures involving skull or face with other bones, with cerebral laceration and contusion, with no loss of consciousness | | | | | | | | | | | | | | | | | | | | | | | | |
| 80420 | | | | | Closed fractures involving skull or face with other bones with subarachnoid, subdural, and extradural hemorrhage, unspecified state of consciousness | | | | | | | | | | | | | | | | | | | | | | | | |
| 80421 | | | | | Closed fractures involving skull or face with other bones with subarachnoid, subdural, and extradural hemorrhage, with no loss of consciousness | | | | | | | | | | | | | | | | | | | | | | | | |
| 80422 | | | | | Closed fractures involving skull or face with other bones with subarachnoid, subdural, and extradural hemorrhage, with brief [less than one hour] loss of consciousness | | | | | | | | | | | | | | | | | | | | | | | | |
| 80423 | | | | | Closed fractures involving skull or face with other bones with subarachnoid, subdural, and extradural hemorrhage, with moderate [1-24 hours] loss of consciousness | | | | | | | | | | | | | | | | | | | | | | | | |
| 80424 | | | | | Closed fractures involving skull or face with other bones with subarachnoid, subdural, and extradural hemorrhage, with prolonged [more than 24 hours] loss of consciousness and return to pre-existing conscious level | | | | | | | | | | | | | | | | | | | | | | | | |
| 80425 | | | | | Closed fractures involving skull or face with other bones with subarachnoid, subdural, and extradural hemorrhage, with prolonged [more than 24 hours] loss of consciousness, without return to pre-existing conscious level | | | | | | | | | | | | | | | | | | | | | | | | |
| 80426 | | | | | Closed fractures involving skull or face with other bones with subarachnoid, subdural, and extradural hemorrhage, with loss of consciousness of unspecified duration | | | | | | | | | | | | | | | | | | | | | | | | |
| 80429 | | | | | Closed fractures involving skull or face with other bones with subarachnoid, subdural, and extradural hemorrhage, with concussion, unspecified | | | | | | | | | | | | | | | | | | | | | | | | |
| 80430 | | | | | Closed fractures involving skull or face with other bones, with other and unspecified intracranial hemorrhage, unspecified state of consciousness | | | | | | | | | | | | | | | | | | | | | | | | |
| 80431 | | | | | Closed fractures involving skull or face with other bones, with other and unspecified intracranial hemorrhage, with no loss of consciousness | | | | | | | | | | | | | | | | | | | | | | | | |
| 80432 | | | | | Closed fractures involving skull or face with other bones, with other and unspecified intracranial hemorrhage, with brief [less than one hour] loss of consciousness | | | | | | | | | | | | | | | | | | | | | | | | |
| 80433 | | | | | Closed fractures involving skull or face with other bones, with other and unspecified intracranial hemorrhage, with moderate [1-24 hours] loss of consciousness | | | | | | | | | | | | | | | | | | | | | | | | |
| 85250 | | | | | Extradural hemorrhage following injury with open intracranial wound, unspecified state of consciousness | | | | | | | | | | | | | | | | | | | | | | | | |
| 85251 | | | | | Extradural hemorrhage following injury with open intracranial wound, with no loss of consciousness | | | | | | | | | | | | | | | | | | | | | | | | |
| 85252 | | | | | Extradural hemorrhage following injury with open intracranial wound, with brief [less than one hour] loss of consciousness | | | | | | | | | | | | | | | | | | | | | | | | |
| 85253 | | | | | Extradural hemorrhage following injury with open intracranial wound, with moderate [1-24 hours] loss of consciousness | | | | | | | | | | | | | | | | | | | | | | | | |
| 85254 | | | | | Extradural hemorrhage following injury with open intracranial wound, with prolonged [more than 24 hours] loss of consciousness and return to pre-existing conscious level | | | | | | | | | | | | | | | | | | | | | | | | |
| 85255 | | | | | Extradural hemorrhage following injury with open intracranial wound, with prolonged [more than 24 hours] loss of consciousness without return to pre-existing conscious level | | | | | | | | | | | | | | | | | | | | | | | | |
| 85256 | | | | | Extradural hemorrhage following injury with open intracranial wound, with loss of consciousness of unspecified duration | | | | | | | | | | | | | | | | | | | | | | | | |
| 85259 | | | | | Extradural hemorrhage following injury with open intracranial wound, with concussion, unspecified | | | | | | | | | | | | | | | | | | | | | | | | |
| 85300 | | | | | Other and unspecified intracranial hemorrhage following injury without mention of open intracranial wound, unspecified state of consciousness | | | | | | | | | | | | | | | | | | | | | | | | |
| 85301 | | | | | Other and unspecified intracranial hemorrhage following injury without mention of open intracranial wound, with no loss of consciousness | | | | | | | | | | | | | | | | | | | | | | | | |
| 85302 | | | | | Other and unspecified intracranial hemorrhage following injury without mention of open intracranial wound, with brief [less than one hour] loss of consciousness | | | | | | | | | | | | | | | | | | | | | | | | |
| 85303 | | | | | Other and unspecified intracranial hemorrhage following injury without mention of open intracranial wound, with moderate [1-24 hours] loss of consciousness | | | | | | | | | | | | | | | | | | | | | | | | |
| 85304 | | | | | Other and unspecified intracranial hemorrhage following injury without mention of open intracranial wound, with prolonged [more than 24 hours] loss of consciousness and return to pre- existing conscious level | | | | | | | | | | | | | | | | | | | | | | | | |
| 85305 | | | | | Other and unspecified intracranial hemorrhage following injury without mention of open intracranial wound, with prolonged [more than 24 hours] loss of consciousness without return to pre-existing conscious level | | | | | | | | | | | | | | | | | | | | | | | | |
| 85306 | | | | | Other and unspecified intracranial hemorrhage following injury without mention of open intracranial wound, with loss of consciousness of unspecified duration | | | | | | | | | | | | | | | | | | | | | | | | |
| 85309 | | | | | Other and unspecified intracranial hemorrhage following injury without mention of open intracranial wound, with concussion, unspecified | | | | | | | | | | | | | | | | | | | | | | | | |
| 85310 | | | | | Other and unspecified intracranial hemorrhage following injury with open intracranial wound, unspecified state of consciousness | | | | | | | | | | | | | | | | | | | | | | | | |
| 85311 | | | | | Other and unspecified intracranial hemorrhage following injury with open intracranial wound, with no loss of consciousness | | | | | | | | | | | | | | | | | | | | | | | | |
| 85312 | | | | | Other and unspecified intracranial hemorrhage following injury with open intracranial wound, with brief [less than one hour] loss of consciousness | | | | | | | | | | | | | | | | | | | | | | | | |
| 85313 | | | | | Other and unspecified intracranial hemorrhage following injury with open intracranial wound, with moderate [1-24 hours] loss of consciousness | | | | | | | | | | | | | | | | | | | | | | | | |
| 85314 | | | | | Other and unspecified intracranial hemorrhage following injury with open intracranial wound, with prolonged [more than 24 hours] loss of consciousness and return to pre-existing conscious level | | | | | | | | | | | | | | | | | | | | | | | | |
| 85315 | | | | | Other and unspecified intracranial hemorrhage following injury with open intracranial wound, with prolonged [more than 24 hours] loss of consciousness without return to pre-existing conscious level | | | | | | | | | | | | | | | | | | | | | | | | |
| 85316 | | | | | Other and unspecified intracranial hemorrhage following injury with open intracranial wound, with loss of consciousness of unspecified duration | | | | | | | | | | | | | | | | | | | | | | | | |
| 85319 | | | | | Other and unspecified intracranial hemorrhage following injury with open intracranial wound, with concussion, unspecified | | | | | | | | | | | | | | | | | | | | | | | | |
| 85400 | | | | | Intracranial injury of other and unspecified nature without mention of open intracranial wound, unspecified state of consciousness | | | | | | | | | | | | | | | | | | | | | | | | |
| 85401 | | | | | Intracranial injury of other and unspecified nature without mention of open intracranial wound, with no loss of consciousness | | | | | | | | | | | | | | | | | | | | | | | | |
| 85402 | | | | | Intracranial injury of other and unspecified nature without mention of open intracranial wound, with brief [less than one hour] loss of consciousness | | | | | | | | | | | | | | | | | | | | | | | | |
| 85403 | | | | | Intracranial injury of other and unspecified nature without mention of open intracranial wound, with moderate [1-24 hours] loss of consciousness | | | | | | | | | | | | | | | | | | | | | | | | |
| 85404 | | | | | Intracranial injury of other and unspecified nature without mention of open intracranial wound, with prolonged [more than 24 hours] loss of consciousness and return to pre-existing conscious level | | | | | | | | | | | | | | | | | | | | | | | | |
| 85405 | | | | | Intracranial injury of other and unspecified nature without mention of open intracranial wound, with prolonged [more than 24 hours] loss of consciousness without return to pre-existing conscious level | | | | | | | | | | | | | | | | | | | | | | | | |
| 85406 | | | | | Intracranial injury of other and unspecified nature without mention of open intracranial wound, with loss of consciousness of unspecified duration | | | | | | | | | | | | | | | | | | | | | | | | |
| 85409 | | | | | Intracranial injury of other and unspecified nature without mention of open intracranial wound, with concussion, unspecified | | | | | | | | | | | | | | | | | | | | | | | | |
| 85410 | | | | | Intracranial injury of other and unspecified nature with open intracranial wound, unspecified state of consciousness | | | | | | | | | | | | | | | | | | | | | | | | |
| 85411 | | | | | Intracranial injury of other and unspecified nature with open intracranial wound, with no loss of consciousness | | | | | | | | | | | | | | | | | | | | | | | | |
| 85412 | | | | | Intracranial injury of other and unspecified nature with open intracranial wound, with brief [less than one hour] loss of consciousness | | | | | | | | | | | | | | | | | | | | | | | | |
| 85413 | | | | | Intracranial injury of other and unspecified nature with open intracranial wound, with moderate [1-24 hours] loss of consciousness | | | | | | | | | | | | | | | | | | | | | | | | |
| 85414 | | | | | Intracranial injury of other and unspecified nature with open intracranial wound, with prolonged [more than 24 hours] loss of consciousness and return to pre-existing conscious level | | | | | | | | | | | | | | | | | | | | | | | | |
| 85415 | | | | | Intracranial injury of other and unspecified nature with open intracranial wound, with prolonged [more than 24 hours] loss of consciousness without return to pre-existing conscious level | | | | | | | | | | | | | | | | | | | | | | | | |
| 85416 | | | | | Intracranial injury of other and unspecified nature with open intracranial wound, with loss of consciousness of unspecified duration | | | | | | | | | | | | | | | | | | | | | | | | |
| 85419 | | | | | Intracranial injury of other and unspecified nature with open intracranial wound, with concussion, unspecified | | | | | | | | | | | | | | | | | | | | | | | | |
| 80434 | | | | | Closed fractures involving skull or face with other bones, with other and unspecified intracranial hemorrhage, with prolonged [more than 24 hours] loss of consciousness and return to pre- existing conscious level | | | | | | | | | | | | | | | | | | | | | | | | |
| 80435 | | | | | Closed fractures involving skull or face with other bones, with other and unspecified intracranial hemorrhage, with prolonged [more than 24 hours] loss of consciousness, without return to pre-existing conscious level | | | | | | | | | | | | | | | | | | | | | | | | |
| 80436 | | | | | Closed fractures involving skull or face with other bones, with other and unspecified intracranial hemorrhage, with loss of consciousness of unspecified duration | | | | | | | | | | | | | | | | | | | | | | | | |
| 80439 | | | | | Closed fractures involving skull or face with other bones, with other and unspecified intracranial hemorrhage, with concussion, unspecified | | | | | | | | | | | | | | | | | | | | | | | | |
| 80440 | | | | | Closed fractures involving skull or face with other bones, with intracranial injury of other and unspecified nature, unspecified state of consciousness | | | | | | | | | | | | | | | | | | | | | | | | |
| 80441 | | | | | Closed fractures involving skull or face with other bones, with intracranial injury of other and unspecified nature, with no loss of consciousness | | | | | | | | | | | | | | | | | | | | | | | | |
| 80442 | | | | | Closed fractures involving skull or face with other bones, with intracranial injury of other and unspecified nature, with brief [less than one hour] loss of consciousness | | | | | | | | | | | | | | | | | | | | | | | | |
| 80443 | | | | | Closed fractures involving skull or face with other bones, with intracranial injury of other and unspecified nature, with moderate [1-24 hours] loss of consciousness | | | | | | | | | | | | | | | | | | | | | | | | |
| 80444 | | | | | Closed fractures involving skull or face with other bones, with intracranial injury of other and unspecified nature, with prolonged [more than 24 hours] loss of consciousness and return to pre-existing conscious level | | | | | | | | | | | | | | | | | | | | | | | | |
| 80445 | | | | | Closed fractures involving skull or face with other bones, with intracranial injury of other and unspecified nature, with prolonged [more than 24 hours] loss of consciousness, without return to pre-existing conscious level | | | | | | | | | | | | | | | | | | | | | | | | |
| 80446 | | | | | Closed fractures involving skull or face with other bones, with intracranial injury of other and unspecified nature, with loss of consciousness of unspecified duration | | | | | | | | | | | | | | | | | | | | | | | | |
| 80449 | | | | | Closed fractures involving skull or face with other bones, with intracranial injury of other and unspecified nature, with concussion, unspecified | | | | | | | | | | | | | | | | | | | | | | | | |
| 80450 | | | | | Open fractures involving skull or face with other bones, without mention of intracranial injury, unspecified state of consciousness | | | | | | | | | | | | | | | | | | | | | | | | |
| 80451 | | | | | Open fractures involving skull or face with other bones, without mention of intracranial injury, with no loss of consciousness | | | | | | | | | | | | | | | | | | | | | | | | |
| 80452 | | | | | Open fractures involving skull or face with other bones, without mention of intracranial injury, with brief [less than one hour] loss of consciousness | | | | | | | | | | | | | | | | | | | | | | | | |
| 80453 | | | | | Open fractures involving skull or face with other bones, without mention of intracranial injury, with moderate [1-24 hours] loss of consciousness | | | | | | | | | | | | | | | | | | | | | | | | |
| 80454 | | | | | Open fractures involving skull or face with other bones, without mention of intracranial injury, with prolonged [more than 24 hours] loss of consciousness and return to pre-existing conscious level | | | | | | | | | | | | | | | | | | | | | | | | |
| 80455 | | | | | Open fractures involving skull or face with other bones, without mention of intracranial injury, with prolonged [more than 24 hours] loss of consciousness, without return to pre-existing conscious level | | | | | | | | | | | | | | | | | | | | | | | | |
| 80456 | | | | | Open fractures involving skull or face with other bones, without mention of intracranial injury, with loss of consciousness of unspecified duration | | | | | | | | | | | | | | | | | | | | | | | | |
| 80459 | | | | | Open fractures involving skull or face with other bones, without mention of intracranial injury, with concussion, unspecified | | | | | | | | | | | | | | | | | | | | | | | | |
| 80460 | | | | | Open fractures involving skull or face with other bones, with cerebral laceration and contusion, unspecified state of consciousness | | | | | | | | | | | | | | | | | | | | | | | | |
| 80461 | | | | | Open fractures involving skull or face with other bones, with cerebral laceration and contusion, with no loss of consciousness | | | | | | | | | | | | | | | | | | | | | | | | |
| 80462 | | | | | Open fractures involving skull or face with other bones, with cerebral laceration and contusion, with brief [less than one hour] loss of consciousness | | | | | | | | | | | | | | | | | | | | | | | | |
| 80463 | | | | | Open fractures involving skull or face with other bones, with cerebral laceration and contusion, with moderate [1-24 hours] loss of consciousness | | | | | | | | | | | | | | | | | | | | | | | | |
| 80464 | | | | | Open fractures involving skull or face with other bones, with cerebral laceration and contusion, with prolonged [more than 24 hours] loss of consciousness and return to pre-existing conscious level | | | | | | | | | | | | | | | | | | | | | | | | |
| 80465 | | | | | Open fractures involving skull or face with other bones, with cerebral laceration and contusion, with prolonged [more than 24 hours] loss of consciousness, without return to pre-existing conscious level | | | | | | | | | | | | | | | | | | | | | | | | |
| 80466 | | | | | Open fractures involving skull or face with other bones, with cerebral laceration and contusion, with loss of consciousness of unspecified duration | | | | | | | | | | | | | | | | | | | | | | | | |
| 80469 | | | | | Open fractures involving skull or face with other bones, with cerebral laceration and contusion, with concussion, unspecified | | | | | | | | | | | | | | | | | | | | | | | | |
| 80470 | | | | | Open fractures involving skull or face with other bones with subarachnoid, subdural, and extradural hemorrhage, unspecified state of consciousness | | | | | | | | | | | | | | | | | | | | | | | | |
| 80471 | | | | | Open fractures involving skull or face with other bones with subarachnoid, subdural, and extradural hemorrhage, with no loss of consciousness | | | | | | | | | | | | | | | | | | | | | | | | |
| 80472 | | | | | Open fractures involving skull or face with other bones with subarachnoid, subdural, and extradural hemorrhage, with brief [less than one hour] loss of consciousness | | | | | | | | | | | | | | | | | | | | | | | | |
| 80473 | | | | | Open fractures involving skull or face with other bones with subarachnoid, subdural, and extradural hemorrhage, with moderate [1-24 hours] loss of consciousness | | | | | | | | | | | | | | | | | | | | | | | | |
| 85103 | | | | | Cortex (cerebral) contusion without mention of open intracranial wound, with moderate [1-24 hours] loss of consciousness | | | | | | | | | | | | | | | | | | | | | | | | |
| 85104 | | | | | Cortex (cerebral) contusion without mention of open intracranial wound, with prolonged [more than 24 hours] loss of consciousness and return to pre-existing conscious level | | | | | | | | | | | | | | | | | | | | | | | | |
| 85105 | | | | | Cortex (cerebral) contusion without mention of open intracranial wound, with prolonged [more than 24 hours] loss of consciousness without return to pre-existing conscious level | | | | | | | | | | | | | | | | | | | | | | | | |
| 85106 | | | | | Cortex (cerebral) contusion without mention of open intracranial wound, with loss of consciousness of unspecified duration | | | | | | | | | | | | | | | | | | | | | | | | |
| 80346 | | | | | Other closed skull fracture with intracranial injury of other and unspecified nature, with loss of consciousness of unspecified duration | | | | | | | | | | | | | | | | | | | | | | | | |
| 80349 | | | | | Other closed skull fracture with intracranial injury of other and unspecified nature, with concussion, unspecified | | | | | | | | | | | | | | | | | | | | | | | | |
| 80350 | | | | | Other open skull fracture without mention of injury, unspecified state of consciousness | | | | | | | | | | | | | | | | | | | | | | | | |
| 80351 | | | | | Other open skull fracture without mention of intracranial injury, with no loss of consciousness | | | | | | | | | | | | | | | | | | | | | | | | |
| 80352 | | | | | Other open skull fracture without mention of intracranial injury, with brief [less than one hour] loss of consciousness | | | | | | | | | | | | | | | | | | | | | | | | |
| 80353 | | | | | Other open skull fracture without mention of intracranial injury, with moderate [1-24 hours] loss of consciousness | | | | | | | | | | | | | | | | | | | | | | | | |
| 80354 | | | | | Other open skull fracture without mention of intracranial injury, with prolonged [more than 24 hours] loss of consciousness and return to pre-existing conscious level | | | | | | | | | | | | | | | | | | | | | | | | |
| 80355 | | | | | Other open skull fracture without mention of intracranial injury, with prolonged [more than 24 hours] loss of consciousness, without return to pre-existing conscious level | | | | | | | | | | | | | | | | | | | | | | | | |
| 80356 | | | | | Other open skull fracture without mention of intracranial injury, with loss of consciousness of unspecified duration | | | | | | | | | | | | | | | | | | | | | | | | |
| 80359 | | | | | Other open skull fracture without mention of intracranial injury, with concussion, unspecified | | | | | | | | | | | | | | | | | | | | | | | | |
| 80360 | | | | | Other open skull fracture with cerebral laceration and contusion, unspecified state of consciousness | | | | | | | | | | | | | | | | | | | | | | | | |
| 80361 | | | | | Other open skull fracture with cerebral laceration and contusion, with no loss of consciousness | | | | | | | | | | | | | | | | | | | | | | | | |
| 80362 | | | | | Other open skull fracture with cerebral laceration and contusion, with brief [less than one hour] loss of consciousness | | | | | | | | | | | | | | | | | | | | | | | | |
| 80363 | | | | | Other open skull fracture with cerebral laceration and contusion, with moderate [1-24 hours] loss of consciousness | | | | | | | | | | | | | | | | | | | | | | | | |
| 80364 | | | | | Other open skull fracture with cerebral laceration and contusion, with prolonged [more than 24 hours] loss of consciousness and return to pre-existing conscious level | | | | | | | | | | | | | | | | | | | | | | | | |
| 80365 | | | | | Other open skull fracture with cerebral laceration and contusion, with prolonged [more than 24 hours] loss of consciousness, without return to pre-existing conscious level | | | | | | | | | | | | | | | | | | | | | | | | |
| 80366 | | | | | Other open skull fracture with cerebral laceration and contusion, with loss of consciousness of unspecified duration | | | | | | | | | | | | | | | | | | | | | | | | |
| 80369 | | | | | Other open skull fracture with cerebral laceration and contusion, with concussion, unspecified | | | | | | | | | | | | | | | | | | | | | | | | |
| 80370 | | | | | Other open skull fracture with subarachnoid, subdural, and extradural hemorrhage, unspecified state of consciousness | | | | | | | | | | | | | | | | | | | | | | | | |
| 80371 | | | | | Other open skull fracture with subarachnoid, subdural, and extradural hemorrhage, with no loss of consciousness | | | | | | | | | | | | | | | | | | | | | | | | |
| 80372 | | | | | Other open skull fracture with subarachnoid, subdural, and extradural hemorrhage, with brief [less than one hour] loss of consciousness | | | | | | | | | | | | | | | | | | | | | | | | |
| 80373 | | | | | Other open skull fracture with subarachnoid, subdural, and extradural hemorrhage, with moderate [1-24 hours] loss of consciousness | | | | | | | | | | | | | | | | | | | | | | | | |
| 80374 | | | | | Other open skull fracture with subarachnoid, subdural, and extradural hemorrhage, with prolonged [more than 24 hours] loss of consciousness and return to pre-existing conscious level | | | | | | | | | | | | | | | | | | | | | | | | |
| 80375 | | | | | Other open skull fracture with subarachnoid, subdural, and extradural hemorrhage, with prolonged [more than 24 hours] loss of consciousness, without return to pre-existing conscious level | | | | | | | | | | | | | | | | | | | | | | | | |
| 80376 | | | | | Other open skull fracture with subarachnoid, subdural, and extradural hemorrhage, with loss of consciousness of unspecified duration | | | | | | | | | | | | | | | | | | | | | | | | |
| 80379 | | | | | Other open skull fracture with subarachnoid, subdural, and extradural hemorrhage, with concussion, unspecified | | | | | | | | | | | | | | | | | | | | | | | | |
| 80380 | | | | | Other open skull fracture with other and unspecified intracranial hemorrhage, unspecified state of consciousness | | | | | | | | | | | | | | | | | | | | | | | | |
| 80381 | | | | | Other open skull fracture with other and unspecified intracranial hemorrhage, with no loss of consciousness | | | | | | | | | | | | | | | | | | | | | | | | |
| 80382 | | | | | Other open skull fracture with other and unspecified intracranial hemorrhage, with brief [less than one hour] loss of consciousness | | | | | | | | | | | | | | | | | | | | | | | | |
| 80383 | | | | | Other open skull fracture with other and unspecified intracranial hemorrhage, with moderate [1-24 hours] loss of consciousness | | | | | | | | | | | | | | | | | | | | | | | | |
| 80384 | | | | | Other open skull fracture with other and unspecified intracranial hemorrhage, with prolonged [more than 24 hours] loss of consciousness and return to pre-existing conscious level | | | | | | | | | | | | | | | | | | | | | | | | |
| 80385 | | | | | Other open skull fracture with other and unspecified intracranial hemorrhage, with prolonged [more than 24 hours] loss of consciousness, without return to pre-existing conscious level | | | | | | | | | | | | | | | | | | | | | | | | |
| 80386 | | | | | Other open skull fracture with other and unspecified intracranial hemorrhage, with loss of consciousness of unspecified duration | | | | | | | | | | | | | | | | | | | | | | | | |
| 80389 | | | | | Other open skull fracture with other and unspecified intracranial hemorrhage, with concussion, unspecified | | | | | | | | | | | | | | | | | | | | | | | | |
| 80390 | | | | | Other open skull fracture with intracranial injury of other and unspecified nature, unspecified state of consciousness | | | | | | | | | | | | | | | | | | | | | | | | |
| 80391 | | | | | Other open skull fracture with intracranial injury of other and unspecified nature, with no loss of consciousness | | | | | | | | | | | | | | | | | | | | | | | | |
| 80392 | | | | | Other open skull fracture with intracranial injury of other and unspecified nature, with brief [less than one hour] loss of consciousness | | | | | | | | | | | | | | | | | | | | | | | | |
| 80393 | | | | | Other open skull fracture with intracranial injury of other and unspecified nature, with moderate [1-24 hours] loss of consciousness | | | | | | | | | | | | | | | | | | | | | | | | |
| 80394 | | | | | Other open skull fracture with intracranial injury of other and unspecified nature, with prolonged [more than 24 hours] loss of consciousness and return to pre-existing conscious level | | | | | | | | | | | | | | | | | | | | | | | | |
| 80395 | | | | | Other open skull fracture with intracranial injury of other and unspecified nature, with prolonged [more than 24 hours] loss of consciousness, without return to pre-existing conscious level | | | | | | | | | | | | | | | | | | | | | | | | |
| 80396 | | | | | Other open skull fracture with intracranial injury of other and unspecified nature, with loss of consciousness of unspecified duration | | | | | | | | | | | | | | | | | | | | | | | | |
| 80399 | | | | | Other open skull fracture with intracranial injury of other and unspecified nature, with concussion, unspecified | | | | | | | | | | | | | | | | | | | | | | | | |
| 80400 | | | | | Closed fractures involving skull or face with other bones, without mention of intracranial injury, unspecified state of consciousness | | | | | | | | | | | | | | | | | | | | | | | | |
| 80401 | | | | | Closed fractures involving skull or face with other bones, without mention of intracranial injury, with no loss of consciousness | | | | | | | | | | | | | | | | | | | | | | | | |
| 80402 | | | | | Closed fractures involving skull or face with other bones, without mention of intracranial injury, with brief [less than one hour] loss of consciousness | | | | | | | | | | | | | | | | | | | | | | | | |
| 80403 | | | | | Closed fractures involving skull or face with other bones, without mention of intracranial injury, with moderate [1-24 hours] loss of consciousness | | | | | | | | | | | | | | | | | | | | | | | | |
| 80404 | | | | | Closed fractures involving skull or face with other bones, without mention or intracranial injury, with prolonged [more than 24 hours] loss of consciousness and return to pre-existing conscious level | | | | | | | | | | | | | | | | | | | | | | | | |
| 80405 | | | | | Closed fractures involving skull of face with other bones, without mention of intracranial injury, with prolonged [more than 24 hours] loss of consciousness, without return to pre-existing conscious level | | | | | | | | | | | | | | | | | | | | | | | | |
| 80406 | | | | | Closed fractures involving skull of face with other bones, without mention of intracranial injury, with loss of consciousness of unspecified duration | | | | | | | | | | | | | | | | | | | | | | | | |
| 80409 | | | | | Closed fractures involving skull of face with other bones, without mention of intracranial injury, with concussion, unspecified | | | | | | | | | | | | | | | | | | | | | | | | |
| 80410 | | | | | Closed fractures involving skull or face with other bones, with cerebral laceration and contusion, unspecified state of consciousness | | | | | | | | | | | | | | | | | | | | | | | | |
| 80474 | | | | | Open fractures involving skull or face with other bones with subarachnoid, subdural, and extradural hemorrhage, with prolonged [more than 24 hours] loss of consciousness and return to pre-existing conscious level | | | | | | | | | | | | | | | | | | | | | | | | |
| 80475 | | | | | Open fractures involving skull or face with other bones with subarachnoid, subdural, and extradural hemorrhage, with prolonged [more than 24 hours] loss of consciousness, without return to pre-existing conscious level | | | | | | | | | | | | | | | | | | | | | | | | |
| 80476 | | | | | Open fractures involving skull or face with other bones with subarachnoid, subdural, and extradural hemorrhage, with loss of consciousness of unspecified duration | | | | | | | | | | | | | | | | | | | | | | | | |
| 80479 | | | | | Open fractures involving skull or face with other bones with subarachnoid, subdural, and extradural hemorrhage, with concussion, unspecified | | | | | | | | | | | | | | | | | | | | | | | | |
| 80480 | | | | | Open fractures involving skull or face with other bones, with other and unspecified intracranial hemorrhage, unspecified state of consciousness | | | | | | | | | | | | | | | | | | | | | | | | |
| 80481 | | | | | Open fractures involving skull or face with other bones, with other and unspecified intracranial hemorrhage, with no loss of consciousness | | | | | | | | | | | | | | | | | | | | | | | | |
| 80482 | | | | | Open fractures involving skull or face with other bones, with other and unspecified intracranial hemorrhage, with brief [less than one hour] loss of consciousness | | | | | | | | | | | | | | | | | | | | | | | | |
| 80483 | | | | | Open fractures involving skull or face with other bones, with other and unspecified intracranial hemorrhage, with moderate [1-24 hours] loss of consciousness | | | | | | | | | | | | | | | | | | | | | | | | |
| 80484 | | | | | Open fractures involving skull or face with other bones, with other and unspecified intracranial hemorrhage, with prolonged [more than 24 hours] loss of consciousness and return to pre-existing conscious level | | | | | | | | | | | | | | | | | | | | | | | | |
| 80485 | | | | | Open fractures involving skull or face with other bones, with other and unspecified intracranial hemorrhage, with prolonged [more than 24 hours] loss consciousness, without return to pre-existing conscious level | | | | | | | | | | | | | | | | | | | | | | | | |
| 80486 | | | | | Open fractures involving skull or face with other bones, with other and unspecified intracranial hemorrhage, with loss of consciousness of unspecified duration | | | | | | | | | | | | | | | | | | | | | | | | |
| 80489 | | | | | Open fractures involving skull or face with other bones, with other and unspecified intracranial hemorrhage, with concussion, unspecified | | | | | | | | | | | | | | | | | | | | | | | | |
| 80490 | | | | | Open fractures involving skull or face with other bones, with intracranial injury of other and unspecified nature, unspecified state of consciousness | | | | | | | | | | | | | | | | | | | | | | | | |
| 80491 | | | | | Open fractures involving skull or face with other bones, with intracranial injury of other and unspecified nature, with no loss of consciousness | | | | | | | | | | | | | | | | | | | | | | | | |
| 80492 | | | | | Open fractures involving skull or face with other bones, with intracranial injury of other and unspecified nature, with brief [less than one hour] loss of consciousness | | | | | | | | | | | | | | | | | | | | | | | | |
| 80493 | | | | | Open fractures involving skull or face with other bones, with intracranial injury of other and unspecified nature, with moderate [1-24 hours] loss of consciousness | | | | | | | | | | | | | | | | | | | | | | | | |
| 80494 | | | | | Open fractures involving skull or face with other bones, with intracranial injury of other and unspecified nature, with prolonged [more than 24 hours] loss of consciousness and return to pre-existing conscious level | | | | | | | | | | | | | | | | | | | | | | | | |
| 80495 | | | | | Open fractures involving skull or face with other bones, with intracranial injury of other and unspecified nature, with prolonged [more than 24 hours] loss of consciousness without return to pre-existing conscious level | | | | | | | | | | | | | | | | | | | | | | | | |
| 80496 | | | | | Open fractures involving skull or face with other bones, with intracranial injury of other and unspecified nature, with loss of consciousness of unspecified duration | | | | | | | | | | | | | | | | | | | | | | | | |
| 80499 | | | | | Open fractures involving skull or face with other bones, with intracranial injury of other and unspecified nature, with concussion, unspecified | | | | | | | | | | | | | | | | | | | | | | | | |
| 85109 | | | | | Cortex (cerebral) contusion without mention of open intracranial wound, with concussion, unspecified | | | | | | | | | | | | | | | | | | | | | | | | |
| 85110 | | | | | Cortex (cerebral) contusion with open intracranial wound, unspecified state of consciousness | | | | | | | | | | | | | | | | | | | | | | | | |
| 85111 | | | | | Cortex (cerebral) contusion with open intracranial wound, with no loss of consciousness | | | | | | | | | | | | | | | | | | | | | | | | |
| 85112 | | | | | Cortex (cerebral) contusion with open intracranial wound, with brief [less than one hour] loss of consciousness | | | | | | | | | | | | | | | | | | | | | | | | |
| 85113 | | | | | Cortex (cerebral) contusion with open intracranial wound, with moderate [1-24 hours] loss of consciousness | | | | | | | | | | | | | | | | | | | | | | | | |
| 85114 | | | | | Cortex (cerebral) contusion with open intracranial wound, with prolonged [more than 24 hours] loss of consciousness and return to pre-existing conscious level | | | | | | | | | | | | | | | | | | | | | | | | |
| 85115 | | | | | Cortex (cerebral) contusion with open intracranial wound, with prolonged [more than 24 hours] loss of consciousness without return to pre-existing conscious level | | | | | | | | | | | | | | | | | | | | | | | | |
| 85116 | | | | | Cortex (cerebral) contusion with open intracranial wound, with loss of consciousness of unspecified duration | | | | | | | | | | | | | | | | | | | | | | | | |
| 85119 | | | | | Cortex (cerebral) contusion with open intracranial wound, with concussion, unspecified | | | | | | | | | | | | | | | | | | | | | | | | |
| 85120 | | | | | Cortex (cerebral) laceration without mention of open intracranial wound, unspecified state of consciousness | | | | | | | | | | | | | | | | | | | | | | | | |
| 85121 | | | | | Cortex (cerebral) laceration without mention of open intracranial wound, with no loss of consciousness | | | | | | | | | | | | | | | | | | | | | | | | |
| 85122 | | | | | Cortex (cerebral) laceration without mention of open intracranial wound, with brief [less than one hour] loss of consciousness | | | | | | | | | | | | | | | | | | | | | | | | |
| 85123 | | | | | Cortex (cerebral) laceration without mention of open intracranial wound, with moderate [1-24 hours] loss of consciousness | | | | | | | | | | | | | | | | | | | | | | | | |
| 85124 | | | | | Cortex (cerebral) laceration without mention of open intracranial wound, with prolonged [more than 24 hours] loss of consciousness and return to pre-existing conscious level | | | | | | | | | | | | | | | | | | | | | | | | |
| 85125 | | | | | Cortex (cerebral) laceration without mention of open intracranial wound, with prolonged [more than 24 hours] loss of consciousness | | | | | | | | | | | | | | | | | | | | | | | | |
| 85126 | | | | | Cortex (cerebral) laceration without mention of open intracranial wound, with loss of consciousness of unspecified duration | | | | | | | | | | | | | | | | | | | | | | | | |
| 85129 | | | | | Cortex (cerebral) laceration without mention of open intracranial wound, with concussion, unspecified | | | | | | | | | | | | | | | | | | | | | | | | |
| 85130 | | | | | Cortex (cerebral) laceration with open intracranial wound, unspecified state of consciousness | | | | | | | | | | | | | | | | | | | | | | | | |
| 85131 | | | | | Cortex (cerebral) laceration with open intracranial wound, with no loss of consciousness | | | | | | | | | | | | | | | | | | | | | | | | |
| 85132 | | | | | Cortex (cerebral) laceration with open intracranial wound, with brief [less than one hour] loss of consciousness | | | | | | | | | | | | | | | | | | | | | | | | |
| 85133 | | | | | Cortex (cerebral) laceration with open intracranial wound, with moderate [1-24 hours] loss of consciousness | | | | | | | | | | | | | | | | | | | | | | | | |
| 85134 | | | | | Cortex (cerebral) laceration with open intracranial wound, with prolonged [more than 24 hours] loss of consciousness and return to pre-existing conscious level | | | | | | | | | | | | | | | | | | | | | | | | |
| 85135 | | | | | Cortex (cerebral) laceration with open intracranial wound, with prolonged [more than 24 hours] loss of consciousness without return to pre-existing conscious level | | | | | | | | | | | | | | | | | | | | | | | | |
| 85136 | | | | | Cortex (cerebral) laceration with open intracranial wound, with loss of consciousness of unspecified duration | | | | | | | | | | | | | | | | | | | | | | | | |
| 85139 | | | | | Cortex (cerebral) laceration with open intracranial wound, with concussion, unspecified | | | | | | | | | | | | | | | | | | | | | | | | |
| 85140 | | | | | Cerebellar or brain stem contusion without mention of open intracranial wound, unspecified state of consciousness | | | | | | | | | | | | | | | | | | | | | | | | |
| 85141 | | | | | Cerebellar or brain stem contusion without mention of open intracranial wound, with no loss of consciousness | | | | | | | | | | | | | | | | | | | | | | | | |
| 85142 | | | | | Cerebellar or brain stem contusion without mention of open intracranial wound, with brief [less than one hour] loss of consciousness | | | | | | | | | | | | | | | | | | | | | | | | |
| 85143 | | | | | Cerebellar or brain stem contusion without mention of open intracranial wound, with moderate [1-24 hours] loss of consciousness | | | | | | | | | | | | | | | | | | | | | | | | |
| 85144 | | | | | Cerebellar or brain stem contusion without mention of open intracranial wound, with prolonged [more than 24 hours] loss consciousness and return to pre-existing conscious level | | | | | | | | | | | | | | | | | | | | | | | | |
| 85145 | | | | | Cerebellar or brain stem contusion without mention of open intracranial wound, with prolonged [more than 24 hours] loss of consciousness without return to pre-existing conscious level | | | | | | | | | | | | | | | | | | | | | | | | |
| 85146 | | | | | Cerebellar or brain stem contusion without mention of open intracranial wound, with loss of consciousness of unspecified duration | | | | | | | | | | | | | | | | | | | | | | | | |
| 85149 | | | | | Cerebellar or brain stem contusion without mention of open intracranial wound, with concussion, unspecified | | | | | | | | | | | | | | | | | | | | | | | | |
| 85150 | | | | | Cerebellar or brain stem contusion with open intracranial wound, unspecified state of consciousness | | | | | | | | | | | | | | | | | | | | | | | | |
| 85151 | | | | | Cerebellar or brain stem contusion with open intracranial wound, with no loss of consciousness | | | | | | | | | | | | | | | | | | | | | | | | |
| 85152 | | | | | Cerebellar or brain stem contusion with open intracranial wound, with brief [less than one hour] loss of consciousness | | | | | | | | | | | | | | | | | | | | | | | | |
| 85153 | | | | | Cerebellar or brain stem contusion with open intracranial wound, with moderate [1-24 hours] loss of consciousness | | | | | | | | | | | | | | | | | | | | | | | | |
| P108 | | | | | Other intracranial lacerations and hemorrhages due to birth injury | | | | | | | | | | | | | | | | | | | | | | | | |
| P109 | | | | | Unspecified intracranial laceration and hemorrhage due to birth injury | | | | | | | | | | | | | | | | | | | | | | | | |
| P258 | | | | | Other intracranial (nontraumatic) hemorrhages of newborn | | | | | | | | | | | | | | | | | | | | | | | | |
| S06810A | | | | | Injury of right internal carotid artery, intracranial portion, not elsewhere classified without loss of consciousness, initial encounter | | | | | | | | | | | | | | | | | | | | | | | | |
| S06810D | | | | | Injury of right internal carotid artery, intracranial portion, not elsewhere classified without loss of consciousness, subsequent encounter | | | | | | | | | | | | | | | | | | | | | | | | |
| S06810S | | | | | Injury of right internal carotid artery, intracranial portion, not elsewhere classified without loss of consciousness, sequela | | | | | | | | | | | | | | | | | | | | | | | | |
| S06811A | | | | | Injury of right internal carotid artery, intracranial portion, not elsewhere classified with loss of consciousness of 30 minutes or less, initial encounter | | | | | | | | | | | | | | | | | | | | | | | | |
| S06811D | | | | | Injury of right internal carotid artery, intracranial portion, not elsewhere classified with loss of consciousness of 30 minutes or less, subsequent encounter | | | | | | | | | | | | | | | | | | | | | | | | |
| S06811S | | | | | Injury of right internal carotid artery, intracranial portion, not elsewhere classified with loss of consciousness of 30 minutes or less, sequela | | | | | | | | | | | | | | | | | | | | | | | | |
| S06812A | | | | | Injury of right internal carotid artery, intracranial portion, not elsewhere classified with loss of consciousness of 31 minutes to 59 minutes, initial encounter | | | | | | | | | | | | | | | | | | | | | | | | |
| S06812D | | | | | Injury of right internal carotid artery, intracranial portion, not elsewhere classified with loss of consciousness of 31 minutes to 59 minutes, subsequent encounter | | | | | | | | | | | | | | | | | | | | | | | | |
| S06812D | | | | | Injury of right internal carotid artery, intracranial portion, not elsewhere classified with loss of consciousness of 31 minutes to 59 minutes, subsequent encounter | | | | | | | | | | | | | | | | | | | | | | | | |
| S06812S | | | | | Injury of right internal carotid artery, intracranial portion, not elsewhere classified with loss of consciousness of 31 minutes to 59 minutes, sequela | | | | | | | | | | | | | | | | | | | | | | | | |
| S06813A | | | | | Injury of right internal carotid artery, intracranial portion, not elsewhere classified with loss of consciousness of 1 hour to 5 hours 59 minutes, initial encounter | | | | | | | | | | | | | | | | | | | | | | | | |
| S06813D | | | | | Injury of right internal carotid artery, intracranial portion, not elsewhere classified with loss of consciousness of 1 hour to 5 hours 59 minutes, subsequent encounter | | | | | | | | | | | | | | | | | | | | | | | | |
| S06813S | | | | | Injury of right internal carotid artery, intracranial portion, not elsewhere classified with loss of consciousness of 1 hour to 5 hours 59 minutes, sequela | | | | | | | | | | | | | | | | | | | | | | | | |
| S06814A | | | | | Injury of right internal carotid artery, intracranial portion, not elsewhere classified with loss of consciousness of 6 hours to 24 hours, initial encounter | | | | | | | | | | | | | | | | | | | | | | | | |
| S06814D | | | | | Injury of right internal carotid artery, intracranial portion, not elsewhere classified with loss of consciousness of 6 hours to 24 hours, subsequent encounter | | | | | | | | | | | | | | | | | | | | | | | | |
| S06814S | | | | | Injury of right internal carotid artery, intracranial portion, not elsewhere classified with loss of consciousness of 6 hours to 24 hours, sequela | | | | | | | | | | | | | | | | | | | | | | | | |
| S06815A | | | | | Injury of right internal carotid artery, intracranial portion, not elsewhere classified with loss of consciousness greater than 24 hours with return to pre-existing conscious level, initial encounter | | | | | | | | | | | | | | | | | | | | | | | | |
| S06815D | | | | | Injury of right internal carotid artery, intracranial portion, not elsewhere classified with loss of consciousness greater than 24 hours with return to pre-existing conscious level, subsequent encounter | | | | | | | | | | | | | | | | | | | | | | | | |
| S06815S | | | | | Injury of right internal carotid artery, intracranial portion, not elsewhere classified with loss of consciousness greater than 24 hours with return to pre-existing conscious level, sequela | | | | | | | | | | | | | | | | | | | | | | | | |
| S06816A | | | | | Injury of right internal carotid artery, intracranial portion, not elsewhere classified with loss of consciousness greater than 24 hours without return to pre-existing conscious level with patient surviving, initial encounter | | | | | | | | | | | | | | | | | | | | | | | | |
| S079XXA | | | | | Crushing injury of head, part unspecified, initial encounter | | | | | | | | | | | | | | | | | | | | | | | | |
| S06816D | | | | | Injury of right internal carotid artery, intracranial portion, not elsewhere classified with loss of consciousness greater than 24 hours without return to pre-existing conscious level with patient surviving, subsequent encounter | | | | | | | | | | | | | | | | | | | | | | | | |
| S06816S | | | | | Injury of right internal carotid artery, intracranial portion, not elsewhere classified with loss of consciousness greater than 24 hours without return to pre-existing conscious level with patient surviving, sequela | | | | | | | | | | | | | | | | | | | | | | | | |
| S06817A | | | | | Injury of right internal carotid artery, intracranial portion, not elsewhere classified with loss of consciousness of any duration with death due to brain injury prior to regaining consciousness, initial encounter | | | | | | | | | | | | | | | | | | | | | | | | |
| S06818A | | | | | Injury of right internal carotid artery, intracranial portion, not elsewhere classified with loss of consciousness of any duration with death due to other cause prior to regaining consciousness, initial encounter | | | | | | | | | | | | | | | | | | | | | | | | |
| S06819A | | | | | Injury of right internal carotid artery, intracranial portion, not elsewhere classified with loss of consciousness of unspecified duration, initial encounter | | | | | | | | | | | | | | | | | | | | | | | | |
| S06819D | | | | | Injury of right internal carotid artery, intracranial portion, not elsewhere classified with loss of consciousness of unspecified duration, subsequent encounter | | | | | | | | | | | | | | | | | | | | | | | | |
| S06819S | | | | | Injury of right internal carotid artery, intracranial portion, not elsewhere classified with loss of consciousness of unspecified duration, sequela | | | | | | | | | | | | | | | | | | | | | | | | |
| S06820A | | | | | Injury of left internal carotid artery, intracranial portion, not elsewhere classified without loss of consciousness, initial encounter | | | | | | | | | | | | | | | | | | | | | | | | |
| S06820D | | | | | Injury of left internal carotid artery, intracranial portion, not elsewhere classified without loss of consciousness, subsequent encounter | | | | | | | | | | | | | | | | | | | | | | | | |
| S06820S | | | | | Injury of left internal carotid artery, intracranial portion, not elsewhere classified without loss of consciousness, sequela | | | | | | | | | | | | | | | | | | | | | | | | |
| S06821A | | | | | Injury of left internal carotid artery, intracranial portion, not elsewhere classified with loss of consciousness of 30 minutes or less, initial encounter | | | | | | | | | | | | | | | | | | | | | | | | |
| S06821D | | | | | Injury of left internal carotid artery, intracranial portion, not elsewhere classified with loss of consciousness of 30 minutes or less, subsequent encounter | | | | | | | | | | | | | | | | | | | | | | | | |
| S06821S | | | | | Injury of left internal carotid artery, intracranial portion, not elsewhere classified with loss of consciousness of 30 minutes or less, sequela | | | | | | | | | | | | | | | | | | | | | | | | |
| S06822A | | | | | Injury of left internal carotid artery, intracranial portion, not elsewhere classified with loss of consciousness of 31 minutes to 59 minutes, initial encounter | | | | | | | | | | | | | | | | | | | | | | | | |
| S06822D | | | | | Injury of left internal carotid artery, intracranial portion, not elsewhere classified with loss of consciousness of 31 minutes to 59 minutes, subsequent encounter | | | | | | | | | | | | | | | | | | | | | | | | |
| S06822S | | | | | Injury of left internal carotid artery, intracranial portion, not elsewhere classified with loss of consciousness of 31 minutes to 59 minutes, sequela | | | | | | | | | | | | | | | | | | | | | | | | |
| S06823A | | | | | Injury of left internal carotid artery, intracranial portion, not elsewhere classified with loss of consciousness of 1 hour to 5 hours 59 minutes, initial encounter | | | | | | | | | | | | | | | | | | | | | | | | |
| S06823D | | | | | Injury of left internal carotid artery, intracranial portion, not elsewhere classified with loss of consciousness of 1 hour to 5 hours 59 minutes, subsequent encounter | | | | | | | | | | | | | | | | | | | | | | | | |
| S06823S | | | | | Injury of left internal carotid artery, intracranial portion, not elsewhere classified with loss of consciousness of 1 hour to 5 hours 59 minutes, sequela | | | | | | | | | | | | | | | | | | | | | | | | |
| S06824A | | | | | Injury of left internal carotid artery, intracranial portion, not elsewhere classified with loss of consciousness of 6 hours to 24 hours, initial encounter | | | | | | | | | | | | | | | | | | | | | | | | |
| S06824D | | | | | Injury of left internal carotid artery, intracranial portion, not elsewhere classified with loss of consciousness of 6 hours to 24 hours, subsequent encounter | | | | | | | | | | | | | | | | | | | | | | | | |
| S06824S | | | | | Injury of left internal carotid artery, intracranial portion, not elsewhere classified with loss of consciousness of 6 hours to 24 hours, sequela | | | | | | | | | | | | | | | | | | | | | | | | |
| S06825A | | | | | Injury of left internal carotid artery, intracranial portion, not elsewhere classified with loss of consciousness greater than 24 hours with return to pre-existing conscious level, initial encounter | | | | | | | | | | | | | | | | | | | | | | | | |
| S06825D | | | | | Injury of left internal carotid artery, intracranial portion, not elsewhere classified with loss of consciousness greater than 24 hours with return to pre-existing conscious level, subsequent encounter | | | | | | | | | | | | | | | | | | | | | | | | |
| S06825S | | | | | Injury of left internal carotid artery, intracranial portion, not elsewhere classified with loss of consciousness greater than 24 hours with return to pre-existing conscious level, sequela | | | | | | | | | | | | | | | | | | | | | | | | |
| S06826A | | | | | Injury of left internal carotid artery, intracranial portion, not elsewhere classified with loss of consciousness greater than 24 hours without return to pre-existing conscious level with patient surviving, initial encounter | | | | | | | | | | | | | | | | | | | | | | | | |
| S06826D | | | | | Injury of left internal carotid artery, intracranial portion, not elsewhere classified with loss of consciousness greater than 24 hours without return to pre-existing conscious level with patient surviving, subsequent encounter | | | | | | | | | | | | | | | | | | | | | | | | |
| S06826S | | | | | Injury of left internal carotid artery, intracranial portion, not elsewhere classified with loss of consciousness greater than 24 hours without return to pre-existing conscious level with patient surviving, sequela | | | | | | | | | | | | | | | | | | | | | | | | |
| S06827A | | | | | Injury of left internal carotid artery, intracranial portion, not elsewhere classified with loss of consciousness of any duration with death due to brain injury prior to regaining consciousness, initial encounter | | | | | | | | | | | | | | | | | | | | | | | | |
| S06828A | | | | | Injury of left internal carotid artery, intracranial portion, not elsewhere classified with loss of consciousness of any duration with death due to other cause prior to regaining consciousness, initial encounter | | | | | | | | | | | | | | | | | | | | | | | | |
| S06829A | | | | | Injury of left internal carotid artery, intracranial portion, not elsewhere classified with loss of consciousness of unspecified duration, initial encounter | | | | | | | | | | | | | | | | | | | | | | | | |
| S06829D | | | | | Injury of left internal carotid artery, intracranial portion, not elsewhere classified with loss of consciousness of unspecified duration, subsequent encounter | | | | | | | | | | | | | | | | | | | | | | | | |
| S06829S | | | | | Injury of left internal carotid artery, intracranial portion, not elsewhere classified with loss of consciousness of unspecified duration, sequela | | | | | | | | | | | | | | | | | | | | | | | | |
| S06890A | | | | | Other specified intracranial injury without loss of consciousness, initial encounter | | | | | | | | | | | | | | | | | | | | | | | | |
| S06890D | | | | | Other specified intracranial injury without loss of consciousness, subsequent encounter | | | | | | | | | | | | | | | | | | | | | | | | |
| S06890S | | | | | Other specified intracranial injury without loss of consciousness, sequela | | | | | | | | | | | | | | | | | | | | | | | | |
| S06891A | | | | | Other specified intracranial injury with loss of consciousness of 30 minutes or less, initial encounter | | | | | | | | | | | | | | | | | | | | | | | | |
| S06891D | | | | | Other specified intracranial injury with loss of consciousness of 30 minutes or less, subsequent encounter | | | | | | | | | | | | | | | | | | | | | | | | |
| S06891S | | | | | Other specified intracranial injury with loss of consciousness of 30 minutes or less, sequela | | | | | | | | | | | | | | | | | | | | | | | | |
| W880XXS | | | | | Exposure to X-rays, sequela | | | | | | | | | | | | | | | | | | | | | | | | |
| S06892A | | | | | Other specified intracranial injury with loss of consciousness of 31 minutes to 59 minutes, initial encounter | | | | | | | | | | | | | | | | | | | | | | | | |
| S06892D | | | | | Other specified intracranial injury with loss of consciousness of 31 minutes to 59 minutes, subsequent encounter | | | | | | | | | | | | | | | | | | | | | | | | |
| S06892S | | | | | Other specified intracranial injury with loss of consciousness of 31 minutes to 59 minutes, sequela | | | | | | | | | | | | | | | | | | | | | | | | |
| S06893A | | | | | Other specified intracranial injury with loss of consciousness of 1 hour to 5 hours 59 minutes, initial encounter | | | | | | | | | | | | | | | | | | | | | | | | |
| S06893D | | | | | Other specified intracranial injury with loss of consciousness of 1 hour to 5 hours 59 minutes, subsequent encounter | | | | | | | | | | | | | | | | | | | | | | | | |
| S06893S | | | | | Other specified intracranial injury with loss of consciousness of 1 hour to 5 hours 59 minutes, sequela | | | | | | | | | | | | | | | | | | | | | | | | |
| S06894A | | | | | Other specified intracranial injury with loss of consciousness of 6 hours to 24 hours, initial encounter | | | | | | | | | | | | | | | | | | | | | | | | |
| S06894D | | | | | Other specified intracranial injury with loss of consciousness of 6 hours to 24 hours, subsequent encounter | | | | | | | | | | | | | | | | | | | | | | | | |
| S06894S | | | | | Other specified intracranial injury with loss of consciousness of 6 hours to 24 hours, sequela | | | | | | | | | | | | | | | | | | | | | | | | |
| S06895A | | | | | Other specified intracranial injury with loss of consciousness greater than 24 hours with return to pre-existing conscious level, initial encounter | | | | | | | | | | | | | | | | | | | | | | | | |
| S06895D | | | | | Other specified intracranial injury with loss of consciousness greater than 24 hours with return to pre-existing conscious level, subsequent encounter | | | | | | | | | | | | | | | | | | | | | | | | |
| S06895S | | | | | Other specified intracranial injury with loss of consciousness greater than 24 hours with return to pre-existing conscious level, sequela | | | | | | | | | | | | | | | | | | | | | | | | |
| S06896A | | | | | Other specified intracranial injury with loss of consciousness greater than 24 hours without return to pre-existing conscious level with patient surviving, initial encounter | | | | | | | | | | | | | | | | | | | | | | | | |
| S06896D | | | | | Other specified intracranial injury with loss of consciousness greater than 24 hours without return to pre-existing conscious level with patient surviving, subsequent encounter | | | | | | | | | | | | | | | | | | | | | | | | |
| S06896S | | | | | Other specified intracranial injury with loss of consciousness greater than 24 hours without return to pre-existing conscious level with patient surviving, sequela | | | | | | | | | | | | | | | | | | | | | | | | |
| S06897A | | | | | Other specified intracranial injury with loss of consciousness of any duration with death due to brain injury prior to regaining consciousness, initial encounter | | | | | | | | | | | | | | | | | | | | | | | | |
| S06898A | | | | | Other specified intracranial injury with loss of consciousness of any duration with death due to other cause prior to regaining consciousness, initial encounter | | | | | | | | | | | | | | | | | | | | | | | | |
| S06899A | | | | | Other specified intracranial injury with loss of consciousness of unspecified duration, initial encounter | | | | | | | | | | | | | | | | | | | | | | | | |
| S06899D | | | | | Other specified intracranial injury with loss of consciousness of unspecified duration, subsequent encounter | | | | | | | | | | | | | | | | | | | | | | | | |
| S06899S | | | | | Other specified intracranial injury with loss of consciousness of unspecified duration, sequela | | | | | | | | | | | | | | | | | | | | | | | | |
| S069X0A | | | | | Unspecified intracranial injury without loss of consciousness, initial encounter | | | | | | | | | | | | | | | | | | | | | | | | |
| S069X0D | | | | | Unspecified intracranial injury without loss of consciousness, subsequent encounter | | | | | | | | | | | | | | | | | | | | | | | | |
| S069X0S | | | | | Unspecified intracranial injury without loss of consciousness, sequela | | | | | | | | | | | | | | | | | | | | | | | | |
| S069X1A | | | | | Unspecified intracranial injury with loss of consciousness of 30 minutes or less, initial encounter | | | | | | | | | | | | | | | | | | | | | | | | |
| S069X1D | | | | | Unspecified intracranial injury with loss of consciousness of 30 minutes or less, subsequent encounter | | | | | | | | | | | | | | | | | | | | | | | | |
| S069X1S | | | | | Unspecified intracranial injury with loss of consciousness of 30 minutes or less, sequela | | | | | | | | | | | | | | | | | | | | | | | | |
| S069X2A | | | | | Unspecified intracranial injury with loss of consciousness of 31 minutes to 59 minutes, initial encounter | | | | | | | | | | | | | | | | | | | | | | | | |
| S069X2D | | | | | Unspecified intracranial injury with loss of consciousness of 31 minutes to 59 minutes, subsequent encounter | | | | | | | | | | | | | | | | | | | | | | | | |
| S069X2S | | | | | Unspecified intracranial injury with loss of consciousness of 31 minutes to 59 minutes, sequela | | | | | | | | | | | | | | | | | | | | | | | | |
| S069X3A | | | | | Unspecified intracranial injury with loss of consciousness of 1 hour to 5 hours 59 minutes, initial encounter | | | | | | | | | | | | | | | | | | | | | | | | |
| S069X3D | | | | | Unspecified intracranial injury with loss of consciousness of 1 hour to 5 hours 59 minutes, subsequent encounter | | | | | | | | | | | | | | | | | | | | | | | | |
| S069X3S | | | | | Unspecified intracranial injury with loss of consciousness of 1 hour to 5 hours 59 minutes, sequela | | | | | | | | | | | | | | | | | | | | | | | | |
| S069X4A | | | | | Unspecified intracranial injury with loss of consciousness of 6 hours to 24 hours, initial encounter | | | | | | | | | | | | | | | | | | | | | | | | |
| S069X4D | | | | | Unspecified intracranial injury with loss of consciousness of 6 hours to 24 hours, subsequent encounter | | | | | | | | | | | | | | | | | | | | | | | | |
| S069X4S | | | | | Unspecified intracranial injury with loss of consciousness of 6 hours to 24 hours, sequela | | | | | | | | | | | | | | | | | | | | | | | | |
| S069X5A | | | | | Unspecified intracranial injury with loss of consciousness greater than 24 hours with return to pre-existing conscious level, initial encounter | | | | | | | | | | | | | | | | | | | | | | | | |
| S069X5D | | | | | Unspecified intracranial injury with loss of consciousness greater than 24 hours with return to pre-existing conscious level, subsequent encounter | | | | | | | | | | | | | | | | | | | | | | | | |
| S069X5S | | | | | Unspecified intracranial injury with loss of consciousness greater than 24 hours with return to pre-existing conscious level, sequela | | | | | | | | | | | | | | | | | | | | | | | | |
| S069X6A | | | | | Unspecified intracranial injury with loss of consciousness greater than 24 hours without return to pre-existing conscious level with patient surviving, initial encounter | | | | | | | | | | | | | | | | | | | | | | | | |
| S069X6D | | | | | Unspecified intracranial injury with loss of consciousness greater than 24 hours without return to pre-existing conscious level with patient surviving, subsequent encounter | | | | | | | | | | | | | | | | | | | | | | | | |
| S069X6S | | | | | Unspecified intracranial injury with loss of consciousness greater than 24 hours without return to pre-existing conscious level with patient surviving, sequela | | | | | | | | | | | | | | | | | | | | | | | | |
| S069X7A | | | | | Unspecified intracranial injury with loss of consciousness of any duration with death due to brain injury prior to regaining consciousness, initial encounter | | | | | | | | | | | | | | | | | | | | | | | | |
| S069X8A | | | | | Unspecified intracranial injury with loss of consciousness of any duration with death due to other cause prior to regaining consciousness, initial encounter | | | | | | | | | | | | | | | | | | | | | | | | |
| S069X9A | | | | | Unspecified intracranial injury with loss of consciousness of unspecified duration, initial encounter | | | | | | | | | | | | | | | | | | | | | | | | |
| S069X9D | | | | | Unspecified intracranial injury with loss of consciousness of unspecified duration, subsequent encounter | | | | | | | | | | | | | | | | | | | | | | | | |
| S069X9S | | | | | Unspecified intracranial injury with loss of consciousness of unspecified duration, sequela | | | | | | | | | | | | | | | | | | | | | | | | |
| S070XXA | | | | | Crushing injury of face, initial encounter | | | | | | | | | | | | | | | | | | | | | | | | |
| S070XXD | | | | | Crushing injury of face, subsequent encounter | | | | | | | | | | | | | | | | | | | | | | | | |
| S070XXS | | | | | Crushing injury of face, sequela | | | | | | | | | | | | | | | | | | | | | | | | |
| S071XXA | | | | | Crushing injury of skull, initial encounter | | | | | | | | | | | | | | | | | | | | | | | | |
| S071XXD | | | | | Crushing injury of skull, subsequent encounter | | | | | | | | | | | | | | | | | | | | | | | | |
| S071XXS | | | | | Crushing injury of skull, sequela | | | | | | | | | | | | | | | | | | | | | | | | |
| S078XXA | | | | | Crushing injury of other parts of head, initial encounter | | | | | | | | | | | | | | | | | | | | | | | | |
| S079XXD | | | | | Crushing injury of head, part unspecified, subsequent encounter | | | | | | | | | | | | | | | | | | | | | | | | |
| S079XXS | | | | | Crushing injury of head, part unspecified, sequela | | | | | | | | | | | | | | | | | | | | | | | | |
| S080XXA | | | | | Avulsion of scalp, initial encounter | | | | | | | | | | | | | | | | | | | | | | | | |
| S080XXD | | | | | Avulsion of scalp, subsequent encounter | | | | | | | | | | | | | | | | | | | | | | | | |
| S080XXS | | | | | Avulsion of scalp, sequela | | | | | | | | | | | | | | | | | | | | | | | | |
| S08111A | | | | | Complete traumatic amputation of right ear, initial encounter | | | | | | | | | | | | | | | | | | | | | | | | |
| S08111D | | | | | Complete traumatic amputation of right ear, subsequent encounter | | | | | | | | | | | | | | | | | | | | | | | | |
| S08111S | | | | | Complete traumatic amputation of right ear, sequela | | | | | | | | | | | | | | | | | | | | | | | | |
| S08112A | | | | | Complete traumatic amputation of left ear, initial encounter | | | | | | | | | | | | | | | | | | | | | | | | |
| S08112D | | | | | Complete traumatic amputation of left ear, subsequent encounter | | | | | | | | | | | | | | | | | | | | | | | | |
| S08112S | | | | | Complete traumatic amputation of left ear, sequela | | | | | | | | | | | | | | | | | | | | | | | | |
| S08119A | | | | | Complete traumatic amputation of unspecified ear, initial encounter | | | | | | | | | | | | | | | | | | | | | | | | |
| S08119D | | | | | Complete traumatic amputation of unspecified ear, subsequent encounter | | | | | | | | | | | | | | | | | | | | | | | | |
| S08119S | | | | | Complete traumatic amputation of unspecified ear, sequela | | | | | | | | | | | | | | | | | | | | | | | | |
| S08121A | | | | | Partial traumatic amputation of right ear, initial encounter | | | | | | | | | | | | | | | | | | | | | | | | |
| S08121D | | | | | Partial traumatic amputation of right ear, subsequent encounter | | | | | | | | | | | | | | | | | | | | | | | | |
| S08121S | | | | | Partial traumatic amputation of right ear, sequela | | | | | | | | | | | | | | | | | | | | | | | | |
| S08122A | | | | | Partial traumatic amputation of left ear, initial encounter | | | | | | | | | | | | | | | | | | | | | | | | |
| S08122D | | | | | Partial traumatic amputation of left ear, subsequent encounter | | | | | | | | | | | | | | | | | | | | | | | | |
| S08122S | | | | | Partial traumatic amputation of left ear, sequela | | | | | | | | | | | | | | | | | | | | | | | | |
| S08129A | | | | | Partial traumatic amputation of unspecified ear, initial encounter | | | | | | | | | | | | | | | | | | | | | | | | |
| S08129D | | | | | Partial traumatic amputation of unspecified ear, subsequent encounter | | | | | | | | | | | | | | | | | | | | | | | | |
| S08129S | | | | | Partial traumatic amputation of unspecified ear, sequela | | | | | | | | | | | | | | | | | | | | | | | | |
| S08811A | | | | | Complete traumatic amputation of nose, initial encounter | | | | | | | | | | | | | | | | | | | | | | | | |
| S08811D | | | | | Complete traumatic amputation of nose, subsequent encounter | | | | | | | | | | | | | | | | | | | | | | | | |
| S08811S | | | | | Complete traumatic amputation of nose, sequela | | | | | | | | | | | | | | | | | | | | | | | | |
| S08812A | | | | | Partial traumatic amputation of nose, initial encounter | | | | | | | | | | | | | | | | | | | | | | | | |
| S08812D | | | | | Partial traumatic amputation of nose, subsequent encounter | | | | | | | | | | | | | | | | | | | | | | | | |
| S08812S | | | | | Partial traumatic amputation of nose, sequela | | | | | | | | | | | | | | | | | | | | | | | | |
| S0889XA | | | | | Traumatic amputation of other parts of head, initial encounter | | | | | | | | | | | | | | | | | | | | | | | | |
| S0889XD | | | | | Traumatic amputation of other parts of head, subsequent encounter | | | | | | | | | | | | | | | | | | | | | | | | |
| S0889XS | | | | | Traumatic amputation of other parts of head, sequela | | | | | | | | | | | | | | | | | | | | | | | | |
| S090XXA | | | | | Injury of blood vessels of head, not elsewhere classified, initial encounter | | | | | | | | | | | | | | | | | | | | | | | | |
| S090XXD | | | | | Injury of blood vessels of head, not elsewhere classified, subsequent encounter | | | | | | | | | | | | | | | | | | | | | | | | |
| S090XXS | | | | | Injury of blood vessels of head, not elsewhere classified, sequela | | | | | | | | | | | | | | | | | | | | | | | | |
| S0280XA | | | | | Fracture of other specified skull and facial bones, unspecified side, initial encounter for closed fracture | | | | | | | | | | | | | | | | | | | | | | | | |
| S0280XB | | | | | Fracture of other specified skull and facial bones, unspecified side, initial encounter for open fracture | | | | | | | | | | | | | | | | | | | | | | | | |
| S0280XD | | | | | Fracture of other specified skull and facial bones, unspecified side, subsequent encounter for fracture with routine healing | | | | | | | | | | | | | | | | | | | | | | | | |
| S0280XG | | | | | Fracture of other specified skull and facial bones, unspecified side, subsequent encounter for fracture with delayed healing | | | | | | | | | | | | | | | | | | | | | | | | |
| S0280XK | | | | | Fracture of other specified skull and facial bones, unspecified side, subsequent encounter for fracture with nonunion | | | | | | | | | | | | | | | | | | | | | | | | |
| S0280XS | | | | | Fracture of other specified skull and facial bones, unspecified side, sequela | | | | | | | | | | | | | | | | | | | | | | | | |
| S0281XA | | | | | Fracture of other specified skull and facial bones, right side, initial encounter for closed fracture | | | | | | | | | | | | | | | | | | | | | | | | |
| S0281XB | | | | | Fracture of other specified skull and facial bones, right side, initial encounter for open fracture | | | | | | | | | | | | | | | | | | | | | | | | |
| S0291XA | | | | | Unspecified fracture of skull, initial encounter for closed fracture | | | | | | | | | | | | | | | | | | | | | | | | |
| S0291XB | | | | | Unspecified fracture of skull, initial encounter for open fracture | | | | | | | | | | | | | | | | | | | | | | | | |
| S0291XD | | | | | Unspecified fracture of skull, subsequent encounter for fracture with routine healing | | | | | | | | | | | | | | | | | | | | | | | | |
| S0291XG | | | | | Unspecified fracture of skull, subsequent encounter for fracture with delayed healing | | | | | | | | | | | | | | | | | | | | | | | | |
| S0291XK | | | | | Unspecified fracture of skull, subsequent encounter for fracture with nonunion | | | | | | | | | | | | | | | | | | | | | | | | |
| S0291XS | | | | | Unspecified fracture of skull, sequela | | | | | | | | | | | | | | | | | | | | | | | | |
| S060X1S | | | | | Concussion with loss of consciousness of 30 minutes or less, sequela | | | | | | | | | | | | | | | | | | | | | | | | |
| S060X9A | | | | | Concussion with loss of consciousness of unspecified duration, initial encounter | | | | | | | | | | | | | | | | | | | | | | | | |
| S060X9D | | | | | Concussion with loss of consciousness of unspecified duration, subsequent encounter | | | | | | | | | | | | | | | | | | | | | | | | |
| S060X9S | | | | | Concussion with loss of consciousness of unspecified duration, sequela | | | | | | | | | | | | | | | | | | | | | | | | |
| S061X0A | | | | | Traumatic cerebral edema without loss of consciousness, initial encounter | | | | | | | | | | | | | | | | | | | | | | | | |
| S061X0D | | | | | Traumatic cerebral edema without loss of consciousness, subsequent encounter | | | | | | | | | | | | | | | | | | | | | | | | |
| S061X0S | | | | | Traumatic cerebral edema without loss of consciousness, sequela | | | | | | | | | | | | | | | | | | | | | | | | |
| S061X1A | | | | | Traumatic cerebral edema with loss of consciousness of 30 minutes or less, initial encounter | | | | | | | | | | | | | | | | | | | | | | | | |
| S061X1D | | | | | Traumatic cerebral edema with loss of consciousness of 30 minutes or less, subsequent encounter | | | | | | | | | | | | | | | | | | | | | | | | |
| S061X1S | | | | | Traumatic cerebral edema with loss of consciousness of 30 minutes or less, sequela | | | | | | | | | | | | | | | | | | | | | | | | |
| S061X2A | | | | | Traumatic cerebral edema with loss of consciousness of 31 minutes to 59 minutes, initial encounter | | | | | | | | | | | | | | | | | | | | | | | | |
| S061X2D | | | | | Traumatic cerebral edema with loss of consciousness of 31 minutes to 59 minutes, subsequent encounter | | | | | | | | | | | | | | | | | | | | | | | | |
| S061X2S | | | | | Traumatic cerebral edema with loss of consciousness of 31 minutes to 59 minutes, sequela | | | | | | | | | | | | | | | | | | | | | | | | |
| S061X3A | | | | | Traumatic cerebral edema with loss of consciousness of 1 hour to 5 hours 59 minutes, initial encounter | | | | | | | | | | | | | | | | | | | | | | | | |
| S061X3D | | | | | Traumatic cerebral edema with loss of consciousness of 1 hour to 5 hours 59 minutes, subsequent encounter | | | | | | | | | | | | | | | | | | | | | | | | |
| S061X3S | | | | | Traumatic cerebral edema with loss of consciousness of 1 hour to 5 hours 59 minutes, sequela | | | | | | | | | | | | | | | | | | | | | | | | |
| S061X4A | | | | | Traumatic cerebral edema with loss of consciousness of 6 hours to 24 hours, initial encounter | | | | | | | | | | | | | | | | | | | | | | | | |
| S061X4D | | | | | Traumatic cerebral edema with loss of consciousness of 6 hours to 24 hours, subsequent encounter | | | | | | | | | | | | | | | | | | | | | | | | |
| S061X4S | | | | | Traumatic cerebral edema with loss of consciousness of 6 hours to 24 hours, sequela | | | | | | | | | | | | | | | | | | | | | | | | |
| S061X5A | | | | | Traumatic cerebral edema with loss of consciousness greater than 24 hours with return to pre-existing conscious level, initial encounter | | | | | | | | | | | | | | | | | | | | | | | | |
| S061X5D | | | | | Traumatic cerebral edema with loss of consciousness greater than 24 hours with return to pre-existing conscious level, subsequent encounter | | | | | | | | | | | | | | | | | | | | | | | | |
| S061X5S | | | | | Traumatic cerebral edema with loss of consciousness greater than 24 hours with return to pre-existing conscious level, sequela | | | | | | | | | | | | | | | | | | | | | | | | |
| S061X6A | | | | | Traumatic cerebral edema with loss of consciousness greater than 24 hours without return to pre-existing conscious level with patient surviving, initial encounter | | | | | | | | | | | | | | | | | | | | | | | | |
| S061X6D | | | | | Traumatic cerebral edema with loss of consciousness greater than 24 hours without return to pre-existing conscious level with patient surviving, subsequent encounter | | | | | | | | | | | | | | | | | | | | | | | | |
| S061X6S | | | | | Traumatic cerebral edema with loss of consciousness greater than 24 hours without return to pre-existing conscious level with patient surviving, sequela | | | | | | | | | | | | | | | | | | | | | | | | |
| S061X7A | | | | | Traumatic cerebral edema with loss of consciousness of any duration with death due to brain injury prior to regaining consciousness, initial encounter | | | | | | | | | | | | | | | | | | | | | | | | |
| S061X8A | | | | | Traumatic cerebral edema with loss of consciousness of any duration with death due to other cause prior to regaining consciousness, initial encounter | | | | | | | | | | | | | | | | | | | | | | | | |
| S061X9A | | | | | Traumatic cerebral edema with loss of consciousness of unspecified duration, initial encounter | | | | | | | | | | | | | | | | | | | | | | | | |
| S061X9D | | | | | Traumatic cerebral edema with loss of consciousness of unspecified duration, subsequent encounter | | | | | | | | | | | | | | | | | | | | | | | | |
| S061X9S | | | | | Traumatic cerebral edema with loss of consciousness of unspecified duration, sequela | | | | | | | | | | | | | | | | | | | | | | | | |
| S062X0A | | | | | Diffuse traumatic brain injury without loss of consciousness, initial encounter | | | | | | | | | | | | | | | | | | | | | | | | |
| S062X0D | | | | | Diffuse traumatic brain injury without loss of consciousness, subsequent encounter | | | | | | | | | | | | | | | | | | | | | | | | |
| S062X0S | | | | | Diffuse traumatic brain injury without loss of consciousness, sequela | | | | | | | | | | | | | | | | | | | | | | | | |
| S062X1A | | | | | Diffuse traumatic brain injury with loss of consciousness of 30 minutes or less, initial encounter | | | | | | | | | | | | | | | | | | | | | | | | |
| S062X1D | | | | | Diffuse traumatic brain injury with loss of consciousness of 30 minutes or less, subsequent encounter | | | | | | | | | | | | | | | | | | | | | | | | |
| S062X1S | | | | | Diffuse traumatic brain injury with loss of consciousness of 30 minutes or less, sequela | | | | | | | | | | | | | | | | | | | | | | | | |
| S062X2A | | | | | Diffuse traumatic brain injury with loss of consciousness of 31 minutes to 59 minutes, initial encounter | | | | | | | | | | | | | | | | | | | | | | | | |
| S062X2D | | | | | Diffuse traumatic brain injury with loss of consciousness of 31 minutes to 59 minutes, subsequent encounter | | | | | | | | | | | | | | | | | | | | | | | | |
| S062X2S | | | | | Diffuse traumatic brain injury with loss of consciousness of 31 minutes to 59 minutes, sequela | | | | | | | | | | | | | | | | | | | | | | | | |
| S062X3A | | | | | Diffuse traumatic brain injury with loss of consciousness of 1 hour to 5 hours 59 minutes, initial encounter | | | | | | | | | | | | | | | | | | | | | | | | |
| S062X3D | | | | | Diffuse traumatic brain injury with loss of consciousness of 1 hour to 5 hours 59 minutes, subsequent encounter | | | | | | | | | | | | | | | | | | | | | | | | |
| S062X3S | | | | | Diffuse traumatic brain injury with loss of consciousness of 1 hour to 5 hours 59 minutes, sequela | | | | | | | | | | | | | | | | | | | | | | | | |
| S062X4A | | | | | Diffuse traumatic brain injury with loss of consciousness of 6 hours to 24 hours, initial encounter | | | | | | | | | | | | | | | | | | | | | | | | |
| S062X4D | | | | | Diffuse traumatic brain injury with loss of consciousness of 6 hours to 24 hours, subsequent encounter | | | | | | | | | | | | | | | | | | | | | | | | |
| S062X4S | | | | | Diffuse traumatic brain injury with loss of consciousness of 6 hours to 24 hours, sequela | | | | | | | | | | | | | | | | | | | | | | | | |
| S062X5A | | | | | Diffuse traumatic brain injury with loss of consciousness greater than 24 hours with return to pre-existing conscious levels, initial encounter | | | | | | | | | | | | | | | | | | | | | | | | |
| S062X5D | | | | | Diffuse traumatic brain injury with loss of consciousness greater than 24 hours with return to pre-existing conscious levels, subsequent encounter | | | | | | | | | | | | | | | | | | | | | | | | |
| S062X5S | | | | | Diffuse traumatic brain injury with loss of consciousness greater than 24 hours with return to pre-existing conscious levels, sequela | | | | | | | | | | | | | | | | | | | | | | | | |
| S062X6A | | | | | Diffuse traumatic brain injury with loss of consciousness greater than 24 hours without return to pre-existing conscious level with patient surviving, initial encounter | | | | | | | | | | | | | | | | | | | | | | | | |
| S062X6D | | | | | Diffuse traumatic brain injury with loss of consciousness greater than 24 hours without return to pre-existing conscious level with patient surviving, subsequent encounter | | | | | | | | | | | | | | | | | | | | | | | | |
| S062X6S | | | | | Diffuse traumatic brain injury with loss of consciousness greater than 24 hours without return to pre-existing conscious level with patient surviving, sequela | | | | | | | | | | | | | | | | | | | | | | | | |
| S062X7A | | | | | Diffuse traumatic brain injury with loss of consciousness of any duration with death due to brain injury prior to regaining consciousness, initial encounter | | | | | | | | | | | | | | | | | | | | | | | | |
| S062X8A | | | | | Diffuse traumatic brain injury with loss of consciousness of any duration with death due to other cause prior to regaining consciousness, initial encounter | | | | | | | | | | | | | | | | | | | | | | | | |
| V1542 | | | | | History of emotional abuse | | | | | | | | | | | | | | | | | | | | | | | | |
| S062X9A | | | | | Diffuse traumatic brain injury with loss of consciousness of unspecified duration, initial encounter | | | | | | | | | | | | | | | | | | | | | | | | |
| S062X9D | | | | | Diffuse traumatic brain injury with loss of consciousness of unspecified duration, subsequent encounter | | | | | | | | | | | | | | | | | | | | | | | | |
| S062X9S | | | | | Diffuse traumatic brain injury with loss of consciousness of unspecified duration, sequela | | | | | | | | | | | | | | | | | | | | | | | | |
| S06300A | | | | | Unspecified focal traumatic brain injury without loss of consciousness, initial encounter | | | | | | | | | | | | | | | | | | | | | | | | |
| S06300D | | | | | Unspecified focal traumatic brain injury without loss of consciousness, subsequent encounter | | | | | | | | | | | | | | | | | | | | | | | | |
| S06300S | | | | | Unspecified focal traumatic brain injury without loss of consciousness, sequela | | | | | | | | | | | | | | | | | | | | | | | | |
| S06301A | | | | | Unspecified focal traumatic brain injury with loss of consciousness of 30 minutes or less, initial encounter | | | | | | | | | | | | | | | | | | | | | | | | |
| S06301D | | | | | Unspecified focal traumatic brain injury with loss of consciousness of 30 minutes or less, subsequent encounter | | | | | | | | | | | | | | | | | | | | | | | | |
| S06301S | | | | | Unspecified focal traumatic brain injury with loss of consciousness of 30 minutes or less, sequela | | | | | | | | | | | | | | | | | | | | | | | | |
| S06302A | | | | | Unspecified focal traumatic brain injury with loss of consciousness of 31 minutes to 59 minutes, initial encounter | | | | | | | | | | | | | | | | | | | | | | | | |
| S06302D | | | | | Unspecified focal traumatic brain injury with loss of consciousness of 31 minutes to 59 minutes, subsequent encounter | | | | | | | | | | | | | | | | | | | | | | | | |
| S06302S | | | | | Unspecified focal traumatic brain injury with loss of consciousness of 31 minutes to 59 minutes, sequela | | | | | | | | | | | | | | | | | | | | | | | | |
| S06303A | | | | | Unspecified focal traumatic brain injury with loss of consciousness of 1 hour to 5 hours 59 minutes, initial encounter | | | | | | | | | | | | | | | | | | | | | | | | |
| S06303D | | | | | Unspecified focal traumatic brain injury with loss of consciousness of 1 hour to 5 hours 59 minutes, subsequent encounter | | | | | | | | | | | | | | | | | | | | | | | | |
| S06303S | | | | | Unspecified focal traumatic brain injury with loss of consciousness of 1 hour to 5 hours 59 minutes, sequela | | | | | | | | | | | | | | | | | | | | | | | | |
| S06304A | | | | | Unspecified focal traumatic brain injury with loss of consciousness of 6 hours to 24 hours, initial encounter | | | | | | | | | | | | | | | | | | | | | | | | |
| S06304D | | | | | Unspecified focal traumatic brain injury with loss of consciousness of 6 hours to 24 hours, subsequent encounter | | | | | | | | | | | | | | | | | | | | | | | | |
| S06304S | | | | | Unspecified focal traumatic brain injury with loss of consciousness of 6 hours to 24 hours, sequela | | | | | | | | | | | | | | | | | | | | | | | | |
| S06305A | | | | | Unspecified focal traumatic brain injury with loss of consciousness greater than 24 hours with return to pre-existing conscious level, initial encounter | | | | | | | | | | | | | | | | | | | | | | | | |
| S06305D | | | | | Unspecified focal traumatic brain injury with loss of consciousness greater than 24 hours with return to pre-existing conscious level, subsequent encounter | | | | | | | | | | | | | | | | | | | | | | | | |
| S06305S | | | | | Unspecified focal traumatic brain injury with loss of consciousness greater than 24 hours with return to pre-existing conscious level, sequela | | | | | | | | | | | | | | | | | | | | | | | | |
| S06306A | | | | | Unspecified focal traumatic brain injury with loss of consciousness greater than 24 hours without return to pre-existing conscious level with patient surviving, initial encounter | | | | | | | | | | | | | | | | | | | | | | | | |
| S06306D | | | | | Unspecified focal traumatic brain injury with loss of consciousness greater than 24 hours without return to pre-existing conscious level with patient surviving, subsequent encounter | | | | | | | | | | | | | | | | | | | | | | | | |
| S06306S | | | | | Unspecified focal traumatic brain injury with loss of consciousness greater than 24 hours without return to pre-existing conscious level with patient surviving, sequela | | | | | | | | | | | | | | | | | | | | | | | | |
| S06307A | | | | | Unspecified focal traumatic brain injury with loss of consciousness of any duration with death due to brain injury prior to regaining consciousness, initial encounter | | | | | | | | | | | | | | | | | | | | | | | | |
| S06308A | | | | | Unspecified focal traumatic brain injury with loss of consciousness of any duration with death due to other cause prior to regaining consciousness, initial encounter | | | | | | | | | | | | | | | | | | | | | | | | |
| S06309A | | | | | Unspecified focal traumatic brain injury with loss of consciousness of unspecified duration, initial encounter | | | | | | | | | | | | | | | | | | | | | | | | |
| S06309D | | | | | Unspecified focal traumatic brain injury with loss of consciousness of unspecified duration, subsequent encounter | | | | | | | | | | | | | | | | | | | | | | | | |
| S06309S | | | | | Unspecified focal traumatic brain injury with loss of consciousness of unspecified duration, sequela | | | | | | | | | | | | | | | | | | | | | | | | |
| S06310A | | | | | Contusion and laceration of right cerebrum without loss of consciousness, initial encounter | | | | | | | | | | | | | | | | | | | | | | | | |
| S06310D | | | | | Contusion and laceration of right cerebrum without loss of consciousness, subsequent encounter | | | | | | | | | | | | | | | | | | | | | | | | |
| S06310S | | | | | Contusion and laceration of right cerebrum without loss of consciousness, sequela | | | | | | | | | | | | | | | | | | | | | | | | |
| S06311A | | | | | Contusion and laceration of right cerebrum with loss of consciousness of 30 minutes or less, initial encounter | | | | | | | | | | | | | | | | | | | | | | | | |
| S06311D | | | | | Contusion and laceration of right cerebrum with loss of consciousness of 30 minutes or less, subsequent encounter | | | | | | | | | | | | | | | | | | | | | | | | |
| S06311S | | | | | Contusion and laceration of right cerebrum with loss of consciousness of 30 minutes or less, sequela | | | | | | | | | | | | | | | | | | | | | | | | |
| S06312A | | | | | Contusion and laceration of right cerebrum with loss of consciousness of 31 minutes to 59 minutes, initial encounter | | | | | | | | | | | | | | | | | | | | | | | | |
| S06312D | | | | | Contusion and laceration of right cerebrum with loss of consciousness of 31 minutes to 59 minutes, subsequent encounter | | | | | | | | | | | | | | | | | | | | | | | | |
| S06312S | | | | | Contusion and laceration of right cerebrum with loss of consciousness of 31 minutes to 59 minutes, sequela | | | | | | | | | | | | | | | | | | | | | | | | |
| S06313A | | | | | Contusion and laceration of right cerebrum with loss of consciousness of 1 hour to 5 hours 59 minutes, initial encounter | | | | | | | | | | | | | | | | | | | | | | | | |
| S06313D | | | | | Contusion and laceration of right cerebrum with loss of consciousness of 1 hour to 5 hours 59 minutes, subsequent encounter | | | | | | | | | | | | | | | | | | | | | | | | |
| S06313S | | | | | Contusion and laceration of right cerebrum with loss of consciousness of 1 hour to 5 hours 59 minutes, sequela | | | | | | | | | | | | | | | | | | | | | | | | |
| S06314A | | | | | Contusion and laceration of right cerebrum with loss of consciousness of 6 hours to 24 hours, initial encounter | | | | | | | | | | | | | | | | | | | | | | | | |
| S06314D | | | | | Contusion and laceration of right cerebrum with loss of consciousness of 6 hours to 24 hours, subsequent encounter | | | | | | | | | | | | | | | | | | | | | | | | |
| S06314S | | | | | Contusion and laceration of right cerebrum with loss of consciousness of 6 hours to 24 hours, sequela | | | | | | | | | | | | | | | | | | | | | | | | |
| S06315A | | | | | Contusion and laceration of right cerebrum with loss of consciousness greater than 24 hours with return to pre-existing conscious level, initial encounter | | | | | | | | | | | | | | | | | | | | | | | | |
| S06315D | | | | | Contusion and laceration of right cerebrum with loss of consciousness greater than 24 hours with return to pre-existing conscious level, subsequent encounter | | | | | | | | | | | | | | | | | | | | | | | | |
| S06315S | | | | | Contusion and laceration of right cerebrum with loss of consciousness greater than 24 hours with return to pre-existing conscious level, sequela | | | | | | | | | | | | | | | | | | | | | | | | |
| S06316A | | | | | Contusion and laceration of right cerebrum with loss of consciousness greater than 24 hours without return to pre-existing conscious level with patient surviving, initial encounter | | | | | | | | | | | | | | | | | | | | | | | | |
| S06316D | | | | | Contusion and laceration of right cerebrum with loss of consciousness greater than 24 hours without return to pre-existing conscious level with patient surviving, subsequent encounter | | | | | | | | | | | | | | | | | | | | | | | | |
| S078XXD | | | | | Crushing injury of other parts of head, subsequent encounter | | | | | | | | | | | | | | | | | | | | | | | | |
| S06316S | | | | | Contusion and laceration of right cerebrum with loss of consciousness greater than 24 hours without return to pre-existing conscious level with patient surviving, sequela | | | | | | | | | | | | | | | | | | | | | | | | |
| S06317A | | | | | Contusion and laceration of right cerebrum with loss of consciousness of any duration with death due to brain injury prior to regaining consciousness, initial encounter | | | | | | | | | | | | | | | | | | | | | | | | |
| S06318A | | | | | Contusion and laceration of right cerebrum with loss of consciousness of any duration with death due to other cause prior to regaining consciousness, initial encounter | | | | | | | | | | | | | | | | | | | | | | | | |
| S06319A | | | | | Contusion and laceration of right cerebrum with loss of consciousness of unspecified duration, initial encounter | | | | | | | | | | | | | | | | | | | | | | | | |
| S06319D | | | | | Contusion and laceration of right cerebrum with loss of consciousness of unspecified duration, subsequent encounter | | | | | | | | | | | | | | | | | | | | | | | | |
| S06319S | | | | | Contusion and laceration of right cerebrum with loss of consciousness of unspecified duration, sequela | | | | | | | | | | | | | | | | | | | | | | | | |
| S06320A | | | | | Contusion and laceration of left cerebrum without loss of consciousness, initial encounter | | | | | | | | | | | | | | | | | | | | | | | | |
| S06320D | | | | | Contusion and laceration of left cerebrum without loss of consciousness, subsequent encounter | | | | | | | | | | | | | | | | | | | | | | | | |
| S06320S | | | | | Contusion and laceration of left cerebrum without loss of consciousness, sequela | | | | | | | | | | | | | | | | | | | | | | | | |
| S06321A | | | | | Contusion and laceration of left cerebrum with loss of consciousness of 30 minutes or less, initial encounter | | | | | | | | | | | | | | | | | | | | | | | | |
| S06321D | | | | | Contusion and laceration of left cerebrum with loss of consciousness of 30 minutes or less, subsequent encounter | | | | | | | | | | | | | | | | | | | | | | | | |
| S06321S | | | | | Contusion and laceration of left cerebrum with loss of consciousness of 30 minutes or less, sequela | | | | | | | | | | | | | | | | | | | | | | | | |
| S06322A | | | | | Contusion and laceration of left cerebrum with loss of consciousness of 31 minutes to 59 minutes, initial encounter | | | | | | | | | | | | | | | | | | | | | | | | |
| S06322D | | | | | Contusion and laceration of left cerebrum with loss of consciousness of 31 minutes to 59 minutes, subsequent encounter | | | | | | | | | | | | | | | | | | | | | | | | |
| S06322S | | | | | Contusion and laceration of left cerebrum with loss of consciousness of 31 minutes to 59 minutes, sequela | | | | | | | | | | | | | | | | | | | | | | | | |
| S06323A | | | | | Contusion and laceration of left cerebrum with loss of consciousness of 1 hour to 5 hours 59 minutes, initial encounter | | | | | | | | | | | | | | | | | | | | | | | | |
| S06323D | | | | | Contusion and laceration of left cerebrum with loss of consciousness of 1 hour to 5 hours 59 minutes, subsequent encounter | | | | | | | | | | | | | | | | | | | | | | | | |
| S06323S | | | | | Contusion and laceration of left cerebrum with loss of consciousness of 1 hour to 5 hours 59 minutes, sequela | | | | | | | | | | | | | | | | | | | | | | | | |
| S06324A | | | | | Contusion and laceration of left cerebrum with loss of consciousness of 6 hours to 24 hours, initial encounter | | | | | | | | | | | | | | | | | | | | | | | | |
| S06324D | | | | | Contusion and laceration of left cerebrum with loss of consciousness of 6 hours to 24 hours, subsequent encounter | | | | | | | | | | | | | | | | | | | | | | | | |
| S06324S | | | | | Contusion and laceration of left cerebrum with loss of consciousness of 6 hours to 24 hours, sequela | | | | | | | | | | | | | | | | | | | | | | | | |
| S06325A | | | | | Contusion and laceration of left cerebrum with loss of consciousness greater than 24 hours with return to pre-existing conscious level, initial encounter | | | | | | | | | | | | | | | | | | | | | | | | |
| S06325D | | | | | Contusion and laceration of left cerebrum with loss of consciousness greater than 24 hours with return to pre-existing conscious level, subsequent encounter | | | | | | | | | | | | | | | | | | | | | | | | |
| S06325S | | | | | Contusion and laceration of left cerebrum with loss of consciousness greater than 24 hours with return to pre-existing conscious level, sequela | | | | | | | | | | | | | | | | | | | | | | | | |
| S06326A | | | | | Contusion and laceration of left cerebrum with loss of consciousness greater than 24 hours without return to pre-existing conscious level with patient surviving, initial encounter | | | | | | | | | | | | | | | | | | | | | | | | |
| S06326D | | | | | Contusion and laceration of left cerebrum with loss of consciousness greater than 24 hours without return to pre-existing conscious level with patient surviving, subsequent encounter | | | | | | | | | | | | | | | | | | | | | | | | |
| S06326S | | | | | Contusion and laceration of left cerebrum with loss of consciousness greater than 24 hours without return to pre-existing conscious level with patient surviving, sequela | | | | | | | | | | | | | | | | | | | | | | | | |
| S06327A | | | | | Contusion and laceration of left cerebrum with loss of consciousness of any duration with death due to brain injury prior to regaining consciousness, initial encounter | | | | | | | | | | | | | | | | | | | | | | | | |
| S06328A | | | | | Contusion and laceration of left cerebrum with loss of consciousness of any duration with death due to other cause prior to regaining consciousness, initial encounter | | | | | | | | | | | | | | | | | | | | | | | | |
| S06329A | | | | | Contusion and laceration of left cerebrum with loss of consciousness of unspecified duration, initial encounter | | | | | | | | | | | | | | | | | | | | | | | | |
| S06329D | | | | | Contusion and laceration of left cerebrum with loss of consciousness of unspecified duration, subsequent encounter | | | | | | | | | | | | | | | | | | | | | | | | |
| S06329S | | | | | Contusion and laceration of left cerebrum with loss of consciousness of unspecified duration, sequela | | | | | | | | | | | | | | | | | | | | | | | | |
| S06330A | | | | | Contusion and laceration of cerebrum, unspecified, without loss of consciousness, initial encounter | | | | | | | | | | | | | | | | | | | | | | | | |
| S06330D | | | | | Contusion and laceration of cerebrum, unspecified, without loss of consciousness, subsequent encounter | | | | | | | | | | | | | | | | | | | | | | | | |
| S06330S | | | | | Contusion and laceration of cerebrum, unspecified, without loss of consciousness, sequela | | | | | | | | | | | | | | | | | | | | | | | | |
| S06331A | | | | | Contusion and laceration of cerebrum, unspecified, with loss of consciousness of 30 minutes or less, initial encounter | | | | | | | | | | | | | | | | | | | | | | | | |
| S06331D | | | | | Contusion and laceration of cerebrum, unspecified, with loss of consciousness of 30 minutes or less, subsequent encounter | | | | | | | | | | | | | | | | | | | | | | | | |
| S06331S | | | | | Contusion and laceration of cerebrum, unspecified, with loss of consciousness of 30 minutes or less, sequela | | | | | | | | | | | | | | | | | | | | | | | | |
| S06332A | | | | | Contusion and laceration of cerebrum, unspecified, with loss of consciousness of 31 minutes to 59 minutes, initial encounter | | | | | | | | | | | | | | | | | | | | | | | | |
| S06332D | | | | | Contusion and laceration of cerebrum, unspecified, with loss of consciousness of 31 minutes to 59 minutes, subsequent encounter | | | | | | | | | | | | | | | | | | | | | | | | |
| S06332S | | | | | Contusion and laceration of cerebrum, unspecified, with loss of consciousness of 31 minutes to 59 minutes, sequela | | | | | | | | | | | | | | | | | | | | | | | | |
| S06333A | | | | | Contusion and laceration of cerebrum, unspecified, with loss of consciousness of 1 hour to 5 hours 59 minutes, initial encounter | | | | | | | | | | | | | | | | | | | | | | | | |
| S06333D | | | | | Contusion and laceration of cerebrum, unspecified, with loss of consciousness of 1 hour to 5 hours 59 minutes, subsequent encounter | | | | | | | | | | | | | | | | | | | | | | | | |
| S06334A | | | | | Contusion and laceration of cerebrum, unspecified, with loss of consciousness of 6 hours to 24 hours, initial encounter | | | | | | | | | | | | | | | | | | | | | | | | |
| S06334D | | | | | Contusion and laceration of cerebrum, unspecified, with loss of consciousness of 6 hours to 24 hours, subsequent encounter | | | | | | | | | | | | | | | | | | | | | | | | |
| S06334S | | | | | Contusion and laceration of cerebrum, unspecified, with loss of consciousness of 6 hours to 24 hours, sequela | | | | | | | | | | | | | | | | | | | | | | | | |
| S06335A | | | | | Contusion and laceration of cerebrum, unspecified, with loss of consciousness greater than 24 hours with return to pre-existing conscious level, initial encounter | | | | | | | | | | | | | | | | | | | | | | | | |
| S06335D | | | | | Contusion and laceration of cerebrum, unspecified, with loss of consciousness greater than 24 hours with return to pre-existing conscious level, subsequent encounter | | | | | | | | | | | | | | | | | | | | | | | | |
| S06335S | | | | | Contusion and laceration of cerebrum, unspecified, with loss of consciousness greater than 24 hours with return to pre-existing conscious level, sequela | | | | | | | | | | | | | | | | | | | | | | | | |
| S06336A | | | | | Contusion and laceration of cerebrum, unspecified, with loss of consciousness greater than 24 hours without return to pre-existing conscious level with patient surviving, initial encounter | | | | | | | | | | | | | | | | | | | | | | | | |
| S06336D | | | | | Contusion and laceration of cerebrum, unspecified, with loss of consciousness greater than 24 hours without return to pre-existing conscious level with patient surviving, subsequent encounter | | | | | | | | | | | | | | | | | | | | | | | | |
| S06336S | | | | | Contusion and laceration of cerebrum, unspecified, with loss of consciousness greater than 24 hours without return to pre-existing conscious level with patient surviving, sequela | | | | | | | | | | | | | | | | | | | | | | | | |
| S06337A | | | | | Contusion and laceration of cerebrum, unspecified, with loss of consciousness of any duration with death due to brain injury prior to regaining consciousness, initial encounter | | | | | | | | | | | | | | | | | | | | | | | | |
| S06338A | | | | | Contusion and laceration of cerebrum, unspecified, with loss of consciousness of any duration with death due to other cause prior to regaining consciousness, initial encounter | | | | | | | | | | | | | | | | | | | | | | | | |
| S06339A | | | | | Contusion and laceration of cerebrum, unspecified, with loss of consciousness of unspecified duration, initial encounter | | | | | | | | | | | | | | | | | | | | | | | | |
| S06339D | | | | | Contusion and laceration of cerebrum, unspecified, with loss of consciousness of unspecified duration, subsequent encounter | | | | | | | | | | | | | | | | | | | | | | | | |
| S06339S | | | | | Contusion and laceration of cerebrum, unspecified, with loss of consciousness of unspecified duration, sequela | | | | | | | | | | | | | | | | | | | | | | | | |
| S06340A | | | | | Traumatic hemorrhage of right cerebrum without loss of consciousness, initial encounter | | | | | | | | | | | | | | | | | | | | | | | | |
| S06340D | | | | | Traumatic hemorrhage of right cerebrum without loss of consciousness, subsequent encounter | | | | | | | | | | | | | | | | | | | | | | | | |
| S06340S | | | | | Traumatic hemorrhage of right cerebrum without loss of consciousness, sequela | | | | | | | | | | | | | | | | | | | | | | | | |
| S06341A | | | | | Traumatic hemorrhage of right cerebrum with loss of consciousness of 30 minutes or less, initial encounter | | | | | | | | | | | | | | | | | | | | | | | | |
| S06341D | | | | | Traumatic hemorrhage of right cerebrum with loss of consciousness of 30 minutes or less, subsequent encounter | | | | | | | | | | | | | | | | | | | | | | | | |
| S06341S | | | | | Traumatic hemorrhage of right cerebrum with loss of consciousness of 30 minutes or less, sequela | | | | | | | | | | | | | | | | | | | | | | | | |
| S06342A | | | | | Traumatic hemorrhage of right cerebrum with loss of consciousness of 31 minutes to 59 minutes, initial encounter | | | | | | | | | | | | | | | | | | | | | | | | |
| S06342D | | | | | Traumatic hemorrhage of right cerebrum with loss of consciousness of 31 minutes to 59 minutes, subsequent encounter | | | | | | | | | | | | | | | | | | | | | | | | |
| S06342S | | | | | Traumatic hemorrhage of right cerebrum with loss of consciousness of 31 minutes to 59 minutes, sequela | | | | | | | | | | | | | | | | | | | | | | | | |
| S06343A | | | | | Traumatic hemorrhage of right cerebrum with loss of consciousness of 1 hours to 5 hours 59 minutes, initial encounter | | | | | | | | | | | | | | | | | | | | | | | | |
| S06343D | | | | | Traumatic hemorrhage of right cerebrum with loss of consciousness of 1 hours to 5 hours 59 minutes, subsequent encounter | | | | | | | | | | | | | | | | | | | | | | | | |
| S06343S | | | | | Traumatic hemorrhage of right cerebrum with loss of consciousness of 1 hours to 5 hours 59 minutes, sequela | | | | | | | | | | | | | | | | | | | | | | | | |
| S06344A | | | | | Traumatic hemorrhage of right cerebrum with loss of consciousness of 6 hours to 24 hours, initial encounter | | | | | | | | | | | | | | | | | | | | | | | | |
| S06344D | | | | | Traumatic hemorrhage of right cerebrum with loss of consciousness of 6 hours to 24 hours, subsequent encounter | | | | | | | | | | | | | | | | | | | | | | | | |
| S06344S | | | | | Traumatic hemorrhage of right cerebrum with loss of consciousness of 6 hours to 24 hours, sequela | | | | | | | | | | | | | | | | | | | | | | | | |
| S06345A | | | | | Traumatic hemorrhage of right cerebrum with loss of consciousness greater than 24 hours with return to pre-existing conscious level, initial encounter | | | | | | | | | | | | | | | | | | | | | | | | |
| S06345D | | | | | Traumatic hemorrhage of right cerebrum with loss of consciousness greater than 24 hours with return to pre-existing conscious level, subsequent encounter | | | | | | | | | | | | | | | | | | | | | | | | |
| S06345S | | | | | Traumatic hemorrhage of right cerebrum with loss of consciousness greater than 24 hours with return to pre-existing conscious level, sequela | | | | | | | | | | | | | | | | | | | | | | | | |
| S06346A | | | | | Traumatic hemorrhage of right cerebrum with loss of consciousness greater than 24 hours without return to pre-existing conscious level with patient surviving, initial encounter | | | | | | | | | | | | | | | | | | | | | | | | |
| S06346D | | | | | Traumatic hemorrhage of right cerebrum with loss of consciousness greater than 24 hours without return to pre-existing conscious level with patient surviving, subsequent encounter | | | | | | | | | | | | | | | | | | | | | | | | |
| S06346S | | | | | Traumatic hemorrhage of right cerebrum with loss of consciousness greater than 24 hours without return to pre-existing conscious level with patient surviving, sequela | | | | | | | | | | | | | | | | | | | | | | | | |
| S06347A | | | | | Traumatic hemorrhage of right cerebrum with loss of consciousness of any duration with death due to brain injury prior to regaining consciousness, initial encounter | | | | | | | | | | | | | | | | | | | | | | | | |
| S06348A | | | | | Traumatic hemorrhage of right cerebrum with loss of consciousness of any duration with death due to other cause prior to regaining consciousness, initial encounter | | | | | | | | | | | | | | | | | | | | | | | | |
| S06349A | | | | | Traumatic hemorrhage of right cerebrum with loss of consciousness of unspecified duration, initial encounter | | | | | | | | | | | | | | | | | | | | | | | | |
| S06349D | | | | | Traumatic hemorrhage of right cerebrum with loss of consciousness of unspecified duration, subsequent encounter | | | | | | | | | | | | | | | | | | | | | | | | |
| S06349S | | | | | Traumatic hemorrhage of right cerebrum with loss of consciousness of unspecified duration, sequela | | | | | | | | | | | | | | | | | | | | | | | | |
| S06350A | | | | | Traumatic hemorrhage of left cerebrum without loss of consciousness, initial encounter | | | | | | | | | | | | | | | | | | | | | | | | |
| S06350D | | | | | Traumatic hemorrhage of left cerebrum without loss of consciousness, subsequent encounter | | | | | | | | | | | | | | | | | | | | | | | | |
| S06350S | | | | | Traumatic hemorrhage of left cerebrum without loss of consciousness, sequela | | | | | | | | | | | | | | | | | | | | | | | | |
| S06351A | | | | | Traumatic hemorrhage of left cerebrum with loss of consciousness of 30 minutes or less, initial encounter | | | | | | | | | | | | | | | | | | | | | | | | |
| S06351D | | | | | Traumatic hemorrhage of left cerebrum with loss of consciousness of 30 minutes or less, subsequent encounter | | | | | | | | | | | | | | | | | | | | | | | | |
| S06351S | | | | | Traumatic hemorrhage of left cerebrum with loss of consciousness of 30 minutes or less, sequela | | | | | | | | | | | | | | | | | | | | | | | | |
| S06352A | | | | | Traumatic hemorrhage of left cerebrum with loss of consciousness of 31 minutes to 59 minutes, initial encounter | | | | | | | | | | | | | | | | | | | | | | | | |
| S06352D | | | | | Traumatic hemorrhage of left cerebrum with loss of consciousness of 31 minutes to 59 minutes, subsequent encounter | | | | | | | | | | | | | | | | | | | | | | | | |
| S06352S | | | | | Traumatic hemorrhage of left cerebrum with loss of consciousness of 31 minutes to 59 minutes, sequela | | | | | | | | | | | | | | | | | | | | | | | | |
| S06353A | | | | | Traumatic hemorrhage of left cerebrum with loss of consciousness of 1 hours to 5 hours 59 minutes, initial encounter | | | | | | | | | | | | | | | | | | | | | | | | |
| S06353D | | | | | Traumatic hemorrhage of left cerebrum with loss of consciousness of 1 hours to 5 hours 59 minutes, subsequent encounter | | | | | | | | | | | | | | | | | | | | | | | | |
| S06353S | | | | | Traumatic hemorrhage of left cerebrum with loss of consciousness of 1 hours to 5 hours 59 minutes, sequela | | | | | | | | | | | | | | | | | | | | | | | | |
| V425 | | | | | Cornea replaced by transplant | | | | | | | | | | | | | | | | | | | | | | | | |
| S06354A | | | | | Traumatic hemorrhage of left cerebrum with loss of consciousness of 6 hours to 24 hours, initial encounter | | | | | | | | | | | | | | | | | | | | | | | | |
| S06354D | | | | | Traumatic hemorrhage of left cerebrum with loss of consciousness of 6 hours to 24 hours, subsequent encounter | | | | | | | | | | | | | | | | | | | | | | | | |
| S06354S | | | | | Traumatic hemorrhage of left cerebrum with loss of consciousness of 6 hours to 24 hours, sequela | | | | | | | | | | | | | | | | | | | | | | | | |
| S06355A | | | | | Traumatic hemorrhage of left cerebrum with loss of consciousness greater than 24 hours with return to pre-existing conscious level, initial encounter | | | | | | | | | | | | | | | | | | | | | | | | |
| S06355D | | | | | Traumatic hemorrhage of left cerebrum with loss of consciousness greater than 24 hours with return to pre-existing conscious level, subsequent encounter | | | | | | | | | | | | | | | | | | | | | | | | |
| S06355S | | | | | Traumatic hemorrhage of left cerebrum with loss of consciousness greater than 24 hours with return to pre-existing conscious level, sequela | | | | | | | | | | | | | | | | | | | | | | | | |
| S06356A | | | | | Traumatic hemorrhage of left cerebrum with loss of consciousness greater than 24 hours without return to pre-existing conscious level with patient surviving, initial encounter | | | | | | | | | | | | | | | | | | | | | | | | |
| S06356D | | | | | Traumatic hemorrhage of left cerebrum with loss of consciousness greater than 24 hours without return to pre-existing conscious level with patient surviving, subsequent encounter | | | | | | | | | | | | | | | | | | | | | | | | |
| S06356S | | | | | Traumatic hemorrhage of left cerebrum with loss of consciousness greater than 24 hours without return to pre-existing conscious level with patient surviving, sequela | | | | | | | | | | | | | | | | | | | | | | | | |
| S06357A | | | | | Traumatic hemorrhage of left cerebrum with loss of consciousness of any duration with death due to brain injury prior to regaining consciousness, initial encounter | | | | | | | | | | | | | | | | | | | | | | | | |
| S06358A | | | | | Traumatic hemorrhage of left cerebrum with loss of consciousness of any duration with death due to other cause prior to regaining consciousness, initial encounter | | | | | | | | | | | | | | | | | | | | | | | | |
| S06359A | | | | | Traumatic hemorrhage of left cerebrum with loss of consciousness of unspecified duration, initial encounter | | | | | | | | | | | | | | | | | | | | | | | | |
| S06359D | | | | | Traumatic hemorrhage of left cerebrum with loss of consciousness of unspecified duration, subsequent encounter | | | | | | | | | | | | | | | | | | | | | | | | |
| S06359S | | | | | Traumatic hemorrhage of left cerebrum with loss of consciousness of unspecified duration, sequela | | | | | | | | | | | | | | | | | | | | | | | | |
| S06360A | | | | | Traumatic hemorrhage of cerebrum, unspecified, without loss of consciousness, initial encounter | | | | | | | | | | | | | | | | | | | | | | | | |
| S06360D | | | | | Traumatic hemorrhage of cerebrum, unspecified, without loss of consciousness, subsequent encounter | | | | | | | | | | | | | | | | | | | | | | | | |
| S06360S | | | | | Traumatic hemorrhage of cerebrum, unspecified, without loss of consciousness, sequela | | | | | | | | | | | | | | | | | | | | | | | | |
| S06361A | | | | | Traumatic hemorrhage of cerebrum, unspecified, with loss of consciousness of 30 minutes or less, initial encounter | | | | | | | | | | | | | | | | | | | | | | | | |
| S06361D | | | | | Traumatic hemorrhage of cerebrum, unspecified, with loss of consciousness of 30 minutes or less, subsequent encounter | | | | | | | | | | | | | | | | | | | | | | | | |
| S06361S | | | | | Traumatic hemorrhage of cerebrum, unspecified, with loss of consciousness of 30 minutes or less, sequela | | | | | | | | | | | | | | | | | | | | | | | | |
| S06362A | | | | | Traumatic hemorrhage of cerebrum, unspecified, with loss of consciousness of 31 minutes to 59 minutes, initial encounter | | | | | | | | | | | | | | | | | | | | | | | | |
| S06362D | | | | | Traumatic hemorrhage of cerebrum, unspecified, with loss of consciousness of 31 minutes to 59 minutes, subsequent encounter | | | | | | | | | | | | | | | | | | | | | | | | |
| S06362S | | | | | Traumatic hemorrhage of cerebrum, unspecified, with loss of consciousness of 31 minutes to 59 minutes, sequela | | | | | | | | | | | | | | | | | | | | | | | | |
| S06363A | | | | | Traumatic hemorrhage of cerebrum, unspecified, with loss of consciousness of 1 hours to 5 hours 59 minutes, initial encounter | | | | | | | | | | | | | | | | | | | | | | | | |
| S06363D | | | | | Traumatic hemorrhage of cerebrum, unspecified, with loss of consciousness of 1 hours to 5 hours 59 minutes, subsequent encounter | | | | | | | | | | | | | | | | | | | | | | | | |
| S06363S | | | | | Traumatic hemorrhage of cerebrum, unspecified, with loss of consciousness of 1 hours to 5 hours 59 minutes, sequela | | | | | | | | | | | | | | | | | | | | | | | | |
| S06364A | | | | | Traumatic hemorrhage of cerebrum, unspecified, with loss of consciousness of 6 hours to 24 hours, initial encounter | | | | | | | | | | | | | | | | | | | | | | | | |
| S06364D | | | | | Traumatic hemorrhage of cerebrum, unspecified, with loss of consciousness of 6 hours to 24 hours, subsequent encounter | | | | | | | | | | | | | | | | | | | | | | | | |
| S06364S | | | | | Traumatic hemorrhage of cerebrum, unspecified, with loss of consciousness of 6 hours to 24 hours, sequela | | | | | | | | | | | | | | | | | | | | | | | | |
| S06365A | | | | | Traumatic hemorrhage of cerebrum, unspecified, with loss of consciousness greater than 24 hours with return to pre-existing conscious level, initial encounter | | | | | | | | | | | | | | | | | | | | | | | | |
| S06365D | | | | | Traumatic hemorrhage of cerebrum, unspecified, with loss of consciousness greater than 24 hours with return to pre-existing conscious level, subsequent encounter | | | | | | | | | | | | | | | | | | | | | | | | |
| S06365S | | | | | Traumatic hemorrhage of cerebrum, unspecified, with loss of consciousness greater than 24 hours with return to pre-existing conscious level, sequela | | | | | | | | | | | | | | | | | | | | | | | | |
| S06366A | | | | | Traumatic hemorrhage of cerebrum, unspecified, with loss of consciousness greater than 24 hours without return to pre-existing conscious level with patient surviving, initial encounter | | | | | | | | | | | | | | | | | | | | | | | | |
| S06366D | | | | | Traumatic hemorrhage of cerebrum, unspecified, with loss of consciousness greater than 24 hours without return to pre-existing conscious level with patient surviving, subsequent encounter | | | | | | | | | | | | | | | | | | | | | | | | |
| S06366S | | | | | Traumatic hemorrhage of cerebrum, unspecified, with loss of consciousness greater than 24 hours without return to pre-existing conscious level with patient surviving, sequela | | | | | | | | | | | | | | | | | | | | | | | | |
| S06367A | | | | | Traumatic hemorrhage of cerebrum, unspecified, with loss of consciousness of any duration with death due to brain injury prior to regaining consciousness, initial encounter | | | | | | | | | | | | | | | | | | | | | | | | |
| S06368A | | | | | Traumatic hemorrhage of cerebrum, unspecified, with loss of consciousness of any duration with death due to other cause prior to regaining consciousness, initial encounter | | | | | | | | | | | | | | | | | | | | | | | | |
| S06369A | | | | | Traumatic hemorrhage of cerebrum, unspecified, with loss of consciousness of unspecified duration, initial encounter | | | | | | | | | | | | | | | | | | | | | | | | |
| S06369D | | | | | Traumatic hemorrhage of cerebrum, unspecified, with loss of consciousness of unspecified duration, subsequent encounter | | | | | | | | | | | | | | | | | | | | | | | | |
| S06369S | | | | | Traumatic hemorrhage of cerebrum, unspecified, with loss of consciousness of unspecified duration, sequela | | | | | | | | | | | | | | | | | | | | | | | | |
| S06370A | | | | | Contusion, laceration, and hemorrhage of cerebellum without loss of consciousness, initial encounter | | | | | | | | | | | | | | | | | | | | | | | | |
| S06370D | | | | | Contusion, laceration, and hemorrhage of cerebellum without loss of consciousness, subsequent encounter | | | | | | | | | | | | | | | | | | | | | | | | |
| S06370S | | | | | Contusion, laceration, and hemorrhage of cerebellum without loss of consciousness, sequela | | | | | | | | | | | | | | | | | | | | | | | | |
| S06371A | | | | | Contusion, laceration, and hemorrhage of cerebellum with loss of consciousness of 30 minutes or less, initial encounter | | | | | | | | | | | | | | | | | | | | | | | | |
| S06371D | | | | | Contusion, laceration, and hemorrhage of cerebellum with loss of consciousness of 30 minutes or less, subsequent encounter | | | | | | | | | | | | | | | | | | | | | | | | |
| S06371S | | | | | Contusion, laceration, and hemorrhage of cerebellum with loss of consciousness of 30 minutes or less, sequela | | | | | | | | | | | | | | | | | | | | | | | | |
| S06372A | | | | | Contusion, laceration, and hemorrhage of cerebellum with loss of consciousness of 31 minutes to 59 minutes, initial encounter | | | | | | | | | | | | | | | | | | | | | | | | |
| S06372D | | | | | Contusion, laceration, and hemorrhage of cerebellum with loss of consciousness of 31 minutes to 59 minutes, subsequent encounter | | | | | | | | | | | | | | | | | | | | | | | | |
| S06372S | | | | | Contusion, laceration, and hemorrhage of cerebellum with loss of consciousness of 31 minutes to 59 minutes, sequela | | | | | | | | | | | | | | | | | | | | | | | | |
| S06373A | | | | | Contusion, laceration, and hemorrhage of cerebellum with loss of consciousness of 1 hour to 5 hours 59 minutes, initial encounter | | | | | | | | | | | | | | | | | | | | | | | | |
| S06373D | | | | | Contusion, laceration, and hemorrhage of cerebellum with loss of consciousness of 1 hour to 5 hours 59 minutes, subsequent encounter | | | | | | | | | | | | | | | | | | | | | | | | |
| S06373S | | | | | Contusion, laceration, and hemorrhage of cerebellum with loss of consciousness of 1 hour to 5 hours 59 minutes, sequela | | | | | | | | | | | | | | | | | | | | | | | | |
| S06374A | | | | | Contusion, laceration, and hemorrhage of cerebellum with loss of consciousness of 6 hours to 24 hours, initial encounter | | | | | | | | | | | | | | | | | | | | | | | | |
| S06374D | | | | | Contusion, laceration, and hemorrhage of cerebellum with loss of consciousness of 6 hours to 24 hours, subsequent encounter | | | | | | | | | | | | | | | | | | | | | | | | |
| S06374S | | | | | Contusion, laceration, and hemorrhage of cerebellum with loss of consciousness of 6 hours to 24 hours, sequela | | | | | | | | | | | | | | | | | | | | | | | | |
| S06375A | | | | | Contusion, laceration, and hemorrhage of cerebellum with loss of consciousness greater than 24 hours with return to pre-existing conscious level, initial encounter | | | | | | | | | | | | | | | | | | | | | | | | |
| S06375D | | | | | Contusion, laceration, and hemorrhage of cerebellum with loss of consciousness greater than 24 hours with return to pre-existing conscious level, subsequent encounter | | | | | | | | | | | | | | | | | | | | | | | | |
| S06375S | | | | | Contusion, laceration, and hemorrhage of cerebellum with loss of consciousness greater than 24 hours with return to pre-existing conscious level, sequela | | | | | | | | | | | | | | | | | | | | | | | | |
| S06376A | | | | | Contusion, laceration, and hemorrhage of cerebellum with loss of consciousness greater than 24 hours without return to pre-existing conscious level with patient surviving, initial encounter | | | | | | | | | | | | | | | | | | | | | | | | |
| S06376D | | | | | Contusion, laceration, and hemorrhage of cerebellum with loss of consciousness greater than 24 hours without return to pre-existing conscious level with patient surviving, subsequent encounter | | | | | | | | | | | | | | | | | | | | | | | | |
| S06376S | | | | | Contusion, laceration, and hemorrhage of cerebellum with loss of consciousness greater than 24 hours without return to pre-existing conscious level with patient surviving, sequela | | | | | | | | | | | | | | | | | | | | | | | | |
| S06377A | | | | | Contusion, laceration, and hemorrhage of cerebellum with loss of consciousness of any duration with death due to brain injury prior to regaining consciousness, initial encounter | | | | | | | | | | | | | | | | | | | | | | | | |
| S06378A | | | | | Contusion, laceration, and hemorrhage of cerebellum with loss of consciousness of any duration with death due to other cause prior to regaining consciousness, initial encounter | | | | | | | | | | | | | | | | | | | | | | | | |
| S06379A | | | | | Contusion, laceration, and hemorrhage of cerebellum with loss of consciousness of unspecified duration, initial encounter | | | | | | | | | | | | | | | | | | | | | | | | |
| S06379D | | | | | Contusion, laceration, and hemorrhage of cerebellum with loss of consciousness of unspecified duration, subsequent encounter | | | | | | | | | | | | | | | | | | | | | | | | |
| S06379S | | | | | Contusion, laceration, and hemorrhage of cerebellum with loss of consciousness of unspecified duration, sequela | | | | | | | | | | | | | | | | | | | | | | | | |
| S06380A | | | | | Contusion, laceration, and hemorrhage of brainstem without loss of consciousness, initial encounter | | | | | | | | | | | | | | | | | | | | | | | | |
| S06380D | | | | | Contusion, laceration, and hemorrhage of brainstem without loss of consciousness, subsequent encounter | | | | | | | | | | | | | | | | | | | | | | | | |
| S06380S | | | | | Contusion, laceration, and hemorrhage of brainstem without loss of consciousness, sequela | | | | | | | | | | | | | | | | | | | | | | | | |
| S06381A | | | | | Contusion, laceration, and hemorrhage of brainstem with loss of consciousness of 30 minutes or less, initial encounter | | | | | | | | | | | | | | | | | | | | | | | | |
| S06381D | | | | | Contusion, laceration, and hemorrhage of brainstem with loss of consciousness of 30 minutes or less, subsequent encounter | | | | | | | | | | | | | | | | | | | | | | | | |
| S06381S | | | | | Contusion, laceration, and hemorrhage of brainstem with loss of consciousness of 30 minutes or less, sequela | | | | | | | | | | | | | | | | | | | | | | | | |
| S06382A | | | | | Contusion, laceration, and hemorrhage of brainstem with loss of consciousness of 31 minutes to 59 minutes, initial encounter | | | | | | | | | | | | | | | | | | | | | | | | |
| S06382D | | | | | Contusion, laceration, and hemorrhage of brainstem with loss of consciousness of 31 minutes to 59 minutes, subsequent encounter | | | | | | | | | | | | | | | | | | | | | | | | |
| S06382S | | | | | Contusion, laceration, and hemorrhage of brainstem with loss of consciousness of 31 minutes to 59 minutes, sequela | | | | | | | | | | | | | | | | | | | | | | | | |
| S06383A | | | | | Contusion, laceration, and hemorrhage of brainstem with loss of consciousness of 1 hour to 5 hours 59 minutes, initial encounter | | | | | | | | | | | | | | | | | | | | | | | | |
| S06383D | | | | | Contusion, laceration, and hemorrhage of brainstem with loss of consciousness of 1 hour to 5 hours 59 minutes, subsequent encounter | | | | | | | | | | | | | | | | | | | | | | | | |
| S06383S | | | | | Contusion, laceration, and hemorrhage of brainstem with loss of consciousness of 1 hour to 5 hours 59 minutes, sequela | | | | | | | | | | | | | | | | | | | | | | | | |
| S06384A | | | | | Contusion, laceration, and hemorrhage of brainstem with loss of consciousness of 6 hours to 24 hours, initial encounter | | | | | | | | | | | | | | | | | | | | | | | | |
| S06384D | | | | | Contusion, laceration, and hemorrhage of brainstem with loss of consciousness of 6 hours to 24 hours, subsequent encounter | | | | | | | | | | | | | | | | | | | | | | | | |
| S06384S | | | | | Contusion, laceration, and hemorrhage of brainstem with loss of consciousness of 6 hours to 24 hours, sequela | | | | | | | | | | | | | | | | | | | | | | | | |
| S06385A | | | | | Contusion, laceration, and hemorrhage of brainstem with loss of consciousness greater than 24 hours with return to pre-existing conscious level, initial encounter | | | | | | | | | | | | | | | | | | | | | | | | |
| S06385D | | | | | Contusion, laceration, and hemorrhage of brainstem with loss of consciousness greater than 24 hours with return to pre-existing conscious level, subsequent encounter | | | | | | | | | | | | | | | | | | | | | | | | |
| S06385S | | | | | Contusion, laceration, and hemorrhage of brainstem with loss of consciousness greater than 24 hours with return to pre-existing conscious level, sequela | | | | | | | | | | | | | | | | | | | | | | | | |
| S06386A | | | | | Contusion, laceration, and hemorrhage of brainstem with loss of consciousness greater than 24 hours without return to pre-existing conscious level with patient surviving, initial encounter | | | | | | | | | | | | | | | | | | | | | | | | |
| S06386D | | | | | Contusion, laceration, and hemorrhage of brainstem with loss of consciousness greater than 24 hours without return to pre-existing conscious level with patient surviving, subsequent encounter | | | | | | | | | | | | | | | | | | | | | | | | |
| S06386S | | | | | Contusion, laceration, and hemorrhage of brainstem with loss of consciousness greater than 24 hours without return to pre-existing conscious level with patient surviving, sequela | | | | | | | | | | | | | | | | | | | | | | | | |
| S06387A | | | | | Contusion, laceration, and hemorrhage of brainstem with loss of consciousness of any duration with death due to brain injury prior to regaining consciousness, initial encounter | | | | | | | | | | | | | | | | | | | | | | | | |
| S06388A | | | | | Contusion, laceration, and hemorrhage of brainstem with loss of consciousness of any duration with death due to other cause prior to regaining consciousness, initial encounter | | | | | | | | | | | | | | | | | | | | | | | | |
| S06389A | | | | | Contusion, laceration, and hemorrhage of brainstem with loss of consciousness of unspecified duration, initial encounter | | | | | | | | | | | | | | | | | | | | | | | | |
| S06389D | | | | | Contusion, laceration, and hemorrhage of brainstem with loss of consciousness of unspecified duration, subsequent encounter | | | | | | | | | | | | | | | | | | | | | | | | |
| S078XXS | | | | | Crushing injury of other parts of head, sequela | | | | | | | | | | | | | | | | | | | | | | | | |
| S06389S | | | | | Contusion, laceration, and hemorrhage of brainstem with loss of consciousness of unspecified duration, sequela | | | | | | | | | | | | | | | | | | | | | | | | |
| S06335D | | | | | Contusion and laceration of cerebrum, unspecified, with loss of consciousness greater than 24 hours with return to pre-existing conscious level, subsequent encounter | | | | | | | | | | | | | | | | | | | | | | | | |
| S06335S | | | | | Contusion and laceration of cerebrum, unspecified, with loss of consciousness greater than 24 hours with return to pre-existing conscious level, sequela | | | | | | | | | | | | | | | | | | | | | | | | |
| S06355A | | | | | Traumatic hemorrhage of left cerebrum with loss of consciousness greater than 24 hours with return to pre-existing conscious level, initial encounter | | | | | | | | | | | | | | | | | | | | | | | | |
| S06355D | | | | | Traumatic hemorrhage of left cerebrum with loss of consciousness greater than 24 hours with return to pre-existing conscious level, subsequent encounter | | | | | | | | | | | | | | | | | | | | | | | | |
| S064X0A | | | | | Epidural hemorrhage without loss of consciousness, initial encounter | | | | | | | | | | | | | | | | | | | | | | | | |
| S064X0D | | | | | Epidural hemorrhage without loss of consciousness, subsequent encounter | | | | | | | | | | | | | | | | | | | | | | | | |
| S064X0S | | | | | Epidural hemorrhage without loss of consciousness, sequela | | | | | | | | | | | | | | | | | | | | | | | | |
| S064X1A | | | | | Epidural hemorrhage with loss of consciousness of 30 minutes or less, initial encounter | | | | | | | | | | | | | | | | | | | | | | | | |
| S064X1D | | | | | Epidural hemorrhage with loss of consciousness of 30 minutes or less, subsequent encounter | | | | | | | | | | | | | | | | | | | | | | | | |
| S064X1S | | | | | Epidural hemorrhage with loss of consciousness of 30 minutes or less, sequela | | | | | | | | | | | | | | | | | | | | | | | | |
| S064X2A | | | | | Epidural hemorrhage with loss of consciousness of 31 minutes to 59 minutes, initial encounter | | | | | | | | | | | | | | | | | | | | | | | | |
| S064X2D | | | | | Epidural hemorrhage with loss of consciousness of 31 minutes to 59 minutes, subsequent encounter | | | | | | | | | | | | | | | | | | | | | | | | |
| S064X2S | | | | | Epidural hemorrhage with loss of consciousness of 31 minutes to 59 minutes, sequela | | | | | | | | | | | | | | | | | | | | | | | | |
| S064X3A | | | | | Epidural hemorrhage with loss of consciousness of 1 hour to 5 hours 59 minutes, initial encounter | | | | | | | | | | | | | | | | | | | | | | | | |
| S064X3D | | | | | Epidural hemorrhage with loss of consciousness of 1 hour to 5 hours 59 minutes, subsequent encounter | | | | | | | | | | | | | | | | | | | | | | | | |
| S064X3S | | | | | Epidural hemorrhage with loss of consciousness of 1 hour to 5 hours 59 minutes, sequela | | | | | | | | | | | | | | | | | | | | | | | | |
| S064X4A | | | | | Epidural hemorrhage with loss of consciousness of 6 hours to 24 hours, initial encounter | | | | | | | | | | | | | | | | | | | | | | | | |
| S064X4D | | | | | Epidural hemorrhage with loss of consciousness of 6 hours to 24 hours, subsequent encounter | | | | | | | | | | | | | | | | | | | | | | | | |
| S064X4S | | | | | Epidural hemorrhage with loss of consciousness of 6 hours to 24 hours, sequela | | | | | | | | | | | | | | | | | | | | | | | | |
| S064X5A | | | | | Epidural hemorrhage with loss of consciousness greater than 24 hours with return to pre-existing conscious level, initial encounter | | | | | | | | | | | | | | | | | | | | | | | | |
| S064X5D | | | | | Epidural hemorrhage with loss of consciousness greater than 24 hours with return to pre-existing conscious level, subsequent encounter | | | | | | | | | | | | | | | | | | | | | | | | |
| S064X5S | | | | | Epidural hemorrhage with loss of consciousness greater than 24 hours with return to pre-existing conscious level, sequela | | | | | | | | | | | | | | | | | | | | | | | | |
| S064X6A | | | | | Epidural hemorrhage with loss of consciousness greater than 24 hours without return to pre-existing conscious level with patient surviving, initial encounter | | | | | | | | | | | | | | | | | | | | | | | | |
| S064X6D | | | | | Epidural hemorrhage with loss of consciousness greater than 24 hours without return to pre-existing conscious level with patient surviving, subsequent encounter | | | | | | | | | | | | | | | | | | | | | | | | |
| S064X6S | | | | | Epidural hemorrhage with loss of consciousness greater than 24 hours without return to pre-existing conscious level with patient surviving, sequela | | | | | | | | | | | | | | | | | | | | | | | | |
| S064X7A | | | | | Epidural hemorrhage with loss of consciousness of any duration with death due to brain injury prior to regaining consciousness, initial encounter | | | | | | | | | | | | | | | | | | | | | | | | |
| S064X8A | | | | | Epidural hemorrhage with loss of consciousness of any duration with death due to other causes prior to regaining consciousness, initial encounter | | | | | | | | | | | | | | | | | | | | | | | | |
| S064X9A | | | | | Epidural hemorrhage with loss of consciousness of unspecified duration, initial encounter | | | | | | | | | | | | | | | | | | | | | | | | |
| S064X9D | | | | | Epidural hemorrhage with loss of consciousness of unspecified duration, subsequent encounter | | | | | | | | | | | | | | | | | | | | | | | | |
| S064X9S | | | | | Epidural hemorrhage with loss of consciousness of unspecified duration, sequela | | | | | | | | | | | | | | | | | | | | | | | | |
| S065X0A | | | | | Traumatic subdural hemorrhage without loss of consciousness, initial encounter | | | | | | | | | | | | | | | | | | | | | | | | |
| S065X0D | | | | | Traumatic subdural hemorrhage without loss of consciousness, subsequent encounter | | | | | | | | | | | | | | | | | | | | | | | | |
| S065X0S | | | | | Traumatic subdural hemorrhage without loss of consciousness, sequela | | | | | | | | | | | | | | | | | | | | | | | | |
| S065X1A | | | | | Traumatic subdural hemorrhage with loss of consciousness of 30 minutes or less, initial encounter | | | | | | | | | | | | | | | | | | | | | | | | |
| S065X1D | | | | | Traumatic subdural hemorrhage with loss of consciousness of 30 minutes or less, subsequent encounter | | | | | | | | | | | | | | | | | | | | | | | | |
| S065X1S | | | | | Traumatic subdural hemorrhage with loss of consciousness of 30 minutes or less, sequela | | | | | | | | | | | | | | | | | | | | | | | | |
| S065X2A | | | | | Traumatic subdural hemorrhage with loss of consciousness of 31 minutes to 59 minutes, initial encounter | | | | | | | | | | | | | | | | | | | | | | | | |
| S065X2D | | | | | Traumatic subdural hemorrhage with loss of consciousness of 31 minutes to 59 minutes, subsequent encounter | | | | | | | | | | | | | | | | | | | | | | | | |
| S065X2S | | | | | Traumatic subdural hemorrhage with loss of consciousness of 31 minutes to 59 minutes, sequela | | | | | | | | | | | | | | | | | | | | | | | | |
| S065X3A | | | | | Traumatic subdural hemorrhage with loss of consciousness of 1 hour to 5 hours 59 minutes, initial encounter | | | | | | | | | | | | | | | | | | | | | | | | |
| S065X3D | | | | | Traumatic subdural hemorrhage with loss of consciousness of 1 hour to 5 hours 59 minutes, subsequent encounter | | | | | | | | | | | | | | | | | | | | | | | | |
| S065X3S | | | | | Traumatic subdural hemorrhage with loss of consciousness of 1 hour to 5 hours 59 minutes, sequela | | | | | | | | | | | | | | | | | | | | | | | | |
| S065X4A | | | | | Traumatic subdural hemorrhage with loss of consciousness of 6 hours to 24 hours, initial encounter | | | | | | | | | | | | | | | | | | | | | | | | |
| S065X4D | | | | | Traumatic subdural hemorrhage with loss of consciousness of 6 hours to 24 hours, subsequent encounter | | | | | | | | | | | | | | | | | | | | | | | | |
| S065X4S | | | | | Traumatic subdural hemorrhage with loss of consciousness of 6 hours to 24 hours, sequela | | | | | | | | | | | | | | | | | | | | | | | | |
| S065X5A | | | | | Traumatic subdural hemorrhage with loss of consciousness greater than 24 hours with return to pre-existing conscious level, initial encounter | | | | | | | | | | | | | | | | | | | | | | | | |
| S065X5D | | | | | Traumatic subdural hemorrhage with loss of consciousness greater than 24 hours with return to pre-existing conscious level, subsequent encounter | | | | | | | | | | | | | | | | | | | | | | | | |
| S065X5S | | | | | Traumatic subdural hemorrhage with loss of consciousness greater than 24 hours with return to pre-existing conscious level, sequela | | | | | | | | | | | | | | | | | | | | | | | | |
| S065X6A | | | | | Traumatic subdural hemorrhage with loss of consciousness greater than 24 hours without return to pre-existing conscious level with patient surviving, initial encounter | | | | | | | | | | | | | | | | | | | | | | | | |
| S065X6D | | | | | Traumatic subdural hemorrhage with loss of consciousness greater than 24 hours without return to pre-existing conscious level with patient surviving, subsequent encounter | | | | | | | | | | | | | | | | | | | | | | | | |
| S065X6S | | | | | Traumatic subdural hemorrhage with loss of consciousness greater than 24 hours without return to pre-existing conscious level with patient surviving, sequela | | | | | | | | | | | | | | | | | | | | | | | | |
| S065X7A | | | | | Traumatic subdural hemorrhage with loss of consciousness of any duration with death due to brain injury before regaining consciousness, initial encounter | | | | | | | | | | | | | | | | | | | | | | | | |
| S065X8A | | | | | Traumatic subdural hemorrhage with loss of consciousness of any duration with death due to other cause before regaining consciousness, initial encounter | | | | | | | | | | | | | | | | | | | | | | | | |
| S065X9A | | | | | Traumatic subdural hemorrhage with loss of consciousness of unspecified duration, initial encounter | | | | | | | | | | | | | | | | | | | | | | | | |
| S065X9D | | | | | Traumatic subdural hemorrhage with loss of consciousness of unspecified duration, subsequent encounter | | | | | | | | | | | | | | | | | | | | | | | | |
| S065X9S | | | | | Traumatic subdural hemorrhage with loss of consciousness of unspecified duration, sequela | | | | | | | | | | | | | | | | | | | | | | | | |
| S066X0A | | | | | Traumatic subarachnoid hemorrhage without loss of consciousness, initial encounter | | | | | | | | | | | | | | | | | | | | | | | | |
| S066X0D | | | | | Traumatic subarachnoid hemorrhage without loss of consciousness, subsequent encounter | | | | | | | | | | | | | | | | | | | | | | | | |
| S066X0S | | | | | Traumatic subarachnoid hemorrhage without loss of consciousness, sequela | | | | | | | | | | | | | | | | | | | | | | | | |
| S066X1A | | | | | Traumatic subarachnoid hemorrhage with loss of consciousness of 30 minutes or less, initial encounter | | | | | | | | | | | | | | | | | | | | | | | | |
| S066X1D | | | | | Traumatic subarachnoid hemorrhage with loss of consciousness of 30 minutes or less, subsequent encounter | | | | | | | | | | | | | | | | | | | | | | | | |
| S066X1S | | | | | Traumatic subarachnoid hemorrhage with loss of consciousness of 30 minutes or less, sequela | | | | | | | | | | | | | | | | | | | | | | | | |
| S066X2A | | | | | Traumatic subarachnoid hemorrhage with loss of consciousness of 31 minutes to 59 minutes, initial encounter | | | | | | | | | | | | | | | | | | | | | | | | |
| S066X2D | | | | | Traumatic subarachnoid hemorrhage with loss of consciousness of 31 minutes to 59 minutes, subsequent encounter | | | | | | | | | | | | | | | | | | | | | | | | |
| S066X2S | | | | | Traumatic subarachnoid hemorrhage with loss of consciousness of 31 minutes to 59 minutes, sequela | | | | | | | | | | | | | | | | | | | | | | | | |
| S066X3A | | | | | Traumatic subarachnoid hemorrhage with loss of consciousness of 1 hour to 5 hours 59 minutes, initial encounter | | | | | | | | | | | | | | | | | | | | | | | | |
| S066X3D | | | | | Traumatic subarachnoid hemorrhage with loss of consciousness of 1 hour to 5 hours 59 minutes, subsequent encounter | | | | | | | | | | | | | | | | | | | | | | | | |
| S066X3S | | | | | Traumatic subarachnoid hemorrhage with loss of consciousness of 1 hour to 5 hours 59 minutes, sequela | | | | | | | | | | | | | | | | | | | | | | | | |
| S066X4A | | | | | Traumatic subarachnoid hemorrhage with loss of consciousness of 6 hours to 24 hours, initial encounter | | | | | | | | | | | | | | | | | | | | | | | | |
| S066X4D | | | | | Traumatic subarachnoid hemorrhage with loss of consciousness of 6 hours to 24 hours, subsequent encounter | | | | | | | | | | | | | | | | | | | | | | | | |
| S066X4S | | | | | Traumatic subarachnoid hemorrhage with loss of consciousness of 6 hours to 24 hours, sequela | | | | | | | | | | | | | | | | | | | | | | | | |
| S066X5A | | | | | Traumatic subarachnoid hemorrhage with loss of consciousness greater than 24 hours with return to pre-existing conscious level, initial encounter | | | | | | | | | | | | | | | | | | | | | | | | |
| S066X5D | | | | | Traumatic subarachnoid hemorrhage with loss of consciousness greater than 24 hours with return to pre-existing conscious level, subsequent encounter | | | | | | | | | | | | | | | | | | | | | | | | |
| S066X5S | | | | | Traumatic subarachnoid hemorrhage with loss of consciousness greater than 24 hours with return to pre-existing conscious level, sequela | | | | | | | | | | | | | | | | | | | | | | | | |
| S066X6A | | | | | Traumatic subarachnoid hemorrhage with loss of consciousness greater than 24 hours without return to pre-existing conscious level with patient surviving, initial encounter | | | | | | | | | | | | | | | | | | | | | | | | |
| S066X6D | | | | | Traumatic subarachnoid hemorrhage with loss of consciousness greater than 24 hours without return to pre-existing conscious level with patient surviving, subsequent encounter | | | | | | | | | | | | | | | | | | | | | | | | |
| S066X6S | | | | | Traumatic subarachnoid hemorrhage with loss of consciousness greater than 24 hours without return to pre-existing conscious level with patient surviving, sequela | | | | | | | | | | | | | | | | | | | | | | | | |
| S066X7A | | | | | Traumatic subarachnoid hemorrhage with loss of consciousness of any duration with death due to brain injury prior to regaining consciousness, initial encounter | | | | | | | | | | | | | | | | | | | | | | | | |
| S066X8A | | | | | Traumatic subarachnoid hemorrhage with loss of consciousness of any duration with death due to other cause prior to regaining consciousness, initial encounter | | | | | | | | | | | | | | | | | | | | | | | | |
| S066X9A | | | | | Traumatic subarachnoid hemorrhage with loss of consciousness of unspecified duration, initial encounter | | | | | | | | | | | | | | | | | | | | | | | | |
| S066X9D | | | | | Traumatic subarachnoid hemorrhage with loss of consciousness of unspecified duration, subsequent encounter | | | | | | | | | | | | | | | | | | | | | | | | |
| S066X9S | | | | | Traumatic subarachnoid hemorrhage with loss of consciousness of unspecified duration, sequela | | | | | | | | | | | | | | | | | | | | | | | | |
| S06810A | | | | | Injury of right internal carotid artery, intracranial portion, not elsewhere classified without loss of consciousness, initial encounter | | | | | | | | | | | | | | | | | | | | | | | | |
| S06810D | | | | | Injury of right internal carotid artery, intracranial portion, not elsewhere classified without loss of consciousness, subsequent encounter | | | | | | | | | | | | | | | | | | | | | | | | |
| S06810S | | | | | Injury of right internal carotid artery, intracranial portion, not elsewhere classified without loss of consciousness, sequela | | | | | | | | | | | | | | | | | | | | | | | | |
| S06811A | | | | | Injury of right internal carotid artery, intracranial portion, not elsewhere classified with loss of consciousness of 30 minutes or less, initial encounter | | | | | | | | | | | | | | | | | | | | | | | | |
| S06811D | | | | | Injury of right internal carotid artery, intracranial portion, not elsewhere classified with loss of consciousness of 30 minutes or less, subsequent encounter | | | | | | | | | | | | | | | | | | | | | | | | |
| S06811S | | | | | Injury of right internal carotid artery, intracranial portion, not elsewhere classified with loss of consciousness of 30 minutes or less, sequela | | | | | | | | | | | | | | | | | | | | | | | | |
| S06812A | | | | | Injury of right internal carotid artery, intracranial portion, not elsewhere classified with loss of consciousness of 31 minutes to 59 minutes, initial encounter | | | | | | | | | | | | | | | | | | | | | | | | |
| S06812D | | | | | Injury of right internal carotid artery, intracranial portion, not elsewhere classified with loss of consciousness of 31 minutes to 59 minutes, subsequent encounter | | | | | | | | | | | | | | | | | | | | | | | | |
| S06812S | | | | | Injury of right internal carotid artery, intracranial portion, not elsewhere classified with loss of consciousness of 31 minutes to 59 minutes, sequela | | | | | | | | | | | | | | | | | | | | | | | | |
| S06813A | | | | | Injury of right internal carotid artery, intracranial portion, not elsewhere classified with loss of consciousness of 1 hour to 5 hours 59 minutes, initial encounter | | | | | | | | | | | | | | | | | | | | | | | | |
| S06813D | | | | | Injury of right internal carotid artery, intracranial portion, not elsewhere classified with loss of consciousness of 1 hour to 5 hours 59 minutes, subsequent encounter | | | | | | | | | | | | | | | | | | | | | | | | |
| S06813S | | | | | Injury of right internal carotid artery, intracranial portion, not elsewhere classified with loss of consciousness of 1 hour to 5 hours 59 minutes, sequela | | | | | | | | | | | | | | | | | | | | | | | | |
| S06814A | | | | | Injury of right internal carotid artery, intracranial portion, not elsewhere classified with loss of consciousness of 6 hours to 24 hours, initial encounter | | | | | | | | | | | | | | | | | | | | | | | | |
| S06814D | | | | | Injury of right internal carotid artery, intracranial portion, not elsewhere classified with loss of consciousness of 6 hours to 24 hours, subsequent encounter | | | | | | | | | | | | | | | | | | | | | | | | |
| S06814S | | | | | Injury of right internal carotid artery, intracranial portion, not elsewhere classified with loss of consciousness of 6 hours to 24 hours, sequela | | | | | | | | | | | | | | | | | | | | | | | | |
| S06815A | | | | | Injury of right internal carotid artery, intracranial portion, not elsewhere classified with loss of consciousness greater than 24 hours with return to pre-existing conscious level, initial encounter | | | | | | | | | | | | | | | | | | | | | | | | |
| S06815D | | | | | Injury of right internal carotid artery, intracranial portion, not elsewhere classified with loss of consciousness greater than 24 hours with return to pre-existing conscious level, subsequent encounter | | | | | | | | | | | | | | | | | | | | | | | | |
| S06815S | | | | | Injury of right internal carotid artery, intracranial portion, not elsewhere classified with loss of consciousness greater than 24 hours with return to pre-existing conscious level, sequela | | | | | | | | | | | | | | | | | | | | | | | | |
| S06816A | | | | | Injury of right internal carotid artery, intracranial portion, not elsewhere classified with loss of consciousness greater than 24 hours without return to pre-existing conscious level with patient surviving, initial encounter | | | | | | | | | | | | | | | | | | | | | | | | |
| S079XXA | | | | | Crushing injury of head, part unspecified, initial encounter | | | | | | | | | | | | | | | | | | | | | | | | |
| S06816D | | | | | Injury of right internal carotid artery, intracranial portion, not elsewhere classified with loss of consciousness greater than 24 hours without return to pre-existing conscious level with patient surviving, subsequent encounter | | | | | | | | | | | | | | | | | | | | | | | | |
| S06816S | | | | | Injury of right internal carotid artery, intracranial portion, not elsewhere classified with loss of consciousness greater than 24 hours without return to pre-existing conscious level with patient surviving, sequela | | | | | | | | | | | | | | | | | | | | | | | | |
| S06817A | | | | | Injury of right internal carotid artery, intracranial portion, not elsewhere classified with loss of consciousness of any duration with death due to brain injury prior to regaining consciousness, initial encounter | | | | | | | | | | | | | | | | | | | | | | | | |
| S06818A | | | | | Injury of right internal carotid artery, intracranial portion, not elsewhere classified with loss of consciousness of any duration with death due to other cause prior to regaining consciousness, initial encounter | | | | | | | | | | | | | | | | | | | | | | | | |
| S06819A | | | | | Injury of right internal carotid artery, intracranial portion, not elsewhere classified with loss of consciousness of unspecified duration, initial encounter | | | | | | | | | | | | | | | | | | | | | | | | |
| S06819D | | | | | Injury of right internal carotid artery, intracranial portion, not elsewhere classified with loss of consciousness of unspecified duration, subsequent encounter | | | | | | | | | | | | | | | | | | | | | | | | |
| S06819S | | | | | Injury of right internal carotid artery, intracranial portion, not elsewhere classified with loss of consciousness of unspecified duration, sequela | | | | | | | | | | | | | | | | | | | | | | | | |
| S06820A | | | | | Injury of left internal carotid artery, intracranial portion, not elsewhere classified without loss of consciousness, initial encounter | | | | | | | | | | | | | | | | | | | | | | | | |
| S06820D | | | | | Injury of left internal carotid artery, intracranial portion, not elsewhere classified without loss of consciousness, subsequent encounter | | | | | | | | | | | | | | | | | | | | | | | | |
| S06820S | | | | | Injury of left internal carotid artery, intracranial portion, not elsewhere classified without loss of consciousness, sequela | | | | | | | | | | | | | | | | | | | | | | | | |
| S06821A | | | | | Injury of left internal carotid artery, intracranial portion, not elsewhere classified with loss of consciousness of 30 minutes or less, initial encounter | | | | | | | | | | | | | | | | | | | | | | | | |
| S06821D | | | | | Injury of left internal carotid artery, intracranial portion, not elsewhere classified with loss of consciousness of 30 minutes or less, subsequent encounter | | | | | | | | | | | | | | | | | | | | | | | | |
| S06821S | | | | | Injury of left internal carotid artery, intracranial portion, not elsewhere classified with loss of consciousness of 30 minutes or less, sequela | | | | | | | | | | | | | | | | | | | | | | | | |
| S06822A | | | | | Injury of left internal carotid artery, intracranial portion, not elsewhere classified with loss of consciousness of 31 minutes to 59 minutes, initial encounter | | | | | | | | | | | | | | | | | | | | | | | | |
| S06822D | | | | | Injury of left internal carotid artery, intracranial portion, not elsewhere classified with loss of consciousness of 31 minutes to 59 minutes, subsequent encounter | | | | | | | | | | | | | | | | | | | | | | | | |
| S06822S | | | | | Injury of left internal carotid artery, intracranial portion, not elsewhere classified with loss of consciousness of 31 minutes to 59 minutes, sequela | | | | | | | | | | | | | | | | | | | | | | | | |
| S06823A | | | | | Injury of left internal carotid artery, intracranial portion, not elsewhere classified with loss of consciousness of 1 hour to 5 hours 59 minutes, initial encounter | | | | | | | | | | | | | | | | | | | | | | | | |
| S06823D | | | | | Injury of left internal carotid artery, intracranial portion, not elsewhere classified with loss of consciousness of 1 hour to 5 hours 59 minutes, subsequent encounter | | | | | | | | | | | | | | | | | | | | | | | | |
| S06823S | | | | | Injury of left internal carotid artery, intracranial portion, not elsewhere classified with loss of consciousness of 1 hour to 5 hours 59 minutes, sequela | | | | | | | | | | | | | | | | | | | | | | | | |
| S06824A | | | | | Injury of left internal carotid artery, intracranial portion, not elsewhere classified with loss of consciousness of 6 hours to 24 hours, initial encounter | | | | | | | | | | | | | | | | | | | | | | | | |
| S06824D | | | | | Injury of left internal carotid artery, intracranial portion, not elsewhere classified with loss of consciousness of 6 hours to 24 hours, subsequent encounter | | | | | | | | | | | | | | | | | | | | | | | | |
| S06824S | | | | | Injury of left internal carotid artery, intracranial portion, not elsewhere classified with loss of consciousness of 6 hours to 24 hours, sequela | | | | | | | | | | | | | | | | | | | | | | | | |
| S06825A | | | | | Injury of left internal carotid artery, intracranial portion, not elsewhere classified with loss of consciousness greater than 24 hours with return to pre-existing conscious level, initial encounter | | | | | | | | | | | | | | | | | | | | | | | | |
| S06825D | | | | | Injury of left internal carotid artery, intracranial portion, not elsewhere classified with loss of consciousness greater than 24 hours with return to pre-existing conscious level, subsequent encounter | | | | | | | | | | | | | | | | | | | | | | | | |
| S06825S | | | | | Injury of left internal carotid artery, intracranial portion, not elsewhere classified with loss of consciousness greater than 24 hours with return to pre-existing conscious level, sequela | | | | | | | | | | | | | | | | | | | | | | | | |
| S06826A | | | | | Injury of left internal carotid artery, intracranial portion, not elsewhere classified with loss of consciousness greater than 24 hours without return to pre-existing conscious level with patient surviving, initial encounter | | | | | | | | | | | | | | | | | | | | | | | | |
| S06826D | | | | | Injury of left internal carotid artery, intracranial portion, not elsewhere classified with loss of consciousness greater than 24 hours without return to pre-existing conscious level with patient surviving, subsequent encounter | | | | | | | | | | | | | | | | | | | | | | | | |
| S06826S | | | | | Injury of left internal carotid artery, intracranial portion, not elsewhere classified with loss of consciousness greater than 24 hours without return to pre-existing conscious level with patient surviving, sequela | | | | | | | | | | | | | | | | | | | | | | | | |
| S06827A | | | | | Injury of left internal carotid artery, intracranial portion, not elsewhere classified with loss of consciousness of any duration with death due to brain injury prior to regaining consciousness, initial encounter | | | | | | | | | | | | | | | | | | | | | | | | |
| S06828A | | | | | Injury of left internal carotid artery, intracranial portion, not elsewhere classified with loss of consciousness of any duration with death due to other cause prior to regaining consciousness, initial encounter | | | | | | | | | | | | | | | | | | | | | | | | |
| S06829A | | | | | Injury of left internal carotid artery, intracranial portion, not elsewhere classified with loss of consciousness of unspecified duration, initial encounter | | | | | | | | | | | | | | | | | | | | | | | | |
| S06829D | | | | | Injury of left internal carotid artery, intracranial portion, not elsewhere classified with loss of consciousness of unspecified duration, subsequent encounter | | | | | | | | | | | | | | | | | | | | | | | | |
| S06829S | | | | | Injury of left internal carotid artery, intracranial portion, not elsewhere classified with loss of consciousness of unspecified duration, sequela | | | | | | | | | | | | | | | | | | | | | | | | |
| S06890A | | | | | Other specified intracranial injury without loss of consciousness, initial encounter | | | | | | | | | | | | | | | | | | | | | | | | |
| S06890D | | | | | Other specified intracranial injury without loss of consciousness, subsequent encounter | | | | | | | | | | | | | | | | | | | | | | | | |
| S06890S | | | | | Other specified intracranial injury without loss of consciousness, sequela | | | | | | | | | | | | | | | | | | | | | | | | |
| S06891A | | | | | Other specified intracranial injury with loss of consciousness of 30 minutes or less, initial encounter | | | | | | | | | | | | | | | | | | | | | | | | |
| S06891D | | | | | Other specified intracranial injury with loss of consciousness of 30 minutes or less, subsequent encounter | | | | | | | | | | | | | | | | | | | | | | | | |
| S06891S | | | | | Other specified intracranial injury with loss of consciousness of 30 minutes or less, sequela | | | | | | | | | | | | | | | | | | | | | | | | |
| W880XXS | | | | | Exposure to X-rays, sequela | | | | | | | | | | | | | | | | | | | | | | | | |
| S06892A | | | | | Other specified intracranial injury with loss of consciousness of 31 minutes to 59 minutes, initial encounter | | | | | | | | | | | | | | | | | | | | | | | | |
| S06892D | | | | | Other specified intracranial injury with loss of consciousness of 31 minutes to 59 minutes, subsequent encounter | | | | | | | | | | | | | | | | | | | | | | | | |
| S06892S | | | | | Other specified intracranial injury with loss of consciousness of 31 minutes to 59 minutes, sequela | | | | | | | | | | | | | | | | | | | | | | | | |
| S06893A | | | | | Other specified intracranial injury with loss of consciousness of 1 hour to 5 hours 59 minutes, initial encounter | | | | | | | | | | | | | | | | | | | | | | | | |
| S06893D | | | | | Other specified intracranial injury with loss of consciousness of 1 hour to 5 hours 59 minutes, subsequent encounter | | | | | | | | | | | | | | | | | | | | | | | | |
| S06893S | | | | | Other specified intracranial injury with loss of consciousness of 1 hour to 5 hours 59 minutes, sequela | | | | | | | | | | | | | | | | | | | | | | | | |
| S06894A | | | | | Other specified intracranial injury with loss of consciousness of 6 hours to 24 hours, initial encounter | | | | | | | | | | | | | | | | | | | | | | | | |
| S06894D | | | | | Other specified intracranial injury with loss of consciousness of 6 hours to 24 hours, subsequent encounter | | | | | | | | | | | | | | | | | | | | | | | | |
| S06894S | | | | | Other specified intracranial injury with loss of consciousness of 6 hours to 24 hours, sequela | | | | | | | | | | | | | | | | | | | | | | | | |
| S06895A | | | | | Other specified intracranial injury with loss of consciousness greater than 24 hours with return to pre-existing conscious level, initial encounter | | | | | | | | | | | | | | | | | | | | | | | | |
| S06895D | | | | | Other specified intracranial injury with loss of consciousness greater than 24 hours with return to pre-existing conscious level, subsequent encounter | | | | | | | | | | | | | | | | | | | | | | | | |
| S06895S | | | | | Other specified intracranial injury with loss of consciousness greater than 24 hours with return to pre-existing conscious level, sequela | | | | | | | | | | | | | | | | | | | | | | | | |
| S06896A | | | | | Other specified intracranial injury with loss of consciousness greater than 24 hours without return to pre-existing conscious level with patient surviving, initial encounter | | | | | | | | | | | | | | | | | | | | | | | | |
| S06896D | | | | | Other specified intracranial injury with loss of consciousness greater than 24 hours without return to pre-existing conscious level with patient surviving, subsequent encounter | | | | | | | | | | | | | | | | | | | | | | | | |
| S06896S | | | | | Other specified intracranial injury with loss of consciousness greater than 24 hours without return to pre-existing conscious level with patient surviving, sequela | | | | | | | | | | | | | | | | | | | | | | | | |
| S06897A | | | | | Other specified intracranial injury with loss of consciousness of any duration with death due to brain injury prior to regaining consciousness, initial encounter | | | | | | | | | | | | | | | | | | | | | | | | |
| S06898A | | | | | Other specified intracranial injury with loss of consciousness of any duration with death due to other cause prior to regaining consciousness, initial encounter | | | | | | | | | | | | | | | | | | | | | | | | |
| S06899A | | | | | Other specified intracranial injury with loss of consciousness of unspecified duration, initial encounter | | | | | | | | | | | | | | | | | | | | | | | | |
| S06899D | | | | | Other specified intracranial injury with loss of consciousness of unspecified duration, subsequent encounter | | | | | | | | | | | | | | | | | | | | | | | | |
| S06899S | | | | | Other specified intracranial injury with loss of consciousness of unspecified duration, sequela | | | | | | | | | | | | | | | | | | | | | | | | |
| S069X0A | | | | | Unspecified intracranial injury without loss of consciousness, initial encounter | | | | | | | | | | | | | | | | | | | | | | | | |
| S069X0D | | | | | Unspecified intracranial injury without loss of consciousness, subsequent encounter | | | | | | | | | | | | | | | | | | | | | | | | |
| S069X0S | | | | | Unspecified intracranial injury without loss of consciousness, sequela | | | | | | | | | | | | | | | | | | | | | | | | |
| S069X1A | | | | | Unspecified intracranial injury with loss of consciousness of 30 minutes or less, initial encounter | | | | | | | | | | | | | | | | | | | | | | | | |
| S069X1D | | | | | Unspecified intracranial injury with loss of consciousness of 30 minutes or less, subsequent encounter | | | | | | | | | | | | | | | | | | | | | | | | |
| S069X1S | | | | | Unspecified intracranial injury with loss of consciousness of 30 minutes or less, sequela | | | | | | | | | | | | | | | | | | | | | | | | |
| S069X2A | | | | | Unspecified intracranial injury with loss of consciousness of 31 minutes to 59 minutes, initial encounter | | | | | | | | | | | | | | | | | | | | | | | | |
| S069X2D | | | | | Unspecified intracranial injury with loss of consciousness of 31 minutes to 59 minutes, subsequent encounter | | | | | | | | | | | | | | | | | | | | | | | | |
| S069X2S | | | | | Unspecified intracranial injury with loss of consciousness of 31 minutes to 59 minutes, sequela | | | | | | | | | | | | | | | | | | | | | | | | |
| S069X3A | | | | | Unspecified intracranial injury with loss of consciousness of 1 hour to 5 hours 59 minutes, initial encounter | | | | | | | | | | | | | | | | | | | | | | | | |
| S069X3D | | | | | Unspecified intracranial injury with loss of consciousness of 1 hour to 5 hours 59 minutes, subsequent encounter | | | | | | | | | | | | | | | | | | | | | | | | |
| S069X3S | | | | | Unspecified intracranial injury with loss of consciousness of 1 hour to 5 hours 59 minutes, sequela | | | | | | | | | | | | | | | | | | | | | | | | |
| S069X4A | | | | | Unspecified intracranial injury with loss of consciousness of 6 hours to 24 hours, initial encounter | | | | | | | | | | | | | | | | | | | | | | | | |
| S069X4D | | | | | Unspecified intracranial injury with loss of consciousness of 6 hours to 24 hours, subsequent encounter | | | | | | | | | | | | | | | | | | | | | | | | |
| S069X4S | | | | | Unspecified intracranial injury with loss of consciousness of 6 hours to 24 hours, sequela | | | | | | | | | | | | | | | | | | | | | | | | |
| S069X5A | | | | | Unspecified intracranial injury with loss of consciousness greater than 24 hours with return to pre-existing conscious level, initial encounter | | | | | | | | | | | | | | | | | | | | | | | | |
| S069X5D | | | | | Unspecified intracranial injury with loss of consciousness greater than 24 hours with return to pre-existing conscious level, subsequent encounter | | | | | | | | | | | | | | | | | | | | | | | | |
| S069X5S | | | | | Unspecified intracranial injury with loss of consciousness greater than 24 hours with return to pre-existing conscious level, sequela | | | | | | | | | | | | | | | | | | | | | | | | |
| S069X6A | | | | | Unspecified intracranial injury with loss of consciousness greater than 24 hours without return to pre-existing conscious level with patient surviving, initial encounter | | | | | | | | | | | | | | | | | | | | | | | | |
| S069X6D | | | | | Unspecified intracranial injury with loss of consciousness greater than 24 hours without return to pre-existing conscious level with patient surviving, subsequent encounter | | | | | | | | | | | | | | | | | | | | | | | | |
| S069X6S | | | | | Unspecified intracranial injury with loss of consciousness greater than 24 hours without return to pre-existing conscious level with patient surviving, sequela | | | | | | | | | | | | | | | | | | | | | | | | |
| S069X7A | | | | | Unspecified intracranial injury with loss of consciousness of any duration with death due to brain injury prior to regaining consciousness, initial encounter | | | | | | | | | | | | | | | | | | | | | | | | |
| S069X8A | | | | | Unspecified intracranial injury with loss of consciousness of any duration with death due to other cause prior to regaining consciousness, initial encounter | | | | | | | | | | | | | | | | | | | | | | | | |
| S069X9A | | | | | Unspecified intracranial injury with loss of consciousness of unspecified duration, initial encounter | | | | | | | | | | | | | | | | | | | | | | | | |
| S069X9D | | | | | Unspecified intracranial injury with loss of consciousness of unspecified duration, subsequent encounter | | | | | | | | | | | | | | | | | | | | | | | | |
| S069X9S | | | | | Unspecified intracranial injury with loss of consciousness of unspecified duration, sequela | | | | | | | | | | | | | | | | | | | | | | | | |
| 430 | | | | | Subarachnoid hemorrhage | | | | | |  |  |  |  | |  | |  | | | |  | | |  | |  |  |  |
| 431 | | | | | Intracerebral hemorrhage | | | | | |  |  |  |  | |  | |  | | | |  | | |  | |  |  |  |
| 5430 | | | | | Subarachnoid hemorrhage | | | | | | | | | | | | | | | |  | | |  | | | |  |  |
| 4329 | | | | | Unspecified intracranial hemorrhage | | | | | | | | | | | | | | | |  | | |  | | | |  |  |
| 4321 | | | | | Subdural hemorrhage | | | | | | | | | | | |  | | | |  | | |  | | | |  |  |
| 4320 | | | | | Nontraumatic extradural hemorrhage | | | | | | | | | |  | |  | | | |  | | |  | | | |  |  |
| 7670 | | | | | Subdural and cerebral hemorrhage | | | | | | | | | |  | |  | | | |  | | |  | | | |  |  |
| 43411 | | | | | Cerebral embolism with cerebral infarction | | | | | | | | | | | | | | | | | | |  | | | |  |  |
| 4376 | | | | | Nonpyogenic thrombosis of intracranial venous sinus | | | | | | | | | | | | | | | | | | |  | | | |  |  |
| V1254 | | | | | Personal history of transient ischemic attack (TIA), and cerebral infarction without residual deficits | | | | | | | | | | | | | | | | | | |  | | | |  |  |
| I6000 | | | | | Nontraumatic subarachnoid hemorrhage from unspecified carotid siphon and bifurcation | | | | | | | | | | | | | | | | | | |  | | | |  |  |
| I6001 | | | | | Nontraumatic subarachnoid hemorrhage from right carotid siphon and bifurcation | | | | | | | | | | | | | | | | | | |  | | | |  |  |
| I6002 | | | | | Nontraumatic subarachnoid hemorrhage from left carotid siphon and bifurcation | | | | | | | | | | | | | | | | | | |  | | | |  |  |
| I6010 | | | | | Nontraumatic subarachnoid hemorrhage from unspecified middle cerebral artery | | | | | | | | | | | | | | | | | | |  | | | |  |  |
| I6011 | | | | | Nontraumatic subarachnoid hemorrhage from right middle cerebral artery | | | | | | | | | | | | | | | | | | |  | | | |  |  |
| I6012 | | | | | Nontraumatic subarachnoid hemorrhage from left middle cerebral artery | | | | | | | | | | | | | | | | | | |  | | | |  |  |
| I602 | | | | | Nontraumatic subarachnoid hemorrhage from anterior communicating artery | | | | | | | | | | | | | | | | | | |  | | | |  |  |
| I6030 | | | | | Nontraumatic subarachnoid hemorrhage from unspecified posterior communicating artery | | | | | | | | | | | | | | | | | | |  | | | |  |  |
| I6031 | | | | | Nontraumatic subarachnoid hemorrhage from right posterior communicating artery | | | | | | | | | | | | | | | | | | |  | | | |  |  |
| I6032 | | | | | Nontraumatic subarachnoid hemorrhage from left posterior communicating artery | | | | | | | | | | | | | | | | | | |  | | | |  |  |
| I604 | | | | | Nontraumatic subarachnoid hemorrhage from basilar artery | | | | | | | | | | | | | | | | | | |  | | | |  |  |
| I6050 | | | | | Nontraumatic subarachnoid hemorrhage from unspecified vertebral artery | | | | | | | | | | | | | | | | | | |  | | | |  |  |
| I6051 | | | | | Nontraumatic subarachnoid hemorrhage from right vertebral artery | | | | | | | | | | | | | | | | | | |  | | | |  |  |
| I6052 | | | | | Nontraumatic subarachnoid hemorrhage from left vertebral artery | | | | | | | | | | | | | | | | | | |  | | | |  |  |
| I606 | | | | | Nontraumatic subarachnoid hemorrhage from other intracranial arteries | | | | | | | | | | | | | | | | | | |  | | | |  |  |
| I607 | | | | | Nontraumatic subarachnoid hemorrhage from unspecified intracranial artery | | | | | | | | | | | | | | | | | | |  | | | |  |  |
| I608 | | | | | Other nontraumatic subarachnoid hemorrhage | | | | | | | | | | | | | | | | | | |  | | | |  |  |
| I609 | | | | | Nontraumatic subarachnoid hemorrhage, unspecified | | | | | | | | | | | | | | | | | | |  | | | |  |  |
| I610 | | | | | Nontraumatic intracerebral hemorrhage in hemisphere, subcortical | | | | | | | | | | | | | | | | | | |  | | | |  |  |
| I611 | | | | | Nontraumatic intracerebral hemorrhage in hemisphere, cortical | | | | | | | | | | | | | | | | | | |  | | | |  |  |
| I612 | | | | | Nontraumatic intracerebral hemorrhage in hemisphere, unspecified | | | | | | | | | | | | | | | | | | |  | | | |  |  |
| I613 | | | | | Nontraumatic intracerebral hemorrhage in brain stem | | | | | | | | | | | | | | | | | | |  | | | |  |  |
| I614 | | | | | Nontraumatic intracerebral hemorrhage in cerebellum | | | | | | | | | | | | | | | | | | |  | | | |  |  |
| I615 | | | | | Nontraumatic intracerebral hemorrhage, intraventricular | | | | | | | | | | | | | | | | | | |  | | | |  |  |
| I616 | | | | | Nontraumatic intracerebral hemorrhage, multiple localized | | | | | | | | | | | | | | | | | | |  | | | |  |  |
| I618 | | | | | Other nontraumatic intracerebral hemorrhage | | | | | | | | | | | | | | | | | | |  | | | |  |  |
| I619 | | | | | Nontraumatic intracerebral hemorrhage, unspecified | | | | | | | | | | | | | | | | | | |  | | | |  |  |
| I6200 | | | | | Nontraumatic subdural hemorrhage, unspecified | | | | | | | | | | | | | | | | | | |  | | | |  |  |
| I6201 | | | | | Nontraumatic acute subdural hemorrhage | | | | | | | | | | | | | | | | | | |  | | | |  |  |
| I6202 | | | | | Nontraumatic subacute subdural hemorrhage | | | | | | | | | | | | | | | | | | |  | | | |  |  |
| I6203 | | | | | Nontraumatic chronic subdural hemorrhage | | | | | | | | | | | | | | | | | | |  | | | |  |  |
| I621 | | | | | Nontraumatic extradural hemorrhage | | | | | | | | | | | | | | | | | | |  | | | |  |  |
| I629 | | | | | Nontraumatic intracranial hemorrhage, unspecified | | | | | | | | | | | | | | | | | | |  | | | |  |  |
| I6300 | | | | | Cerebral infarction due to thrombosis of unspecified precerebral artery | | | | | | | | | | | | | | | | | | |  | | | |  |  |
| I63011 | | | | | Cerebral infarction due to thrombosis of right vertebral artery | | | | | | | | | | | | | | | | | | |  | | | |  |  |
| I63012 | | | | | Cerebral infarction due to thrombosis of left vertebral artery | | | | | | | | | | | | | | | | | | |  | | | |  |  |
| I63013 | | | | | Cerebral infarction due to thrombosis of bilateral vertebral arteries | | | | | | | | | | | | | | | | | | |  | | | |  |  |
| I63019 | | | | | Cerebral infarction due to thrombosis of unspecified vertebral artery | | | | | | | | | | | | | | | | | | |  | | | |  |  |
| I6302 | | | | | Cerebral infarction due to thrombosis of basilar artery | | | | | | | | | | | | | | | | | | |  | | | |  |  |
| I63031 | | | | | Cerebral infarction due to thrombosis of right carotid artery | | | | | | | | | | | | | | | | | | |  | | | |  |  |
| I63032 | | | | | Cerebral infarction due to thrombosis of left carotid artery | | | | | | | | | | | | | | | | | | |  | | | |  |  |
| I63033 | | | | | Cerebral infarction due to thrombosis of bilateral carotid arteries | | | | | | | | | | | | | | | | | | |  | | | |  |  |
| I63039 | | | | | Cerebral infarction due to thrombosis of unspecified carotid artery | | | | | | | | | | | | | | | | | | |  | | | |  |  |
| I6309 | | | | | Cerebral infarction due to thrombosis of other precerebral artery | | | | | | | | | | | | | | | | | | |  | | | |  |  |
| I6310 | | | | | Cerebral infarction due to embolism of unspecified precerebral artery | | | | | | | | | | | | | | | | | | |  | | | |  |  |
| I63111 | | | | | Cerebral infarction due to embolism of right vertebral artery | | | | | | | | | | | | | | | | | | |  | | | |  |  |
| I63112 | | | | | Cerebral infarction due to embolism of left vertebral artery | | | | | | | | | | | | | | | | | | |  | | | |  |  |
| I63113 | | | | | Cerebral infarction due to embolism of bilateral vertebral arteries | | | | | | | | | | | | | | | | | | |  | | | |  |  |
| I63119 | | | | | Cerebral infarction due to embolism of unspecified vertebral artery | | | | | | | | | | | | | | | | | | |  | | | |  |  |
| I6312 | | | | | Cerebral infarction due to embolism of basilar artery | | | | | | | | | | | | | | | | | | |  | | | |  |  |
| I63131 | | | | | Cerebral infarction due to embolism of right carotid artery | | | | | | | | | | | | | | | | | | |  | | | |  |  |
| I63132 | | | | | Cerebral infarction due to embolism of left carotid artery | | | | | | | | | | | | | | | | | | |  | | | |  |  |
| I63133 | | | | | Cerebral infarction due to embolism of bilateral carotid arteries | | | | | | | | | | | | | | | | | | |  | | | |  |  |
| I63139 | | | | | Cerebral infarction due to embolism of unspecified carotid artery | | | | | | | | | | | | | | | | | | |  | | | |  |  |
| I6319 | | | | | Cerebral infarction due to embolism of other precerebral artery | | | | | | | | | | | | | | | | | | |  | | | |  |  |
| I6320 | | | | | Cerebral infarction due to unspecified occlusion or stenosis of unspecified precerebral arteries | | | | | | | | | | | | | | | | | | |  | | | |  |  |
| I63211 | | | | | Cerebral infarction due to unspecified occlusion or stenosis of right vertebral artery | | | | | | | | | | | | | | | | | | |  | | | |  |  |
| I63212 | | | | | Cerebral infarction due to unspecified occlusion or stenosis of left vertebral artery | | | | | | | | | | | | | | | | | | |  | | | |  |  |
| I63213 | | | | | Cerebral infarction due to unspecified occlusion or stenosis of bilateral vertebral arteries | | | | | | | | | | | | | | | | | | |  | | | |  |  |
| I63219 | | | | | Cerebral infarction due to unspecified occlusion or stenosis of unspecified vertebral artery | | | | | | | | | | | | | | | | | | |  | | | |  |  |
| I6322 | | | | | Cerebral infarction due to unspecified occlusion or stenosis of basilar artery | | | | | | | | | | | | | | | | | | |  | | | |  |  |
| I63231 | | | | | Cerebral infarction due to unspecified occlusion or stenosis of right carotid arteries | | | | | | | | | | | | | | | | | | |  | | | |  |  |
| I63232 | | | | | Cerebral infarction due to unspecified occlusion or stenosis of left carotid arteries | | | | | | | | | | | | | | | | | | |  | | | |  |  |
| I63233 | | | | | Cerebral infarction due to unspecified occlusion or stenosis of bilateral carotid arteries | | | | | | | | | | | | | | | | | | |  | | | |  |  |
| I63239 | | | | | Cerebral infarction due to unspecified occlusion or stenosis of unspecified carotid artery | | | | | | | | | | | | | | | | | | |  | | | |  |  |
| I6329 | | | | | Cerebral infarction due to unspecified occlusion or stenosis of other precerebral arteries | | | | | | | | | | | | | | | | | | |  | | | |  |  |
| I6330 | | | | | Cerebral infarction due to thrombosis of unspecified cerebral artery | | | | | | | | | | | | | | | | | | |  | | | |  |  |
| I63311 | | | | | Cerebral infarction due to thrombosis of right middle cerebral artery | | | | | | | | | | | | | | | | | | |  | | | |  |  |
| I63312 | | | | | Cerebral infarction due to thrombosis of left middle cerebral artery | | | | | | | | | | | | | | | | | | |  | | | |  |  |
| I63313 | | | | | Cerebral infarction due to thrombosis of bilateral middle cerebral arteries | | | | | | | | | | | | | | | | | | |  | | | |  |  |
| I63319 | | | | | Cerebral infarction due to thrombosis of unspecified middle cerebral artery | | | | | | | | | | | | | | | | | | |  | | | |  |  |
| I63321 | | | | | Cerebral infarction due to thrombosis of right anterior cerebral artery | | | | | | | | | | | | | | | | | | |  | | | |  |  |
| I63322 | | | | | Cerebral infarction due to thrombosis of left anterior cerebral artery | | | | | | | | | | | | | | | | | | |  | | | |  |  |
| I63323 | | | | | Cerebral infarction due to thrombosis of bilateral anterior cerebral arteries | | | | | | | | | | | | | | | | | | |  | | | |  |  |
| I63329 | | | | | Cerebral infarction due to thrombosis of unspecified anterior cerebral artery | | | | | | | | | | | | | | | | | | |  | | | |  |  |
| I63331 | | | | | Cerebral infarction due to thrombosis of right posterior cerebral artery | | | | | | | | | | | | | | | | | | |  | | | |  |  |
| I63332 | | | | | Cerebral infarction due to thrombosis of left posterior cerebral artery | | | | | | | | | | | | | | | | | | |  | | | |  |  |
| I63333 | | | | | Cerebral infarction due to thrombosis of bilateral posterior cerebral arteries | | | | | | | | | | | | | | | | | | |  | | | |  |  |
| I63339 | | | | | Cerebral infarction due to thrombosis of unspecified posterior cerebral artery | | | | | | | | | | | | | | | | | | |  | | | |  |  |
| I63341 | | | | | Cerebral infarction due to thrombosis of right cerebellar artery | | | | | | | | | | | | | | | | | | |  | | | |  |  |
| I63342 | | | | | Cerebral infarction due to thrombosis of left cerebellar artery | | | | | | | | | | | | | | | | | | |  | | | |  |  |
| I63343 | | | | | Cerebral infarction due to thrombosis of bilateral cerebellar arteries | | | | | | | | | | | | | | | | | | |  | | | |  |  |
| I63349 | | | | | Cerebral infarction due to thrombosis of unspecified cerebellar artery | | | | | | | | | | | | | | | | | | |  | | | |  |  |
| I6339 | | | | | Cerebral infarction due to thrombosis of other cerebral artery | | | | | | | | | | | | | | | | | | |  | | | |  |  |
| I6340 | | | | | Cerebral infarction due to embolism of unspecified cerebral artery | | | | | | | | | | | | | | | | | | |  | | | |  |  |
| I63411 | | | | | Cerebral infarction due to embolism of right middle cerebral artery | | | | | | | | | | | | | | | | | | |  | | | |  |  |
| I63412 | | | | | Cerebral infarction due to embolism of left middle cerebral artery | | | | | | | | | | | | | | | | | | |  | | | |  |  |
| I63413 | | | | | Cerebral infarction due to embolism of bilateral middle cerebral arteries | | | | | | | | | | | | | | | | | | |  | | | |  |  |
| I63419 | | | | | Cerebral infarction due to embolism of unspecified middle cerebral artery | | | | | | | | | | | | | | | | | | |  | | | |  |  |
| I63421 | | | | | Cerebral infarction due to embolism of right anterior cerebral artery | | | | | | | | | | | | | | | | | | |  | | | |  |  |
| I63422 | | | | | Cerebral infarction due to embolism of left anterior cerebral artery | | | | | | | | | | | | | | | | | | |  | | | |  |  |
| I63423 | | | | | Cerebral infarction due to embolism of bilateral anterior cerebral arteries | | | | | | | | | | | | | | | | | | |  | | | |  |  |
| I63429 | | | | | Cerebral infarction due to embolism of unspecified anterior cerebral artery | | | | | | | | | | | | | | | | | | |  | | | |  |  |
| I63431 | | | | | Cerebral infarction due to embolism of right posterior cerebral artery | | | | | | | | | | | | | | | | | | |  | | | |  |  |
| I63432 | | | | | Cerebral infarction due to embolism of left posterior cerebral artery | | | | | | | | | | | | | | | | | | |  | | | |  |  |
| I63433 | | | | | Cerebral infarction due to embolism of bilateral posterior cerebral arteries | | | | | | | | | | | | | | | | | | |  | | | |  |  |
| I63439 | | | | | Cerebral infarction due to embolism of unspecified posterior cerebral artery | | | | | | | | | | | | | | | | | | |  | | | |  |  |
| I63441 | | | | | Cerebral infarction due to embolism of right cerebellar artery | | | | | | | | | | | | | | | | | | |  | | | |  |  |
| I63442 | | | | | Cerebral infarction due to embolism of left cerebellar artery | | | | | | | | | | | | | | | | | | |  | | | |  |  |
| I63443 | | | | | Cerebral infarction due to embolism of bilateral cerebellar arteries | | | | | | | | | | | | | | | | | | |  | | | |  |  |
| I63449 | | | | | Cerebral infarction due to embolism of unspecified cerebellar artery | | | | | | | | | | | | | | | | | | |  | | | |  |  |
| I6349 | | | | | Cerebral infarction due to embolism of other cerebral artery | | | | | | | | | | | | | | | | | | |  | | | |  |  |
| I6350 | | | | | Cerebral infarction due to unspecified occlusion or stenosis of unspecified cerebral artery | | | | | | | | | | | | | | | | | | |  | | | |  |  |
| I63511 | | | | | Cerebral infarction due to unspecified occlusion or stenosis of right middle cerebral artery | | | | | | | | | | | | | | | | | | |  | | | |  |  |
| I63512 | | | | | Cerebral infarction due to unspecified occlusion or stenosis of left middle cerebral artery | | | | | | | | | | | | | | | | | | |  | | | |  |  |
| I63513 | | | | | Cerebral infarction due to unspecified occlusion or stenosis of bilateral middle cerebral arteries | | | | | | | | | | | | | | | | | | |  | | | |  |  |
| I63519 | | | | | Cerebral infarction due to unspecified occlusion or stenosis of unspecified middle cerebral artery | | | | | | | | | | | | | | | | | | |  | | | |  |  |
| I63521 | | | | | Cerebral infarction due to unspecified occlusion or stenosis of right anterior cerebral artery | | | | | | | | | | | | | | | | | | |  | | | |  |  |
| I63522 | | | | | Cerebral infarction due to unspecified occlusion or stenosis of left anterior cerebral artery | | | | | | | | | | | | | | | | | | |  | | | |  |  |
| I63523 | | | | | Cerebral infarction due to unspecified occlusion or stenosis of bilateral anterior cerebral arteries | | | | | | | | | | | | | | | | | | |  | | | |  |  |
| I63529 | | | | | Cerebral infarction due to unspecified occlusion or stenosis of unspecified anterior cerebral artery | | | | | | | | | | | | | | | | | | |  | | | |  |  |
| I63531 | | | | | Cerebral infarction due to unspecified occlusion or stenosis of right posterior cerebral artery | | | | | | | | | | | | | | | | | | |  | | | |  |  |
| I63532 | | | | | Cerebral infarction due to unspecified occlusion or stenosis of left posterior cerebral artery | | | | | | | | | | | | | | | | | | |  | | | |  |  |
| I63533 | | | | | Cerebral infarction due to unspecified occlusion or stenosis of bilateral posterior cerebral arteries | | | | | | | | | | | | | | | | | | |  | | | |  |  |
| I63539 | | | | | Cerebral infarction due to unspecified occlusion or stenosis of unspecified posterior cerebral artery | | | | | | | | | | | | | | | | | | |  | | | |  |  |
| I63541 | | | | | Cerebral infarction due to unspecified occlusion or stenosis of right cerebellar artery | | | | | | | | | | | | | | | | | | |  | | | |  |  |
| I63542 | | | | | Cerebral infarction due to unspecified occlusion or stenosis of left cerebellar artery | | | | | | | | | | | | | | | | | | |  | | | |  |  |
| I63543 | | | | | Cerebral infarction due to unspecified occlusion or stenosis of bilateral cerebellar arteries | | | | | | | | | | | | | | | | | | |  | | | |  |  |
| I63549 | | | | | Cerebral infarction due to unspecified occlusion or stenosis of unspecified cerebellar artery | | | | | | | | | | | | | | | | | | |  | | | |  |  |
| I6359 | | | | | Cerebral infarction due to unspecified occlusion or stenosis of other cerebral artery | | | | | | | | | | | | | | | | | | |  | | | |  |  |
| I636 | | | | | Cerebral infarction due to cerebral venous thrombosis, nonpyogenic | | | | | | | | | | | | | | | | | | |  | | | |  |  |
| I6381 | | | | | Other cerebral infarction due to occlusion or stenosis of small artery | | | | | | | | | | | | | | | | | | |  | | | |  |  |
| I6389 | | | | | Other cerebral infarction | | | | | | | | | | | | | | | | | | |  | | | |  |  |
| I639 | | | | | Cerebral infarction, unspecified | | | | | | | | | | | | | | | | | | |  | | | |  |  |
| I6501 | | | | | Occlusion and stenosis of right vertebral artery | | | | | | | | | | | | | | | | | | |  | | | |  |  |
| I6502 | | | | | Occlusion and stenosis of left vertebral artery | | | | | | | | | | | | | | | | | | |  | | | |  |  |
| I6503 | | | | | Occlusion and stenosis of bilateral vertebral arteries | | | | | | | | | | | | | | | | | | |  | | | |  |  |
| I6509 | | | | | Occlusion and stenosis of unspecified vertebral artery | | | | | | | | | | | | | | | | | | |  | | | |  |  |
| I651 | | | | | Occlusion and stenosis of basilar artery | | | | | | | | | | | | | | | | | | |  | | | |  |  |
| I6521 | | | | | Occlusion and stenosis of right carotid artery | | | | | | | | | | | | | | | | | | |  | | | |  |  |
| I6522 | | | | | Occlusion and stenosis of left carotid artery | | | | | | | | | | | | | | | | | | |  | | | |  |  |
| I6523 | | | | | Occlusion and stenosis of bilateral carotid arteries | | | | | | | | | | | | | | | | | | |  | | | |  |  |
| I6529 | | | | | Occlusion and stenosis of unspecified carotid artery | | | | | | | | | | | | | | | | | | |  | | | |  |  |
| I658 | | | | | Occlusion and stenosis of other precerebral arteries | | | | | | | | | | | | | | | | | | |  | | | |  |  |
| I659 | | | | | Occlusion and stenosis of unspecified precerebral artery | | | | | | | | | | | | | | | | | | |  | | | |  |  |
| I6601 | | | | | Occlusion and stenosis of right middle cerebral artery | | | | | | | | | | | | | | | | | | |  | | | |  |  |
| I6602 | | | | | Occlusion and stenosis of left middle cerebral artery | | | | | | | | | | | | | | | | | | |  | | | |  |  |
| I6603 | | | | | Occlusion and stenosis of bilateral middle cerebral arteries | | | | | | | | | | | | | | | | | | |  | | | |  |  |
| I6609 | | | | | Occlusion and stenosis of unspecified middle cerebral artery | | | | | | | | | | | | | | | | | | |  | | | |  |  |
| I6611 | | | | | Occlusion and stenosis of right anterior cerebral artery | | | | | | | | | | | | | | | | | | |  | | | |  |  |
| I6612 | | | | | Occlusion and stenosis of left anterior cerebral artery | | | | | | | | | | | | | | | | | | |  | | | |  |  |
| I6613 | | | | | Occlusion and stenosis of bilateral anterior cerebral arteries | | | | | | | | | | | | | | | | | | |  | | | |  |  |
| I6619 | | | | | Occlusion and stenosis of unspecified anterior cerebral artery | | | | | | | | | | | | | | | | | | |  | | | |  |  |
| I6621 | | | | | Occlusion and stenosis of right posterior cerebral artery | | | | | | | | | | | | | | | | | | |  | | | |  |  |
| I6622 | | | | | Occlusion and stenosis of left posterior cerebral artery | | | | | | | | | | | | | | | | | | |  | | | |  |  |
| I6623 | | | | | Occlusion and stenosis of bilateral posterior cerebral arteries | | | | | | | | | | | | | | | | | | |  | | | |  |  |
| I6629 | | | | | Occlusion and stenosis of unspecified posterior cerebral artery | | | | | | | | | | | | | | | | | | |  | | | |  |  |
| I663 | | | | | Occlusion and stenosis of cerebellar arteries | | | | | | | | | | | | | | | | | | |  | | | |  |  |
| I668 | | | | | Occlusion and stenosis of other cerebral arteries | | | | | | | | | | | | | | | | | | |  | | | |  |  |
| I669 | | | | | Occlusion and stenosis of unspecified cerebral artery | | | | | | | | | | | | | | | | | | |  | | | |  |  |
| I670 | | | | | Dissection of cerebral arteries, nonruptured | | | | | | | | | | | | | | | | | | |  | | | |  |  |
| I676 | | | | | Nonpyogenic thrombosis of intracranial venous system | | | | | | | | | | | | | | | | | | |  | | | |  |  |
| I677 | | | | | Cerebral arteritis, not elsewhere classified | | | | | | | | | | | | | | | | | | |  | | | |  |  |
| I6781 | | | | | Acute cerebrovascular insufficiency | | | | | | | | | | | | | | | | | | |  | | | |  |  |
| I6782 | | | | | Cerebral ischemia | | | | | | | | | | | | | | | | | | |  | | | |  |  |
| I6783 | | | | | Posterior reversible encephalopathy syndrome | | | | | | | | | | | | | | | | | | |  | | | |  |  |
| I67841 | | | | | Reversible cerebrovascular vasoconstriction syndrome | | | | | | | | | | | | | | | | | | |  | | | |  |  |
| I67848 | | | | | Other cerebrovascular vasospasm and vasoconstriction | | | | | | | | | | | | | | | | | | |  | | | |  |  |
| I67850 | | | | | Cerebral autosomal dominant arteriopathy with subcortical infarcts and leukoencephalopathy | | | | | | | | | | | | | | | | | | |  | | | |  |  |
| I67858 | | | | | Other hereditary cerebrovascular disease | | | | | | | | | | | | | | | | | | |  | | | |  |  |
| I6789 | | | | | Other cerebrovascular disease | | | | | | | | | | | | | | | | | | |  | | | |  |  |
| I679 | | | | | Cerebrovascular disease, unspecified | | | | | | | | | | | | | | | | | | |  | | | |  |  |
| I680 | | | | | Cerebral amyloid angiopathy | | | | | | | | | | | | | | | | | | |  | | | |  |  |
| I682 | | | | | Cerebral arteritis in other diseases classified elsewhere | | | | | | | | | | | | | | | | | | |  | | | |  |  |
| I688 | | | | | Other cerebrovascular disorders in diseases classified elsewhere | | | | | | | | | | | | | | | | | | |  | | | |  |  |
| I6900 | | | | | Unspecified sequelae of nontraumatic subarachnoid hemorrhage | | | | | | | | | | | | | | | | | | |  | | | |  |  |
| I69010 | | | | | Attention and concentration deficit following nontraumatic subarachnoid hemorrhage | | | | | | | | | | | | | | | | | | |  | | | |  |  |
| I69011 | | | | | Memory deficit following nontraumatic subarachnoid hemorrhage | | | | | | | | | | | | | | | | | | |  | | | |  |  |
| I69012 | | | | | Visuospatial deficit and spatial neglect following nontraumatic subarachnoid hemorrhage | | | | | | | | | | | | | | | | | | |  | | | |  |  |
| I69013 | | | | | Psychomotor deficit following nontraumatic subarachnoid hemorrhage | | | | | | | | | | | | | | | | | | |  | | | |  |  |
| I69014 | | | | | Frontal lobe and executive function deficit following nontraumatic subarachnoid hemorrhage | | | | | | | | | | | | | | | | | | |  | | | |  |  |
| I69015 | | | | | Cognitive social or emotional deficit following nontraumatic subarachnoid hemorrhage | | | | | | | | | | | | | | | | | | |  | | | |  |  |
| I69018 | | | | | Other symptoms and signs involving cognitive functions following nontraumatic subarachnoid hemorrhage | | | | | | | | | | | | | | | | | | |  | | | |  |  |
| I69019 | | | | | Unspecified symptoms and signs involving cognitive functions following nontraumatic subarachnoid hemorrhage | | | | | | | | | | | | | | | | | | |  | | | |  |  |
| I69020 | | | | | Aphasia following nontraumatic subarachnoid hemorrhage | | | | | | | | | | | | | | | | | | |  | | | |  |  |
| I69021 | | | | | Dysphasia following nontraumatic subarachnoid hemorrhage | | | | | | | | | | | | | | | | | | |  | | | |  |  |
| I69022 | | | | | Dysarthria following nontraumatic subarachnoid hemorrhage | | | | | | | | | | | | | | | | | | |  | | | |  |  |
| I69023 | | | | | Fluency disorder following nontraumatic subarachnoid hemorrhage | | | | | | | | | | | | | | | | | | |  | | | |  |  |
| I69028 | | | | | Other speech and language deficits following nontraumatic subarachnoid hemorrhage | | | | | | | | | | | | | | | | | | |  | | | |  |  |
| I69031 | | | | | Monoplegia of upper limb following nontraumatic subarachnoid hemorrhage affecting right dominant side | | | | | | | | | | | | | | | | | | |  | | | |  |  |
| I69032 | | | | | Monoplegia of upper limb following nontraumatic subarachnoid hemorrhage affecting left dominant side | | | | | | | | | | | | | | | | | | |  | | | |  |  |
| I69033 | | | | | Monoplegia of upper limb following nontraumatic subarachnoid hemorrhage affecting right non-dominant side | | | | | | | | | | | | | | | | | | |  | | | |  |  |
| I69034 | | | | | Monoplegia of upper limb following nontraumatic subarachnoid hemorrhage affecting left non-dominant side | | | | | | | | | | | | | | | | | | |  | | | |  |  |
| I69039 | | | | | Monoplegia of upper limb following nontraumatic subarachnoid hemorrhage affecting unspecified side | | | | | | | | | | | | | | | | | | |  | | | |  |  |
| I69041 | | | | | Monoplegia of lower limb following nontraumatic subarachnoid hemorrhage affecting right dominant side | | | | | | | | | | | | | | | | | | |  | | | |  |  |
| I69042 | | | | | Monoplegia of lower limb following nontraumatic subarachnoid hemorrhage affecting left dominant side | | | | | | | | | | | | | | | | | | |  | | | |  |  |
| I69043 | | | | | Monoplegia of lower limb following nontraumatic subarachnoid hemorrhage affecting right non-dominant side | | | | | | | | | | | | | | | | | | |  | | | |  |  |
| I69044 | | | | | Monoplegia of lower limb following nontraumatic subarachnoid hemorrhage affecting left non-dominant side | | | | | | | | | | | | | | | | | | |  | | | |  |  |
| I69049 | | | | | Monoplegia of lower limb following nontraumatic subarachnoid hemorrhage affecting unspecified side | | | | | | | | | | | | | | | | | | |  | | | |  |  |
| I69051 | | | | | Hemiplegia and hemiparesis following nontraumatic subarachnoid hemorrhage affecting right dominant side | | | | | | | | | | | | | | | | | | |  | | | |  |  |
| I69052 | | | | | Hemiplegia and hemiparesis following nontraumatic subarachnoid hemorrhage affecting left dominant side | | | | | | | | | | | | | | | | | | |  | | | |  |  |
| I69053 | | | | | Hemiplegia and hemiparesis following nontraumatic subarachnoid hemorrhage affecting right non-dominant side | | | | | | | | | | | | | | | | | | |  | | | |  |  |
| I69054 | | | | | Hemiplegia and hemiparesis following nontraumatic subarachnoid hemorrhage affecting left non-dominant side | | | | | | | | | | | | | | | | | | |  | | | |  |  |
| I69059 | | | | | Hemiplegia and hemiparesis following nontraumatic subarachnoid hemorrhage affecting unspecified side | | | | | | | | | | | | | | | | | | |  | | | |  |  |
| I69061 | | | | | Other paralytic syndrome following nontraumatic subarachnoid hemorrhage affecting right dominant side | | | | | | | | | | | | | | | | | | |  | | | |  |  |
| I69062 | | | | | Other paralytic syndrome following nontraumatic subarachnoid hemorrhage affecting left dominant side | | | | | | | | | | | | | | | | | | |  | | | |  |  |
| I69063 | | | | | Other paralytic syndrome following nontraumatic subarachnoid hemorrhage affecting right non-dominant side | | | | | | | | | | | | | | | | | | |  | | | |  |  |
| I69064 | | | | | Other paralytic syndrome following nontraumatic subarachnoid hemorrhage affecting left non-dominant side | | | | | | | | | | | | | | | | | | |  | | | |  |  |
| I69065 | | | | | Other paralytic syndrome following nontraumatic subarachnoid hemorrhage, bilateral | | | | | | | | | | | | | | | | | | |  | | | |  |  |
| I69069 | | | | | Other paralytic syndrome following nontraumatic subarachnoid hemorrhage affecting unspecified side | | | | | | | | | | | | | | | | | | |  | | | |  |  |
| I69090 | | | | | Apraxia following nontraumatic subarachnoid hemorrhage | | | | | | | | | | | | | | | | | | |  | | | |  |  |
| I69091 | | | | | Dysphagia following nontraumatic subarachnoid hemorrhage | | | | | | | | | | | | | | | | | | |  | | | |  |  |
| I69092 | | | | | Facial weakness following nontraumatic subarachnoid hemorrhage | | | | | | | | | | | | | | | | | | |  | | | |  |  |
| I69093 | | | | | Ataxia following nontraumatic subarachnoid hemorrhage | | | | | | | | | | | | | | | | | | |  | | | |  |  |
| I69098 | | | | | Other sequelae following nontraumatic subarachnoid hemorrhage | | | | | | | | | | | | | | | | | | |  | | | |  |  |
| I6910 | | | | | Unspecified sequelae of nontraumatic intracerebral hemorrhage | | | | | | | | | | | | | | | | | | |  | | | |  |  |
| I69110 | | | | | Attention and concentration deficit following nontraumatic intracerebral hemorrhage | | | | | | | | | | | | | | | | | | |  | | | |  |  |
| I69111 | | | | | Memory deficit following nontraumatic intracerebral hemorrhage | | | | | | | | | | | | | | | | | | |  | | | |  |  |
| I69112 | | | | | Visuospatial deficit and spatial neglect following nontraumatic intracerebral hemorrhage | | | | | | | | | | | | | | | | | | |  | | | |  |  |
| I69113 | | | | | Psychomotor deficit following nontraumatic intracerebral hemorrhage | | | | | | | | | | | | | | | | | | |  | | | |  |  |
| I69114 | | | | | Frontal lobe and executive function deficit following nontraumatic intracerebral hemorrhage | | | | | | | | | | | | | | | | | | |  | | | |  |  |
| I69115 | | | | | Cognitive social or emotional deficit following nontraumatic intracerebral hemorrhage | | | | | | | | | | | | | | | | | | |  | | | |  |  |
| I69118 | | | | | Other symptoms and signs involving cognitive functions following nontraumatic intracerebral hemorrhage | | | | | | | | | | | | | | | | | | |  | | | |  |  |
| I69119 | | | | | Unspecified symptoms and signs involving cognitive functions following nontraumatic intracerebral hemorrhage | | | | | | | | | | | | | | | | | | |  | | | |  |  |
| I69120 | | | | | Aphasia following nontraumatic intracerebral hemorrhage | | | | | | | | | | | | | | | | | | |  | | | |  |  |
| I69121 | | | | | Dysphasia following nontraumatic intracerebral hemorrhage | | | | | | | | | | | | | | | | | | |  | | | |  |  |
| I69122 | | | | | Dysarthria following nontraumatic intracerebral hemorrhage | | | | | | | | | | | | | | | | | | |  | | | |  |  |
| I69123 | | | | | Fluency disorder following nontraumatic intracerebral hemorrhage | | | | | | | | | | | | | | | | | | |  | | | |  |  |
| I69128 | | | | | Other speech and language deficits following nontraumatic intracerebral hemorrhage | | | | | | | | | | | | | | | | | | |  | | | |  |  |
| I69311 | | | | | Memory deficit following cerebral infarction | | | | | | | | | | | | | | | | | | |  | | | |  |  |
| I69312 | | | | | Visuospatial deficit and spatial neglect following cerebral infarction | | | | | | | | | | | | | | | | | | |  | | | |  |  |
| I69313 | | | | | Psychomotor deficit following cerebral infarction | | | | | | | | | | | | | | | | | | |  | | | |  |  |
| I69314 | | | | | Frontal lobe and executive function deficit following cerebral infarction | | | | | | | | | | | | | | | | | | |  | | | |  |  |
| I69315 | | | | | Cognitive social or emotional deficit following cerebral infarction | | | | | | | | | | | | | | | | | | |  | | | |  |  |
| I69318 | | | | | Other symptoms and signs involving cognitive functions following cerebral infarction | | | | | | | | | | | | | | | | | | |  | | | |  |  |
| I69319 | | | | | Unspecified symptoms and signs involving cognitive functions following cerebral infarction | | | | | | | | | | | | | | | | | | |  | | | |  |  |
| 1300 | | | | |  | | Tuberculous meningitis, unspecified | | | | | | | | | | | |  |  | | | | | |  |  |  |  |
| 1301 | | | | |  |  | Tuberculous meningitis, bacteriological or histological examination not done | | | | | | | | | | | |  |  | | | | | |  |  |  |  |
| 468 | | | | |  |  | Other specified slow virus infection of central nervous system | | | | | | | | | | | |  |  | | | | | |  |  |  |  |
| 469 | | | | |  |  | Unspecified slow virus infection of central nervous system | | | | | | | | | | | |  |  | | | | | |  |  |  |  |
| 470 | | | | |  |  | Meningitis due to coxsackie virus | | | | | | | | | | | |  |  | | | | | |  |  |  |  |
| 471 | | | | |  |  | Meningitis due to echo virus | | | | | | | | | | | |  |  | | | | | |  |  |  |  |
| 478 | | | | |  |  | Other specified viral meningitis | | | | | | | | | | | |  |  | | | | | |  |  |  |  |
| 479 | | | | |  |  | Unspecified viral meningitis | | | | | | | | | | | |  |  | | | | | |  |  |  |  |
| 491 | | | | |  |  | Meningitis due to adenovirus | | | | | | | | | | | |  |  | | | | | |  |  |  |  |
| 498 | | | | |  |  | Other specified non-arthropod-borne viral diseases of central nervous system | | | | | | | | | | | |  |  | | | | | |  |  |  |  |
| 499 | | | | |  |  | Unspecified non-arthropod-borne viral diseases of central nervous system | | | | | | | | | | | |  |  | | | | | |  |  |  |  |
| 520 | | | | |  |  | Postvaricella encephalitis | | | | | | | | | | | |  |  | | | | | |  |  |  |  |
| 530 | | | | |  |  | Herpes zoster with meningitis | | | | | | | | | | | |  |  | | | | | |  |  |  |  |
| 5319 | | | | |  |  | Herpes zoster with other nervous system complications | | | | | | | | | | | |  |  | | | | | |  |  |  |  |
| 5379 | | | | |  |  | Herpes zoster with other specified complications | | | | | | | | | | | |  |  | | | | | |  |  |  |  |
| 5472 | | | | |  |  | Herpes simplex meningitis | | | | | | | | | | | |  |  | | | | | |  |  |  |  |
| 550 | | | | |  |  | Postmeasles encephalitis | | | | | | | | | | | |  |  | | | | | |  |  |  |  |
| 360 | | | | |  |  | Meningococcal meningitis | | | | | | | | | | | |  |  | | | | | |  |  |  |  |
| 361 | | | | |  |  | Meningococcal encephalitis | | | | | | | | | | | |  |  | | | | | |  |  |  |  |
| 362 | | | | |  |  | Meningococcemia | | | | | | | | | | | |  |  | | | | | |  |  |  |  |
| 363 | | | | |  |  | Waterhouse-Friderichsen syndrome, meningococcal | | | | | | | | | | | |  |  | | | | | |  |  |  |  |
| 1302 | | | | |  |  | Tuberculous meningitis, bacteriological or histological examination unknown (at present) | | | | | | | | | | | |  |  | | | | | |  |  |  |  |
| 1303 | | | | |  |  | Tuberculous meningitis, tubercle bacilli found (in sputum) by microscopy | | | | | | | | | | | |  |  | | | | | |  |  |  |  |
| 1304 | | | | |  |  | Tuberculous meningitis, tubercle bacilli not found (in sputum) by microscopy, but found by bacterial culture | | | | | | | | | | | |  |  | | | | | |  |  |  |  |
| 1305 | | | | |  |  | Tuberculous meningitis, tubercle bacilli not found by bacteriological examination, but tuberculosis confirmed histologically | | | | | | | | | | | |  |  | | | | | |  |  |  |  |
| 1306 | | | | |  |  | Tuberculous meningitis, tubercle bacilli not found by bacteriological or histological examination, but tuberculosis confirmed by other methods [inoculation of animals] | | | | | | | | | | | |  |  | | | | | |  |  |  |  |
| 1310 | | | | |  |  | Tuberculoma of meninges, unspecified | | | | | | | | | | | |  |  | | | | | |  |  |  |  |
| 1311 | | | | |  |  | Tuberculoma of meninges, bacteriological or histological examination not done | | | | | | | | | | | |  |  | | | | | |  |  |  |  |
| 1312 | | | | |  |  | Tuberculoma of meninges, bacteriological or histological examination unknown (at present) | | | | | | | | | | | |  |  | | | | | |  |  |  |  |
| 1313 | | | | |  |  | Tuberculoma of meninges, tubercle bacilli found (in sputum) by microscopy | | | | | | | | | | | |  |  | | | | | |  |  |  |  |
| 1314 | | | | |  |  | Tuberculoma of meninges, tubercle bacilli not found (in sputum) by microscopy, but found by bacterial culture | | | | | | | | | | | |  |  | | | | | |  |  |  |  |
| 1315 | | | | |  |  | Tuberculoma of meninges, tubercle bacilli not found by bacteriological examination, but tuberculosis confirmed histologically | | | | | | | | | | | |  |  | | | | | |  |  |  |  |
| 1316 | | | | |  |  | Tuberculoma of meninges, tubercle bacilli not found by bacteriological or histological examination, but tuberculosis confirmed by other methods [inoculation of animals] | | | | | | | | | | | |  |  | | | | | |  |  |  |  |
| 1321 | | | | |  |  | Tuberculoma of brain, bacteriological or histological examination not done | | | | | | | | | | | |  |  | | | | | |  |  |  |  |
| 1322 | | | | |  |  | Tuberculoma of brain, bacteriological or histological examination unknown (at present) | | | | | | | | | | | |  |  | | | | | |  |  |  |  |
| 1323 | | | | |  |  | Tuberculoma of brain, tubercle bacilli found (in sputum) by microscopy | | | | | | | | | | | |  |  | | | | | |  |  |  |  |
| 1324 | | | | |  |  | Tuberculoma of brain, tubercle bacilli not found (in sputum) by microscopy, but found by bacterial culture | | | | | | | | | | | |  |  | | | | | |  |  |  |  |
| 1326 | | | | |  |  | Tuberculoma of brain, tubercle bacilli not found by bacteriological or histological examination, but tuberculosis confirmed by other | | | | | | | | | | | |  |  | | | | | |  |  |  |  |
| 1331 | | | | |  |  | Tuberculous abscess of brain, bacteriological or histological examination not done | | | | | | | | | | | |  |  | | | | | |  |  |  |  |
| 1332 | | | | |  |  | Tuberculous abscess of brain, bacteriological or histological examination unknown (at present) | | | | | | | | | | | |  |  | | | | | |  |  |  |  |
| 1333 | | | | |  |  | Tuberculous abscess of brain, tubercle bacilli found (in sputum) by microscopy | | | | | | | | | | | |  |  | | | | | |  |  |  |  |
| 1334 | | | | |  |  | Tuberculous abscess of brain, tubercle bacilli not found (in sputum) by microscopy, but found by bacterial culture | | | | | | | | | | | |  |  | | | | | |  |  |  |  |
| 1335 | | | | |  |  | Tuberculous abscess of brain, tubercle bacilli not found by bacteriological examination, but tuberculosis confirmed histologically | | | | | | | | | | | |  |  | | | | | |  |  |  |  |
| 1336 | | | | |  |  | Tuberculous abscess of brain, tubercle bacilli not found by bacteriological or histological examination, but tuberculosis confirmed by other methods [inoculation of animals] | | | | | | | | | | | |  |  | | | | | |  |  |  |  |
| 1360 | | | | |  |  | Tuberculous encephalitis or myelitis, unspecified | | | | | | | | | | | |  |  | | | | | |  |  |  |  |
| 1361 | | | | |  |  | Tuberculous encephalitis or myelitis, bacteriological or histological examination not done | | | | | | | | | | | |  |  | | | | | |  |  |  |  |
| 1362 | | | | |  |  | Tuberculous encephalitis or myelitis, bacteriological or histological examination unknown (at present) | | | | | | | | | | | |  |  | | | | | |  |  |  |  |
| 1363 | | | | |  |  | Tuberculous encephalitis or myelitis, tubercle bacilli found (in sputum) by microscopy | | | | | | | | | | | |  |  | | | | | |  |  |  |  |
| 1364 | | | | |  |  | Tuberculous encephalitis or myelitis, tubercle bacilli not found (in sputum) by microscopy, but found by bacterial culture | | | | | | | | | | | |  |  | | | | | |  |  |  |  |
| 1365 | | | | |  |  | Tuberculous encephalitis or myelitis, tubercle bacilli not found by bacteriological examination, but tuberculosis confirmed histologically | | | | | | | | | | | |  |  | | | | | |  |  |  |  |
| 1366 | | | | |  |  | Tuberculous encephalitis or myelitis, tubercle bacilli not found by bacteriological or histological examination, but tuberculosis confirmed by other methods [inoculation of animals] | | | | | | | | | | | |  |  | | | | | |  |  |  |  |
| 1380 | | | | |  |  | Other specified tuberculosis of central nervous system, unspecified | | | | | | | | | | | |  |  | | | | | |  |  |  |  |
| 1381 | | | | |  |  | Other specified tuberculosis of central nervous system, bacteriological or histological examination not done | | | | | | | | | | | |  |  | | | | | |  |  |  |  |
| 1382 | | | | |  |  | Other specified tuberculosis of central nervous system, bacteriological or histological examination unknown (at present) | | | | | | | | | | | |  |  | | | | | |  |  |  |  |
| 1383 | | | | |  |  | Other specified tuberculosis of central nervous system, tubercle bacilli found (in sputum) by microscopy | | | | | | | | | | | |  |  | | | | | |  |  |  |  |
| 1384 | | | | |  |  | Other specified tuberculosis of central nervous system, tubercle bacilli not found (in sputum) by microscopy, but found by bacterial culture | | | | | | | | | | | |  |  | | | | | |  |  |  |  |
| 1385 | | | | |  |  | Other specified tuberculosis of central nervous system, tubercle bacilli not found by bacteriological examination, but tuberculosis confirmed histologically | | | | | | | | | | | |  |  | | | | | |  |  |  |  |
| 1386 | | | | |  |  | Other specified tuberculosis of central nervous system, tubercle bacilli not found by bacteriological or histological examination, but tuberculosis confirmed by other methods [inoculation of animals] | | | | | | | | | | | |  |  | | | | | |  |  |  |  |
| 1390 1 | | | | |  |  | Unspecified tuberculosis of central nervous system, unspecified | | | | | | | | | | | |  |  | | | | | |  |  |  |  |
| 1391 | | | | |  |  | Unspecified tuberculosis of central nervous system, bacteriological or histological examination not done | | | | | | | | | | | |  |  | | | | | |  |  |  |  |
| 1392 | | | | |  |  | Unspecified tuberculosis of central nervous system, bacteriological or histological examination unknown (at present) | | | | | | | | | | | |  |  | | | | | |  |  |  |  |
| 1393 | | | | |  |  | Unspecified tuberculosis of central nervous system, tubercle bacilli found (in sputum) by microscopy | | | | | | | | | | | |  |  | | | | | |  |  |  |  |
| 1394 | | | | |  |  | Unspecified tuberculosis of central nervous system, tubercle bacilli not found (in sputum) by microscopy, but found by bacterial culture | | | | | | | | | | | |  |  | | | | | |  |  |  |  |
| 1395 | | | | |  |  | Unspecified tuberculosis of central nervous system, tubercle bacilli not found by bacteriological examination, but tuberculosis confirmed histologically | | | | | | | | | | | |  |  | | | | | |  |  |  |  |
| 1396 | | | | |  |  | Unspecified tuberculosis of central nervous system, tubercle bacilli not found by bacteriological or histological examination, but tuberculosis confirmed by other methods [inoculation of animals] | | | | | | | | | | | |  |  | | | | | |  |  |  |  |
| 1142 | | | | |  |  | Coccidioidal meningitis | | | | | | | | | | | |  |  | | | | | |  |  |  |  |
| 621 | | | | |  |  | Western equine encephalitis | | | | | | | | | | | |  |  | | | | | |  |  |  |  |
| 622 | | | | |  |  | Eastern equine encephalitis | | | | | | | | | | | |  |  | | | | | |  |  |  |  |
| 623 | | | | |  |  | St. Louis encephalitis | | | | | | | | | | | |  |  | | | | | |  |  |  |  |
| 624 | | | | |  |  | Australian encephalitis | | | | | | | | | | | |  |  | | | | | |  |  |  |  |
| 625 | | | | |  |  | California virus encephalitis | | | | | | | | | | | |  |  | | | | | |  |  |  |  |
| 632 | | | | |  |  | Central european encephalitis | | | | | | | | | | | |  |  | | | | | |  |  |  |  |
| 638 | | | | |  |  | Other specified tick-borne viral encephalitis | | | | | | | | | | | |  |  | | | | | |  |  |  |  |
| 3222 | | | | |  |  | Chronic meningitis | | | | | | | | | | | |  |  | | | | | |  |  |  |  |
| 3212 | | | | |  |  | Meningitis due to viruses not elsewhere classified | | | | | | | | | | | |  |  | | | | | |  |  |  |  |
| 3201 | | | | |  |  | Pneumococcal meningitis | | | | | | | | | | | |  |  | | | | | |  |  |  |  |
| A1782 | | | | |  | | Tuberculous meningoencephalitis | | | | | | | | | | | |  |  | | | | | |  |  |  |  |
| A3212 | | | | |  | | Listerial meningoencephalitis | | | | | | | | | | | |  |  | | | | | |  |  |  |  |
| A3981 | | | | |  | | Meningococcal encephalitis | | | | | | | | | | | |  |  | | | | | |  |  |  |  |
| A4282 | | | | |  | | Actinomycotic encephalitis | | | | | | | | | | | |  |  | | | | | |  |  |  |  |
| A5042 | | | | |  | | Late congenital syphilitic encephalitis | | | | | | | | | | | |  |  | | | | | |  |  |  |  |
| A5214 | | | | |  | | Late syphilitic encephalitis | | | | | | | | | | | |  |  | | | | | |  |  |  |  |
| A811 | | | | |  | | Subacute sclerosing panencephalitis | | | | | | | | | | | |  |  | | | | | |  |  |  |  |
| A830 | | | | |  | | Japanese encephalitis | | | | | | | | | | | |  |  | | | | | |  |  |  |  |
| A831 | | | | |  | | Western equine encephalitis | | | | | | | | | | | |  |  | | | | | |  |  |  |  |
| A832 | | | | |  | | Eastern equine encephalitis | | | | | | | | | | | |  |  | | | | | |  |  |  |  |
| A833 | | | | |  | | St Louis encephalitis | | | | | | | | | | | |  |  | | | | | |  |  |  |  |
| A834 | | | | |  | | Australian encephalitis | | | | | | | | | | | |  |  | | | | | |  |  |  |  |
| A835 | | | | |  | | California encephalitis | | | | | | | | | | | |  |  | | | | | |  |  |  |  |
| A838 | | | | |  | | Other mosquito-borne viral encephalitis | | | | | | | | | | | |  |  | | | | | |  |  |  |  |
| A839 | | | | |  | | Mosquito-borne viral encephalitis, unspecified | | | | | | | | | | | |  |  | | | | | |  |  |  |  |
| A840 | | | | |  | | Far Eastern tick-borne encephalitis [Russian spring-summer encephalitis] | | | | | | | | | | | |  |  | | | | | |  |  |  |  |
| A841 | | | | |  | | Central European tick-borne encephalitis | | | | | | | | | | | |  |  | | | | | |  |  |  |  |
| A848 | | | | |  | | Other tick-borne viral encephalitis | | | | | | | | | | | |  |  | | | | | |  |  |  |  |
| A849 | | | | |  | | Tick-borne viral encephalitis, unspecified | | | | | | | | | | | |  |  | | | | | |  |  |  |  |
| A850 | | | | |  | | Enteroviral encephalitis | | | | | | | | | | | |  |  | | | | | |  |  |  |  |
| A851 | | | | |  | | Adenoviral encephalitis | | | | | | | | | | | |  |  | | | | | |  |  |  |  |
| A852 | | | | |  | | Arthropod-borne viral encephalitis, unspecified | | | | | | | | | | | |  |  | | | | | |  |  |  |  |
| A858 | | | | |  | | Other specified viral encephalitis | | | | | | | | | | | |  |  | | | | | |  |  |  |  |
| A86 | | | | |  | | Unspecified viral encephalitis | | | | | | | | | | | |  |  | | | | | |  |  |  |  |
| A870 | | | | |  | | Enteroviral meningitis | | | | | | | | | | | |  |  | | | | | |  |  |  |  |
| A871 | | | | |  | | Adenoviral meningitis | | | | | | | | | | | |  |  | | | | | |  |  |  |  |
| A872 | | | | |  | | Lymphocytic choriomeningitis | | | | | | | | | | | |  |  | | | | | |  |  |  |  |
| A878 | | | | |  | | Other viral meningitis | | | | | | | | | | | |  |  | | | | | |  |  |  |  |
| A879 | | | | |  | | Viral meningitis, unspecified | | | | | | | | | | | |  |  | | | | | |  |  |  |  |
| A880 | | | | |  | | Enteroviral exanthematous fever [Boston exanthem] | | | | | | | | | | | |  |  | | | | | |  |  |  |  |
| A888 | | | | |  | | Other specified viral infections of central nervous system | | | | | | | | | | | |  |  | | | | | |  |  |  |  |
| A89 | | | | |  | | Unspecified viral infection of central nervous system | | | | | | | | | | | |  |  | | | | | |  |  |  |  |
| A90 | | | | |  | | Dengue fever [classical dengue] | | | | | | | | | | | |  |  | | | | | |  |  |  |  |
| B004 | | | | |  | | Herpesviral encephalitis | | | | | | | | | | | |  |  | | | | | |  |  |  |  |
| B0111 | | | | |  | | Varicella encephalitis and encephalomyelitis | | | | | | | | | | | |  |  | | | | | |  |  |  |  |
| B020 | | | | |  | | Zoster encephalitis | | | | | | | | | | | |  |  | | | | | |  |  |  |  |
| B050 | | | | |  | | Measles complicated by encephalitis | | | | | | | | | | | |  |  | | | | | |  |  |  |  |
| B0601 | | | | |  | | Rubella encephalitis | | | | | | | | | | | |  |  | | | | | |  |  |  |  |
| B1001 | | | | |  | | Human herpesvirus 6 encephalitis | | | | | | | | | | | |  |  | | | | | |  |  |  |  |
| B1009 | | | | |  | | Other human herpesvirus encephalitis | | | | | | | | | | | |  |  | | | | | |  |  |  |  |
| B262 | | | | |  | | Mumps encephalitis | | | | | | | | | | | |  |  | | | | | |  |  |  |  |
| B4081 | | | | |  | | Blastomycotic meningoencephalitis | | | | | | | | | | | |  |  | | | | | |  |  |  |  |
| B5742 | | | | |  | | Meningoencephalitis in Chagas' disease | | | | | | | | | | | |  |  | | | | | |  |  |  |  |
| B582 | | | | |  | | Toxoplasma meningoencephalitis | | | | | | | | | | | |  |  | | | | | |  |  |  |  |
| B6011 | | | | |  | | Meningoencephalitis due to Acanthamoeba (culbertsoni) | | | | | | | | | | | |  |  | | | | | |  |  |  |  |
| B941 | | | | |  | | Sequelae of viral encephalitis | | | | | | | | | | | |  |  | | | | | |  |  |  |  |
| G0400 | | | | |  | | Acute disseminated encephalitis and encephalomyelitis, unspecified | | | | | | | | | | | |  |  | | | | | |  |  |  |  |
| G0401 | | | | |  | | Postinfectious acute disseminated encephalitis and encephalomyelitis (postinfectious ADEM) | | | | | | | | | | | |  |  | | | | | |  |  |  |  |
| G0402 | | | | |  | | Postimmunization acute disseminated encephalitis, myelitis and encephalomyelitis | | | | | | | | | | | |  |  | | | | | |  |  |  |  |
| G042 | | | | |  | | Bacterial meningoencephalitis and meningomyelitis, not elsewhere classified | | | | | | | | | | | |  |  | | | | | |  |  |  |  |
| G0481 | | | | |  | | Other encephalitis and encephalomyelitis | | | | | | | | | | | |  |  | | | | | |  |  |  |  |
| G0490 | | | | |  | | Encephalitis and encephalomyelitis, unspecified | | | | | | | | | | | |  |  | | | | | |  |  |  |  |
| G361 | | | | |  | | Acute and subacute hemorrhagic leukoencephalitis [Hurst] | | | | | | | | | | | |  |  | | | | | |  |  |  |  |
| A1782 | | | | |  | | Tuberculous meningoencephalitis | | | | | | | | | | | |  |  | | | | | |  |  |  |  |
| A3212 | | | | |  | | Listerial meningoencephalitis | | | | | | | | | | | |  |  | | | | | |  |  |  |  |
| A5141 | | | | |  | | Secondary syphilitic meningitis | | | | | | | | | | | |  |  | | | | | |  |  |  |  |
| A5213 | | | | |  | | Late syphilitic meningitis | | | | | | | | | | | |  |  | | | | | |  |  |  |  |
| A5481 | | | | |  | | Gonococcal meningitis | | | | | | | | | | | |  |  | | | | | |  |  |  |  |
| A6921 | | | | |  | | Meningitis due to Lyme disease | | | | | | | | | | | |  |  | | | | | |  |  |  |  |
| A870 | | | | |  | | Enteroviral meningitis | | | | | | | | | | | |  |  | | | | | |  |  |  |  |
| A871 | | | | |  | | Adenoviral meningitis | | | | | | | | | | | |  |  | | | | | |  |  |  |  |
| A872 | | | | |  | | Lymphocytic choriomeningitis | | | | | | | | | | | |  |  | | | | | |  |  |  |  |
| A878 | | | | |  | | Other viral meningitis | | | | | | | | | | | |  |  | | | | | |  |  |  |  |
| A879 | | | | |  | | Viral meningitis, unspecified | | | | | | | | | | | |  |  | | | | | |  |  |  |  |
| A880 | | | | |  | | Enteroviral exanthematous fever [Boston exanthem] | | | | | | | | | | | |  |  | | | | | |  |  |  |  |
| A888 | | | | |  | | Other specified viral infections of central nervous system | | | | | | | | | | | |  |  | | | | | |  |  |  |  |
| A89 | | | | |  | | Unspecified viral infection of central nervous system | | | | | | | | | | | |  |  | | | | | |  |  |  |  |
| B003 | | | | |  | | Herpesviral meningitis | | | | | | | | | | | |  |  | | | | | |  |  |  |  |
| B010 | | | | |  | | Varicella meningitis | | | | | | | | | | | |  |  | | | | | |  |  |  |  |
| B021 | | | | |  | | Zoster meningitis | | | | | | | | | | | |  |  | | | | | |  |  |  |  |
| B051 | | | | |  | | Measles complicated by meningitis | | | | | | | | | | | |  |  | | | | | |  |  |  |  |
| B0602 | | | | |  | | Rubella meningitis | | | | | | | | | | | |  |  | | | | | |  |  |  |  |
| B261 | | | | |  | | Mumps meningitis | | | | | | | | | | | |  |  | | | | | |  |  |  |  |
| B2702 | | | | |  | | Gammaherpesviral mononucleosis with meningitis | | | | | | | | | | | |  |  | | | | | |  |  |  |  |
| B2712 | | | | |  | | Cytomegaloviral mononucleosis with meningitis | | | | | | | | | | | |  |  | | | | | |  |  |  |  |
| B2782 | | | | |  | | Other infectious mononucleosis with meningitis | | | | | | | | | | | |  |  | | | | | |  |  |  |  |
| B2792 | | | | |  | | Infectious mononucleosis, unspecified with meningitis | | | | | | | | | | | |  |  | | | | | |  |  |  |  |
| B384 | | | | |  | | Coccidioidomycosis meningitis | | | | | | | | | | | |  |  | | | | | |  |  |  |  |
| B5741 | | | | |  | | Meningitis in Chagas' disease | | | | | | | | | | | |  |  | | | | | |  |  |  |  |
| D8681 | | | | |  | | Sarcoid meningitis | | | | | | | | | | | |  |  | | | | | |  |  |  |  |
| G000 | | | | |  | | Hemophilus meningitis | | | | | | | | | | | |  |  | | | | | |  |  |  |  |
| G001 | | | | |  | | Pneumococcal meningitis | | | | | | | | | | | |  |  | | | | | |  |  |  |  |
| G002 | | | | |  | | Streptococcal meningitis | | | | | | | | | | | |  |  | | | | | |  |  |  |  |
| G003 | | | | |  | | Staphylococcal meningitis | | | | | | | | | | | |  |  | | | | | |  |  |  |  |
| G008 | | | | |  | | Other bacterial meningitis | | | | | | | | | | | |  |  | | | | | |  |  |  |  |
| G009 | | | | |  | | Bacterial meningitis, unspecified | | | | | | | | | | | |  |  | | | | | |  |  |  |  |
| G01 | | | | |  | | Meningitis in bacterial diseases classified elsewhere | | | | | | | | | | | |  |  | | | | | |  |  |  |  |
| G02 | | | | |  | | Meningitis in other infectious and parasitic diseases classified elsewhere | | | | | | | | | | | |  |  | | | | | |  |  |  |  |
| G030 | | | | |  | | Nonpyogenic meningitis | | | | | | | | | | | |  |  | | | | | |  |  |  |  |
| G031 | | | | |  | | Chronic meningitis | | | | | | | | | | | |  |  | | | | | |  |  |  |  |
| G032 | | | | |  | | Benign recurrent meningitis [Mollaret] | | | | | | | | | | | |  |  | | | | | |  |  |  |  |
| G038 | | | | |  | | Meningitis due to other specified causes | | | | | | | | | | | |  |  | | | | | |  |  |  |  |
| G039 | | | | |  | | Meningitis, unspecified | | | | | | | | | | | |  |  | | | | | |  |  |  |  |
| 34591 | |  | | | Epilepsy, unspecified, with intractable epilepsy | | | | | | | | | | | | | | | | |  |  |  |  |  |  |  |  |
| 34570 | |  |  |  | Epilepsia partialis continua, without mention of intractable epilepsy | | | | | | | | | | | | | | | | |  |  |  |  |  |  |  |  |
| 34571 | |  |  |  | Epilepsia partialis continua, with intractable epilepsy | | | | | | | | | | | | | | | | |  |  |  |  |  |  |  |  |
| 34580 | |  |  |  | Other forms of epilepsy and recurrent seizures, without mention of intractable epilepsy | | | | | | | | | | | | | | | | |  |  |  |  |  |  |  |  |
| 34581 | |  |  |  | Other forms of epilepsy and recurrent seizures, with intractable epilepsy | | | | | | | | | | | | | | | | |  |  |  |  |  |  |  |  |
| 34590 | |  |  |  | Epilepsy, unspecified, without mention of intractable epilepsy | | | | | | | | | | | | | | | | |  |  |  |  |  |  |  |  |
| 34591 | |  |  |  | Epilepsy, unspecified, with intractable epilepsy | | | | | | | | | | | | | | | | |  |  |  |  |  |  |  |  |
| 64940 | |  |  |  | Epilepsy complicating pregnancy, childbirth, or the puerperium, unspecified as to episode of care or not applicable | | | | | | | | | | | | | | | | |  |  |  |  |  |  |  |  |
| 64941 | |  |  |  | Epilepsy complicating pregnancy, childbirth, or the puerperium, delivered, with or without mention of antepartum condition | | | | | | | | | | | | | | | | |  |  |  |  |  |  |  |  |
| 64942 | |  |  |  | Epilepsy complicating pregnancy, childbirth, or the puerperium, delivered, with mention of postpartum complication | | | | | | | | | | | | | | | | |  |  |  |  |  |  |  |  |
| 64943 | |  |  |  | Epilepsy complicating pregnancy, childbirth, or the puerperium, antepartum condition or complication | | | | | | | | | | | | | | | | |  |  |  |  |  |  |  |  |
| 64944 | |  |  |  | Epilepsy complicating pregnancy, childbirth, or the puerperium, postpartum condition or complication | | | | | | | | | | | | | | | | |  |  |  |  |  |  |  |  |
| G40001 | |  | | | Localization-related (focal) (partial) idiopathic epilepsy and epileptic syndromes with seizures of localized onset, not intractable, with status epilepticus | | | | | | | | | | | | | | | | |  |  |  |  |  |  |  |  |
| G40009 | |  | | | Localization-related (focal) (partial) idiopathic epilepsy and epileptic syndromes with seizures of localized onset, not intractable, without status epilepticus | | | | | | | | | | | | | | | | |  |  |  |  |  |  |  |  |
| G40011 | |  | | | Localization-related (focal) (partial) idiopathic epilepsy and epileptic syndromes with seizures of localized onset, intractable, with status epilepticus | | | | | | | | | | | | | | | | |  |  |  |  |  |  |  |  |
| G40019 | |  | | | Localization-related (focal) (partial) idiopathic epilepsy and epileptic syndromes with seizures of localized onset, intractable, without status epilepticus | | | | | | | | | | | | | | | | |  |  |  |  |  |  |  |  |
| G40101 | |  | | | | | | Localization-related (focal) (partial) symptomatic epilepsy and epileptic syndromes with simple partial seizures, not intractable, with status epilepticus | | | | | | | | | | | | | |  |  |  |  |  |  |  |  |
| G40109 | |  | | | | | | Localization-related (focal) (partial) symptomatic epilepsy and epileptic syndromes with simple partial seizures, not intractable, without status epilepticus | | | | | | | | | | | | | |  |  |  |  |  |  |  |  |
| G40111 | |  | | | | | | Localization-related (focal) (partial) symptomatic epilepsy and epileptic syndromes with simple partial seizures, intractable, with status epilepticus | | | | | | | | | | | | | |  |  |  |  |  |  |  |  |
| G40119 | |  | | | | | | Localization-related (focal) (partial) symptomatic epilepsy and epileptic syndromes with simple partial seizures, intractable, without status epilepticus | | | | | | | | | | | | | |  |  |  |  |  |  |  |  |
| G40201 | |  | | | | | | Localization-related (focal) (partial) symptomatic epilepsy and epileptic syndromes with complex partial seizures, not intractable, with status epilepticus | | | | | | | | | | | | | |  |  |  |  |  |  |  |  |
| G40209 | |  | | | | | | Localization-related (focal) (partial) symptomatic epilepsy and epileptic syndromes with complex partial seizures, not intractable, without status epilepticus | | | | | | | | | | | | | |  |  |  |  |  |  |  |  |
| G40211 | |  | | | | | | Localization-related (focal) (partial) symptomatic epilepsy and epileptic syndromes with complex partial seizures, intractable, with status epilepticus | | | | | | | | | | | | | |  |  |  |  |  |  |  |  |
| G40219 | |  | | | | | | Localization-related (focal) (partial) symptomatic epilepsy and epileptic syndromes with complex partial seizures, intractable, without status epilepticus | | | | | | | | | | | | | |  |  |  |  |  |  |  |  |
| G40301 | |  | | | | | | Generalized idiopathic epilepsy and epileptic syndromes, not intractable, with status epilepticus | | | | | | | | | | | | | |  |  |  |  |  |  |  |  |
| G40309 | |  | | | | | | Generalized idiopathic epilepsy and epileptic syndromes, not intractable, without status epilepticus | | | | | | | | | | | | | |  |  |  |  |  |  |  |  |
| G40311 | |  | | | | | | Generalized idiopathic epilepsy and epileptic syndromes, intractable, with status epilepticus | | | | | | | | | | | | | |  |  |  |  |  |  |  |  |
| G40319 | |  | | | | | | Generalized idiopathic epilepsy and epileptic syndromes, intractable, without status epilepticus | | | | | | | | | | | | | |  |  |  |  |  |  |  |  |
| G40401 | |  | | | | | | Other generalized epilepsy and epileptic syndromes, not intractable, with status epilepticus | | | | | | | | | | | | | |  |  |  |  |  |  |  |  |
| G40409 | |  | | | | | | Other generalized epilepsy and epileptic syndromes, not intractable, without status epilepticus | | | | | | | | | | | | | |  |  |  |  |  |  |  |  |
| G40411 | |  | | | | | | Other generalized epilepsy and epileptic syndromes, intractable, with status epilepticus | | | | | | | | | | | | | |  |  |  |  |  |  |  |  |
| G40419 | |  | | | | | | Other generalized epilepsy and epileptic syndromes, intractable, without status epilepticus | | | | | | | | | | | | | |  |  |  |  |  |  |  |  |
| G40501 | |  | | | | | | Epileptic seizures related to external causes, not intractable, with status epilepticus | | | | | | | | | | | | | |  |  |  |  |  |  |  |  |
| G40509 | |  | | | | | | Epileptic seizures related to external causes, not intractable, without status epilepticus | | | | | | | | | | | | | |  |  |  |  |  |  |  |  |
| G40801 | |  | | | | | | Other epilepsy, not intractable, with status epilepticus | | | | | | | | | | | | | |  |  |  |  |  |  |  |  |
| G40802 | |  | | | | | | Other epilepsy, not intractable, without status epilepticus | | | | | | | | | | | | | |  |  |  |  |  |  |  |  |
| G40803 | |  | | | | | | Other epilepsy, intractable, with status epilepticus | | | | | | | | | | | | | |  |  |  |  |  |  |  |  |
| G40804 | |  | | | | | | Other epilepsy, intractable, without status epilepticus | | | | | | | | | | | | | |  |  |  |  |  |  |  |  |
| G40811 | |  | | | | | | Lennox-Gastaut syndrome, not intractable, with status epilepticus | | | | | | | | | | | | | |  |  |  |  |  |  |  |  |
| G40812 | |  | | | | | | Lennox-Gastaut syndrome, not intractable, without status epilepticus | | | | | | | | | | | | | |  |  |  |  |  |  |  |  |
| G40813 | |  | | | | | | Lennox-Gastaut syndrome, intractable, with status epilepticus | | | | | | | | | | | | | |  |  |  |  |  |  |  |  |
| G40821 | |  | | | | | | Epileptic spasms, not intractable, with status epilepticus | | | | | | | | | | | | | |  |  |  |  |  |  |  |  |
| G40822 | |  | | | | | | Epileptic spasms, not intractable, without status epilepticus | | | | | | | | | | | | | |  |  |  |  |  |  |  |  |
| G40823 | |  | | | | | | Epileptic spasms, intractable, with status epilepticus | | | | | | | | | | | | | |  |  |  |  |  |  |  |  |
| G40824 | |  | | | | | | Epileptic spasms, intractable, without status epilepticus | | | | | | | | | | | | | |  |  |  |  |  |  |  |  |
| G4089 | |  | | | | | | Other seizures | | | | | | | | | | | | | |  |  |  |  |  |  |  |  |
| G40901 | |  | | | | | | Epilepsy, unspecified, not intractable, with status epilepticus | | | | | | | | | | | | | |  |  |  |  |  |  |  |  |
| G40909 | |  | | | | | | Epilepsy, unspecified, not intractable, without status epilepticus | | | | | | | | | | | | | |  |  |  |  |  |  |  |  |
| G40911 | |  | | | | | | Epilepsy, unspecified, intractable, with status epilepticus | | | | | | | | | | | | | |  |  |  |  |  |  |  |  |
| G40919 | |  | | | | | | Epilepsy, unspecified, intractable, without status epilepticus | | | | | | | | | | | | | |  |  |  |  |  |  |  |  |
| G40A01 | |  | | | | | | Absence epileptic syndrome, not intractable, with status epilepticus | | | | | | | | | | | | | |  |  |  |  |  |  |  |  |
| G40A09 | |  | | | | | | Absence epileptic syndrome, not intractable, without status epilepticus | | | | | | | | | | | | | |  |  |  |  |  |  |  |  |
| G40A11 | |  | | | | | | Absence epileptic syndrome, intractable, with status epilepticus | | | | | | | | | | | | | |  |  |  |  |  |  |  |  |
| G40A19 | |  | | | | | | Absence epileptic syndrome, intractable, without status epilepticus | | | | | | | | | | | | | |  |  |  |  |  |  |  |  |
| G40B01 | |  | | | | | | Juvenile myoclonic epilepsy, not intractable, with status epilepticus | | | | | | | | | | | | | |  |  |  |  |  |  |  |  |
| G40B09 | |  | | | | | | Juvenile myoclonic epilepsy, not intractable, without status epilepticus | | | | | | | | | | | | | |  |  |  |  |  |  |  |  |
| G40B11 | |  | | | | | | Juvenile myoclonic epilepsy, intractable, with status epilepticus | | | | | | | | | | | | | |  |  |  |  |  |  |  |  |
| G40B19 | |  | | | | | | Juvenile myoclonic epilepsy, intractable, without status epilepticus | | | | | | | | | | | | | |  |  |  |  |  |  |  |  |
| G40A01 | |  | | | | | | Absence epileptic syndrome, not intractable, with status epilepticus | | | | | | | | | | | | | |  |  |  |  |  |  |  |  |
| G40A09 | |  | | | | | | Absence epileptic syndrome, not intractable, without status epilepticus | | | | | | | | | | | | | |  |  |  |  |  |  |  |  |
| G40A11 | |  | | | | | | Absence epileptic syndrome, intractable, with status epilepticus | | | | | | | | | | | | | |  |  |  |  |  |  |  |  |
| G40A19 | |  | | | | | | Absence epileptic syndrome, intractable, without status epilepticus | | | | | | | | | | | | | |  |  |  |  |  |  |  |  |
| G40B01 | |  | | | | | | Juvenile myoclonic epilepsy, not intractable, with status epilepticus | | | | | | | | | | | | | |  |  |  |  |  |  |  |  |
| G40B09 | |  | | | | | | Juvenile myoclonic epilepsy, not intractable, without status epilepticus | | | | | | | | | | | | | |  |  |  |  |  |  |  |  |
| G40B11 | |  | | | | | | Juvenile myoclonic epilepsy, intractable, with status epilepticus | | | | | | | | | | | | | |  |  |  |  |  |  |  |  |
| G40B19 | |  | | | | | | Juvenile myoclonic epilepsy, intractable, without status epilepticus | | | | | | | | | | | | | |  |  |  |  |  |  |  |  |
| 3312 | | |  | | | |  | | Senile degeneration of brain | | | | | | | | | | | | | | | | | | |  |  |
| 3313 | | |  | | | |  |  | Communicating hydrocephalus | | | | | | | | | | | | | | | | | | |  |  |
| 3314 | | |  | | | |  |  | Obstructive hydrocephalus | | | | | | | | | | | | | | | | | | |  |  |
| 33189 | | |  | | | |  |  | Other cerebral degeneration | | | | | | | | | | | | | | | | | | |  |  |
| 3319 | | |  | | | |  |  | Cerebral degeneration, unspecified | | | | | | | | | | | | | | | | | | |  |  |
| 3301 | | |  | | | |  |  | Cerebral lipidoses | | | | | | | | | | | | | | | | | | |  |  |
| 3302 | | |  | | | |  |  | Cerebral degeneration in generalized lipidoses | | | | | | | | | | | | | | | | | | |  |  |
| 3303 | | |  | | | |  |  | Cerebral degeneration of childhood in other diseases classified elsewhere | | | | | | | | | | | | | | | | | | |  |  |
| 3308 | | |  | | | |  |  | Other specified cerebral degenerations in childhood | | | | | | | | | | | | | | | | | | |  |  |
| 3309 | | |  | | | |  |  | Unspecified cerebral degeneration in childhood | | | | | | | | | | | | | | | | | | |  |  |
| 3310 | | |  | | | |  |  | Alzheimer's disease | | | | | | | | | | | | | | | | | | |  |  |
| 3317 | | |  | | | |  |  | Cerebral degeneration in diseases classified elsewhere | | | | | | | | | | | | | | | | | | |  |  |
| 33189 | | |  | | | |  |  | Other cerebral degeneration | | | | | | | | | | | | | | | | | | |  |  |
| 3319 | | |  | | | |  |  | Cerebral degeneration, unspecified | | | | | | | | | | | | | | | | | | |  |  |
|  | | |  | | | |  |  |  | | | | | | | | | | | | | | | | | | |  |  |
| 4378 | | |  | | | |  |  | Other ill-defined cerebrovascular disease | | | | | | | | | | | | | | | | | | |  |  |
| 4379 | | |  | | | |  |  | Unspecified cerebrovascular disease | | | | | | | | | | | | | | | | | | |  |  |
| 74100 | | |  | | | |  |  | Spina bifida with hydrocephalus, unspecified region | | | | | | | | | | | | | | | | | | |  |  |
| 74101 | | |  | | | |  |  | Spina bifida with hydrocephalus, cervical region | | | | | | | | | | | | | | | | | | |  |  |
| 74102 | | |  | | | |  |  | Spina bifida with hydrocephalus, dorsal (thoracic) region | | | | | | | | | | | | | | | | | | |  |  |
| 74103 | | |  | | | |  |  | Spina bifida with hydrocephalus, lumbar region | | | | | | | | | | | | | | | | | | |  |  |
| 74190 | | |  | | | |  |  | Spina bifida without mention of hydrocephalus, unspecified region | | | | | | | | | | | | | | | | | | |  |  |
| 74191 | | |  | | | |  |  | Spina bifida without mention of hydrocephalus, cervical region | | | | | | | | | | | | | | | | | | |  |  |
| 74192 | | |  | | | |  |  | Spina bifida without mention of hydrocephalus, dorsal (thoracic) region | | | | | | | | | | | | | | | | | | |  |  |
| 74193 | | |  | | | |  |  | Spina bifida without mention of hydrocephalus, lumbar region | | | | | | | | | | | | | | | | | | |  |  |
| 7423 | | |  | | | |  |  | Coxsackie myocarditis | | | | | | | | | | | | | | | | | | |  |  |
| 4380 | | |  | | | |  |  | Late effects of cerebrovascular disease, cognitive deficits | | | | | | | | | | | | | | | | | | |  |  |
| 43810 | | |  | | | |  |  | Late effects of cerebrovascular disease, speech and language deficit, unspecified | | | | | | | | | | | | | | | | | | |  |  |
| 43811 | | |  | | | |  |  | Late effects of cerebrovascular disease, aphasia | | | | | | | | | | | | | | | | | | |  |  |
| 43812 | | |  | | | |  |  | Late effects of cerebrovascular disease, dysphasia | | | | | | | | | | | | | | | | | | |  |  |
| 43813 | | |  | | | |  |  | Late effects of cerebrovascular disease, dysarthria | | | | | | | | | | | | | | | | | | |  |  |
| 43814 | | |  | | | |  |  | Late effects of cerebrovascular disease, fluency disorder | | | | | | | | | | | | | | | | | | |  |  |
| 43819 | | |  | | | |  |  | Late effects of cerebrovascular disease, other speech and language deficits | | | | | | | | | | | | | | | | | | |  |  |
| 43820 | | |  | | | |  |  | Late effects of cerebrovascular disease, hemiplegia affecting unspecified side | | | | | | | | | | | | | | | | | | |  |  |
| 43821 | | |  | | | |  |  | Late effects of cerebrovascular disease, hemiplegia affecting dominant side | | | | | | | | | | | | | | | | | | |  |  |
| 43822 | | |  | | | |  |  | Late effects of cerebrovascular disease, hemiplegia affecting nondominant side | | | | | | | | | | | | | | | | | | |  |  |
| 43830 | | |  | | | |  |  | Late effects of cerebrovascular disease, monoplegia of upper limb affecting unspecified side | | | | | | | | | | | | | | | | | | |  |  |
| 43831 | | |  | | | |  |  | Late effects of cerebrovascular disease, monoplegia of upper limb affecting dominant side | | | | | | | | | | | | | | | | | | |  |  |
| 43832 | | |  | | | |  |  | Late effects of cerebrovascular disease, monoplegia of upper limb affecting nondominant side | | | | | | | | | | | | | | | | | | |  |  |
| 43840 | | |  | | | |  |  | Late effects of cerebrovascular disease, monoplegia of lower limb affecting unspecified side | | | | | | | | | | | | | | | | | | |  |  |
| 43841 | | |  | | | |  |  | Late effects of cerebrovascular disease, monoplegia of lower limb affecting dominant side | | | | | | | | | | | | | | | | | | |  |  |
| 43842 | | |  | | | |  |  | Late effects of cerebrovascular disease, monoplegia of lower limb affecting nondominant side | | | | | | | | | | | | | | | | | | |  |  |
| 43850 | | |  | | | |  |  | Late effects of cerebrovascular disease, other paralytic syndrome affecting unspecified side | | | | | | | | | | | | | | | | | | |  |  |
| 43851 | | |  | | | |  |  | Late effects of cerebrovascular disease, other paralytic syndrome affecting dominant side | | | | | | | | | | | | | | | | | | |  |  |
| 43852 | | |  | | | |  |  | Late effects of cerebrovascular disease, other paralytic syndrome affecting nondominant side | | | | | | | | | | | | | | | | | | |  |  |
| 43853 | | |  | | | |  |  | Late effects of cerebrovascular disease, other paralytic syndrome, bilateral | | | | | | | | | | | | | | | | | | |  |  |
| 4386 | | |  | | | |  |  | Late effects of cerebrovascular disease, alterations of sensations | | | | | | | | | | | | | | | | | | |  |  |
| 4387 | | |  | | | |  |  | Late effects of cerebrovascular disease, disturbances of vision | | | | | | | | | | | | | | | | | | |  |  |
| 43881 | | |  | | | |  |  | Other late effects of cerebrovascular disease, apraxia | | | | | | | | | | | | | | | | | | |  |  |
| 43882 | | |  | | | |  |  | Other late effects of cerebrovascular disease, dysphagia | | | | | | | | | | | | | | | | | | |  |  |
| 43883 | | |  | | | |  |  | Other late effects of cerebrovascular disease, facial weakness | | | | | | | | | | | | | | | | | | |  |  |
| 43884 | | |  | | | |  |  | Other late effects of cerebrovascular disease, ataxia | | | | | | | | | | | | | | | | | | |  |  |
| 43885 | | |  | | | |  |  | Other late effects of cerebrovascular disease, vertigo | | | | | | | | | | | | | | | | | | |  |  |
| 43889 | | |  | | | |  |  | Other late effects of cerebrovascular disease | | | | | | | | | | | | | | | | | | |  |  |
| 4380 | | |  | | | |  |  | Unspecified cerebrovascular disease | | | | | | | | | | | | | | | | | | |  |  |
| 43810 | | |  | | | |  |  | Late effects of cerebrovascular disease, speech and language deficit, unspecified | | | | | | | | | | | | | | | | | | |  |  |
| G311 | | |  | | | |  | | Senile degeneration of brain, not elsewhere classified | | | | | | | | | | | | | | | | | | |  |  |
| G910 | | |  | | | |  | | Communicating hydrocephalus | | | | | | | | | | | | | | | | | | |  |  |
| G911 | | |  | | | |  | | Obstructive hydrocephalus | | | | | | | | | | | | | | | | | | |  |  |
| G912 | | |  | | | |  | | (Idiopathic) normal pressure hydrocephalus | | | | | | | | | | | | | | | | | | |  |  |
| G913 | | |  | | | |  | | Post-traumatic hydrocephalus, unspecified | | | | | | | | | | | | | | | | | | |  |  |
| G914 | | |  | | | |  | | Hydrocephalus in diseases classified elsewhere | | | | | | | | | | | | | | | | | | |  |  |
| Q038 | | |  | | | |  | | Other congenital hydrocephalus | | | | | | | | | | | | | | | | | | |  |  |
| Q039 | | |  | | | |  | | Congenital hydrocephalus, unspecified | | | | | | | | | | | | | | | | | | |  |  |
| Q050 | | |  | | | |  | | Cervical spina bifida with hydrocephalus | | | | | | | | | | | | | | | | | | |  |  |
| Q051 | | |  | | | |  | | Thoracic spina bifida with hydrocephalus | | | | | | | | | | | | | | | | | | |  |  |
| Q052 | | |  | | | |  | | Lumbar spina bifida with hydrocephalus | | | | | | | | | | | | | | | | | | |  |  |
| Q053 | | |  | | | |  | | Sacral spina bifida with hydrocephalus | | | | | | | | | | | | | | | | | | |  |  |
| Q054 | | |  | | | |  | | Unspecified spina bifida with hydrocephalus | | | | | | | | | | | | | | | | | | |  |  |
| Q055 | | |  | | | |  | | Cervical spina bifida without hydrocephalus | | | | | | | | | | | | | | | | | | |  |  |
| Q056 | | |  | | | |  | | Thoracic spina bifida without hydrocephalus | | | | | | | | | | | | | | | | | | |  |  |
| Q057 | | |  | | | |  | | Lumbar spina bifida without hydrocephalus | | | | | | | | | | | | | | | | | | |  |  |
| Q058 | | |  | | | |  | | Sacral spina bifida without hydrocephalus | | | | | | | | | | | | | | | | | | |  |  |
| Q050 | | |  | | | |  | | Cervical spina bifida with hydrocephalus | | | | | | | | | | | | | | | | | | |  |  |
| Q0700 | | |  | | | |  | | Arnold-Chiari syndrome without spina bifida or hydrocephalus | | | | | | | | | | | | | | | | | | |  |  |
| Q0701 | | |  | | | |  | | Arnold-Chiari syndrome with spina bifida | | | | | | | | | | | | | | | | | | |  |  |
| Q0702 | | |  | | | |  | | Arnold-Chiari syndrome with hydrocephalus | | | | | | | | | | | | | | | | | | |  |  |
| Q0703 | | |  | | | |  | | Arnold-Chiari syndrome with spina bifida and hydrocephalus | | | | | | | | | | | | | | | | | | |  |  |
| Q078 | | |  | | | |  | | Other specified congenital malformations of nervous system | | | | | | | | | | | | | | | | | | |  |  |
| Q079 | | |  | | | |  | | Congenital malformation of nervous system, unspecified | | | | | | | | | | | | | | | | | | |  |  |
| A066 | | |  | | | |  | | Amebic brain abscess | | | | | | | | | | | | | | | | | | |  |  |
| A5482 | | |  | | | |  | | Gonococcal brain abscess | | | | | | | | | | | | | | | | | | |  |  |
| B431 | | |  | | | |  | | Pheomycotic brain abscess | | | | | | | | | | | | | | | | | | |  |  |
| C710 | | |  | | | |  | | Malignant neoplasm of cerebrum, except lobes and ventricles | | | | | | | | | | | | | | | | | | |  |  |
| C711 | | |  | | | |  | | Malignant neoplasm of frontal lobe | | | | | | | | | | | | | | | | | | |  |  |
| C712 | | |  | | | |  | | Malignant neoplasm of temporal lobe | | | | | | | | | | | | | | | | | | |  |  |
| C713 | | |  | | | |  | | Malignant neoplasm of parietal lobe | | | | | | | | | | | | | | | | | | |  |  |
| C714 | | |  | | | |  | | Malignant neoplasm of occipital lobe | | | | | | | | | | | | | | | | | | |  |  |
| C715 | | |  | | | |  | | Malignant neoplasm of cerebral ventricle | | | | | | | | | | | | | | | | | | |  |  |
| C716 | | |  | | | |  | | Malignant neoplasm of cerebellum | | | | | | | | | | | | | | | | | | |  |  |
| C717 | | |  | | | |  | | Malignant neoplasm of brain stem | | | | | | | | | | | | | | | | | | |  |  |
| C718 | | |  | | | |  | | Malignant neoplasm of overlapping sites of brain | | | | | | | | | | | | | | | | | | |  |  |
| C719 | | |  | | | |  | | Malignant neoplasm of brain, unspecified | | | | | | | | | | | | | | | | | | |  |  |
| C729 | | |  | | | |  | | Malignant neoplasm of central nervous system, unspecified | | | | | | | | | | | | | | | | | | |  |  |
| C7932 | | |  | | | |  | | Secondary malignant neoplasm of cerebral meninges | | | | | | | | | | | | | | | | | | |  |  |
| C7932 | | |  | | | |  | | Secondary malignant neoplasm of cerebral meninges | | | | | | | | | | | | | | | | | | |  |  |
| C7940 | | |  | | | |  | | Secondary malignant neoplasm of unspecified part of nervous system | | | | | | | | | | | | | | | | | | |  |  |
| C7949 | | |  | | | |  | | Secondary malignant neoplasm of other parts of nervous system | | | | | | | | | | | | | | | | | | |  |  |
| D320 | | |  | | | |  | | Benign neoplasm of cerebral meninges | | | | | | | | | | | | | | | | | | |  |  |
| D321 | | |  | | | |  | | Benign neoplasm of spinal meninges | | | | | | | | | | | | | | | | | | |  |  |
| D329 | | |  | | | |  | | Benign neoplasm of meninges, unspecified | | | | | | | | | | | | | | | | | | |  |  |
| D330 | | |  | | | |  | | Benign neoplasm of brain, supratentorial | | | | | | | | | | | | | | | | | | |  |  |
| D331 | | |  | | | |  | | Benign neoplasm of brain, infratentorial | | | | | | | | | | | | | | | | | | |  |  |
| D332 | | |  | | | |  | | Benign neoplasm of brain, unspecified | | | | | | | | | | | | | | | | | | |  |  |
| D333 | | |  | | | |  | | Benign neoplasm of cranial nerves | | | | | | | | | | | | | | | | | | |  |  |
| D337 | | |  | | | |  | | Benign neoplasm of other specified parts of central nervous system | | | | | | | | | | | | | | | | | | |  |  |
| D339 | | |  | | | |  | | Benign neoplasm of central nervous system, unspecified | | | | | | | | | | | | | | | | | | |  |  |
| D420 | | |  | | | |  | | Neoplasm of uncertain behavior of cerebral meninges | | | | | | | | | | | | | | | | | | |  |  |
| D430 | | |  | | | |  | | Neoplasm of uncertain behavior of brain, supratentorial | | | | | | | | | | | | | | | | | | |  |  |
| D431 | | |  | | | |  | | Neoplasm of uncertain behavior of brain, infratentorial | | | | | | | | | | | | | | | | | | |  |  |
| D432 | | |  | | | |  | | Neoplasm of uncertain behavior of brain, unspecified | | | | | | | | | | | | | | | | | | |  |  |
| D433 | | |  | | | |  | | Neoplasm of uncertain behavior of cranial nerves | | | | | | | | | | | | | | | | | | |  |  |
| D438 | | |  | | | |  | | Neoplasm of uncertain behavior of other specified parts of central nervous system | | | | | | | | | | | | | | | | | | |  |  |
| D439 | | |  | | | |  | | Neoplasm of uncertain behavior of central nervous system, unspecified | | | | | | | | | | | | | | | | | | |  |  |
| D496 | | |  | | | |  | | Neoplasm of unspecified behavior of brain | | | | | | | | | | | | | | | | | | |  |  |
| V1085 | | |  | | | |  | | Personal history of malignant neoplasm of brain | | | | | | | | | | | | | | | | | | |  |  |
| V1086 | | |  | | | |  | | Personal history of malignant neoplasm of other parts of nervous system | | | | | | | | | | | | | | | | | | |  |  |
| V1241 | | |  | | | |  | | Personal history of benign neoplasm of the brain | | | | | | | | | | | | | | | | | | |  |  |
| V1242 | | |  | | | |  | | Personal history of infections of the central nervous system | | | | | | | | | | | | | | | | | | |  |  |
| Z1282 | | |  | | | |  | | Encounter for screening for malignant neoplasm of nervous system | | | | | | | | | | | | | | | | | | |  |  |
| Z85841 | | |  | | | |  | | Personal history of malignant neoplasm of brain | | | | | | | | | | | | | | | | | | |  |  |
| Z8603 | | |  | | | |  | | Personal history of neoplasm of uncertain behavior | | | | | | | | | | | | | | | | | | |  |  |

| 34831 |  | Metabolic encephalopathy |
| --- | --- | --- |
| 5722 |  | Hepatic encephalopathy |
| 700 |  | Viral hepatitis A with hepatic coma |
| 7020 |  | Viral hepatitis B with hepatic coma, acute or unspecified, without mention of hepatitis delta |
| 7021 |  | Viral hepatitis B with hepatic coma, acute or unspecified, with hepatitis delta |
| 7022 |  | Chronic viral hepatitis B with hepatic coma without hepatitis delta |
| 7023 |  | Chronic viral hepatitis B with hepatic coma with hepatitis delta |
| 7041 |  | Acute hepatitis C with hepatic coma |
| 7042 |  | Hepatitis delta without mention of active hepatitis B disease with hepatic coma |
| 7043 |  | Hepatitis E with hepatic coma |
| 7044 |  | Chronic hepatitis C with hepatic coma |
| 7049 |  | Other specified viral hepatitis with hepatic coma |
| 7052 |  | Hepatitis delta without mention of active hepatitis B disease or hepatic coma |
| 706 |  | Unspecified viral hepatitis with hepatic coma |
| 7071 |  | Unspecified viral hepatitis C with hepatic coma |
| 2706 |  | Disorders of urea cycle metabolism |
| 2510 |  | Hypoglycemic coma |
| 4372 |  | Hypertensive encephalopathy |
| G9341 |  | Metabolic encephalopathy |
| B150 |  | Hepatitis A with hepatic coma |
| B159 |  | Hepatitis A without hepatic coma |
| B160 |  | Acute hepatitis B with delta-agent with hepatic coma |
| B161 |  | Acute hepatitis B with delta-agent without hepatic coma |
| B162 |  | Acute hepatitis B without delta-agent with hepatic coma |
| B169 |  | Acute hepatitis B without delta-agent and without hepatic coma |
| B170 |  | Acute delta-(super) infection of hepatitis B carrier |
| B1710 |  | Acute hepatitis C without hepatic coma |
| B1711 |  | Acute hepatitis C with hepatic coma |
| B190 |  | Unspecified viral hepatitis with hepatic coma |
| B1910 |  | Unspecified viral hepatitis B without hepatic coma |
| B1911 |  | Unspecified viral hepatitis B with hepatic coma |
| B1920 |  | Unspecified viral hepatitis C without hepatic coma |
| B1921 |  | Unspecified viral hepatitis C with hepatic coma |
| B199 |  | Unspecified viral hepatitis without hepatic coma |
| E7220 |  | Disorder of urea cycle metabolism, unspecified |
| E7229 |  | Other disorders of urea cycle metabolism |
| E15 |  | Nondiabetic hypoglycemic coma |
| E160 |  | Drug-induced hypoglycemia without coma |
| E161 |  | Other hypoglycemia |
| E162 |  | Hypoglycemia, unspecified |
| E512 |  | Wernicke's encephalopathy |
| G92 |  | Toxic encephalopathy |
| G9341 |  | Metabolic encephalopathy |
| I674 |  | Hypertensive encephalopathy |
| P9160 |  | Hypoxic ischemic encephalopathy [HIE], unspecified |
| P9161 |  | Mild hypoxic ischemic encephalopathy [HIE] |
| P9162 |  | Moderate hypoxic ischemic encephalopathy [HIE] |
| P9163 |  | Severe hypoxic ischemic encephalopathy [HIE] |

| 29634 |  |  | Major depressive affective disorder, recurrent episode, severe, specified as with psychotic behavior |  |  |
| --- | --- | --- | --- | --- | --- |
| 29635 |  |  | Major depressive affective disorder, recurrent episode, in partial or unspecified remission |  |  |
| 29636 |  |  | Major depressive affective disorder, recurrent episode, in full remission |  |  |
| 29640 |  |  | Bipolar I disorder, most recent episode (or current) manic, unspecified |  |  |
| 29641 |  |  | Bipolar I disorder, most recent episode (or current) manic, mild |  |  |
| 29642 |  |  | Bipolar I disorder, most recent episode (or current) manic, moderate |  |  |
| 29643 |  |  | Bipolar I disorder, most recent episode (or current) manic, severe, without mention of psychotic behavior |  |  |
| 29644 |  |  | Bipolar I disorder, most recent episode (or current) manic, severe, specified as with psychotic behavior |  |  |
| 29645 |  |  | Bipolar I disorder, most recent episode (or current) manic, in partial or unspecified remission |  |  |
| 29646 |  |  | Bipolar I disorder, most recent episode (or current) manic, in full remission |  |  |
| 29650 |  |  | Bipolar I disorder, most recent episode (or current) depressed, unspecified |  |  |
| 29651 |  |  | Bipolar I disorder, most recent episode (or current) depressed, mild |  |  |
| 29652 |  |  | Bipolar I disorder, most recent episode (or current) depressed, moderate |  |  |
| 29653 |  |  | Bipolar I disorder, most recent episode (or current) depressed, severe, without mention of psychotic behavior |  |  |
| 29654 |  |  | Bipolar I disorder, most recent episode (or current) depressed, severe, specified as with psychotic behavior |  |  |
| 29655 |  |  | Bipolar I disorder, most recent episode (or current) depressed, in partial or unspecified remission |  |  |
| 29656 |  |  | Bipolar I disorder, most recent episode (or current) depressed, in full remission |  |  |
| 29660 |  |  | Bipolar I disorder, most recent episode (or current) mixed, unspecified |  |  |
| 29661 |  |  | Bipolar I disorder, most recent episode (or current) mixed, mild |  |  |
| 29662 |  |  | Bipolar I disorder, most recent episode (or current) mixed, moderate |  |  |
| 29620 |  |  | Major depressive affective disorder, single episode, unspecified |  |  |
| 29621 |  |  | Major depressive affective disorder, single episode, mild |  |  |
| 29622 |  |  | Major depressive affective disorder, single episode, moderate |  |  |
| 29623 |  |  | Major depressive affective disorder, single episode, severe, without mention of psychotic behavior |  |  |
| 29624 |  |  | Major depressive affective disorder, single episode, severe, specified as with psychotic behavior |  |  |
| 29625 |  |  | Major depressive affective disorder, single episode, in partial or unspecified remission |  |  |
| 29626 |  |  | Major depressive affective disorder, single episode, in full remission |  |  |
| 29630 |  |  | Major depressive affective disorder, recurrent episode, unspecified |  |  |
| 29631 |  |  | Major depressive affective disorder, recurrent episode, mild |  |  |
| 29632 |  |  | Major depressive affective disorder, recurrent episode, moderate |  |  |
| 29633 |  |  | Major depressive affective disorder, recurrent episode, severe, without mention of psychotic behavior |  |  |
| 29634 |  |  | Major depressive affective disorder, recurrent episode, severe, specified as with psychotic behavior |  |  |
| 29635 |  |  | Major depressive affective disorder, recurrent episode, in partial or unspecified remission |  |  |
| 29636 |  |  | Major depressive affective disorder, recurrent episode, in full remission |  |  |
| 29663 |  |  | Bipolar I disorder, most recent episode (or current) mixed, severe, without mention of psychotic behavior |  |  |
| 29664 |  |  | Bipolar I disorder, most recent episode (or current) mixed, severe, specified as with psychotic behavior |  |  |
| 29665 |  |  | Bipolar I disorder, most recent episode (or current) mixed, in partial or unspecified remission |  |  |
| 29666 |  |  | Bipolar I disorder, most recent episode (or current) mixed, in full remission |  |  |
| 2967 |  |  | Bipolar I disorder, most recent episode (or current) unspecified |  |  |
| 29680 |  |  | Bipolar disorder, unspecified |  |  |
| 29681 |  |  | Atypical manic disorder |  |  |
| 29682 |  |  | Atypical depressive disorder |  |  |
| 29689 |  |  | Other bipolar disorders |  |  |
| 30289 |  |  | Other specified psychosexual disorders |  |  |
| 3029 |  |  | Unspecified psychosexual disorder |  |  |
| 29682 |  |  | Atypical depressive disorder |  |  |
| 2971 |  |  | Delusional disorder |  |  |
| 2972 |  |  | Paraphrenia |  |  |
| 2980 |  |  | Depressive type psychosis |  |  |
| 2981 |  |  | Excitative type psychosis |  |  |
| 2982 |  |  | Reactive confusion |  |  |
| 2983 |  |  | Acute paranoid reaction |  |  |
| 2984 |  |  | Psychogenic paranoid psychosis |  |  |
| 2988 |  |  | Other and unspecified reactive psychosis |  |  |
| 2989 |  |  | Unspecified psychosis |  |  |
| 30111 |  |  | Chronic hypomanic personality disorder |  |  |
| 30020 |  |  | Phobia, unspecified |  |  |
| 30021 |  |  | Agoraphobia with panic disorder |  |  |
| 30022 |  |  | Agoraphobia without mention of panic attacks |  |  |
| 30023 |  |  | Social phobia |  |  |
| 30029 |  |  | Other isolated or specific phobias |  |  |
| 30112 |  |  | Chronic depressive personality disorder |  |  |
| 30113 |  |  | Cyclothymic disorder |  |  |
| 30120 |  |  | Schizoid personality disorder, unspecified |  |  |
| 29381 |  |  | Psychotic disorder with delusions in conditions classified elsewhere |  |  |
| 29410 |  |  | Dementia in conditions classified elsewhere without behavioral disturbance |  |  |
| 29411 |  |  | Dementia in conditions classified elsewhere with behavioral disturbance |  |  |
| 29420 |  |  | Dementia, unspecified, without behavioral disturbance |  |  |
| 29421 |  |  | Dementia, unspecified, with behavioral disturbance |  |  |
| 2949 |  |  | Unspecified persistent mental disorders due to conditions classified elsewhere |  |  |
| 29500 |  |  | Simple type schizophrenia, unspecified |  |  |
| 29501 |  |  | Simple type schizophrenia, subchronic |  |  |
| 29502 |  |  | Simple type schizophrenia, chronic |  |  |
| 29503 |  |  | Simple type schizophrenia, subchronic with acute exacerbation |  |  |
| 29504 |  |  | Simple type schizophrenia, chronic with acute exacerbation |  |  |
| 29505 |  |  | Simple type schizophrenia, in remission |  |  |
| 29510 |  |  | Disorganized type schizophrenia, unspecified |  |  |
| 29511 |  |  | Disorganized type schizophrenia, subchronic |  |  |
| 29512 |  |  | Disorganized type schizophrenia, chronic |  |  |
| 29513 |  |  | Disorganized type schizophrenia, subchronic with acute exacerbation |  |  |
| 29514 |  |  | Disorganized type schizophrenia, chronic with acute exacerbation |  |  |
| 29515 |  |  | Disorganized type schizophrenia, in remission |  |  |
| 29520 |  |  | Catatonic type schizophrenia, unspecified |  |  |
| 29521 |  |  | Catatonic type schizophrenia, subchronic |  |  |
| 29522 |  |  | Catatonic type schizophrenia, chronic |  |  |
| 29523 |  |  | Catatonic type schizophrenia, subchronic with acute exacerbation |  |  |
| 29524 |  |  | Catatonic type schizophrenia, chronic with acute exacerbation |  |  |
| 29525 |  |  | Catatonic type schizophrenia, in remission |  |  |
| 29530 |  |  | Paranoid type schizophrenia, unspecified |  |  |
| 29531 |  |  | Paranoid type schizophrenia, subchronic |  |  |
| 29532 |  |  | Paranoid type schizophrenia, chronic |  |  |
| 29533 |  |  | Paranoid type schizophrenia, subchronic with acute exacerbation |  |  |
| 29534 |  |  | Paranoid type schizophrenia, chronic with acute exacerbation |  |  |
| 29535 |  |  | Paranoid type schizophrenia, in remission |  |  |
| 29540 |  |  | Schizophreniform disorder, unspecified |  |  |
| 29541 |  |  | Schizophreniform disorder, subchronic |  |  |
| 29542 |  |  | Schizophreniform disorder, chronic |  |  |
| 29543 |  |  | Schizophreniform disorder, subchronic with acute exacerbation |  |  |
| 29544 |  |  | Schizophreniform disorder, chronic with acute exacerbation |  |  |
| 29545 |  |  | Schizophreniform disorder, in remission |  |  |
| 29550 |  |  | Latent schizophrenia, unspecified |  |  |
| 29551 |  |  | Latent schizophrenia, unspecified |  |  |
| 29552 |  |  | Latent schizophrenia, chronic |  |  |
| 29553 |  |  | Latent schizophrenia, subchronic with acute exacerbation |  |  |
| 29554 |  |  | Latent schizophrenia, chronic with acute exacerbation |  |  |
| 29555 |  |  | Latent schizophrenia, in remission |  |  |
| 29560 |  |  | Schizophrenic disorders, residual type, unspecified |  |  |
| 29561 |  |  | Schizophrenic disorders, residual type, subchronic |  |  |
| 29562 |  |  | Schizophrenic disorders, residual type, chronic |  |  |
| 29563 |  |  | Schizophrenic disorders, residual type, subchronic with acute exacerbation |  |  |
| 29564 |  |  | Schizophrenic disorders, residual type, chronic with acute exacerbation |  |  |
| 29565 |  |  | Schizophrenic disorders, residual type, in remission |  |  |
| 29570 |  |  | Schizoaffective disorder, unspecified |  |  |
| 29571 |  |  | Schizoaffective disorder, subchronic |  |  |
| 29572 |  |  | Schizoaffective disorder, chronic |  |  |
| 29573 |  |  | Schizoaffective disorder, subchronic with acute exacerbation |  |  |
| 29574 |  |  | Schizoaffective disorder, chronic with acute exacerbation |  |  |
| 29580 |  |  | Other specified types of schizophrenia, unspecified |  |  |
| 29581 |  |  | Other specified types of schizophrenia, subchronic |  |  |
| 29582 |  |  | Other specified types of schizophrenia, chronic |  |  |
| 29583 |  |  | Other specified types of schizophrenia, subchronic with acute exacerbation |  |  |
| 29584 |  |  | Other specified types of schizophrenia, chronic with acute exacerbation |  |  |
| 29585 |  |  | Other specified types of schizophrenia, in remission |  |  |
| 29590 |  |  | Unspecified schizophrenia, unspecified |  |  |
| 29591 |  |  | Unspecified schizophrenia, subchronic |  |  |
| 29592 |  |  | Unspecified schizophrenia, chronic |  |  |
| 29593 |  |  | Unspecified schizophrenia, subchronic with acute exacerbation |  |  |
| 29594 |  |  | Unspecified schizophrenia, chronic with acute exacerbation |  |  |
| 29595 |  |  | Unspecified schizophrenia, in remission |  |  |
| 29600 |  |  | Bipolar I disorder, single manic episode, unspecified |  |  |
| 29601 |  |  | Bipolar I disorder, single manic episode, mild |  |  |
| 29602 |  |  | Bipolar I disorder, single manic episode, moderate |  |  |
| 29603 |  |  | Bipolar I disorder, single manic episode, severe, without mention of psychotic behavior |  |  |
| 29604 |  |  | Bipolar I disorder, single manic episode, severe, specified as with psychotic behavior |  |  |
| 29605 |  |  | Bipolar I disorder, single manic episode, in partial or unspecified remission |  |  |
| 29606 |  |  | Bipolar I disorder, single manic episode, in full remission |  |  |
| 29610 |  |  | Manic affective disorder, recurrent episode, unspecified |  |  |
| 29611 |  |  | Manic affective disorder, recurrent episode, mild |  |  |
| 29612 |  |  | Manic affective disorder, recurrent episode, moderate |  |  |
| 29613 |  |  | Manic affective disorder, recurrent episode, severe, without mention of psychotic behavior |  |  |
| 29614 |  |  | Manic affective disorder, recurrent episode, severe, specified as with psychotic behavior |  |  |
| 29615 |  |  | Manic affective disorder, recurrent episode, in partial or unspecified remission |  |  |
| 29616 |  |  | Manic affective disorder, recurrent episode, in full remission |  |  |
| 29620 |  |  | Major depressive affective disorder, single episode, unspecified |  |  |
| 29621 |  |  | Major depressive affective disorder, single episode, mild |  |  |
| 29622 |  |  | Major depressive affective disorder, single episode, moderate |  |  |
| 29623 |  |  | Major depressive affective disorder, single episode, severe, without mention of psychotic behavior |  |  |
| 29624 |  |  | Major depressive affective disorder, single episode, severe, specified as with psychotic behavior |  |  |
| 29625 |  |  | Major depressive affective disorder, single episode, in partial or unspecified remission |  |  |
| 29626 |  |  | Major depressive affective disorder, single episode, in full remission |  |  |
| 29630 |  |  | Major depressive affective disorder, recurrent episode, unspecified |  |  |
| 29631 |  |  | Major depressive affective disorder, recurrent episode, mild |  |  |
| 29632 |  |  | Major depressive affective disorder, recurrent episode, moderate |  |  |
| 29633 |  |  | Major depressive affective disorder, recurrent episode, severe, without mention of psychotic behavior |  |  |
| 33182 |  |  | Dementia with lewy bodies |  |  |
| 3310 |  |  | Alzheimer's disease |  |  |
| 33119 |  |  | Other frontotemporal dementia |  |  |
| 64842 |  |  | Mental disorders of mother, delivered, with mention of postpartum complication |  |  |
| 64843 |  |  | Mental disorders of mother, antepartum condition or complication |  |  |
| 64844 |  |  | Mental disorders of mother, postpartum condition or complication |  |  |
| 3181 |  |  | Severe intellectual disabilities |  |  |
| 3182 |  |  | Profound intellectual disabilities |  |  |
| 33182 |  |  | Dementia with lewy bodies |  |  |
| F0150 |  |  | Vascular dementia without behavioral disturbance |  |  |
| F0151 |  |  | Vascular dementia with behavioral disturbance |  |  |
| F0280 |  |  | Dementia in other diseases classified elsewhere without behavioral disturbance |  |  |
| F0281 |  |  | Dementia in other diseases classified elsewhere with behavioral disturbance |  |  |
| F0390 |  |  | Unspecified dementia without behavioral disturbance |  |  |
| F0391 |  |  | Unspecified dementia with behavioral disturbance |  |  |
| F04 |  |  | Amnestic disorder due to known physiological condition |  |  |
| F05 |  |  | Delirium due to known physiological condition |  |  |
| F060 |  |  | Psychotic disorder with hallucinations due to known physiological condition |  |  |
| F061 |  |  | Catatonic disorder due to known physiological condition |  |  |
| F062 |  |  | Psychotic disorder with delusions due to known physiological condition |  |  |
| F0630 |  |  | Mood disorder due to known physiological condition, unspecified |  |  |
| F0631 |  |  | Mood disorder due to known physiological condition with depressive features |  |  |
| F0632 |  |  | Mood disorder due to known physiological condition with major depressive-like episode |  |  |
| F0633 |  |  | Mood disorder due to known physiological condition with manic features |  |  |
| F0634 |  |  | Mood disorder due to known physiological condition with mixed features |  |  |
| F064 |  |  | Anxiety disorder due to known physiological condition |  |  |
| F068 |  |  | Other specified mental disorders due to known physiological condition |  |  |
| F070 |  |  | Personality change due to known physiological condition |  |  |
| F0781 |  |  | Postconcussional syndrome |  |  |
| F0789 |  |  | Other personality and behavioral disorders due to known physiological condition |  |  |
| F079 |  |  | Unspecified personality and behavioral disorder due to known physiological condition |  |  |
| F09 |  |  | Unspecified mental disorder due to known physiological condition |  |  |
| F200 |  |  | Paranoid schizophrenia |  |  |
| F201 |  |  | Disorganized schizophrenia |  |  |
| F202 |  |  | Catatonic schizophrenia |  |  |
| F203 |  |  | Undifferentiated schizophrenia |  |  |
| F205 |  |  | Residual schizophrenia |  |  |
| F2081 |  |  | Schizophreniform disorder |  |  |
| F2089 |  |  | Other schizophrenia |  |  |
| F209 |  |  | Schizophrenia, unspecified |  |  |
| F21 |  |  | Schizotypal disorder |  |  |
| F22 |  |  | Delusional disorders |  |  |
| F23 |  |  | Brief psychotic disorder |  |  |
| F24 |  |  | Shared psychotic disorder |  |  |
| F250 |  |  | Schizoaffective disorder, bipolar type |  |  |
| F251 |  |  | Schizoaffective disorder, depressive type |  |  |
| F258 |  |  | Other schizoaffective disorders |  |  |
| F259 |  |  | Schizoaffective disorder, unspecified |  |  |
| F28 |  |  | Other psychotic disorder not due to a substance or known physiological condition |  |  |
| F29 |  |  | Unspecified psychosis not due to a substance or known physiological condition |  |  |
| F3010 |  |  | Manic episode without psychotic symptoms, unspecified |  |  |
| F3011 |  |  | Manic episode without psychotic symptoms, mild |  |  |
| F3012 |  |  | Manic episode without psychotic symptoms, moderate |  |  |
| F3013 |  |  | Manic episode, severe, without psychotic symptoms |  |  |
| F302 |  |  | Manic episode, severe with psychotic symptoms |  |  |
| F303 |  |  | Manic episode in partial remission |  |  |
| F304 |  |  | Manic episode in full remission |  |  |
| F308 |  |  | Other manic episodes |  |  |
| F309 |  |  | Manic episode, unspecified |  |  |
| F310 |  |  | Bipolar disorder, current episode hypomanic |  |  |
| F3110 |  |  | Bipolar disorder, current episode manic without psychotic features, unspecified |  |  |
| F3111 |  |  | Bipolar disorder, current episode manic without psychotic features, mild |  |  |
| F3112 |  |  | Bipolar disorder, current episode manic without psychotic features, moderate |  |  |
| F3113 |  |  | Bipolar disorder, current episode manic without psychotic features, severe |  |  |
| F312 |  |  | Bipolar disorder, current episode manic severe with psychotic features |  |  |
| F3130 |  |  | Bipolar disorder, current episode depressed, mild or moderate severity, unspecified |  |  |
| F3131 |  |  | Bipolar disorder, current episode depressed, mild |  |  |
| F3132 |  |  | Bipolar disorder, current episode depressed, moderate |  |  |
| F314 |  |  | Bipolar disorder, current episode depressed, severe, without psychotic features |  |  |
| F315 |  |  | Bipolar disorder, current episode depressed, severe, with psychotic features |  |  |
| F3160 |  |  | Bipolar disorder, current episode mixed, unspecified |  |  |
| F3161 |  |  | Bipolar disorder, current episode mixed, mild |  |  |
| F3162 |  |  | Bipolar disorder, current episode mixed, moderate |  |  |
| F3163 |  |  | Bipolar disorder, current episode mixed, severe, without psychotic features |  |  |
| F3164 |  |  | Bipolar disorder, current episode mixed, severe, with psychotic features |  |  |
| F3170 |  |  | Bipolar disorder, currently in remission, most recent episode unspecified |  |  |
| F3171 |  |  | Bipolar disorder, in partial remission, most recent episode hypomanic |  |  |
| F3172 |  |  | Bipolar disorder, in full remission, most recent episode hypomanic |  |  |
| F3173 |  |  | Bipolar disorder, in partial remission, most recent episode manic |  |  |
| F3174 |  |  | Bipolar disorder, in full remission, most recent episode manic |  |  |
| F3175 |  |  | Bipolar disorder, in partial remission, most recent episode depressed |  |  |
| F3176 |  |  | Bipolar disorder, in full remission, most recent episode depressed |  |  |
| F3177 |  |  | Bipolar disorder, in partial remission, most recent episode mixed |  |  |
| F3178 |  |  | Bipolar disorder, in full remission, most recent episode mixed |  |  |
| F3181 |  |  | Bipolar II disorder |  |  |
| F3189 |  |  | Other bipolar disorder |  |  |
| F319 |  |  | Bipolar disorder, unspecified |  |  |
| F320 |  |  | Major depressive disorder, single episode, mild |  |  |
| F321 |  |  | Major depressive disorder, single episode, moderate |  |  |
| F322 |  |  | Major depressive disorder, single episode, severe without psychotic features |  |  |
| F323 |  |  | Major depressive disorder, single episode, severe with psychotic features |  |  |
| F324 |  |  | Major depressive disorder, single episode, in partial remission |  |  |
| F325 |  |  | Major depressive disorder, single episode, in full remission |  |  |
| F3281 |  |  | Premenstrual dysphoric disorder |  |  |
| F3289 |  |  | Other specified depressive episodes |  |  |
| F329 |  |  | Major depressive disorder, single episode, unspecified |  |  |
| F330 |  |  | Major depressive disorder, recurrent, mild |  |  |
| F331 |  |  | Major depressive disorder, recurrent, moderate |  |  |
| F332 |  |  | Major depressive disorder, recurrent severe without psychotic features |  |  |
| F333 |  |  | Major depressive disorder, recurrent, severe with psychotic symptoms |  |  |
| F3340 |  |  | Major depressive disorder, recurrent, in remission, unspecified |  |  |
| F3341 |  |  | Major depressive disorder, recurrent, in partial remission |  |  |
| F3342 |  |  | Major depressive disorder, recurrent, in full remission |  |  |
| F338 |  |  | Other recurrent depressive disorders |  |  |
| F340 |  |  | Cyclothymic disorder |  |  |
| F341 |  |  | Dysthymic disorder |  |  |
| F3481 |  |  | Disruptive mood dysregulation disorder |  |  |
| F3489 |  |  | Other specified persistent mood disorders |  |  |
| F349 |  |  | Persistent mood [affective] disorder, unspecified |  |  |
| F39 |  |  | Unspecified mood [affective] disorder |  |  |
| F4000 |  |  | Agoraphobia, unspecified |  |  |
| F4001 |  |  | Agoraphobia with panic disorder |  |  |
| F4002 |  |  | Agoraphobia without panic disorder |  |  |
| F4010 |  |  | Social phobia, unspecified |  |  |
| F4011 |  |  | Social phobia, generalized |  |  |
| F40248 |  |  | Other situational type phobia |  |  |
| F40290 |  |  | Androphobia |  |  |
| F40291 |  |  | Gynephobia |  |  |
| F40298 |  |  | Other specified phobia |  |  |
| F408 |  |  | Other phobic anxiety disorders |  |  |
| F409 |  |  | Phobic anxiety disorder, unspecified |  |  |
| F410 |  |  | Panic disorder [episodic paroxysmal anxiety] |  |  |
| F411 |  |  | Generalized anxiety disorder |  |  |
| F413 |  |  | Other mixed anxiety disorders |  |  |
| F418 |  |  | Other specified anxiety disorders |  |  |
| F419 |  |  | Anxiety disorder, unspecified |  |  |
| F422 |  |  | Mixed obsessional thoughts and acts |  |  |
| F423 |  |  | Hoarding disorder |  |  |
| F424 |  |  | Excoriation (skin-picking) disorder |  |  |
| F428 |  |  | Other obsessive-compulsive disorder |  |  |
| F429 |  |  | Obsessive-compulsive disorder, unspecified |  |  |
| F430 |  |  | Acute stress reaction |  |  |
| F4310 |  |  | Post-traumatic stress disorder, unspecified |  |  |
| F4311 |  |  | Post-traumatic stress disorder, acute |  |  |
| F4312 |  |  | Post-traumatic stress disorder, chronic |  |  |
| F4320 |  |  | Adjustment disorder, unspecified |  |  |
| F4321 |  |  | Adjustment disorder with depressed mood |  |  |
| F4322 |  |  | Adjustment disorder with anxiety |  |  |
| F4323 |  |  | Adjustment disorder with mixed anxiety and depressed mood |  |  |
| F4324 |  |  | Adjustment disorder with disturbance of conduct |  |  |
| F4325 |  |  | Adjustment disorder with mixed disturbance of emotions and conduct |  |  |
| F4329 |  |  | Adjustment disorder with other symptoms |  |  |
| F70 |  |  | Mild intellectual disabilities |  |  |
| F71 |  |  | Moderate intellectual disabilities |  |  |
| F72 |  |  | Severe intellectual disabilities |  |  |
| F73 |  |  | Profound intellectual disabilities |  |  |
| F78 |  |  | Other intellectual disabilities |  |  |
| F79 |  |  | Unspecified intellectual disabilities |  |  |
| F800 |  |  | Phonological disorder |  |  |
| F801 |  |  | Expressive language disorder |  |  |
| F802 |  |  | Mixed receptive-expressive language disorder |  |  |
| F804 |  |  | Speech and language development delay due to hearing loss |  |  |
| F8081 |  |  | Childhood onset fluency disorder |  |  |
| F8082 |  |  | Social pragmatic communication disorder |  |  |
| F8089 |  |  | Other developmental disorders of speech and language |  |  |
| F809 |  |  | Developmental disorder of speech and language, unspecified |  |  |
| F812 |  |  | Mathematics disorder |  |  |
| F8181 |  |  | Disorder of written expression |  |  |
| G300 |  |  | Alzheimer's disease with early onset |  |  |
| G301 |  |  | Alzheimer's disease with late onset |  |  |
| G308 |  |  | Other Alzheimer's disease |  |  |
| G309 |  |  | Alzheimer's disease, unspecified |  |  |
| G3101 |  |  | Pick's disease |  |  |
| G3109 |  |  | Other frontotemporal dementia |  |  |
| G311 |  |  | Senile degeneration of brain, not elsewhere classified |  |  |
| G312 |  |  | Degeneration of nervous system due to alcohol |  |  |
| G3101 |  |  | Pick's disease |  |  |
| G3109 |  |  | Other frontotemporal dementia |  |  |
| G311 |  |  | Senile degeneration of brain, not elsewhere classified |  |  |
| G312 |  |  | Degeneration of nervous system due to alcohol |  |  |
| G3181 |  |  | Alpers disease |  |  |
| G3182 |  |  | Leigh's disease |  |  |
| G3183 |  |  | Dementia with Lewy bodies |  |  |
| G360 |  |  | Neuromyelitis optica [Devic] |  |  |
| G361 |  |  | Acute and subacute hemorrhagic leukoencephalitis [Hurst] |  |  |
| G368 |  |  | Other specified acute disseminated demyelination |  |  |
| G369 |  |  | Acute disseminated demyelination, unspecified |  |  |
| G370 |  |  | Diffuse sclerosis of central nervous system |  |  |
| G371 |  |  | Central demyelination of corpus callosum |  |  |
| G372 |  |  | Central pontine myelinolysis |  |  |
| G373 |  |  | Acute transverse myelitis in demyelinating disease of central nervous system |  |  |
| G374 |  |  | Subacute necrotizing myelitis of central nervous system |  |  |
| G375 |  |  | Concentric sclerosis [Balo] of central nervous system |  |  |
| G378 |  |  | Other specified demyelinating diseases of central nervous system |  |  |
| G379 |  |  | Demyelinating disease of central nervous system, unspecified |  |  |

| 30303 |  | Acute alcoholic intoxication in alcoholism, in remission |
| --- | --- | --- |
| 30390 |  | Other and unspecified alcohol dependence, unspecified |
| 30391 |  | Other and unspecified alcohol dependence, continuous |
| 30392 |  | Other and unspecified alcohol dependence, episodic |
| 30393 |  | Other and unspecified alcohol dependence, in remission |
| 30400 |  | Opioid type dependence, unspecified |
| 30401 |  | Opioid type dependence, continuous |
| 30300 |  | Acute alcoholic intoxication in alcoholism, unspecified |
| 30301 |  | Acute alcoholic intoxication in alcoholism, continuous |
| 30302 |  | Acute alcoholic intoxication in alcoholism, episodic |
| 30402 |  | Opioid type dependence, episodic |
| 30403 |  | Opioid type dependence, in remission |
| 30410 |  | Sedative, hypnotic or anxiolytic dependence, unspecified |
| 30411 |  | Sedative, hypnotic or anxiolytic dependence, continuous |
| 30412 |  | Sedative, hypnotic or anxiolytic dependence, episodic |
| 30413 |  | Sedative, hypnotic or anxiolytic dependence, in remission |
| 30420 |  | Cocaine dependence, unspecified |
| 30421 |  | Cocaine dependence, continuous |
| 30422 |  | Cocaine dependence, episodic |
| 30423 |  | Cocaine dependence, in remission |
| 30430 |  | Cannabis dependence, unspecified |
| 30431 |  | Cannabis dependence, continuous |
| 30432 |  | Cannabis dependence, episodic |
| 30433 |  | Cannabis dependence, in remission |
| 30440 |  | Amphetamine and other psychostimulant dependence, unspecified |
| 30441 |  | Amphetamine and other psychostimulant dependence, continuous |
| 30442 |  | Amphetamine and other psychostimulant dependence, episodic |
| 30443 |  | Amphetamine and other psychostimulant dependence, in remission |
| 30450 |  | Hallucinogen dependence, unspecified |
| 30451 |  | Hallucinogen dependence, continuous |
| 30452 |  | Hallucinogen dependence, episodic |
| 30453 |  | Hallucinogen dependence, in remission |
| 30460 |  | Other specified drug dependence, unspecified |
| Z7141 |  | Alcohol abuse counseling and surveillance of alcoholic |
| Z811 |  | Family history of alcohol abuse and dependence |
| 2910 |  | Alcohol withdrawal delirium |
| 2911 |  | Alcohol-induced persisting amnestic disorder |
| 2912 |  | Alcohol-induced persisting dementia |
| 2913 |  | Alcohol-induced psychotic disorder with hallucinations |
| 2914 |  | Idiosyncratic alcohol intoxication |
| 2915 |  | Alcohol-induced psychotic disorder with delusions |
| 29181 |  | Alcohol withdrawal |
| 29182 |  | Alcohol induced sleep disorders |
| 29189 |  | Other alcohol-induced mental disorders |
| 2919 |  | Unspecified alcohol-induced mental disorders |
| 3051 |  | Tobacco use disorder |
| 30520 |  | Cannabis abuse, unspecified |
| 30521 |  | Cannabis abuse, continuous |
| 30522 |  | Cannabis abuse, episodic |
| 30523 |  | Cannabis abuse, in remission |
| 30530 |  | Hallucinogen abuse, unspecified |
| 30531 |  | Hallucinogen abuse, continuous |
| 30532 |  | Hallucinogen abuse, episodic |
| 30533 |  | Hallucinogen abuse, in remission |
| 30540 |  | Sedative, hypnotic or anxiolytic abuse, unspecified |
| 30541 |  | Sedative, hypnotic or anxiolytic abuse, continuous |
| 30542 |  | Sedative, hypnotic or anxiolytic abuse, episodic |
| 30543 |  | Sedative, hypnotic or anxiolytic abuse, in remission |
| 30550 |  | Opioid abuse, unspecified |
| 30551 |  | Opioid abuse, continuous |
| 30552 |  | Opioid abuse, episodic |
| 30553 |  | Opioid abuse, in remission |
| 30560 |  | Cocaine abuse, unspecified |
| 30561 |  | Cocaine abuse, continuous |
| 30562 |  | Cocaine abuse, episodic |
| 30563 |  | Cocaine abuse, in remission |
| 30570 |  | Amphetamine or related acting sympathomimetic abuse, unspecified |
| 30571 |  | Amphetamine or related acting sympathomimetic abuse, continuous |
| 30572 |  | Amphetamine or related acting sympathomimetic abuse, episodic |
| 30573 |  | Amphetamine or related acting sympathomimetic abuse, in remission |
| 30580 |  | Antidepressant type abuse, unspecified |
| 30581 |  | Antidepressant type abuse, continuous |
| 30582 |  | Antidepressant type abuse, episodic |
| 30583 |  | Antidepressant type abuse, in remission |
| 64900 |  | Tobacco use disorder complicating pregnancy, childbirth, or the puerperium, unspecified as to episode of care or not applicable |
| 64901 |  | Tobacco use disorder complicating pregnancy, childbirth, or the puerperium, delivered, with or without mention of antepartum condition |
| 64902 |  | Tobacco use disorder complicating pregnancy, childbirth, or the puerperium, delivered, with mention of postpartum complication |
| 64903 |  | Tobacco use disorder complicating pregnancy, childbirth, or the puerperium, antepartum condition or complication |
| 64904 |  | Tobacco use disorder complicating pregnancy, childbirth, or the puerperium, postpartum condition or complication |
| F1010 |  | Alcohol abuse, uncomplicated |
| F1011 |  | Alcohol abuse, in remission |
| F10120 |  | Alcohol abuse with intoxication, uncomplicated |
| F10121 |  | Alcohol abuse with intoxication delirium |
| F10129 |  | Alcohol abuse with intoxication, unspecified |
| F1014 |  | Alcohol abuse with alcohol-induced mood disorder |
| F10150 |  | Alcohol abuse with alcohol-induced psychotic disorder with delusions |
| F10151 |  | Alcohol abuse with alcohol-induced psychotic disorder with hallucinations |
| F10159 |  | Alcohol abuse with alcohol-induced psychotic disorder, unspecified |
| F10180 |  | Alcohol abuse with alcohol-induced anxiety disorder |
| F10181 |  | Alcohol abuse with alcohol-induced sexual dysfunction |
| F10182 |  | Alcohol abuse with alcohol-induced sleep disorder |
| F10188 |  | Alcohol abuse with other alcohol-induced disorder |
| F1019 |  | Alcohol abuse with unspecified alcohol-induced disorder |
| F1020 |  | Alcohol dependence, uncomplicated |
| F1021 |  | Alcohol dependence, in remission |
| F10220 |  | Alcohol dependence with intoxication, uncomplicated |
| F10221 |  | Alcohol dependence with intoxication delirium |
| F10229 |  | Alcohol dependence with intoxication, unspecified |
| F10230 |  | Alcohol dependence with withdrawal, uncomplicated |
| F10231 |  | Alcohol dependence with withdrawal delirium |
| F10232 |  | Alcohol dependence with withdrawal with perceptual disturbance |
| F10239 |  | Alcohol dependence with withdrawal, unspecified |
| F1024 |  | Alcohol dependence with alcohol-induced mood disorder |
| F10250 |  | Alcohol dependence with alcohol-induced psychotic disorder with delusions |
| F10251 |  | Alcohol dependence with alcohol-induced psychotic disorder with hallucinations |
| F10259 |  | Alcohol dependence with alcohol-induced psychotic disorder, unspecified |
| F1026 |  | Alcohol dependence with alcohol-induced persisting amnestic disorder |
| F1027 |  | Alcohol dependence with alcohol-induced persisting dementia |
| F10280 |  | Alcohol dependence with alcohol-induced anxiety disorder |
| F10281 |  | Alcohol dependence with alcohol-induced sexual dysfunction |
| F10282 |  | Alcohol dependence with alcohol-induced sleep disorder |
| F10288 |  | Alcohol dependence with other alcohol-induced disorder |
| F1029 |  | Alcohol dependence with unspecified alcohol-induced disorder |
| F10920 |  | Alcohol use, unspecified with intoxication, uncomplicated |
| F10921 |  | Alcohol use, unspecified with intoxication delirium |
| F10929 |  | Alcohol use, unspecified with intoxication, unspecified |
| F1094 |  | Alcohol use, unspecified with alcohol-induced mood disorder |
| F10950 |  | Alcohol use, unspecified with alcohol-induced psychotic disorder with delusions |
| F10951 |  | Alcohol use, unspecified with alcohol-induced psychotic disorder with hallucinations |
| F10959 |  | Alcohol use, unspecified with alcohol-induced psychotic disorder, unspecified |
| F1096 |  | Alcohol use, unspecified with alcohol-induced persisting amnestic disorder |
| F1097 |  | Alcohol use, unspecified with alcohol-induced persisting dementia |
| F10980 |  | Alcohol use, unspecified with alcohol-induced anxiety disorder |
| F10981 |  | Alcohol use, unspecified with alcohol-induced sexual dysfunction |
| F10982 |  | Alcohol use, unspecified with alcohol-induced sleep disorder |
| F10988 |  | Alcohol use, unspecified with other alcohol-induced disorder |
| F1099 |  | Alcohol use, unspecified with unspecified alcohol-induced disorder |
| F1110 |  | Opioid abuse, uncomplicated |
| F1111 |  | Opioid abuse, in remission |
| F11120 |  | Opioid abuse with intoxication, uncomplicated |
| F11121 |  | Opioid abuse with intoxication delirium |
| F11122 |  | Opioid abuse with intoxication with perceptual disturbance |
| F11129 |  | Opioid abuse with intoxication, unspecified |
| F1114 |  | Opioid abuse with opioid-induced mood disorder |
| F11150 |  | Opioid abuse with opioid-induced psychotic disorder with delusions |
| F11151 |  | Opioid abuse with opioid-induced psychotic disorder with hallucinations |
| F11159 |  | Opioid abuse with opioid-induced psychotic disorder, unspecified |
| F11181 |  | Opioid abuse with opioid-induced sexual dysfunction |
| F11182 |  | Opioid abuse with opioid-induced sleep disorder |
| F11188 |  | Opioid abuse with other opioid-induced disorder |
| F1119 |  | Opioid abuse with unspecified opioid-induced disorder |
| F1120 |  | Opioid dependence, uncomplicated |
| F1121 |  | Opioid dependence, in remission |
| F11220 |  | Opioid dependence with intoxication, uncomplicated |
| F11221 |  | Opioid dependence with intoxication delirium |
| F11222 |  | Opioid dependence with intoxication with perceptual disturbance |
| F11229 |  | Opioid dependence with intoxication, unspecified |
| F1123 |  | Opioid dependence with withdrawal |
| F1124 |  | Opioid dependence with opioid-induced mood disorder |
| F11250 |  | Opioid dependence with opioid-induced psychotic disorder with delusions |
| F11251 |  | Opioid dependence with opioid-induced psychotic disorder with hallucinations |
| F11259 |  | Opioid dependence with opioid-induced psychotic disorder, unspecified |
| F11281 |  | Opioid dependence with opioid-induced sexual dysfunction |
| F11282 |  | Opioid dependence with opioid-induced sleep disorder |
| F11288 |  | Opioid dependence with other opioid-induced disorder |
| F1129 |  | Opioid dependence with unspecified opioid-induced disorder |
| F1190 |  | Opioid use, unspecified, uncomplicated |
| F11920 |  | Opioid use, unspecified with intoxication, uncomplicated |
| F11921 |  | Opioid use, unspecified with intoxication delirium |
| F11922 |  | Opioid use, unspecified with intoxication with perceptual disturbance |
| F11929 |  | Opioid use, unspecified with intoxication, unspecified |
| F1193 |  | Opioid use, unspecified with withdrawal |
| F1194 |  | Opioid use, unspecified with opioid-induced mood disorder |
| F11950 |  | Opioid use, unspecified with opioid-induced psychotic disorder with delusions |
| F11951 |  | Opioid use, unspecified with opioid-induced psychotic disorder with hallucinations |
| F11959 |  | Opioid use, unspecified with opioid-induced psychotic disorder, unspecified |
| F11981 |  | Opioid use, unspecified with opioid-induced sexual dysfunction |
| F11982 |  | Opioid use, unspecified with opioid-induced sleep disorder |
| F11988 |  | Opioid use, unspecified with other opioid-induced disorder |
| F1199 |  | Opioid use, unspecified with unspecified opioid-induced disorder |
| F1210 |  | Cannabis abuse, uncomplicated |
| F1211 |  | Cannabis abuse, in remission |
| F12120 |  | Cannabis abuse with intoxication, uncomplicated |
| F12121 |  | Cannabis abuse with intoxication delirium |
| F12122 |  | Cannabis abuse with intoxication with perceptual disturbance |
| F12129 |  | Cannabis abuse with intoxication, unspecified |
| F12150 |  | Cannabis abuse with psychotic disorder with delusions |
| F12151 |  | Cannabis abuse with psychotic disorder with hallucinations |
| F12159 |  | Cannabis abuse with psychotic disorder, unspecified |
| F12180 |  | Cannabis abuse with cannabis-induced anxiety disorder |
| F12188 |  | Cannabis abuse with other cannabis-induced disorder |
| F1219 |  | Cannabis abuse with unspecified cannabis-induced disorder |
| F1220 |  | Cannabis dependence, uncomplicated |
| F1221 |  | Cannabis dependence, in remission |
| F12220 |  | Cannabis dependence with intoxication, uncomplicated |
| F12221 |  | Cannabis dependence with intoxication delirium |
| F12222 |  | Cannabis dependence with intoxication with perceptual disturbance |
| F12229 |  | Cannabis dependence with intoxication, unspecified |
| F1223 |  | Cannabis dependence with withdrawal |
| F12250 |  | Cannabis dependence with psychotic disorder with delusions |
| F12251 |  | Cannabis dependence with psychotic disorder with hallucinations |
| F12259 |  | Cannabis dependence with psychotic disorder, unspecified |
| F12280 |  | Cannabis dependence with cannabis-induced anxiety disorder |
| F12288 |  | Cannabis dependence with other cannabis-induced disorder |
| F1229 |  | Cannabis dependence with unspecified cannabis-induced disorder |
| F1290 |  | Cannabis use, unspecified, uncomplicated |
| F12920 |  | Cannabis use, unspecified with intoxication, uncomplicated |
| F12921 |  | Cannabis use, unspecified with intoxication delirium |
| F12922 |  | Cannabis use, unspecified with intoxication with perceptual disturbance |
| F12929 |  | Cannabis use, unspecified with intoxication, unspecified |
| F1293 |  | Cannabis use, unspecified with withdrawal |
| F12950 |  | Cannabis use, unspecified with psychotic disorder with delusions |
| F12951 |  | Cannabis use, unspecified with psychotic disorder with hallucinations |
| F12959 |  | Cannabis use, unspecified with psychotic disorder, unspecified |
| F12980 |  | Cannabis use, unspecified with anxiety disorder |
| F12988 |  | Cannabis use, unspecified with other cannabis-induced disorder |
| F1299 |  | Cannabis use, unspecified with unspecified cannabis-induced disorder |
| F1310 |  | Sedative, hypnotic or anxiolytic abuse, uncomplicated |
| F1311 |  | Sedative, hypnotic or anxiolytic abuse, in remission |
| F13120 |  | Sedative, hypnotic or anxiolytic abuse with intoxication, uncomplicated |
| F13121 |  | Sedative, hypnotic or anxiolytic abuse with intoxication delirium |
| F13129 |  | Sedative, hypnotic or anxiolytic abuse with intoxication, unspecified |
| F1314 |  | Sedative, hypnotic or anxiolytic abuse with sedative, hypnotic or anxiolytic-induced mood disorder |
| F13150 |  | Sedative, hypnotic or anxiolytic abuse with sedative, hypnotic or anxiolytic-induced psychotic disorder with delusions |
| F13151 |  | Sedative, hypnotic or anxiolytic abuse with sedative, hypnotic or anxiolytic-induced psychotic disorder with hallucinations |
| F13159 |  | Sedative, hypnotic or anxiolytic abuse with sedative, hypnotic or anxiolytic-induced psychotic disorder, unspecified |
| F13180 |  | Sedative, hypnotic or anxiolytic abuse with sedative, hypnotic or anxiolytic-induced anxiety disorder |
| F13181 |  | Sedative, hypnotic or anxiolytic abuse with sedative, hypnotic or anxiolytic-induced sexual dysfunction |
| F13182 |  | Sedative, hypnotic or anxiolytic abuse with sedative, hypnotic or anxiolytic-induced sleep disorder |
| F13188 |  | Sedative, hypnotic or anxiolytic abuse with other sedative, hypnotic or anxiolytic-induced disorder |
| F1319 |  | Sedative, hypnotic or anxiolytic abuse with unspecified sedative, hypnotic or anxiolytic-induced disorder |
| F1320 |  | Sedative, hypnotic or anxiolytic dependence, uncomplicated |
| F1321 |  | Sedative, hypnotic or anxiolytic dependence, in remission |
| F13220 |  | Sedative, hypnotic or anxiolytic dependence with intoxication, uncomplicated |
| F13221 |  | Sedative, hypnotic or anxiolytic dependence with intoxication delirium |
| F13229 |  | Sedative, hypnotic or anxiolytic dependence with intoxication, unspecified |
| F13230 |  | Sedative, hypnotic or anxiolytic dependence with withdrawal, uncomplicated |
| F13231 |  | Sedative, hypnotic or anxiolytic dependence with withdrawal delirium |
| F13232 |  | Sedative, hypnotic or anxiolytic dependence with withdrawal with perceptual disturbance |
| F13239 |  | Sedative, hypnotic or anxiolytic dependence with withdrawal, unspecified |
| F1324 |  | Sedative, hypnotic or anxiolytic dependence with sedative, hypnotic or anxiolytic-induced mood disorder |
| F13250 |  | Sedative, hypnotic or anxiolytic dependence with sedative, hypnotic or anxiolytic-induced psychotic disorder with delusions |
| F13251 |  | Sedative, hypnotic or anxiolytic dependence with sedative, hypnotic or anxiolytic-induced psychotic disorder with hallucinations |
| F13259 |  | Sedative, hypnotic or anxiolytic dependence with sedative, hypnotic or anxiolytic-induced psychotic disorder, unspecified |
| F1326 |  | Sedative, hypnotic or anxiolytic dependence with sedative, hypnotic or anxiolytic-induced persisting amnestic disorder |
| F1327 |  | Sedative, hypnotic or anxiolytic dependence with sedative, hypnotic or anxiolytic-induced persisting dementia |
| F13280 |  | Sedative, hypnotic or anxiolytic dependence with sedative, hypnotic or anxiolytic-induced anxiety disorder |
| F13281 |  | Sedative, hypnotic or anxiolytic dependence with sedative, hypnotic or anxiolytic-induced sexual dysfunction |
| F13282 |  | Sedative, hypnotic or anxiolytic dependence with sedative, hypnotic or anxiolytic-induced sleep disorder |
| F13288 |  | Sedative, hypnotic or anxiolytic dependence with other sedative, hypnotic or anxiolytic-induced disorder |
| F1329 |  | Sedative, hypnotic or anxiolytic dependence with unspecified sedative, hypnotic or anxiolytic-induced disorder |
| F1390 |  | Sedative, hypnotic, or anxiolytic use, unspecified, uncomplicated |
| F13920 |  | Sedative, hypnotic or anxiolytic use, unspecified with intoxication, uncomplicated |
| F13921 |  | Sedative, hypnotic or anxiolytic use, unspecified with intoxication delirium |
| F13929 |  | Sedative, hypnotic or anxiolytic use, unspecified with intoxication, unspecified |
| F13930 |  | Sedative, hypnotic or anxiolytic use, unspecified with withdrawal, uncomplicated |
| F13931 |  | Sedative, hypnotic or anxiolytic use, unspecified with withdrawal delirium |
| F13932 |  | Sedative, hypnotic or anxiolytic use, unspecified with withdrawal with perceptual disturbances |
| F15221 |  | Other stimulant dependence with intoxication delirium |
| F13939 |  | Sedative, hypnotic or anxiolytic use, unspecified with withdrawal, unspecified |
| F1394 |  | Sedative, hypnotic or anxiolytic use, unspecified with sedative, hypnotic or anxiolytic-induced mood disorder |
| F13950 |  | Sedative, hypnotic or anxiolytic use, unspecified with sedative, hypnotic or anxiolytic-induced psychotic disorder with delusions |
| F13951 |  | Sedative, hypnotic or anxiolytic use, unspecified with sedative, hypnotic or anxiolytic-induced psychotic disorder with hallucinations |
| F13959 |  | Sedative, hypnotic or anxiolytic use, unspecified with sedative, hypnotic or anxiolytic-induced psychotic disorder, unspecified |
| F1396 |  | Sedative, hypnotic or anxiolytic use, unspecified with sedative, hypnotic or anxiolytic-induced persisting amnestic disorder |
| F1397 |  | Sedative, hypnotic or anxiolytic use, unspecified with sedative, hypnotic or anxiolytic-induced persisting dementia |
| F13980 |  | Sedative, hypnotic or anxiolytic use, unspecified with sedative, hypnotic or anxiolytic-induced anxiety disorder |
| F13981 |  | Sedative, hypnotic or anxiolytic use, unspecified with sedative, hypnotic or anxiolytic-induced sexual dysfunction |
| F13982 |  | Sedative, hypnotic or anxiolytic use, unspecified with sedative, hypnotic or anxiolytic-induced sleep disorder |
| F13988 |  | Sedative, hypnotic or anxiolytic use, unspecified with other sedative, hypnotic or anxiolytic-induced disorder |
| F1399 |  | Sedative, hypnotic or anxiolytic use, unspecified with unspecified sedative, hypnotic or anxiolytic-induced disorder |
| F1410 |  | Cocaine abuse, uncomplicated |
| F1411 |  | Cocaine abuse, in remission |
| F14120 |  | Cocaine abuse with intoxication, uncomplicated |
| F14121 |  | Cocaine abuse with intoxication with delirium |
| F14122 |  | Cocaine abuse with intoxication with perceptual disturbance |
| F14129 |  | Cocaine abuse with intoxication, unspecified |
| F1414 |  | Cocaine abuse with cocaine-induced mood disorder |
| F14150 |  | Cocaine abuse with cocaine-induced psychotic disorder with delusions |
| F14151 |  | Cocaine abuse with cocaine-induced psychotic disorder with hallucinations |
| F14159 |  | Cocaine abuse with cocaine-induced psychotic disorder, unspecified |
| F14180 |  | Cocaine abuse with cocaine-induced anxiety disorder |
| F14181 |  | Cocaine abuse with cocaine-induced sexual dysfunction |
| F14182 |  | Cocaine abuse with cocaine-induced sleep disorder |
| F14188 |  | Cocaine abuse with other cocaine-induced disorder |
| F1419 |  | Cocaine abuse with unspecified cocaine-induced disorder |
| F1420 |  | Cocaine dependence, uncomplicated |
| F1421 |  | Cocaine dependence, in remission |
| F14220 |  | Cocaine dependence with intoxication, uncomplicated |
| F14221 |  | Cocaine dependence with intoxication delirium |
| F14222 |  | Cocaine dependence with intoxication with perceptual disturbance |
| F14229 |  | Cocaine dependence with intoxication, unspecified |
| F1423 |  | Cocaine dependence with withdrawal |
| F1424 |  | Cocaine dependence with cocaine-induced mood disorder |
| F14250 |  | Cocaine dependence with cocaine-induced psychotic disorder with delusions |
| F14251 |  | Cocaine dependence with cocaine-induced psychotic disorder with hallucinations |
| F14259 |  | Cocaine dependence with cocaine-induced psychotic disorder, unspecified |
| F14280 |  | Cocaine dependence with cocaine-induced anxiety disorder |
| F14281 |  | Cocaine dependence with cocaine-induced sexual dysfunction |
| F14282 |  | Cocaine dependence with cocaine-induced sleep disorder |
| F14288 |  | Cocaine dependence with other cocaine-induced disorder |
| F1429 |  | Cocaine dependence with unspecified cocaine-induced disorder |
| F1490 |  | Cocaine use, unspecified, uncomplicated |
| F14920 |  | Cocaine use, unspecified with intoxication, uncomplicated |
| F14921 |  | Cocaine use, unspecified with intoxication delirium |
| F14922 |  | Cocaine use, unspecified with intoxication with perceptual disturbance |
| F14929 |  | Cocaine use, unspecified with intoxication, unspecified |
| F1494 |  | Cocaine use, unspecified with cocaine-induced mood disorder |
| F14950 |  | Cocaine use, unspecified with cocaine-induced psychotic disorder with delusions |
| F14951 |  | Cocaine use, unspecified with cocaine-induced psychotic disorder with hallucinations |
| F14959 |  | Cocaine use, unspecified with cocaine-induced psychotic disorder, unspecified |
| F14980 |  | Cocaine use, unspecified with cocaine-induced anxiety disorder |
| F14981 |  | Cocaine use, unspecified with cocaine-induced sexual dysfunction |
| F14982 |  | Cocaine use, unspecified with cocaine-induced sleep disorder |
| F14988 |  | Cocaine use, unspecified with other cocaine-induced disorder |
| F1499 |  | Cocaine use, unspecified with unspecified cocaine-induced disorder |
| F1510 |  | Other stimulant abuse, uncomplicated |
| F1511 |  | Other stimulant abuse, in remission |
| F15120 |  | Other stimulant abuse with intoxication, uncomplicated |
| F15121 |  | Other stimulant abuse with intoxication delirium |
| F15122 |  | Other stimulant abuse with intoxication with perceptual disturbance |
| F15129 |  | Other stimulant abuse with intoxication, unspecified |
| F1514 |  | Other stimulant abuse with stimulant-induced mood disorder |
| F15150 |  | Other stimulant abuse with stimulant-induced psychotic disorder with delusions |
| F15151 |  | Other stimulant abuse with stimulant-induced psychotic disorder with hallucinations |
| F15159 |  | Other stimulant abuse with stimulant-induced psychotic disorder, unspecified |
| F15180 |  | Other stimulant abuse with stimulant-induced anxiety disorder |
| F15181 |  | Other stimulant abuse with stimulant-induced sexual dysfunction |
| F15182 |  | Other stimulant abuse with stimulant-induced sleep disorder |
| F15188 |  | Other stimulant abuse with other stimulant-induced disorder |
| F1519 |  | Other stimulant abuse with unspecified stimulant-induced disorder |
| F1520 |  | Other stimulant dependence, uncomplicated |
| F1521 |  | Other stimulant dependence, in remission |
| F15220 |  | Other stimulant dependence with intoxication, uncomplicated |
| F15222 |  | Other stimulant dependence with intoxication with perceptual disturbance |
| F15229 |  | Other stimulant dependence with intoxication, unspecified |
| F1523 |  | Other stimulant dependence with withdrawal |
| F1524 |  | Other stimulant dependence with stimulant-induced mood disorder |
| F15250 |  | Other stimulant dependence with stimulant-induced psychotic disorder with delusions |
| F15251 |  | Other stimulant dependence with stimulant-induced psychotic disorder with hallucinations |
| F15259 |  | Other stimulant dependence with stimulant-induced psychotic disorder, unspecified |
| F15280 |  | Other stimulant dependence with stimulant-induced anxiety disorder |
| F15281 |  | Other stimulant dependence with stimulant-induced sexual dysfunction |
| F15282 |  | Other stimulant dependence with stimulant-induced sleep disorder |
| F15288 |  | Other stimulant dependence with other stimulant-induced disorder |
| F1529 |  | Other stimulant dependence with unspecified stimulant-induced disorder |
| F1590 |  | Other stimulant use, unspecified, uncomplicated |
| F15920 |  | Other stimulant use, unspecified with intoxication, uncomplicated |
| F15921 |  | Other stimulant use, unspecified with intoxication delirium |
| F15922 |  | Other stimulant use, unspecified with intoxication with perceptual disturbance |
| F15929 |  | Other stimulant use, unspecified with intoxication, unspecified |
| F1593 |  | Other stimulant use, unspecified with withdrawal |
| F1594 |  | Other stimulant use, unspecified with stimulant-induced mood disorder |
| F15950 |  | Other stimulant use, unspecified with stimulant-induced psychotic disorder with delusions |
| F15951 |  | Other stimulant use, unspecified with stimulant-induced psychotic disorder with hallucinations |
| F15959 |  | Other stimulant use, unspecified with stimulant-induced psychotic disorder, unspecified |
| F15980 |  | Other stimulant use, unspecified with stimulant-induced anxiety disorder |
| F15981 |  | Other stimulant use, unspecified with stimulant-induced sexual dysfunction |
| F15982 |  | Other stimulant use, unspecified with stimulant-induced sleep disorder |
| F15988 |  | Other stimulant use, unspecified with other stimulant-induced disorder |
| F1599 |  | Other stimulant use, unspecified with unspecified stimulant-induced disorder |
| F1511 |  | Other stimulant abuse, in remission |
| F15120 |  | Other stimulant abuse with intoxication, uncomplicated |
| F15121 |  | Other stimulant abuse with intoxication delirium |
| F15122 |  | Other stimulant abuse with intoxication with perceptual disturbance |
| F15129 |  | Other stimulant abuse with intoxication, unspecified |
| F1514 |  | Other stimulant abuse with stimulant-induced mood disorder |
| F15150 |  | Other stimulant abuse with stimulant-induced psychotic disorder with delusions |
| F15151 |  | Other stimulant abuse with stimulant-induced psychotic disorder with hallucinations |
| F15159 |  | Other stimulant abuse with stimulant-induced psychotic disorder, unspecified |
| F15180 |  | Other stimulant abuse with stimulant-induced anxiety disorder |
| F15181 |  | Other stimulant abuse with stimulant-induced sexual dysfunction |
| F15182 |  | Other stimulant abuse with stimulant-induced sleep disorder |
| F15188 |  | Other stimulant abuse with other stimulant-induced disorder |
| F1519 |  | Other stimulant abuse with unspecified stimulant-induced disorder |
| F1520 |  | Other stimulant dependence, uncomplicated |
| F1521 |  | Other stimulant dependence, in remission |
| F15220 |  | Other stimulant dependence with intoxication, uncomplicated |
| F15222 |  | Other stimulant dependence with intoxication with perceptual disturbance |
| F15229 |  | Other stimulant dependence with intoxication, unspecified |
| F1523 |  | Other stimulant dependence with withdrawal |
| F1524 |  | Other stimulant dependence with stimulant-induced mood disorder |
| F15250 |  | Other stimulant dependence with stimulant-induced psychotic disorder with delusions |
| F15251 |  | Other stimulant dependence with stimulant-induced psychotic disorder with hallucinations |
| F15259 |  | Other stimulant dependence with stimulant-induced psychotic disorder, unspecified |
| F15280 |  | Other stimulant dependence with stimulant-induced anxiety disorder |
| F15281 |  | Other stimulant dependence with stimulant-induced sexual dysfunction |
| F15282 |  | Other stimulant dependence with stimulant-induced sleep disorder |
| F15288 |  | Other stimulant dependence with other stimulant-induced disorder |
| F1529 |  | Other stimulant dependence with unspecified stimulant-induced disorder |
| F1590 |  | Other stimulant use, unspecified, uncomplicated |
| F15920 |  | Other stimulant use, unspecified with intoxication, uncomplicated |
| F15921 |  | Other stimulant use, unspecified with intoxication delirium |
| F15922 |  | Other stimulant use, unspecified with intoxication with perceptual disturbance |
| F15929 |  | Other stimulant use, unspecified with intoxication, unspecified |
| F1593 |  | Other stimulant use, unspecified with withdrawal |
| F1594 |  | Other stimulant use, unspecified with stimulant-induced mood disorder |
| F15950 |  | Other stimulant use, unspecified with stimulant-induced psychotic disorder with delusions |
| F15951 |  | Other stimulant use, unspecified with stimulant-induced psychotic disorder with hallucinations |
| F15959 |  | Other stimulant use, unspecified with stimulant-induced psychotic disorder, unspecified |
| F15980 |  | Other stimulant use, unspecified with stimulant-induced anxiety disorder |
| F15981 |  | Other stimulant use, unspecified with stimulant-induced sexual dysfunction |
| F15982 |  | Other stimulant use, unspecified with stimulant-induced sleep disorder |
| F15988 |  | Other stimulant use, unspecified with other stimulant-induced disorder |
| F1599 |  | Other stimulant use, unspecified with unspecified stimulant-induced disorder |
| F1610 |  | Hallucinogen abuse, uncomplicated |
| F1611 |  | Hallucinogen abuse, in remission |
| F16120 |  | Hallucinogen abuse with intoxication, uncomplicated |
| F16121 |  | Hallucinogen abuse with intoxication with delirium |
| F16122 |  | Hallucinogen abuse with intoxication with perceptual disturbance |
| F16129 |  | Hallucinogen abuse with intoxication, unspecified |
| F1614 |  | Hallucinogen abuse with hallucinogen-induced mood disorder |
| F16150 |  | Hallucinogen abuse with hallucinogen-induced psychotic disorder with delusions |
| F16151 |  | Hallucinogen abuse with hallucinogen-induced psychotic disorder with hallucinations |
| F16159 |  | Hallucinogen abuse with hallucinogen-induced psychotic disorder, unspecified |
| F16180 |  | Hallucinogen abuse with hallucinogen-induced anxiety disorder |
| F16183 |  | Hallucinogen abuse with hallucinogen persisting perception disorder (flashbacks) |
| F16188 |  | Hallucinogen abuse with other hallucinogen-induced disorder |
| F1619 |  | Hallucinogen abuse with unspecified hallucinogen-induced disorder |
| F1620 |  | Hallucinogen dependence, uncomplicated |
| F1621 |  | Hallucinogen dependence, in remission |
| F16220 |  | Hallucinogen dependence with intoxication, uncomplicated |
| F16221 |  | Hallucinogen dependence with intoxication with delirium |
| F16229 |  | Hallucinogen dependence with intoxication, unspecified |
| F1624 |  | Hallucinogen dependence with hallucinogen-induced mood disorder |
| F16250 |  | Hallucinogen dependence with hallucinogen-induced psychotic disorder with delusions |
| F16251 |  | Hallucinogen dependence with hallucinogen-induced psychotic disorder with hallucinations |
| F16259 |  | Hallucinogen dependence with hallucinogen-induced psychotic disorder, unspecified |
| F16280 |  | Hallucinogen dependence with hallucinogen-induced anxiety disorder |
| F16283 |  | Hallucinogen dependence with hallucinogen persisting perception disorder (flashbacks) |
| F16288 |  | Hallucinogen dependence with other hallucinogen-induced disorder |
| F1629 |  | Hallucinogen dependence with unspecified hallucinogen-induced disorder |
| F1690 |  | Hallucinogen use, unspecified, uncomplicated |
| F16920 |  | Hallucinogen use, unspecified with intoxication, uncomplicated |
| F16921 |  | Hallucinogen use, unspecified with intoxication with delirium |
| F16929 |  | Hallucinogen use, unspecified with intoxication, unspecified |
| F1694 |  | Hallucinogen use, unspecified with hallucinogen-induced mood disorder |
| F16950 |  | Hallucinogen use, unspecified with hallucinogen-induced psychotic disorder with delusions |
| F16951 |  | Hallucinogen use, unspecified with hallucinogen-induced psychotic disorder with hallucinations |
| F16959 |  | Hallucinogen use, unspecified with hallucinogen-induced psychotic disorder, unspecified |
| F16980 |  | Hallucinogen use, unspecified with hallucinogen-induced anxiety disorder |
| F16983 |  | Hallucinogen use, unspecified with hallucinogen persisting perception disorder (flashbacks) |
| F16988 |  | Hallucinogen use, unspecified with other hallucinogen-induced disorder |
| F1699 |  | Hallucinogen use, unspecified with unspecified hallucinogen-induced disorder |
| F17200 |  | Nicotine dependence, unspecified, uncomplicated |
| F17201 |  | Nicotine dependence, unspecified, in remission |
| F17203 |  | Nicotine dependence unspecified, with withdrawal |
| F17208 |  | Nicotine dependence, unspecified, with other nicotine-induced disorders |
| F17209 |  | Nicotine dependence, unspecified, with unspecified nicotine-induced disorders |
| F17210 |  | Nicotine dependence, cigarettes, uncomplicated |
| F17211 |  | Nicotine dependence, cigarettes, in remission |
| F17213 |  | Nicotine dependence, cigarettes, with withdrawal |
| F17218 |  | Nicotine dependence, cigarettes, with other nicotine-induced disorders |
| F17219 |  | Nicotine dependence, cigarettes, with unspecified nicotine-induced disorders |
| F17220 |  | Nicotine dependence, chewing tobacco, uncomplicated |
| F17221 |  | Nicotine dependence, chewing tobacco, in remission |
| F17223 |  | Nicotine dependence, chewing tobacco, with withdrawal |
| F17228 |  | Nicotine dependence, chewing tobacco, with other nicotine-induced disorders |
| F17229 |  | Nicotine dependence, chewing tobacco, with unspecified nicotine-induced disorders |
| F17290 |  | Nicotine dependence, other tobacco product, uncomplicated |
| F17291 |  | Nicotine dependence, other tobacco product, in remission |
| F17293 |  | Nicotine dependence, other tobacco product, with withdrawal |
| F17298 |  | Nicotine dependence, other tobacco product, with other nicotine-induced disorders |
| F17299 |  | Nicotine dependence, other tobacco product, with unspecified nicotine-induced disorders |
| F1910 |  | Other psychoactive substance abuse, uncomplicated |
| F1911 |  | Other psychoactive substance abuse, in remission |
| F19120 |  | Other psychoactive substance abuse with intoxication, uncomplicated |
| F19121 |  | Other psychoactive substance abuse with intoxication delirium |
| F19122 |  | Other psychoactive substance abuse with intoxication with perceptual disturbances |
| F19129 |  | Other psychoactive substance abuse with intoxication, unspecified |
| F1914 |  | Other psychoactive substance abuse with psychoactive substance-induced mood disorder |
| F19150 |  | Other psychoactive substance abuse with psychoactive substance-induced psychotic disorder with delusions |
| F19151 |  | Other psychoactive substance abuse with psychoactive substance-induced psychotic disorder with hallucinations |
| F19159 |  | Other psychoactive substance abuse with psychoactive substance-induced psychotic disorder, unspecified |
| F1916 |  | Other psychoactive substance abuse with psychoactive substance-induced persisting amnestic disorder |
| F1917 |  | Other psychoactive substance abuse with psychoactive substance-induced persisting dementia |
| F19180 |  | Other psychoactive substance abuse with psychoactive substance-induced anxiety disorder |
| F19181 |  | Other psychoactive substance abuse with psychoactive substance-induced sexual dysfunction |
| F19182 |  | Other psychoactive substance abuse with psychoactive substance-induced sleep disorder |
| F19188 |  | Other psychoactive substance abuse with other psychoactive substance-induced disorder |
| F1919 |  | Other psychoactive substance abuse with unspecified psychoactive substance-induced disorder |
| F1920 |  | Other psychoactive substance dependence, uncomplicated |
| F1921 |  | Other psychoactive substance dependence, in remission |
| F19220 |  | Other psychoactive substance dependence with intoxication, uncomplicated |
| F19221 |  | Other psychoactive substance dependence with intoxication delirium |
| F19222 |  | Other psychoactive substance dependence with intoxication with perceptual disturbance |
| F19229 |  | Other psychoactive substance dependence with intoxication, unspecified |
| F19230 |  | Other psychoactive substance dependence with withdrawal, uncomplicated |
| F19231 |  | Other psychoactive substance dependence with withdrawal delirium |
| F19232 |  | Other psychoactive substance dependence with withdrawal with perceptual disturbance |
| F458 |  | Other somatoform disorders |
| F19239 |  | Other psychoactive substance dependence with withdrawal, unspecified |
| F1924 |  | Other psychoactive substance dependence with psychoactive substance-induced mood disorder |
| F19250 |  | Other psychoactive substance dependence with psychoactive substance-induced psychotic disorder with delusions |
| F19251 |  | Other psychoactive substance dependence with psychoactive substance-induced psychotic disorder with hallucinations |
| F19259 |  | Other psychoactive substance dependence with psychoactive substance-induced psychotic disorder, unspecified |
| F1926 |  | Other psychoactive substance dependence with psychoactive substance-induced persisting amnestic disorder |
| F1927 |  | Other psychoactive substance dependence with psychoactive substance-induced persisting dementia |
| F19280 |  | Other psychoactive substance dependence with psychoactive substance-induced anxiety disorder |
| F19281 |  | Other psychoactive substance dependence with psychoactive substance-induced sexual dysfunction |
| F19282 |  | Other psychoactive substance dependence with psychoactive substance-induced sleep disorder |
| F19288 |  | Other psychoactive substance dependence with other psychoactive substance-induced disorder |
| F1929 |  | Other psychoactive substance dependence with unspecified psychoactive substance-induced disorder |
| F1990 |  | Other psychoactive substance use, unspecified, uncomplicated |
| F19920 |  | Other psychoactive substance use, unspecified with intoxication, uncomplicated |
| F19921 |  | Other psychoactive substance use, unspecified with intoxication with delirium |
| F19922 |  | Other psychoactive substance use, unspecified with intoxication with perceptual disturbance |
| F19929 |  | Other psychoactive substance use, unspecified with intoxication, unspecified |
| F19930 |  | Other psychoactive substance use, unspecified with withdrawal, uncomplicated |
| F19931 |  | Other psychoactive substance use, unspecified with withdrawal delirium |
| F19932 |  | Other psychoactive substance use, unspecified with withdrawal with perceptual disturbance |
| F19939 |  | Other psychoactive substance use, unspecified with withdrawal, unspecified |
| F1994 |  | Other psychoactive substance use, unspecified with psychoactive substance-induced mood disorder |
| F19950 |  | Other psychoactive substance use, unspecified with psychoactive substance-induced psychotic disorder with delusions |
| F19951 |  | Other psychoactive substance use, unspecified with psychoactive substance-induced psychotic disorder with hallucinations |
| F19959 |  | Other psychoactive substance use, unspecified with psychoactive substance-induced psychotic disorder, unspecified |
| F1996 |  | Other psychoactive substance use, unspecified with psychoactive substance-induced persisting amnestic disorder |
| F1997 |  | Other psychoactive substance use, unspecified with psychoactive substance-induced persisting dementia |
| F19980 |  | Other psychoactive substance use, unspecified with psychoactive substance-induced anxiety disorder |
| F19981 |  | Other psychoactive substance use, unspecified with psychoactive substance-induced sexual dysfunction |
| F19982 |  | Other psychoactive substance use, unspecified with psychoactive substance-induced sleep disorder |
| F19988 |  | Other psychoactive substance use, unspecified with other psychoactive substance-induced disorder |
| F1999 |  | Other psychoactive substance use, unspecified with unspecified psychoactive substance-induced disorder |
|  |  |  |
