# Supplementary figures and images for: Development and validation of prediction models for neurocognitive disorders in adult patients admitted to the ICU with sleep disturbance
Source: CNS Neurosci Ther. 2021 Dec 23;28(4):554–65. doi: 10.1111/cns.13772 (PMC8928914; doi:10.1111/cns.13772)

Appendix S4
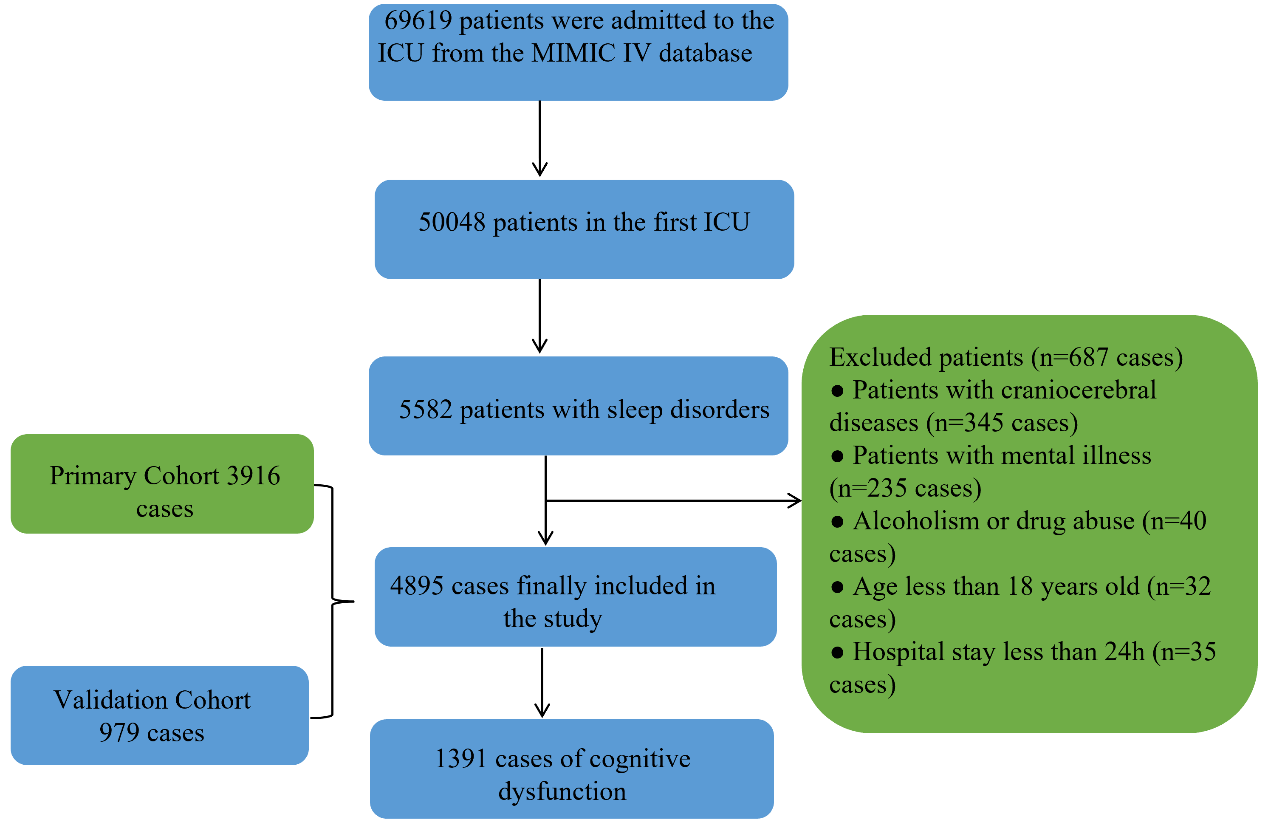

Supplement: Supplementary file 4 — App S4 [file CNS-28-554-s001.docx]

Appendix S5
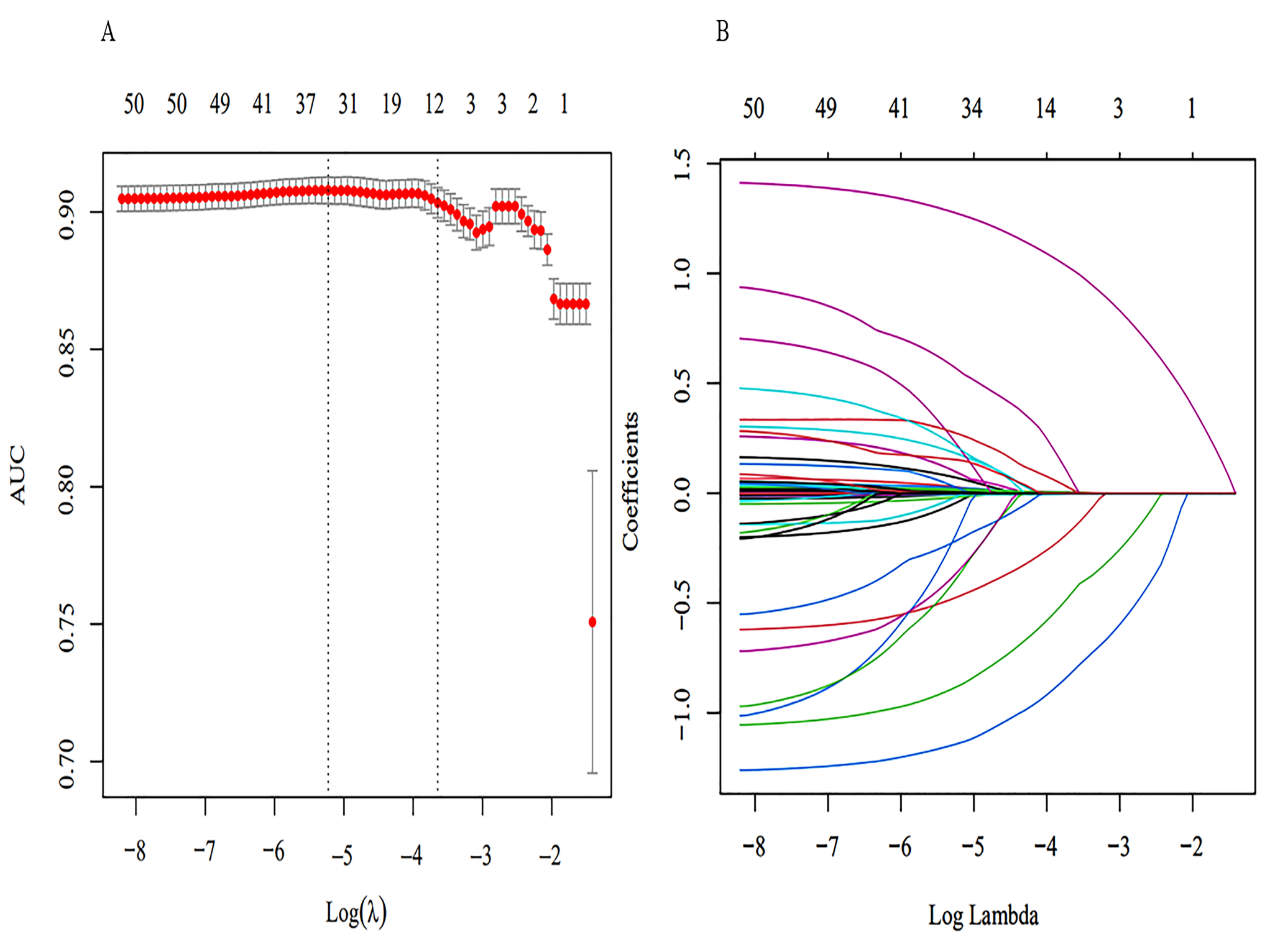

Supplement: Supplementary file 5 — App S5 [file CNS-28-554-s003.docx]

Appendix S7
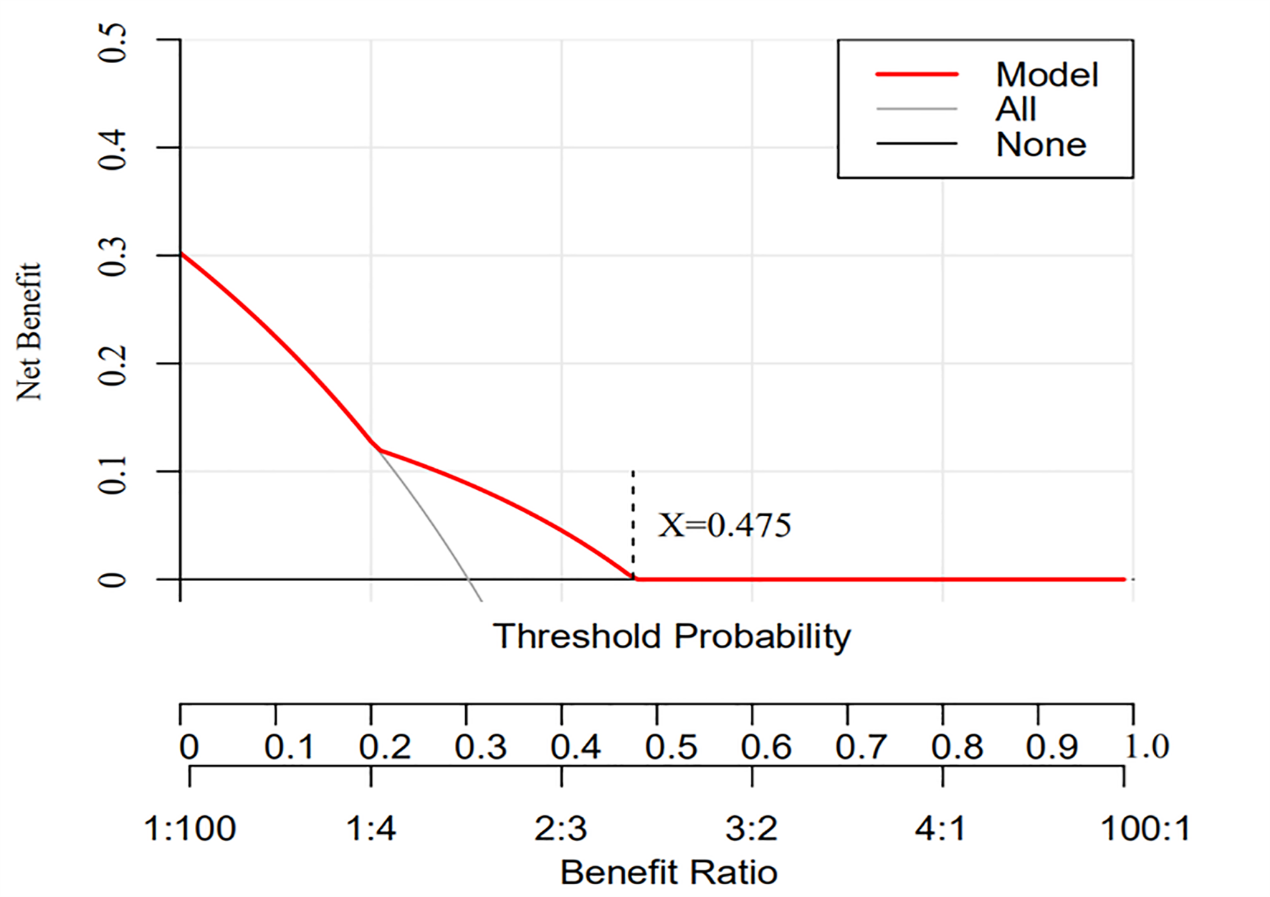

Supplement: Supplementary file 7 — App S7 [file CNS-28-554-s007.docx]
