# Supplementary material for: Development and validation of prediction models for neurocognitive disorders in adult patients admitted to the ICU with sleep disturbance
Source: CNS Neurosci Ther. 2021 Dec 23;28(4):554–65. doi: 10.1111/cns.13772 (PMC8928914; doi:10.1111/cns.13772)
Supplement: Supplementary file 6 — App S6 [file CNS-28-554-s005.docx]

| **Factors** | **Coefficients** | **Lambda.S1se** |
| --- | --- | --- |
| Gender | -0.16177 | 0.026 |
| Platelets_min(×10^9^) | -0.00012 |  |
| Glucose_max(mg/dl) | 0.00012 |  |
| Potassium_max(mmol/L) | 0.0525 |  |
| International standard ratio_max | 0.00322 |  |
| Partial prothrombin time_max(s) | 0.00417 |  |
| Respiratory rate_max(bpm) | 0.00221 |  |
| Age(year) | 0.00296 |  |
| Diabetes | 0.01602 |  |
| Glasgow | 1.01589 |  |
| Morphine | 0.45012 |  |
| Midazolam | 0.81195 |  |

**Appendix S6** Coefficients and Lambda.S1se Value of the LASSO Regression
