# Supplementary material for: Development and validation of prediction models for neurocognitive disorders in adult patients admitted to the ICU with sleep disturbance
Source: CNS Neurosci Ther. 2021 Dec 23;28(4):554–65. doi: 10.1111/cns.13772 (PMC8928914; doi:10.1111/cns.13772)
Supplement: Supplementary file 8 — Data S1 [file CNS-28-554-s008.pdf]

Data Profiling Report

- Basic Statistics
  - Raw Counts
  - Percentages
- Data Structure
- Missing Data Profile
- Univariate Distribution
  - Histogram
  - Bar Chart (with frequency)
  - QQ Plot
- Correlation Analysis
- Principal Component Analysis

Basic Statistics

Raw Counts

**Name**	**Value**
Rows	4,895
Columns	32
Discrete columns	1
Continuous columns	31
All missing columns	0
Missing observations	1,129
Complete Rows	4,747
Total observations	156,640
Memory allocation	982.2 Kb

Percentages

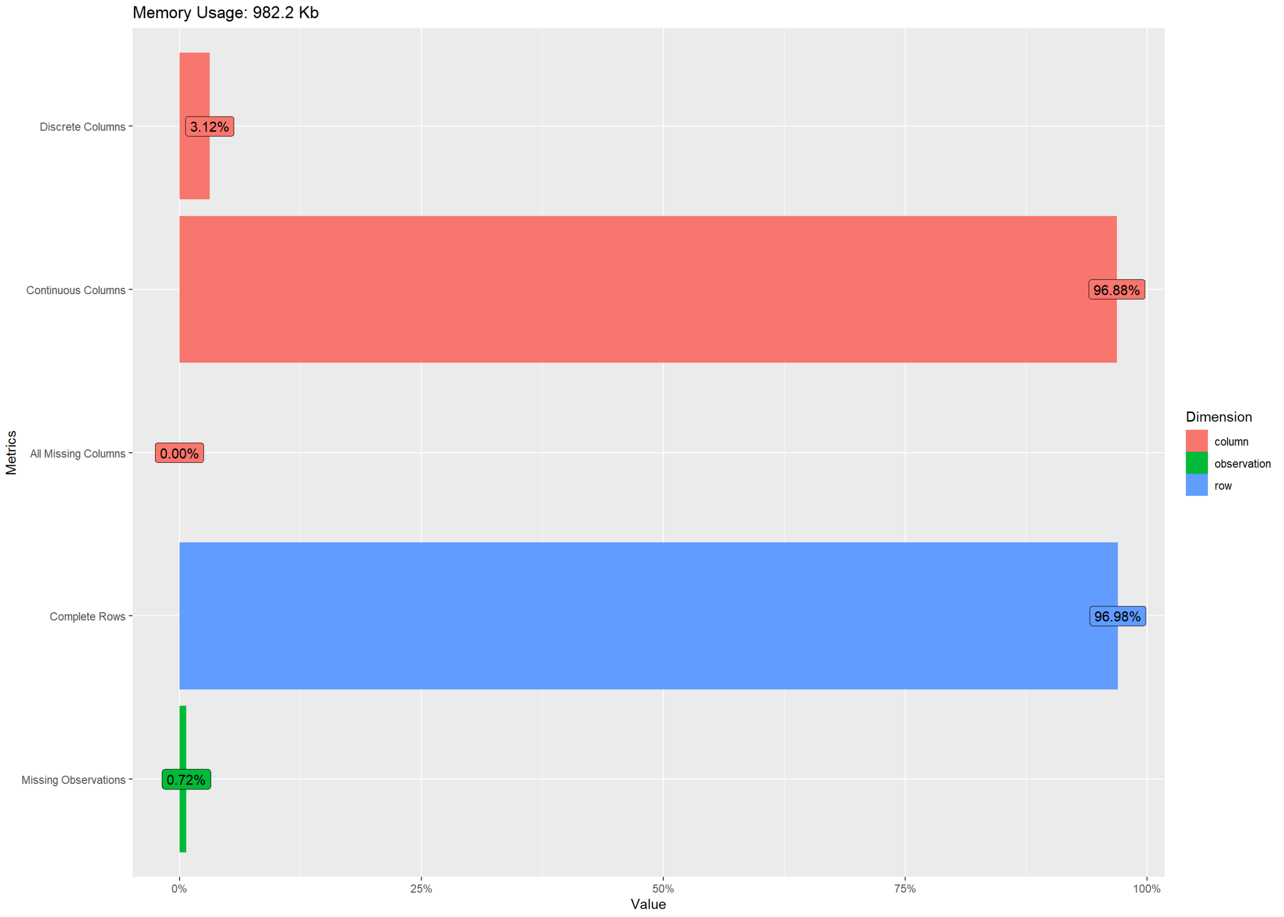

Data Structure

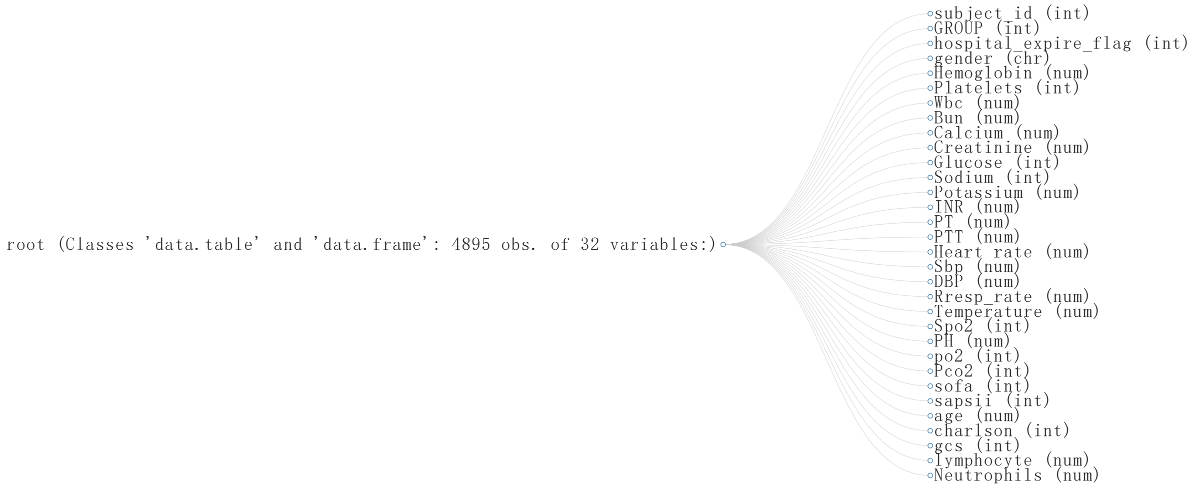

Missing Data Profile

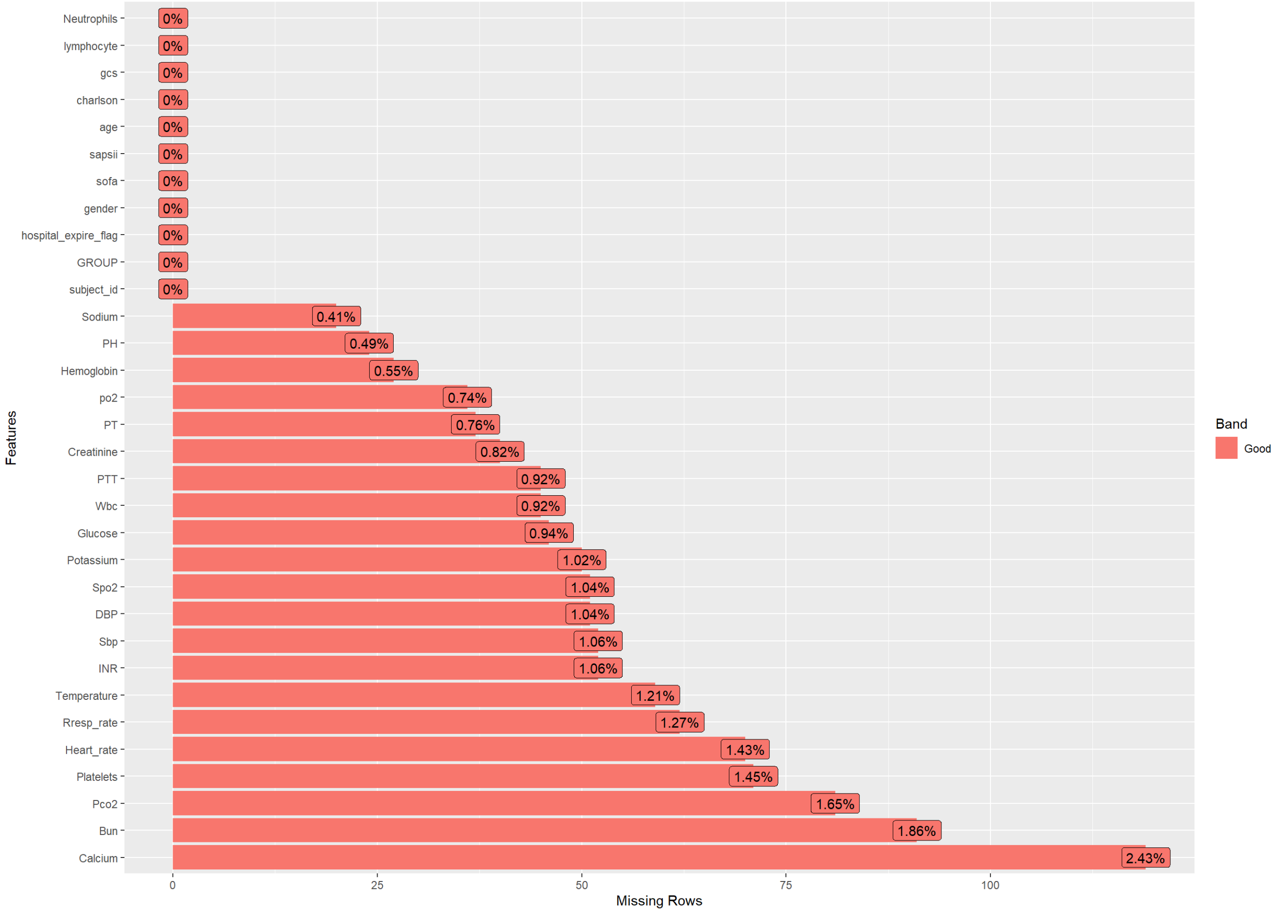

Univariate Distribution

Histogram

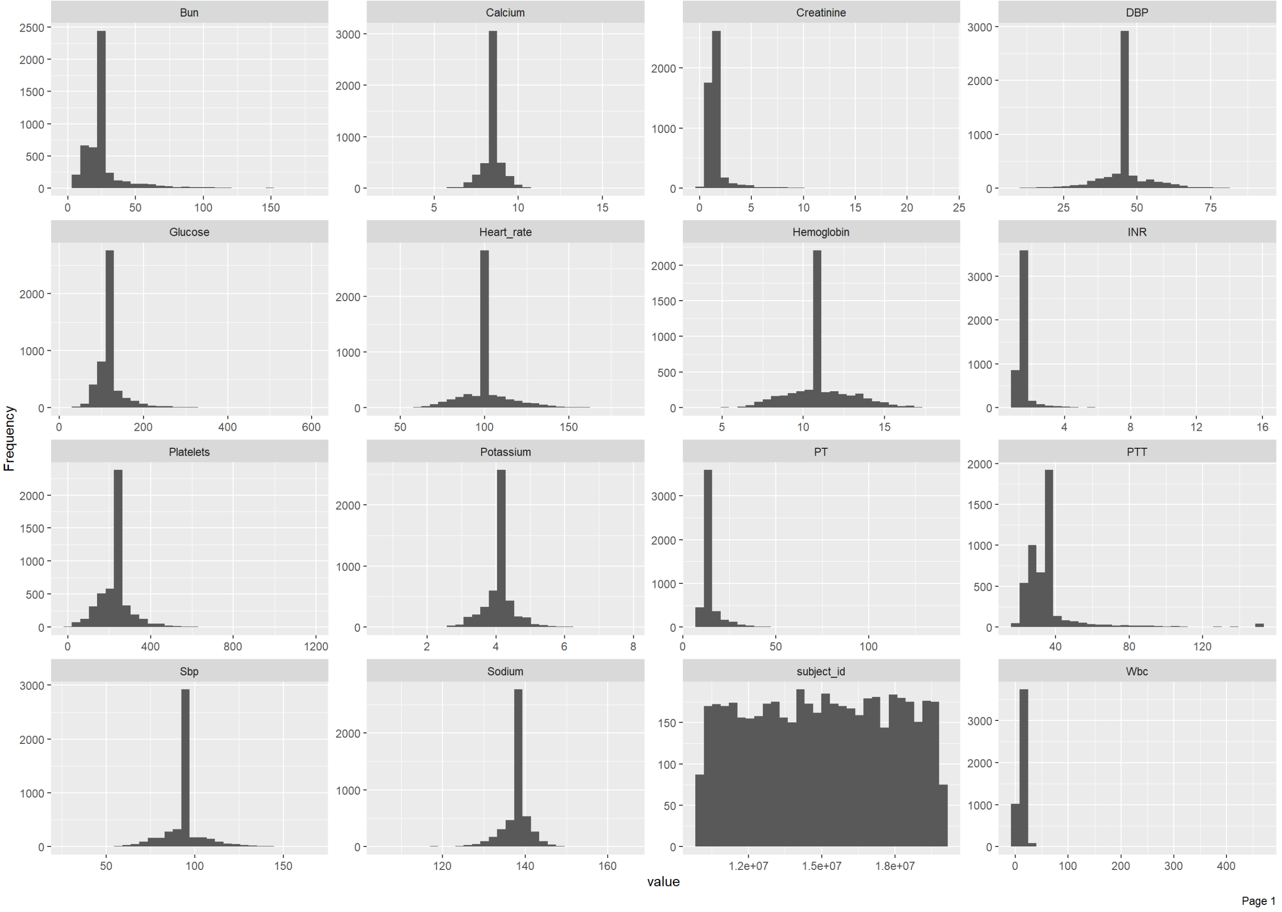

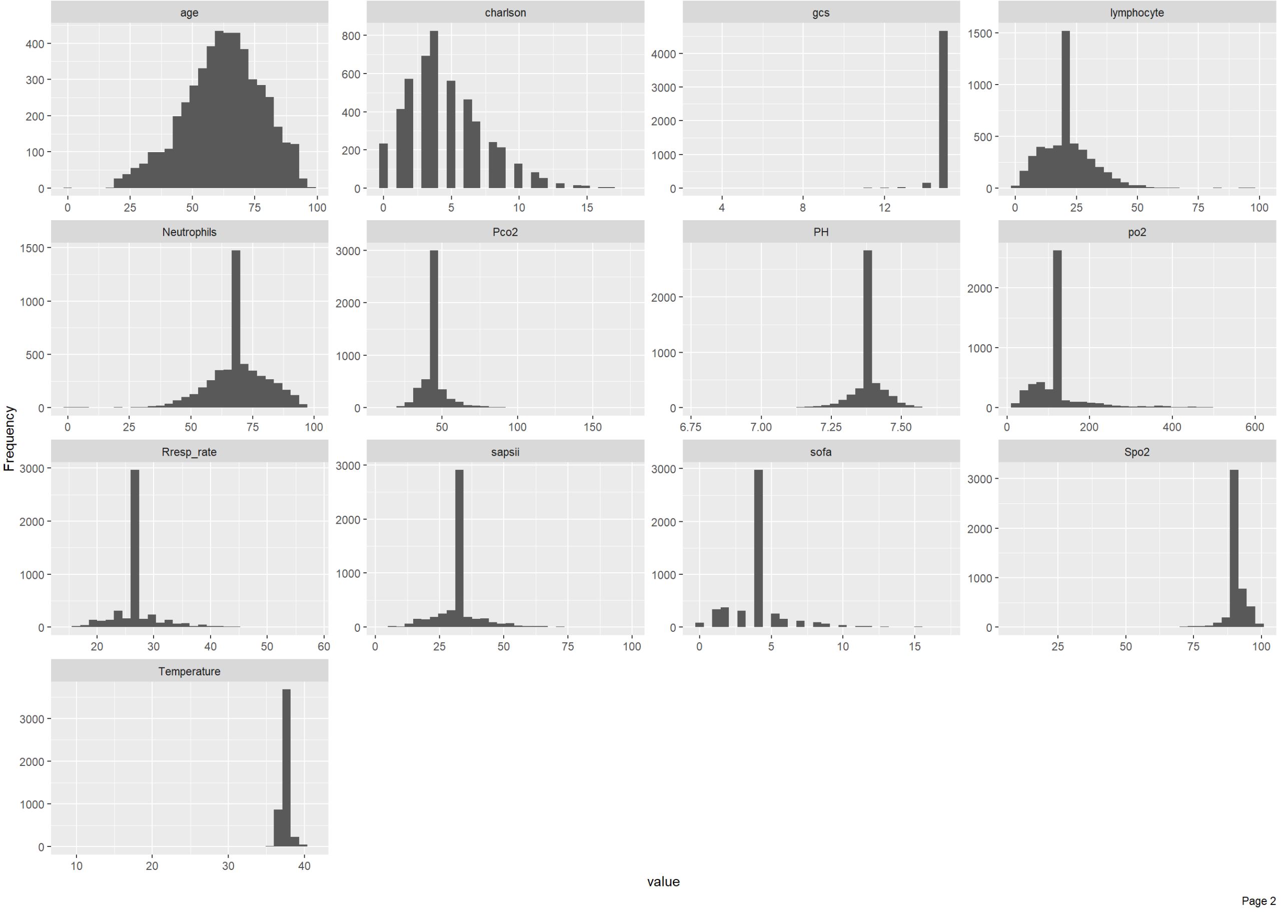

Bar Chart (with frequency)

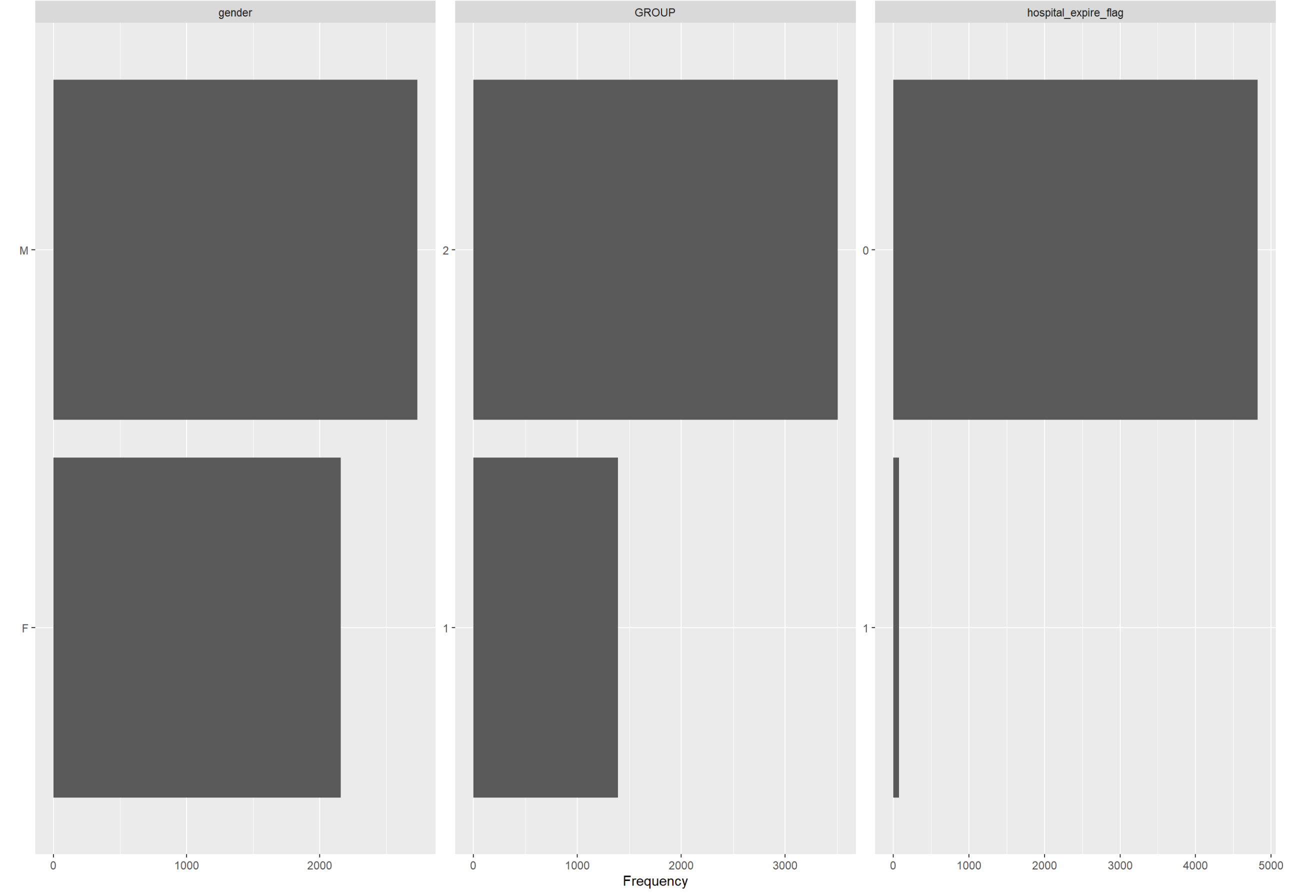

QQ Plot

```
## Warning: Removed 91 rows containing non-finite values (stat_qq).
```

```
## Warning: Removed 91 rows containing non-finite values (stat_qq_line).
```

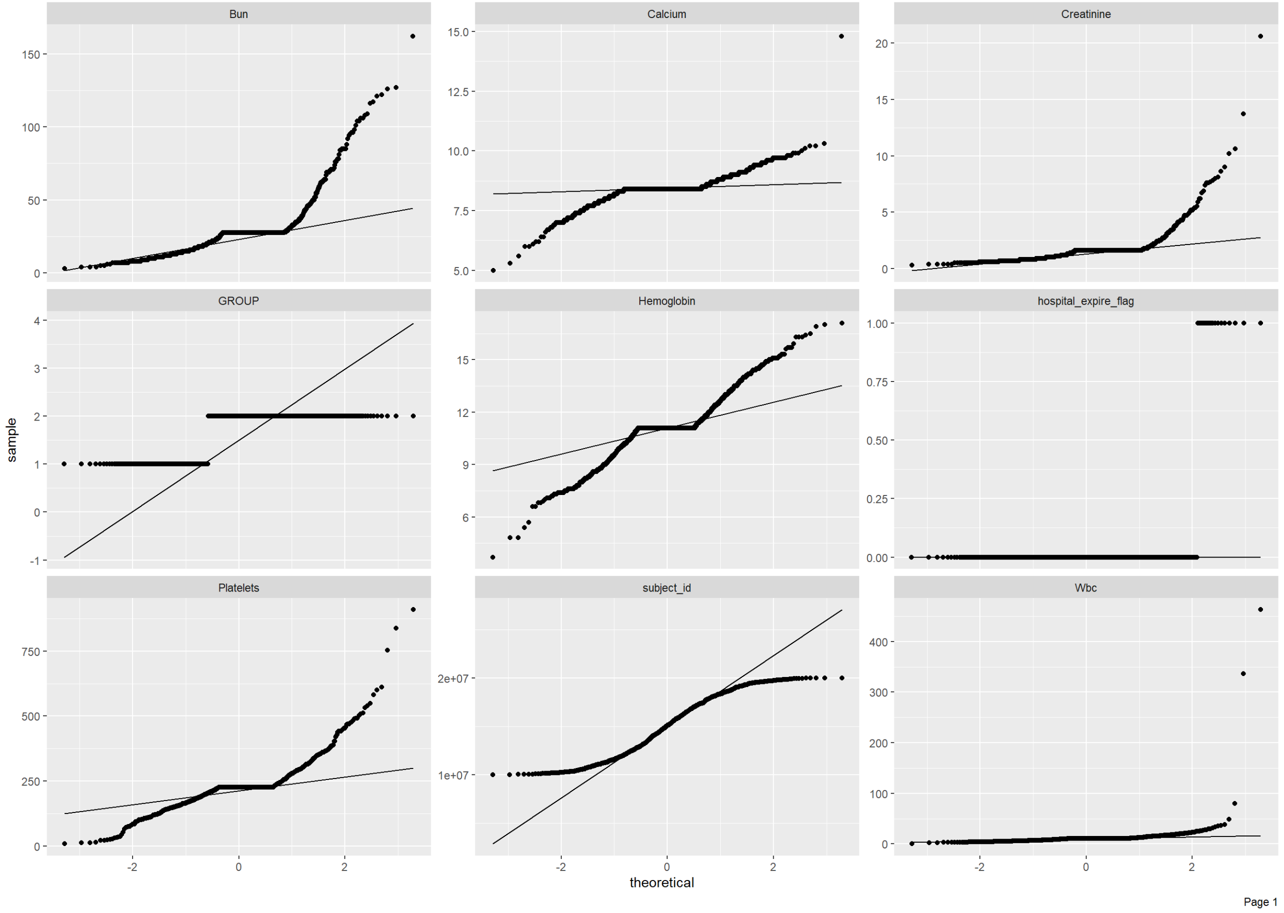

```
## Warning: Removed 95 rows containing non-finite values (stat_qq).
```

```
## Warning: Removed 95 rows containing non-finite values (stat_qq_line).
```

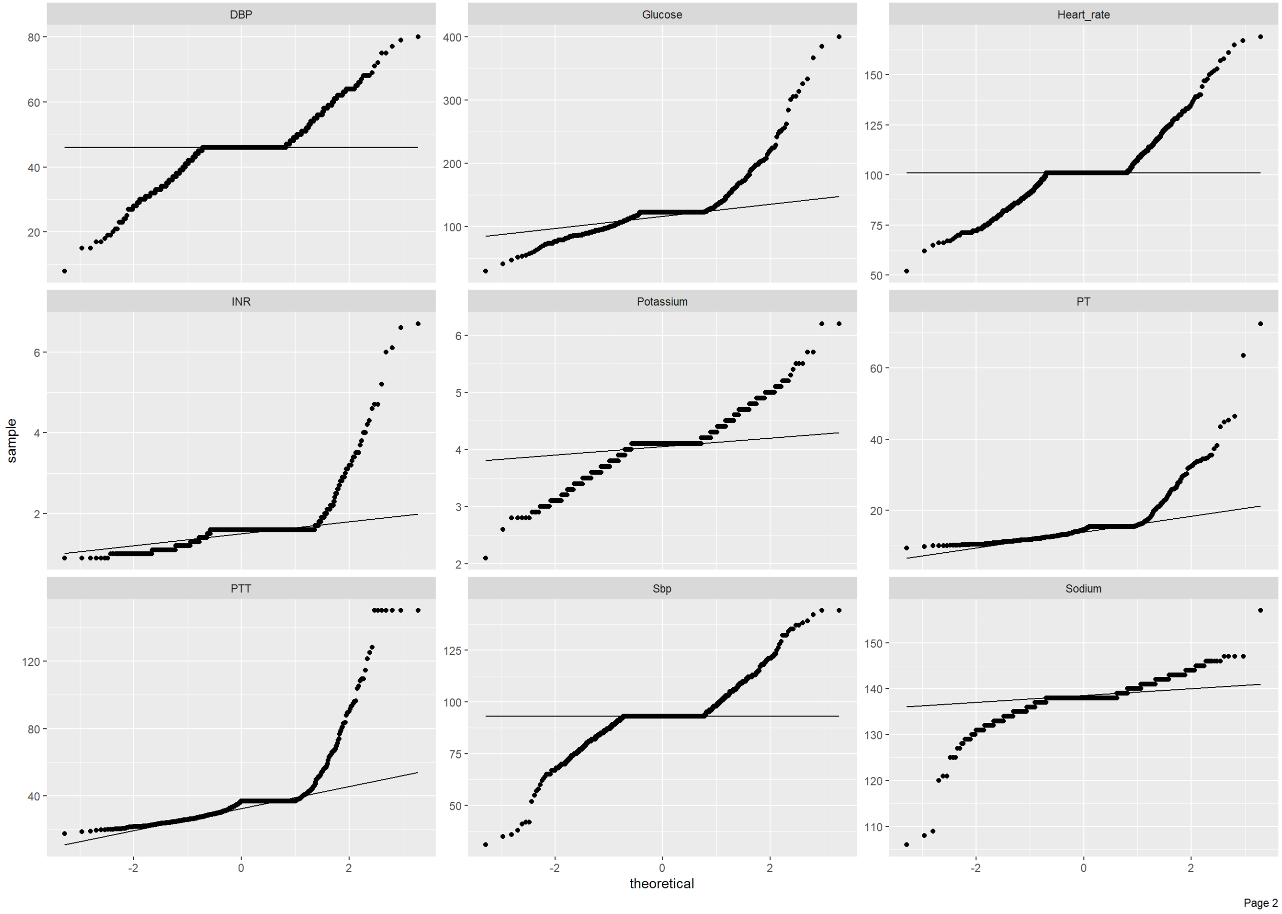


```
## Warning: Removed 73 rows containing non-finite values (stat_qq).
```

```
## Warning: Removed 73 rows containing non-finite values (stat_qq_line).
```

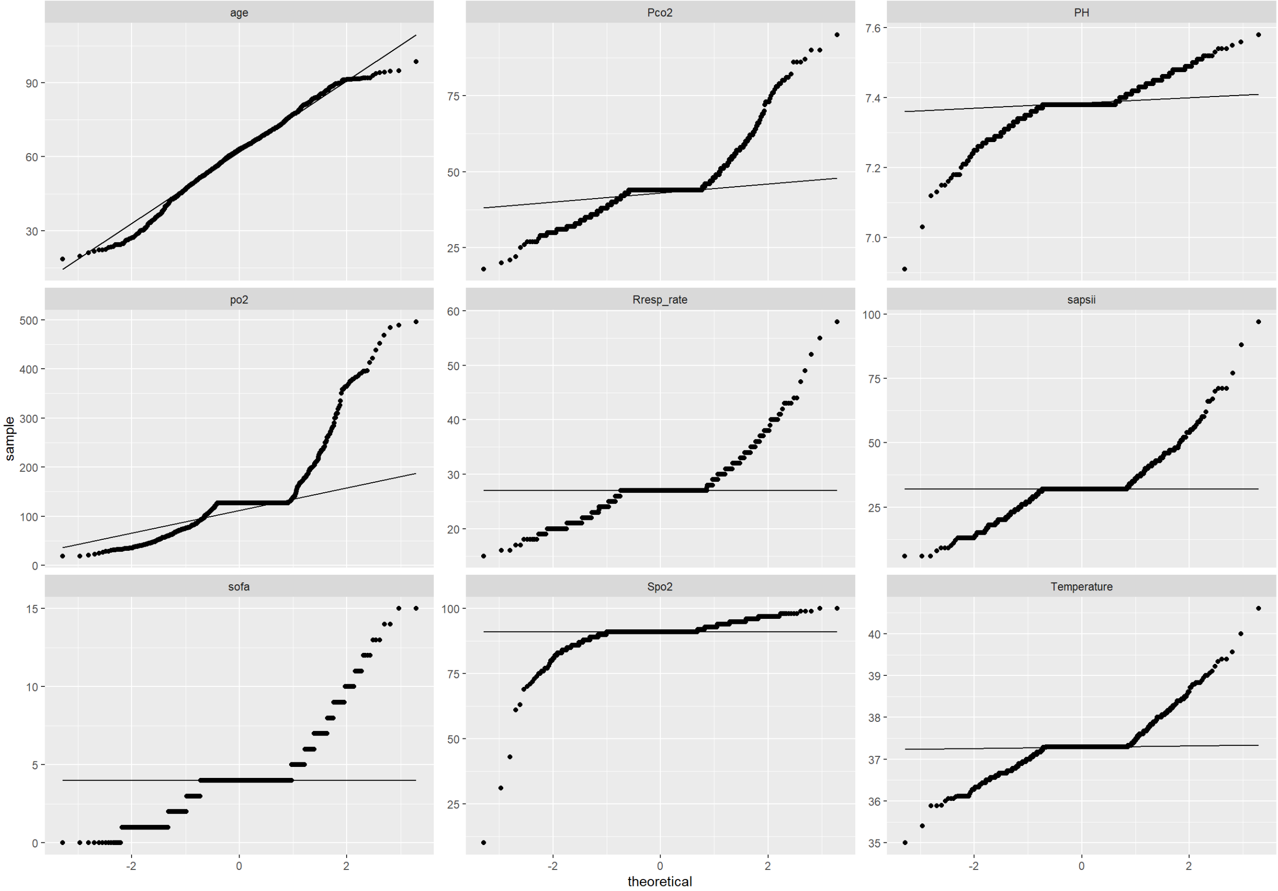

Page 3

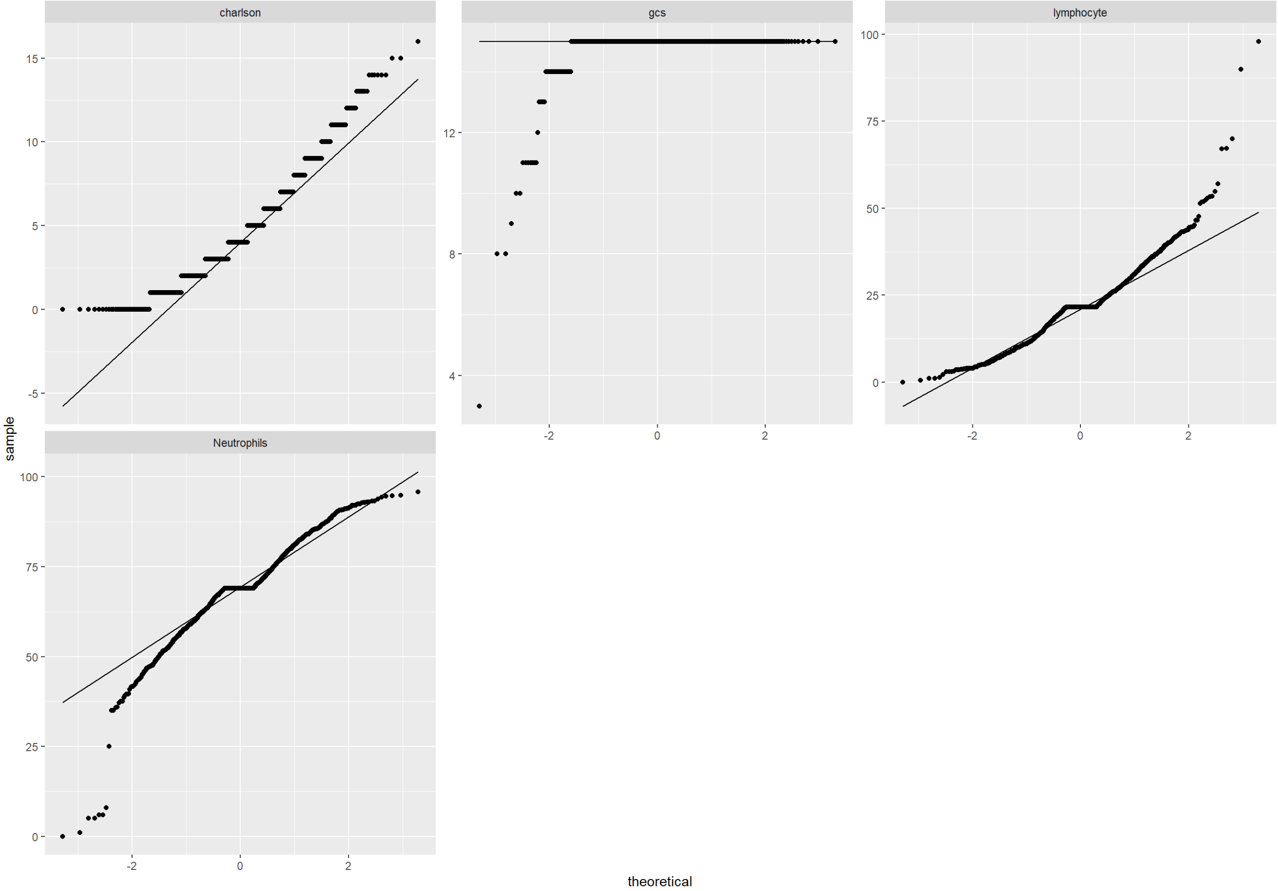

Page 4

Correlation Analysis

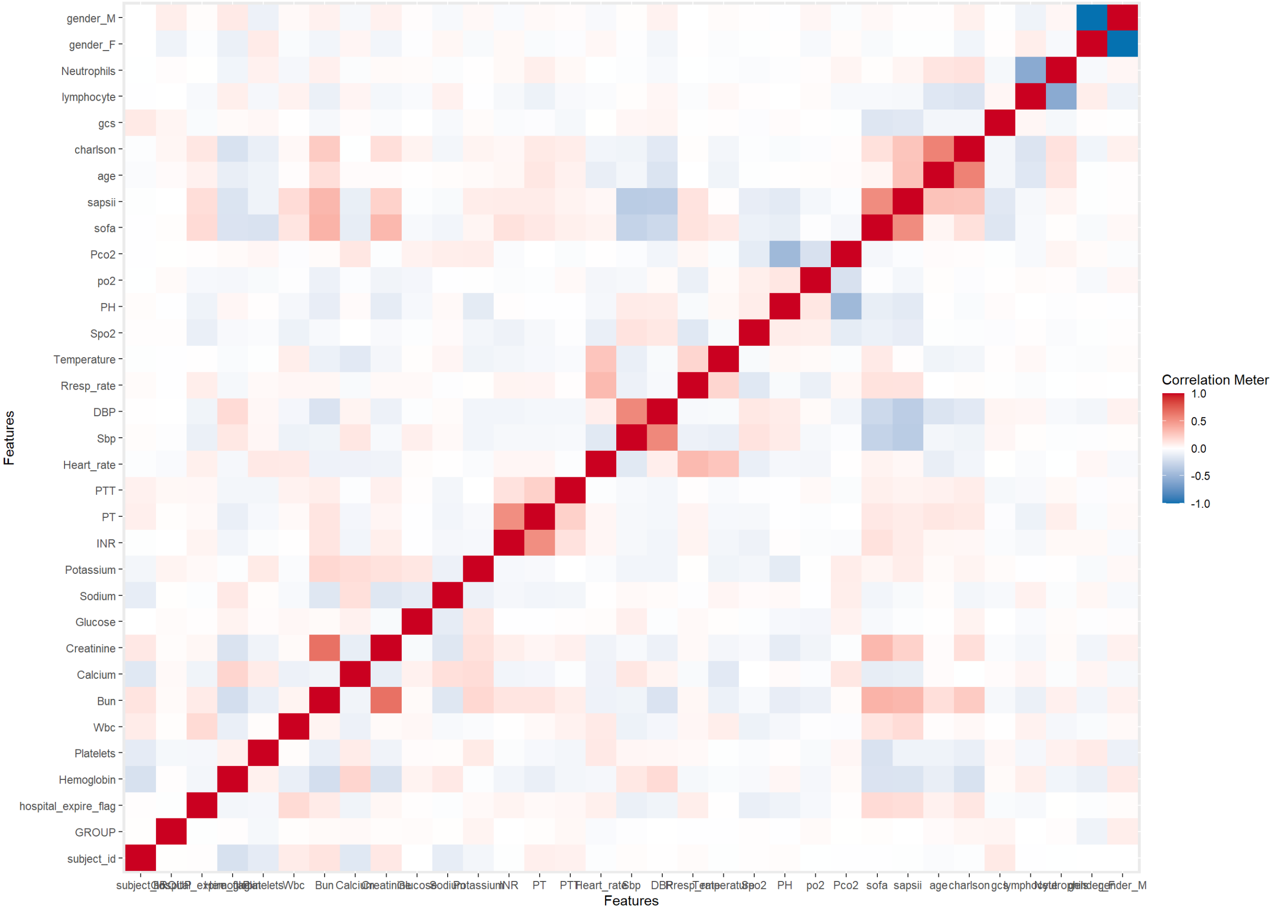

Principal Component Analysis

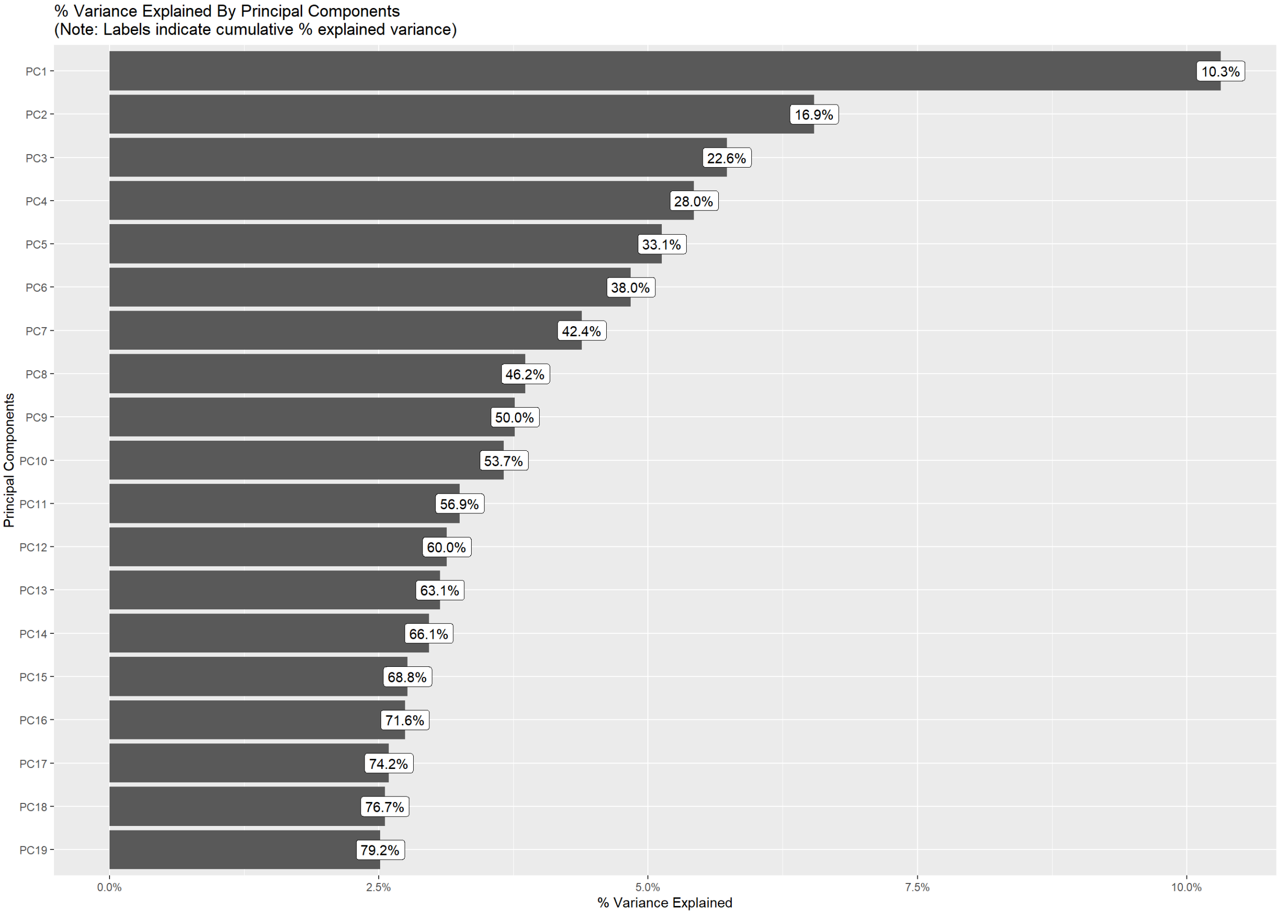
% Variance Explained By Principal Components
(Note: Labels indicate cumulative % explained variance)
Principal Components
% Variance Explained
PC1 10.3%
PC2 16.9%
PC3 22.6%
PC4 28.0%
PC5 33.1%
PC6 38.0%
PC7 42.4%
PC8 46.2%
PC9 50.0%
PC10 53.7%
PC11 56.9%
PC12 60.0%
PC13 63.1%
PC14 66.1%
PC15 68.8%
PC16 71.6%
PC17 74.2%
PC18 76.7%
PC19 79.2%
0.0%
2.5%
5.0%
7.5%
10.0%

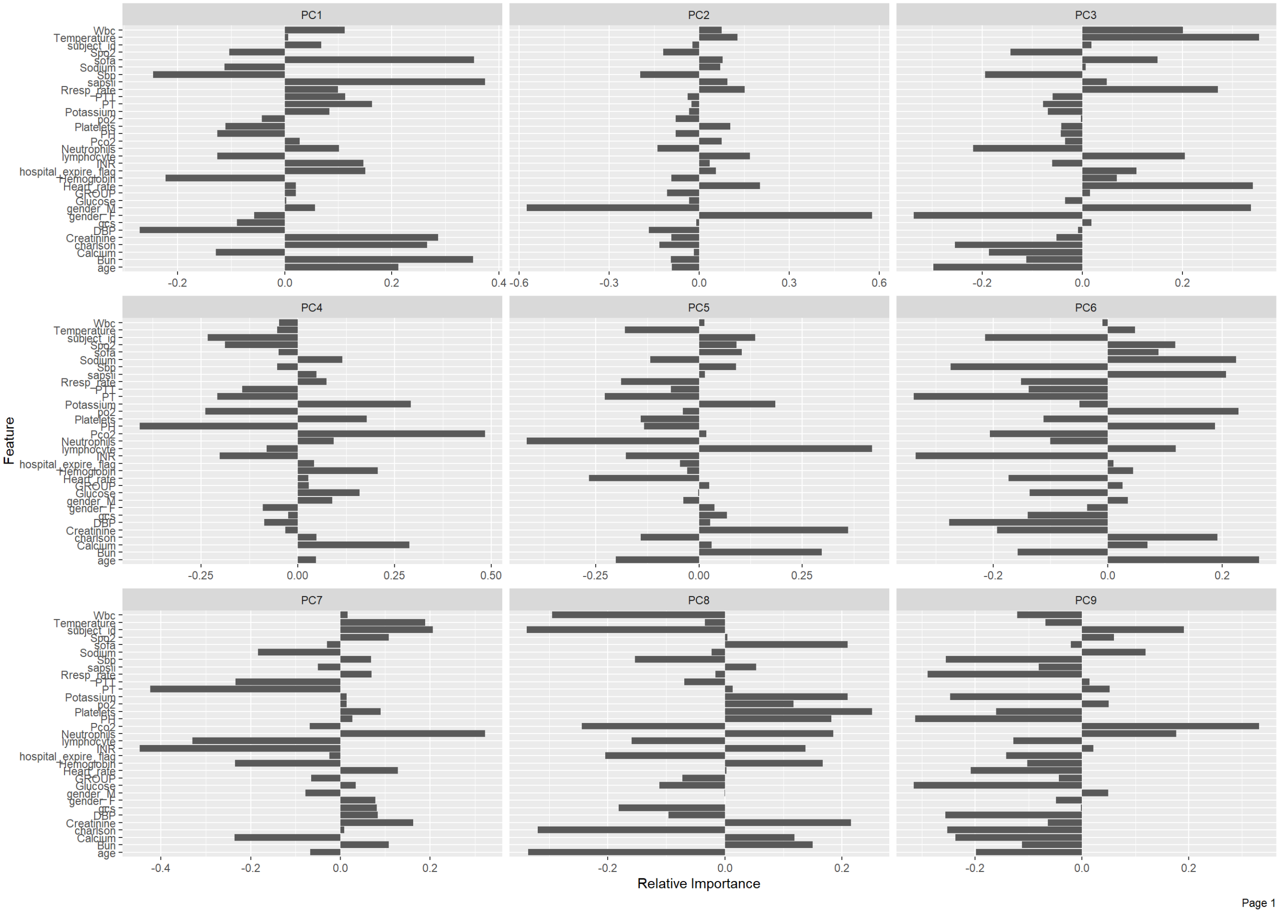
PC1
PC2
PC3
PC4
PC5
PC6
PC7
PC8
PC9
Feature
Relative Importance
Wbc
Temperature
subject_id
Spo2
sofa
Sodium
Sbp
sapsii
Rresp_rate
PTT
PT
Potassium
po2
Platelets
PH
Pco2
Neutrophils
lymphocyte
INR
hospital_expire_flag
Hemoglobin
Heart_rate
GROUP
Glucose
gender_M
gender_F
gcs
DBP
Creatinine
charlson
Calcium
Bun
age
Page 1

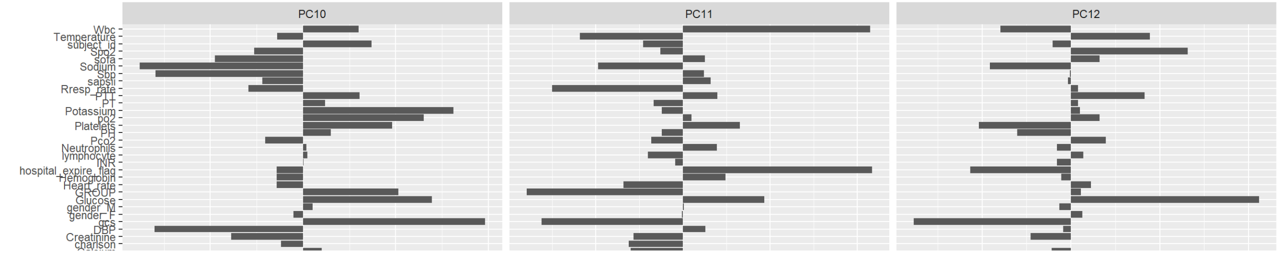
PC10
PC11
PC12

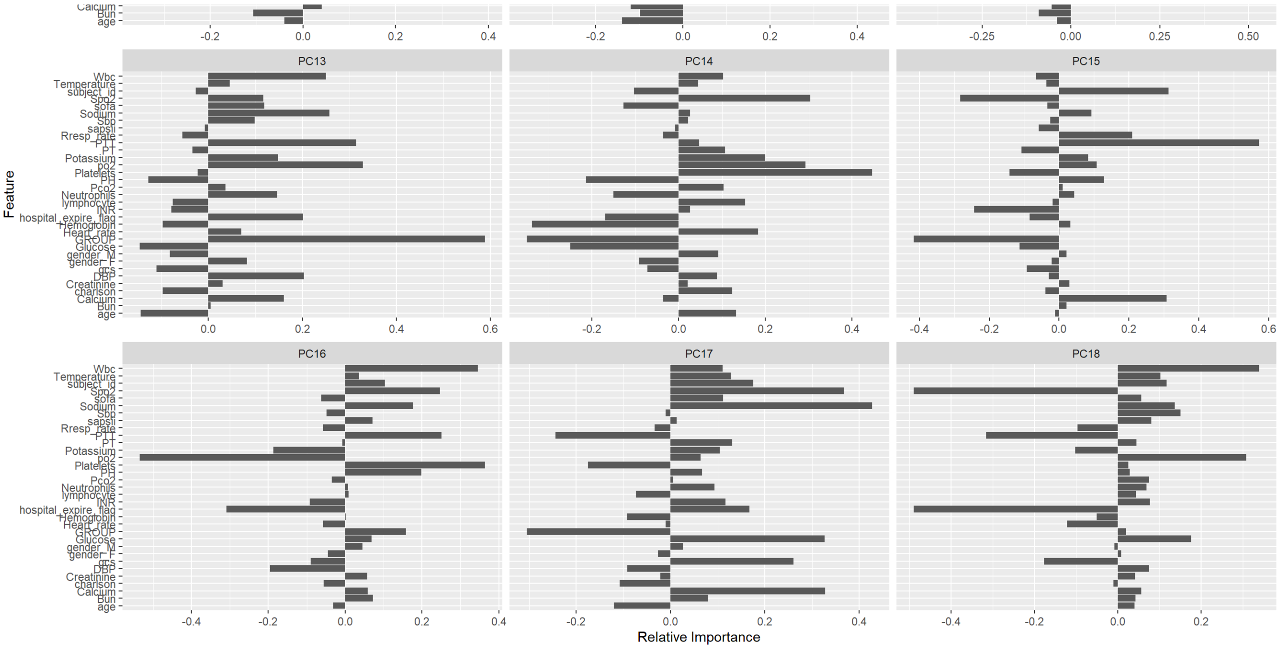
PC13
PC14
PC15
PC16
PC17
PC18
Feature
Relative Importance

Page 2

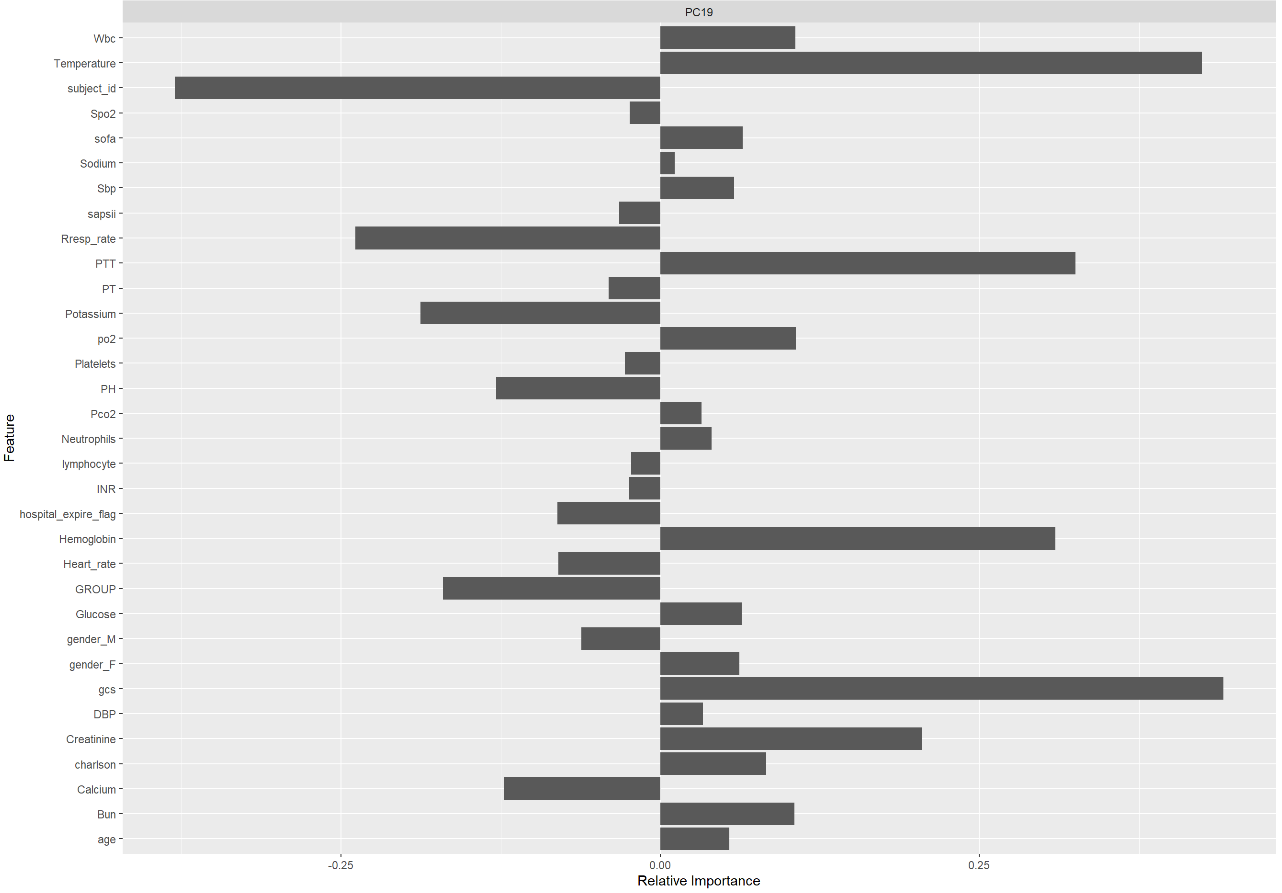
PC19
Wbc
Temperature
subject_id
Spo2
sofa
Sodium
Sbp
sapsii
Rresp_rate
PTT
PT
Potassium
po2
Platelets
PH
Pco2
Neutrophils
lymphocyte
INR
hospital_expire_flag
Hemoglobin
Heart_rate
GROUP
Glucose
gender_M
gender_F
gcs
DBP
Creatinine
charlson
Calcium
Bun
age
Feature
-0.25
0.00
0.25
Relative Importance

Page 3
